# Modulating the Optoelectronic Properties of Tripodal Fluorophores Through Fluorine‐Substituted Peripheral Phenyls

**DOI:** 10.1002/chem.202503470

**Published:** 2025-12-24

**Authors:** Milan Klikar, Eva Prokopová, Lefteris Laleas, Giorgos Soultse, Alexandros Katsidas, Zdeňka Růžičková, Zuzana Burešová, Oldřich Pytela, Filip Bureš, Mihalis Fakis

**Affiliations:** ^1^ Institute of Organic Chemistry and Technology Faculty of Chemical Technology University of Pardubice Pardubice Czechia; ^2^ Department of Physics University of Patras Patras Greece; ^3^ Department of General an Inorganic Chemistry Faculty of Chemical Technology University of Pardubice Pardubice Czechia

**Keywords:** C_3_‐symmetric fluorophores, DFT calculations, fluorination effect, intramolecular charge transfer (ICT), two‐photon absorption (2PA)

## Abstract

Fourteen novel tripodal fluorophores based on a central triphenylamine donor, electron‐rich, and polarizable divinylthiophene linker, and eight different fluorine‐based substituents have been designed and prepared via a straightforward four‐step sequence. Altering the peripheral F‐substitution has been demonstrated to largely affect their fundamental properties such as thermal robustness (210–420 °C), the LUMO energies (*E*_LUMO_ = −2.35 to −3.11 eV), the HOMO–LUMO gap (Δ*E* = 2.07–2.66 eV), and the absorption/emission maxima (*λ*_max_^A/E^ = 442–478/521–678 nm). The experimental data, corroborated by DFT calculations, further revealed twofold and tunable ICT employing both central triphenylamine and auxiliary thiophene donors, and the peripheral F‐substitution either boosting or switching‐off two‐photon absorption activity. Whereas the ─SF_5_ groups impart an exceptional cross‐section of 1930 GM, the ─COCF_3_ group may completely suppress the nonlinear optical response.

## Graphical abstract

C_3_‐symmetric D‐(π‐D‐π‐A)_3_ fluorophores featuring central triphenylamine donor, polarizable 2,5‐divinylthiophene linker, and a broad portfolio of fluorine‐based substituents show tunable (non)linear optical properties.

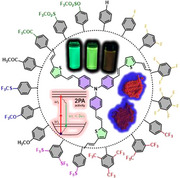

## 1 Introduction

The last couple of decades has demonstrated triphenylamine (TPA) as a robust electron‐donating building block widely utilized in the modular construction of push‐pull D‐π‐A chromophores (D = electron‐donor, π  =  conjugated system and A = electron‐acceptor) with numerous applications across optoelectronics [1, 2]. This situation stems from its direct synthetic availability, good electron‐donating and transporting capability, hole‐transporting properties, and facile functionalization [3]. The propeller‐shaped arrangement predisposes TPA to building octupolar chromophores with the centrifugal D‐(π‐A)_3_ architecture featuring various π‐branches and diverse peripheral electron‐acceptors [4]. Due to strong electronic coupling between the individual branches, tripodal TPA chromophores generally possess exceptional hyperpolarizability and a two‐photon absorption (2PA) cross‐section [3, 5, 6, 7, 8, 9] that have been utilized across materials science, biology, and medicine [10, 11, 12]. Two‐photon fluorescence microscopy [13], 2PA fluorescence cellular imaging [14, 15, 16, 17], and photodynamic therapy [18, 19, 20, 21] have become routinely employed noninvasive biomedical methods, while microfabrication [22, 23, 24], 3D data storage [25, 26], and up‐converted lasing [27] represent prominent material applications.

Fluorine is a relatively small atom with the highest electronegativity and low polarizability. It forms a high‐energy C─F bond (≈ 480 kJ·mol^−1^), which is strategically used to improve chemical and thermal stability of (per)fluorinated organic compounds as well as to modulate their biological activity. In this respect, fluorination primarily improves cell permeability, lipophilicity, solubility, and other pharmacological parameters [28, 29]. Furthermore, the strongly polarized C─F bond may affect molecular geometry, charge/electron distribution, and specific noncovalent interactions such as C─F∙∙∙H hydrogen bonding or π–π^F^ interactions [30, 31, 32]. Appending fluorine atom(s) to a π‐conjugated system substantially alters the electronic distribution and allows tuning of the HOMO/LUMO levels and semiconducting properties. Numerous reports have demonstrated enhanced performance and characteristics of fluorinated organic materials over their nonfluorinated analogues [33], for example, pronounced nonlinear optical (NLO) activity [30, 31, 32]. Single‐ (*σ*_p_(F) = 0.06), perfluoro‐ or trifluoromethyl‐substituted (−CF_3_; *σ*_p_ = 0.35) push‐pull systems are among the most widely utilized strategies [28, 29]. In contrast, the octahedral pentafluorosulfanyl [34] (─SF_5_; *σ*_p _ =  0.68) is much less employed, while the trifluoroacetyl (─COCF_3_; *σ*_p_ = 0.80) is a relatively scarce electron‐acceptor [35, 36, 37]. Trifluoromethoxy (─OCF_3_; *σ*_p_ = 0.35), trifluorosulfanyl (─SCF_3_; *σ*_p_ = 0.50), and trifluoromethyl sulfone (─SO_2_CF_3_; *σ*_p_ = 0.96) are known substituents in agrochemicals or pharmaceuticals [38, 39, 40]. The listed Hammett substituent constants (*σ*_p_) allow a rough evaluation of the electronic effects and point out the trifluoromethyl sulfone and trifluoroacetyl as very potent acceptors, superior to the common nitro group (*σ*_p_ = 0.78) [41]. While the moderate withdrawing strength of the ─CF_3_ and ─SF_5_ substituents stems from negative inductive effects and eventual hyperconjugation, the ─COCF_3_ or ─SO_2_CF_3_ substituents combine negative mesomeric and inductive effects. In addition, the less withdrawing ─OCF_3_ and ─SCF_3_ groups oscillate orthogonally to the appended aryl ring, thereby affecting π‐conjugation and electron distribution differently from the tetrahedral ─CF_3_ substituent [38, 42].

**FIGURE 1:**
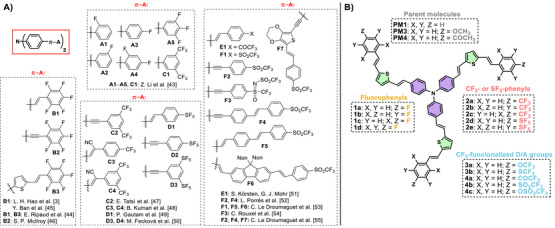
(A) Known tripodal TPA chromophores bearing varied π‐system and fluorine‐based acceptors; (B) Investigated tripodal TPA‐based fluorophores in this work.

Figure 1A summarizes the reported TPA chromophores bearing (poly)fluorinated acceptors **A–F**. Gradually fluorinated benzenes have been used as peripheral acceptors in TPA derivatives **A1**−**5** and **C1**, which proved useful as blue‐violet emitters or hole‐transporters of OLEDs [43]. Even better OLED characteristics were measured for pentafluorophenyl derivatives **B1** and **B3**, [44] while **B1** also showed noticeable third‐order optical nonlinearity [3, 45]. The pentafluorophenylethynyl pendant in **B2** allowed a substantial singlet oxygen production upon two‐photon excitation [46]. Bis(trifluoromethyl) derivative **C2** has been reported as a moderate luminophore for luminescent solar concentrators [47] and CF_3_‐capped cyanostilbenes **C3**−**C4** are AIEgens forming stable organogels [48]. The first pentafluorosulfanyl‐terminated TPA chromophore **D1**, reported in 2018 by Gautam et al., demonstrated a large 2PA cross‐section reaching 2000 GM [49], verifying the SF_5_ group as a potent electron‐withdrawing unit for efficient multiphoton absorbers. This year, we further demonstrated a selective employment of the electronic effects of the SF_5_ group in a series of tripodal fluorophores **D2−D3**. The SF_5_ group placed in the *para* position allows hyperconjugation with the TPA donor and imparts **D2** a 2PA cross‐section of 600 GM [50]. The trifluoroacetyl‐substituted derivative **E1** is capable of sensing amines [51], while the strongly withdrawing trifluoromethyl sulfone has been investigated by the research team of M. Blanchard–Desce [52, 53, 54, 55]. Property tuning in **F1**−**F7** was achieved by altering the π‐system, while the olefinic linker turned out to be more efficient as compared to the acetylenic one. When comparing **F4** and **F5**, the latter showed twofold increase in the 2PA activity, reaching 2000 GM. A comparison of the fluorinated sulfone and sulfoximinyl groups in **F2** and **F3** reveals the SO(NSO_2_CF_3_)CF_3_ group as “superacceptor” with the *σ*_p_ = 1.35 and enhanced 2PA activity (500 vs. 870 GM).

Despite increasing popularity, the current state‐of‐the‐art lacks a systematic study comprehensively comparing fluorine‐based substituents and their impact on the fundamental optoelectronic properties of push‐pull molecules, while only fragmented and limited reports available to date [56, 57, 58]. Hence, based on our continuing interest in octupolar fluorophores with 2PA activity [59, 60, 61, 62, 63] and following our recent study on SF_5_‐terminated TPA fluorophores [50], we present herein an extensive series of C_3_‐symmetric push‐pull molecules **1**−**4** (Figure 1B). These designed systems feature varying numbers (1−5) of the fluorinated groups at the periphery (─F, ─CF_3_, ─SF_5_, ─OCF_3_, ─SCF_3_, ─COCF_3_, ─SO_2_CF_3_, and ─OSO_2_CF_3_) placed at different position along the periphery (*ortho*, *meta*, *para*) to systematically investigate their optoelectronic properties. The central TPA donor and the peripheral acceptors are interconnected via a novel, highly polarizable 2,5‐divinylthiophene π‐linker possessing thiophene as an auxiliary donor, which is introduced into TPA chromophores for the first time. Both vinylene spacers enforce planarity, assure efficient intramolecular charge transfer (ICT), and, supposedly, also enhance 2PA activity. In addition, we present a series of parent molecules **PM1**−**3** and one linear analogue **2aL** to properly address the effect of fluorination and branching. The fundamental chemical, thermal, electrochemical, photophysical, and (non)linear optical properties of **1**−**4** and **PM1**−**3** were further corroborated by quantum‐chemical calculations.

## 2 Results and Discussion

### 2.1 Synthesis

**SCHEME 1:**
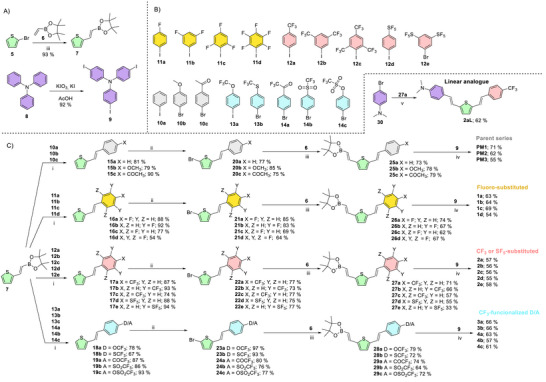
Synthetic pathways towards tripodal fluorophores **1–4**. (A) Synthesis of the key intermediates **7** and **9**. (B) Structures of the starting bromo or iodobenzenes bearing fluoro acceptors. (C) Straightforward four‐step reaction pathway towards all target fluorophores **PM1−3** and **1−4**: (i) **7** (1.1 eq), [PdCl_2_(PPh_3_)_2_] (0.02 eq), K_2_CO_3_ (1.5 eq), THF/H_2_O 4:1, 60 °C, 18 h; (ii) NBS (1.05 eq), CHCl_3_/AcOH 2:1, 80 °C, 18 h; (iii) vinylboronic acid pinacol ester **6** (1.5 eq), [Pd_2_(dba)_3_] (0.01 eq), P(*t*Bu)_3_ (0.05 eq), DIPEA (2 eq), toluene, 95 °C, 18 h; (iv) corresponding boronic acid pinacol ester (3.6 eq), [PdCl_2_(PPh_3_)_2_] (0.06 eq), K_2_CO_3_ (5 eq), THF/H_2_O 4:1, 60 °C, 18 h; (v) **27a** (1.1 eq), [PdCl_2_(PPh_3_)_2_] (0.02 eq), K_2_CO_3_ (1.5 eq), THF/H_2_O 4:1, 60 °C, 2 h.

To synthesize all target fluorophores **PM1**−**3** and **1**−**4** in a straightforward manner, four synthetic steps involving Suzuki‐Miyaura cross‐coupling (2×), selective bromination, and Heck olefination were employed (Scheme 1C). The substituted iodo‐ or bromobenzenes **10–14** (Scheme 1B) were used to introduce the fluorinated acceptors; intermediates **12c**, **12d**, and **9** were prepared according to the modified literature procedures (see the Supporting Information for more details) [52, 64, 65]. (*E*)‐2‐(Thiophen‐2‐yl)ethenylboronic acid pinacol ester **7**, a key intermediate allowing the facile and systematic construction of each branch, was smoothly prepared from 2‐bromothiophene **5** and the vinylboronic acid pinacol ester **6** via a Pd‐catalyzed Heck olefination in 93% yield (Scheme 1A) [66]. The fluorinated acceptors **10**−**14** were first cross‐coupled with the intermediate **7** affording the corresponding phenylethenylthiophenes **15**−**19** in satisfactory yields of ca. 60−90% (Scheme 1C). Selective bromination with NBS furnished the bromothiophenes **20**−**24** (64%–97%), which underwent subsequent Heck olefination with vinylboronic acid pinacol ester **6** under optimized reaction conditions ([Pd_2_(dba)_3_], P(*t*Bu)_3_, DIPEA, toluene) to give boronic acid pinacol esters **25**−**29** [55−80%; expect **27e** (33%)]. These esters (branches) and tris(4‐iodophenyl)amine **9** were finally threefold cross‐coupled to furnish the target fluorophores **PM1**−**3** and **1**−**4** (55−70%). Using 4‐bromo‐*N*,*N*‐dimethylaniline **30** allowed the construction of the linear analogue **2aL**, whose X‐ray analysis confirmed the exclusive *E*‐configuration of both double bonds within the divinylthiophene linker (Figure S1).

### 2.2 Thermal Properties

Thermal properties of **PM1**−**3** and **1**−**4** were determined by differential scanning calorimetry, the recorded temperatures of melting *T*_m_, glass transition *T*_g_ and thermal decomposition *T*_d_ are summarized in Table 1; the native DSC thermograms are provided in the SI. The studied compounds represent large tripodal structures with relatively high molecular weights (876–1632 g·mol^−1^), which hinder their crystallization and tend them to form amorphous glassy or semicrystalline solids upon precipitation from solution. Consequently, glass transitions of amorphs or broad melting processes of semicrystalline solids were primarily observed upon heating. A sharp endothermic melting, preceded by a cold crystallization at 137 °C, was recorded only for the pentafluorophenyl derivative **1d** at 223 °C. The semicrystalline compounds **PM2**, **1a**, **1c**, **2a**, **2b**, and **3a** underwent broad melting process (111−186 °C), but a reheating cycle was accompanied only by a glass transition, pointing to their complete amorphization. A distinctive and reversible glass transition (*T*_g_ = 89−147 °C) without any subsequent melting was observed for the remaining compounds. Whereas the tripodal **2a** showed only an exothermic irreversible peak at around 280 °C, the linear analogue **2aL** melted at 233 °C and exothermically decomposed up to 300 °C. The thermal degradation was generally recorded as either a vigorous or gradual exothermic process, with *T*_d_ values ranging from 210 to 420 °C. Sharing the same central TPA donor and the π‐system, the differences in thermal behavior are attributed to the peripheral fluoro‐acceptors. A rapid decomposition peak was revealed for **1d** (5×F), **2c** (3×CF_3_), **2d** (SF_5_), **3b** (SCF_3_), **4a** (COCF_3_), and **4c** (OSO_2_CF_3_), whereas a slow and gradual degradation was seen for molecules **PM1** (H), **PM2** (OCH_3_), **1a** (F), **2a** (CF_3_), **2b** (2×CF_3_), and **4b** (SO_2_CF_3_). Thermal stability is apparently enhanced by CF_3_ groups in the series **2a**−**c** (*T*_d_ = 290−420 °C), by the two SF_5_ groups in **2e** (*T*_d_ = 290 °C), and by the SO_2_CF_3_ pendants in **4b** (*T*_d_ = 340 °C). Consequently, the highest thermal robustness was measured for **2c** (*T*_d_  =  420 °C) bearing nine peripheral trifluoromethyl groups.

**TABLE 1:** Thermal and electrochemical properties of fluorophores **PM1**−**3** and **1−4**.

	Comp.	*T*_m_ [°C]^[^ ^a^ ^]^	*T*_g_ [°C]^[^ ^a^ ^]^	*T*_d_ [°C]^[^ ^b^ ^]^	*E*_1/2(ox1)_ [V]^[^ ^c^ ^]^	*E*_p(ox2)_ [V]^[^ ^d^ ^]^	*E*_1/2(red1)_ [V]^[^ ^c^ ^]^	Δ*E* ^CV^ [eV]^[^ ^c^ ^]^	*E*_HOMO_^CV^ [eV]^[^ ^f^ ^]^	*E*_LUMO_^CV^ [eV]^[^ ^f^ ^]^
Parent series	**PM1**	−	97	250	0.79	1.11	−1.81	2.60	−5.11	−2.51
	**PM2**	134	100	230	0.77	1.03	−1.89	2.66	−5.09	−2.43
	**PM3**	−	118	220	0.79	1.08	−1.62	2.41	−5.11	−2.70
Fluoro‐subst.	**1a**	154	102	230	0.89	1.23	−1.73	2.62	−5.21	−2.59
	**1b**	−	92	240	0.88	1.14	−1.64	2.52	−5.20	−2.68
	**1c**	186	−	250	0.88	1.15	−1.69	2.57	−5.20	−2.63
	**1d**	223	114	250	0.88	1.15	−1.52	2.40	−5.20	−2.80
CF_3_‐ and SF_5_‐subst.	**2a**	183	112	310	0.83	1.08	−1.68	2.51	−5.15	−2.64
	**2aL**	233	−	300	0.50	0.79	−1.97^[^ ^e^ ^]^	2.47	−4.82	−2.35
	**2b**	120	107	290	0.81	1.08	−1.64	2.45	−5.13	−2.68
	**2c**	−	−	420	0.82	1.10	−1.42	2.24	−5.14	−2.90
	**2d**	−	135	230	0.82	1.09	−1.56^[^ ^e^ ^]^	2.38	−5.14	−2.76
	**2e**	−	−	290	0.86	1.10	−1.45^[^ ^e^ ^]^	2.31	−5.18	−2.87
CF_3_‐funcional. D/A	**3a**	111	89	220	0.75	1.08	−1.84	2.59	−5.07	−2.48
	**3b**	−	92	230	0.76	1.05	−1.72	2.48	−5.08	−2.60
	**4a**	−	110	210	0.86	1.12	−1.21	2.07	−5.18	−3.11
	**4b**	−	147	340	0.87	1.14	−1.30	2.17	−5.19	−3.02
	**4c**	−	100	230	0.85	1.13	−1.59^[^ ^e^ ^]^	2.44	−5.17	−2.73

^[a]^ *T*_m_ = temperature of melting, *T*_g_ = temperature of glass transition (the point of intersection of a baseline and a tangent of DSC peak/step = onset).

^[b]^ *T*_d_  =  thermal decomposition (pyrolysis in N_2_ atmosphere).

^[c]^ *E*_1/2(ox1)_ and *E*_1/2(red1)_ are half‐wave potentials of the first oxidation and reduction, respectively, as measured by CV in THF containing 0.1 M Bu_4_NPF_6_; all potentials are given *vs*. SSCE, Δ*E* ^CV^  =  *E*_1/2(ox1)−_ *E*_1/2(red1)_.

^[d]^ *E*_p(ox2)_ is peak potential of the irreversible second (subsequent) oxidation.

^[e]^ Fully irreversible first reduction (*E*_p_^c^ value).

^[f]^ Experimentally deduced *E*_HOMO_^CV^ and *E*_LUMO_^CV^: −*E*_HOMO/LUMO_^CV^ = (*E*_1/2(ox1)_ + 0.036) or (*E*_1/2(red1)_ + 0.036) + 4.28 (vs. SCE) [67, 68]. The increment of +0.036 V corresponds to the difference between SCE (0.241 vs. SHE) and SSCE (0.205 vs. SHE)) [69].

### 2.3 Electrochemistry

The electrochemical behavior of the target fluorophores **PM1−3** and **1−4** was investigated in THF containing 0.1 M Bu_4_NPF_6_ using a three‐electrode cell by cyclic voltammetry (CV). The acquired electrochemical data are summarized in Table 1, while further experimental details, recorded CV diagrams, and comparison of the tripodal and linear analogues are given in the Supporting Information.

**FIGURE 2:**
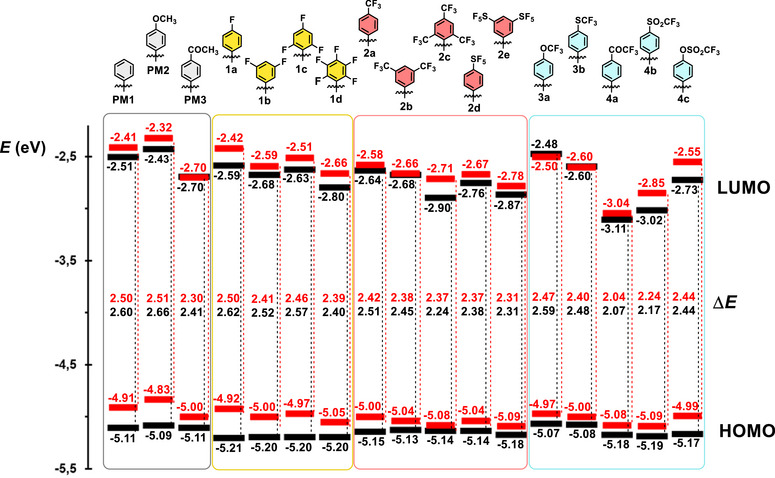
Energy level diagram of the electrochemically estimated (black) and the DFT‐calculated (red) HOMO/LUMO values for fluorophores **PM1−3** and **1−4**.

Oxidation was recorded as a multi‐electron process consisting of the first reversible one‐electron oxidation appearing as a distinguishable shoulder (*E*_1/2(ox1)_), presumably involving the TPA donor, followed by consecutive irreversible oxidations (*E*_p(ox2)_) associated with the thiophene auxiliary donors. This electrochemical behavior is consistent with the TPA‐(π‐Th‐π‐FluoroA)_3_ arrangement of the studied tripodal systems. Reduction is a multi‐electron process occurring over the peripheral withdrawing groups as well as the adjacent π‐system. The (quasi)reversible first reduction (*E*_1/2(red1)_) was recorded either as a shoulder or as a fully developed peak. However, the SF_5_‐ and F_3_CSO_2_O‐substituted compounds **2d−e** and **4c** exhibited fully irreversible first reductions (*E*_p(red1)_), consistent with our previous observation [50]. The multi‐electron transfer is related to the tripodal character and the number of appended fluorine atoms. For instance, incorporating two SF_5_ (**2e**) or three CF_3_ groups (**2c**) into each branch proportionally increases the number of transferred electrons during the consecutive reductions. The obtained half‐wave potentials of the first oxidation and reduction *E*_1/2(ox1/red1)_ for tripodal **PM1−3** and **1−4** were found within the range of 0.75 to 0.89 V and −1.21 to −1.89 V, respectively, and were further converted into energy range (eV) in order to experimentally estimate the HOMO/LUMO levels (Table 1). The energy level diagram in Figure 2 visualizes the experimentally estimated HOMO/LUMO energies and electrochemical gaps Δ*E* ^CV^. Taking the unsubstituted derivative **PM1** as the parent molecule (*E*_HOMO/LUMO_^CV^ = −5.11/−2.51 eV, Δ*E* ^CV^ = 2.60 eV), the HOMO level remains relatively steady across the series and principal changes are seen in the LUMO level upon further substitution. The LUMO is raised/lowered depending on whether an electron donor or acceptor is appended, e.g. **PM2** with a methoxy donor (*E*_LUMO_^CV^ = −2.43 eV) or **PM3** bearing an electron‐withdrawing acetyl group (*E*_LUMO_^CV^ = −2.70 eV). Attaching a single fluorine (**1a**), CF_3_ (**2a**) or SF_5_ (**1d**) into the *para* position gradually decreases the *E*_LUMO_^CV^ from −2.59 to −2.76 eV as a result of increasing negative inductive effect and eventually employing negative hyperconjugation [70]. Beginning with these mono‐substituted derivatives and gradually attaching additional fluorine‐containing substituents (**1a**→**1d**, **2a**→**2c** or **2d**→**2e**), the LUMO is further deepened, with the narrowest HOMO–LUMO gap observed for tris(trifluoromethyl) derivative **2c**. Nearly identical HOMO/LUMO levels were measured for the methoxy‐ and trifluoromethoxy‐substituted molecules **PM2** and **3a**, indicating persistent mesomeric electron‐donating behavior of both substituents. In contrast, a replacement of the chalcogens (O→S, **3a**→**3b**) lowers the LUMO energy due to the weaker donating ability of the SCF_3_ group. Comparison of the acetyl and trifluoracetyl derivatives **PM3** and **4a** reveals significantly pronounced electron‐withdrawing character of the latter. Hence, compound **4a**, bearing three peripheral COCF_3_ acceptors, possesses the narrowest HOMO–LUMO gap (Δ*E* ^CV^  =  2.07 eV), whereas the analogous sulfone **4b** was found to be a less effective mesomeric acceptor (Δ*E*_LUMO_^CV^ ∼ 0.1 eV). Contradictorily to **PM2** and **3a**, the trifluoromethyl sulfonate group in **4c** behaves rather as an electron‐acceptor and demonstrates electronic effects comparable to the SF_5_‐group in **2d**. These trends, particularly for the *para*‐substituted derivatives, correspond to the Hammett *σ*_p_^−^ constants [41] as demonstrated by the correlation present in Figure S24, and identify the ─SF_5_, ─SO_2_CF_3_, and ─COCF_3_ groups as among the most powerful electron‐withdrawing substituents within this series.

### 2.4 Linear Optical Properties

**FIGURE 3:**
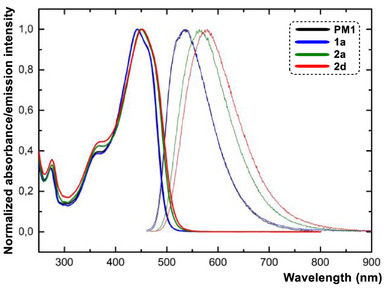
Normalized UV‐Vis absorption (bold line) and emission spectra (thin line) of fluorophores **PM1** (4‐H), **1a** (4‐F), **2a** (4‐CF_3_), and **2d** (4‐SF_5_) measured in THF (*c* ≈ 5×10^−6^ M).

Chromophores **PM1**−**3** and **1**−**4** are intensively orange or red solids, with their fundamental photophysical parameters listed in Tables 2 (THF), S2 (toluene), and S3 (acetonitrile). The absorption and emission spectra of representative **PM1**, **1a**, **2a**, and **2d** in THF are shown in Figure 3. Based on the Frenkel exciton model [8], three excited states are generally predicted for *C*_3_‐symmetric tripodal chromophores. However, the two low‐energy states (relative to the corresponding linear chromophore) are expected to be degenerate, while the high‐energy state possesses zero oscillator strength. Consequently, the absorption spectra of tripodal chromophores typically appear as a single band corresponding to that of the linear analogue [50, 73]. Fluorophores **PM1**−**3** and **1**−**4** exhibit a single absorption band (Figures S25–29), accompanied by a high‐energy shoulder at around 360 nm. When comparing the linear **2aL** (*λ*_max_^A/E^ = 429/583 nm) with the tripodal **2a** (*λ*_max_^A/E^ = 450/563 nm), both absorption and emission spectra are nearly identical (Figure S30), supporting the aforementioned assumption. The longest‐wavelength absorption maxima of the tripodal fluorophores **PM1**−**3** and **1**−**4** lie between 442 and 478 nm, with molar absorption coefficients in the range of 105−180×10^3^ M^−1^cm^−1^. This contrasts with the linear derivative **2aL** (*ε*_max_ = 55.6 ×10^3^ M^−1^cm^−1^). Thus, increasing the number of chromophoric units enhances the absorption, which is a typical feature of linear vs. tripodal systems [73]. In agreement with the electrochemical measurements, the observed red/blue shifts in the absorption maxima arise from structural variations in the fluorine‐based peripheral substituents. The same trends are obeyed as can be demonstrated by a tight correlation of the optical (1240/*λ*_max_^A^) and electrochemical gaps (Δ*E*), see Figures S35 in the Supporting Information. Fluorophore **2c** features different photophysical behavior, including blue‐shifted absorption maxima, substantially quenched fluorescence in THF, the most red‐shifted emission band in toluene, and intense solid‐state emission. This behavior is likely associated with a nonplanar arrangement of the terminal phenyl ring caused by *ortho*‐substitution with bulky CF_3_‐groups [74]. The most red‐shifted CT bands were recorded for **4a** and **4b** (478 and 473 nm), bearing ─COCF_3_ and ─SO_2_CF_3_ powerful acceptors. Only minor solvatochromism was detected in the absorption spectra (Figure S31−34), which is a typical feature of push–pull chromophores [75].

**TABLE 2:** Linear and nonlinear optical data of fluorophores **PM1**−**3** and **1−4** in THF.

		Solution							Powder
Comp.		*λ*_max_^A^ [nm/eV]^[^ ^a^ ^]^	*ε*_max_^A^ [10^3^ M^−1^cm^−1^]^[^ ^a^ ^]^	*λ*_max_^E^ [nm/eV]^[^ ^a^ ^]^	<τ> [ns]^[^ ^a^ ^]^	*Φ* ^F^ [‐]^[^ ^b^ ^]^	Stokes shift [cm^−1^/eV]	*δ*_2PA_/*λ*_2PA_ [GM/nm]^[^ ^c^ ^]^	*λ*_max_^E^ [nm]^[^ ^d^ ^]^
Parent series	**PM1**	443/2.80	181	535/2.32	1.61	0.50	3880/0.48	890/740	^[^ ^f^ ^]^
	**PM2**	443/2.80	159	521/2.38	1.39	0.56	3380/0.42	850/740	^[^ ^f^ ^]^
	**PM3**	456/2.72	110	585/2.12	1.90	0.47	4840/0.60	550/730	650
Fluoro‐subst.	**1a**	442/2.81	134	535/2.32	1.65	0.46	3900/0.49	910/740	^[^ ^f^ ^]^
	**1b**	448/2.77	117	562/2.21	1.93	0.49	4530/0.56	800/740	^[^ ^f^ ^]^
	**1c**	446/2.78	105	550/2.25	1.92	0.54	4240/0.53	610/740	^[^ ^f^ ^]^
	**1d**	457/2.71	146	587/2.11	2.23	0.52	4850/0.60	810/740	^[^ ^f^ ^]^
CF_3_‐ and SF_5_‐subst.	**2a**	450/2.76	130	563/2.20	1.91	0.49	4460/0.55	670/740	^[^ ^f^ ^]^
	**2aL**	429/2.89	55.6	583/2.13	^[^ ^h^ ^]^	^[^ ^h^ ^]^	6160/0.76	^[^ ^h^ ^]^	^[^ ^f^ ^]^
	**2b**	453/2.74	119	583/2.13	2.06	0.48	4920/0.61	560/740	^[^ ^f^ ^]^
	**2c**	443/2.80	175	^[^ ^e^ ^]^	0.32	^[^ ^e^ ^]^	^[^ ^e^ ^]^	^[^ ^g^ ^]^	622
	**2d**	452/2.74	138	578/2.15	1.94	0.45	4820/0.60	530/740	^[^ ^f^ ^]^
	**2e**	458/2.71	120	603/2.06	1.90	0.36	5250/0.65	1930/740	617
CF_3_‐funcion. D/A	**3a**	444/2.79	129	545/2.28	1.76	0.48	4170/0.52	1250/740	^[^ ^f^ ^]^
	**3b**	452/2.74	118	568/2.18	1.87	0.49	4520/0.56	780/750	^[^ ^f^ ^]^
	**4a**	478/2.59	163	678/1.83	0.34	0.06	6170/0.77	^[^ ^g^ ^]^	^[^ ^f^ ^]^
	**4b**	473/2.62	124	665/1.86	0.64	0.11	6100/0.76	1170/730	710
	**4c**	447/2.77	134	553/2.24	1.83	0.49	4290/0.53	710/740	^[^ ^f^ ^]^

^[a]^ Measured in THF (Dimroth–Reichardt polarity parameter $E_{T}^{N}$ = 0.207 [71]) at concentration ≈ 5×10^−6^ M; emitted at the absorption maximum wavelength.

^[b]^ Fluorescence quantum yield (±10%) determined relative to perylene as a standard (*Φ* ^F^ = 0.94 in cyclohexane) [72].

^[c]^ Measured in THF.

^[d]^ Emitted at the absorption maximum wavelength determined in THF solution.

^[e]^ Non‐emissive in THF.

^[f]^ Low intensity of emission in solid state.

^[g]^ Low 2PA excitation fluorescence signal.

^[h]^ Not measured.

**FIGURE 4:**
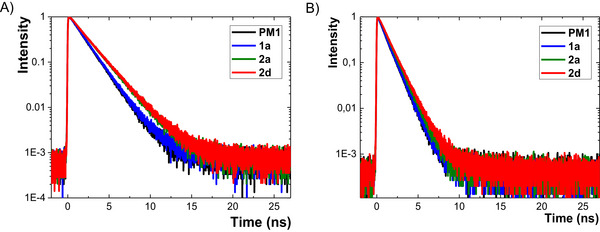
Fluorescence decays in the ns timescale for fluorophores **PM1** (4‐H), **1a** (4‐F), **2a** (4‐CF_3_), and **2d** (4‐SF_5_) measured in A) THF and B) toluene (*c* ≈ 5×10^−6^ M).

The target molecules showed a single broad emission band in THF and ACN, while an apparent low‐energy shoulder was recorded in nonpolar toluene due to vibronic transitions commonly found in nonpolar media [76]. Compared to the absorption spectra, the emission maxima of **PM1**−**3** and **1**−**4** span a broad range (521 to 678 nm) and are obviously much more influenced by the structural aspects, similarly to the aforementioned electrochemical data (see Figure S36 for the correlation). This implies a greater polarization/stabilization of the excited state, arising from considerable structural/geometrical reorganization compared to the ground state. As expected, this effect is most pronounced for the nonplanar **2c**, which showed the largest Stokes shift above 6100 cm^−1^ in toluene (Table S2). In general, the emission maximum shifts bathochromically with an increasing number of fluorine atoms/fluorine‐based substituents or upon replacing CH_3_ by CF_3_ groups. A positive solvatochromism observed in more polar solvents (ACN) further confirms the polar nature of the excited state, however, the stabilization is accompanied by a diminished fluorescence intensity. In THF, the fluorescence quantum yields (*Φ* ^F^) range within a narrow interval of 0.36–0.56 and decrease significantly upon attaching powerful withdrawing substituents (−COCF_3_ and −SO_2_CF_3_), which enhance ICT, as in **4a**−**b** (*Φ* ^F^ = 0.06−0.11). A similarly narrow range of *Φ* ^F^ values (0.44−0.54) was found in toluene. The excited states of **PM1**−**3** and **1**−**4** were further studied by fluorescence‐decay measurements in toluene and THF (Figures 4 and S37–46 and Tables 2 and S4,S5). The average fluorescence lifetimes *τ* range from 0.3 to 2.2 ns (THF, Table 2) and depend on both structural changes and solvent polarity. Figure 4 presents the decay profiles for the representative fluorophores **PM1**, **1a**, **2a**, and **2d** in toluene and THF showing extended lifetimes for chromophores with a larger number of fluorine atoms and fluorine‐based substituents. On the contrary, replacing CH_3_ by CF_3_ in the acetyl group (**PM3**→**4a**) results in a substantial decrease in the lifetime (1.9→0.34 ns), while it increases again for **4b** (0.64 ns, −SO_2_CF_3_) and **4c** (1.83 ns, −OSO_2_CF_3_). However, **4a–c** exhibited even longer lifetimes in toluene than the parent systems **PM2**/**3** (Table S5).

Fluorophores **PM3**, **2c**, **2e**, and **4b**, all bearing powerful withdrawing termini, also showed a noticeable solid‐state emission (Table 2, Figures S47−48). Compared to their solution emissions, the solid‐state emission spectra are red‐shifted and display low‐energy shoulders, indicating significant intermolecular interactions and possible formation of excimers [77]. Fluorophore **2c** exhibits the strongest solid‐state intensity, attributed to the *ortho*‐oriented CF_3_‐groups, which affect molecular packing in the solid state [78]. The nonplanar arrangement restricts an intramolecular rotation and opens aggregation‐induced emission [74].

### 2.5 Theoretical DFT Calculations

**FIGURE 5:**
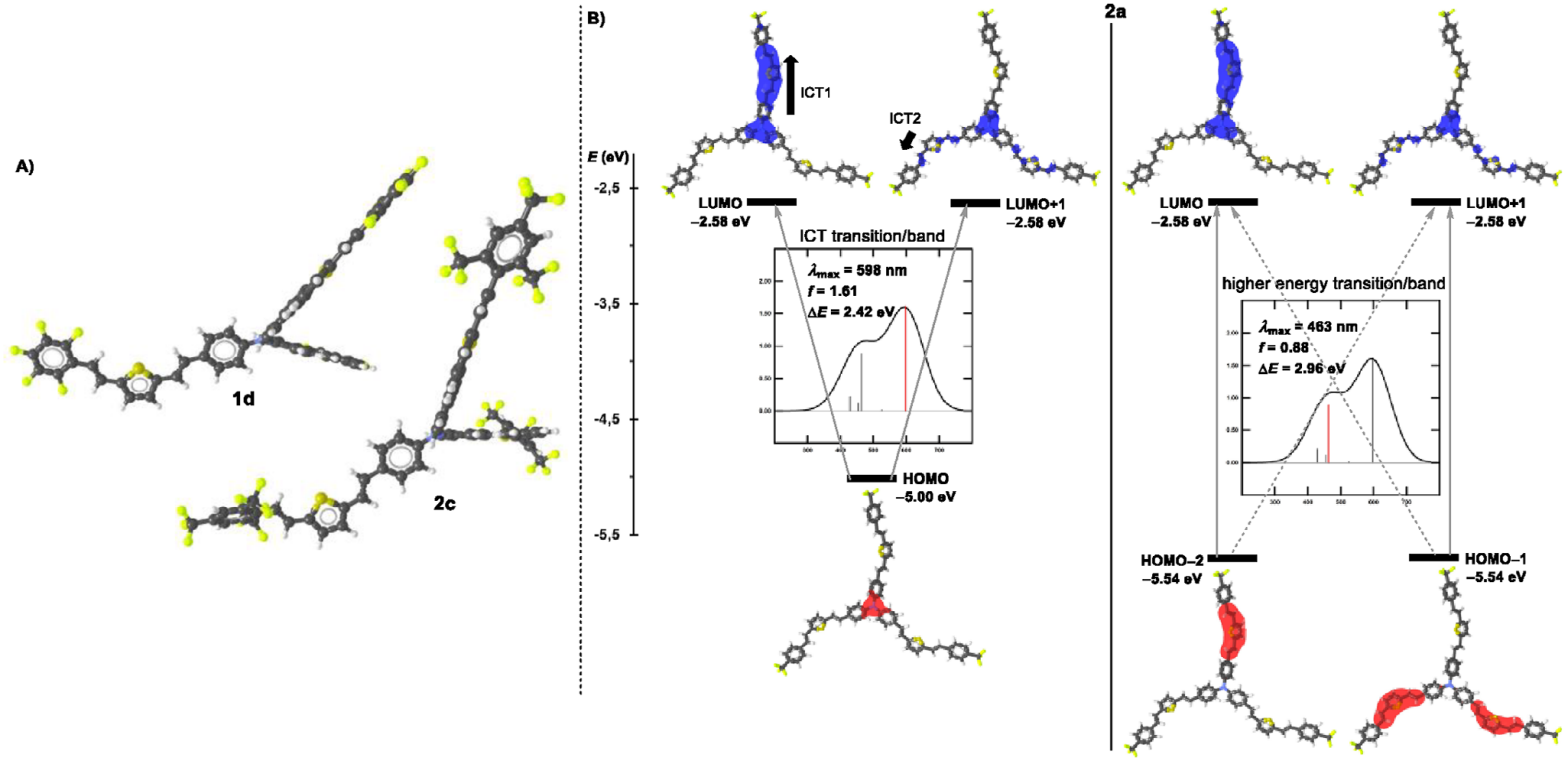
(A) Optimized geometries of fluorophores **1d** and **2c** calculated using DFT B3LYP/6‐311+G(2d,p) level; color of atoms: carbon—black, hydrogen—white, nitrogen—blue, sulfur—yellow, fluorine—yellow‐green. (B) The principal electron transitions, FMOs and DFT‐calculated parameters of the representative trifluoromethyl derivative **2a** [the HOMO(−1,−2) are shown in red, the LUMO(+1,+2) in blue; the vertical lines in the calculated absorption spectra represent oscillator strengths (f)].

The spatial, electronic and optical properties of the target fluorophores **PM1**−**3** and **1**−**4** were further predicted using DFT methods implemented in Gaussian16 W software package [79]. The initial geometries, frontier molecular orbital energies, and ground‐state dipole moments *µ*_g_^DFT^ were optimized/calculated by the DFT B3LYP/6−311+G(2d,p) method in THF (Table 3). Theoretical electronic absorption spectra were calculated at the TD‐DFT (nstates = 8) level using both B3LYP/6‐311++G(2d,p) and CAM‐B3LYP/6‐311++G(2d,p) functionals in THF, and the corresponding *λ*_max_^DFT^ values were extracted for both methods, see the Supporting Information for more details. The optimized geometry of the representative fluorophore **1d** (Figure 5A) reveals completely planar branches appended to the propeller‐shaped TPA, this structural feature is consistent across other molecules in the series. However, it contrasts with the twisted terminal phenyls (dihedral angle ∼ 50 °) of **2c** bearing the bulky trifluoromethyl groups. This geometrical distinction further supports our aforementioned conclusions drawn from the experimental photophysical data. For a comparison between the tripodal and linear analogues **2a**/**2aL**, see the Supporting Information.

**TABLE 3:** The DFT calculated data of fluorophores **PM1**−**3** and **1−4**.

	Comp.	*E*_HOMO_^DFT^ [eV]^[^ ^a^ ^]^	*E*_HOMO−1(−2)_^DFT^ [eV]^[^ ^a^ ^]^	*E*_LUMO(+1)_^DFT^ [eV]^[^ ^a^ ^]^	*E*_LUMO+2_^DFT^ [eV]^[^ ^a^ ^]^	Δ*E* ^DFT^ [eV]^[^ ^a^ ^]^	*µ*_g_^DFT^ (D)^[^ ^a^ ^]^	*λ*_max_^DFT^ (nm/eV)^[^ ^b^ ^]^	*λ*_max_^DFT^ (nm/eV)^[^ ^c^ ^]^
Parent series	**PM1**	−4.91	−5.40	−2.41	−2.20	2.50	1.4	577/2.15	451/2.75
	**PM2**	−4.83	−5.24	−2.32	−2.10	2.51	2.6	575/2.16	454/2.73
	**PM3**	−5.00	−5.53	−2.70	−2.57	2.30	0.8	623/1.99	467/2.66
Fluoro‐subst.	**1a**	−4.92	−5.41	−2.42	−2.22	2.50	3.4	576/2.15	462/2.68
	**1b**	−5.00	−5.54	−2.59	−2.40	2.41	4.6	550/2.25	430/2.88
	**1c**	−4.97	−5.50	−2.51	−2.32	2.46	2.6	572/2.17	446/2.78
	**1d**	−5.05	−5.62	−2.66	−2.49	2.39	5.6	583/2.13	446/2.78
CF_3_‐ and SF_5_‐subst.	**2a**	−5.00	−5.54	−2.58	−2.41	2.42	9.4	598/2.07	459/2.70
	**2aL**	−4.98	−5.96(−7.01)	−2.38(−1.25)	−0.84	2.60	10.9	534/2.32	448.2.77
	**2b**	−5.04	−5.61	−2.66	−2.50	2.38	9.0	593/2.09	451/2.75
	**2c**	−5.08	−5.72	−2.71	−2.58	2.37	4.6	608/2.04	−^[^ ^d^ ^]^
	**2d**	−5.04	−5.60	−2.67	−2.51	2.37	9.2	600/2.07	453/2.74
	**2e**	−5.09	−5.69	−2.78	−2.64	2.31	10.5	623/1.99	465/2.67
CF_3_‐funcional. D/A	**3a**	−4.97	−5.49	−2.50	−2.31	2.47	3.5	587/2.11	455/2.73
	**3b**	−5.00	−5.54	−2.60	−2.44	2.40	8.6	596/2.08	456/2.72
	**4a**	−5.08	−5.63	−3.04	−2.96	2.04	10.5	698/1.78	488/2.54
	**4b**	−5.09	−5.67	−2.85	−2.73	2.24	13.3	621/2.00	456/2.72
	**4c**	−4.99	−5.51	−2.55	−2.36	2.44	3.8	572/2.17	441/2.81

^[a]^ Calculated using the DFT B3LYP/6−311+G(2d,p) level in THF.

^[b]^ Calculated using the TD‐DFT (nstates = 8) B3LYP/6−311++G(2d,p) level in THF.

^[c]^ Calculated using the TD‐DFT (nstates = 8) CAM‐B3LYP/6−311++G(2d,p) level in THF.

^[d]^ Calculation failed.

The calculated HOMO and LUMO energies *E*_HOMO/LUMO_^DFT^ (Figure 2) follow the same trends as seen by the CV measurements, and the calculated *E*_LUMO_^DFT^ and HOMO–LUMO gaps correlate tightly with the experimental values (Figures S50−S51). As a general trend, the steady HOMO and the varied LUMO were observed upon changing the terminal fluorine‐based substituents. Nearly identical energies were calculated for the HOMO−1/−2 and the LUMO/+1, indicating these pairs of FMOs to be degenerate (Figure 5B), which is in accordance with previous observations [50, 80].

Visualization of the FMOs in the representative chromophore **2a** (Figure 5B; see also Figures S52−S60 for other derivatives) shows that the HOMO is localized on the central TPA unit, while the HOMO−1 is spread over the auxiliary thiophene donor and the adjacent π‐system of one branch. The degenerate HOMO−2 displays an analogous distribution over the remaining two branches. The unoccupied orbitals display heterogenous distributions depending on the electron‐withdrawing strength of the given acceptor. Fluorophores with weak or absent acceptors possess the LUMO+2 spread over both the thiophene and the adjacent π‐system across all three branches (except **2c**). Increasing acceptor character shifts LUMO+2 toward the terminal phenyls (**2b**−**e**) or directly onto the mesomeric acceptors (**PM3**, **4a**−**b**). The LUMO+1 is transitioned from the central TPA donor (**PM2**, **1a**, **2a**, and **3b**) to individual arm (**PM1**, **1b**−**c**, **2d**−**e**, and **3a** or **1d**, **2b**, **2c**, and **4c**) and finally to the terminal strong acceptors (**PM3**, **4a**, **4b**), depending on withdrawing strength. The LUMO itself exhibits a nearly identical distribution as the degenerate LUMO+1 across the second (or third) branch(es), a such splitting is a typical feature of tripodal D(–π–A)_3_ scaffolds [61]. Hence, the degree of charge separation in the studied molecules can be effectively tuned by varying the electron‐withdrawing ability of the terminal substituents.

The UV‐Vis absorption spectra of target fluorophores **PM1**−**3** and **1**−**4** were predicted using two different functionals, namely B3LYP and CAM‐B3LYP in THF (Table 3 and Figures S61−S68). While the CAM‐B3LYP‐calculated spectra align well with the experimental bands, the B3LYP‐derived spectra obey the shape of the experimental ones but are shifted bathochromically. However, when correlating the experimental and theoretical *λ*_max_ values, the CAM‐B3LYP‐derived longest‐wavelength absorption maxima showed no meaningful correlation, whereas the B3LYP‐calculated data fit well with the experimental values (Figure S69), including the high‐energy shoulder. Transition analysis (Figure 5B) revealed that the longest‐wavelength band (534–698 nm) originates from two principal transitions: HOMO → LUMO or HOMO → degenerate LUMO+1, both exhibiting the same oscillator strength. The high‐energy band arises from four transitions between the degenerate HOMO−1/HOMO−2 to the degenerate LUMO/LUMO+1. All these transitions possess the same oscillator strengths (roughly half those responsible for the CT‐band), correspond well to the experimental data, and are largely neglected by the CAM‐B3LYP functional. Altogether, the DFT predicted outcomes are in line with the TPA‐(π‐Th‐π‐FluoroA)_3_ structural arrangement, exhibiting two principal ICTs originating from both the TPA and thiophene donors (Figure 5B).

### 2.6 Two‐Photon Absorption Properties

**FIGURE 6:**
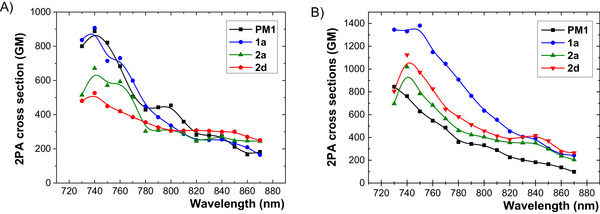
Two‐photon absorption spectra for fluorophores **PM1** (4‐H), **1a** (4‐F), **2a** (4‐CF_3_), and **2d** (4‐SF_5_) measured in (A) THF and (B) toluene.

With respect to their adjustable ICT character and tripodal arrangement, the two‐photon absorption properties of **PM1**−**3** and **1**−**4** were further examined by a two‐photon excited fluorescence (TPEF) method in THF and toluene (Tables 2 and S2 and Figures 6 and S70−S79). Except **2c** and **4a**, the investigated fluorophores generally exhibited good to excellent 2PA performance, with 2PA cross sections around 1000 GM. When going from the unsubstituted derivative **PM1** to the monofluoro‐substituted derivative **1a**, the peak 2PA cross‐section at 740 nm in THF slightly increases from 890 to 910 GM; however, the effect is far more pronounced in toluene (1380 GM at 750 nm). Further addition of the fluorine atoms (**1a**→**1d**) did not improve the 2PA activity. Attaching the trifluoromethyl along with the *para*‐pentafluorosulfanyl substituents proved rather detrimental, whereas the introduction of two *meta*‐positioned SF_5_ groups together with the polarizable branches in **2e** yields an exceptional 2PA cross‐section of 1930 GM in THF (Table 2). When comparing **2e** to structurally related chromophores, bearing olefinic [49] and acetylenic units [50] and possessing markedly different cross‐sections of around 2000 and 500 GM, the influence of a planar chromophoric unit and a cumulation effect of two SF_5_ groups must be highlighted. Further increasing the ICT by employing strong mesomeric acceptors such as COCF_3_ (**4a**) results in a dramatic drop of the TPEF signal, falling below a detectable limit, whereas the trifluoromethyl sulfone derivative **4b** still displayed a noticeable cross‐section of 1170 GM. Interestingly, derivative **3a**, bearing three pseudo‐donating OCF_3_ groups, exhibited an enhanced *δ*_2PA_ of 1250 GM, surpassing the 850 GM observed for its methoxy‐substituted analogue **PM2**. This further demonstrates that balancing the D/A behavior of the OCF_3_ group through a combination of −I and +M effects may represent a viable strategy for increasing two‐photon activity. Our TPEF measurements clearly show that a pronounced ICT tends to suppress the two‐photon absorption cross‐section, particularly in non‐polar solvents where stabilization of radiative ICT states is diminished. Chromophores with strong mesomeric acceptors (**PM3**, **4a**, **4b**) show the lowest 2PA cross‐sections in toluene, whereas analogues containing none/moderate acceptors (**PM1**−**PM2**, **1a**, **2a**, **2d, 3a**−**b**) maintain higher radiative efficiency, despite lacking octopolar features. Hence, the structural arrangement (D(–π–A)_3_ or D(–π–D)_3_, planarization and composition of the chromophoric units, properly adjusting the extent of ICT, and selecting an appropriate environment seem to be proper tools to design organic absorbers with admirable 2PA activity. Considering the whole portfolio of studied fluorine‐based peripheral moieties, the 3,5‐diSF_5_‐substituted chromophore **2e** stands out as particularly exceptional.

## 3 Conclusion

Starting from the central TPA donor, we constructed a comprehensive series of propeller‐shaped, C_3_‐symmetric tripodal push–pull fluorophores by linking polarizable 2,5‐divinylthiophene units with peripheral fluorinated phenyl rings acting as weak‐to‐strong electron acceptors. In addition to the tripodal push‐pull chromophores **1**−**4**, the series was complemented by one linear (**2aL**) and three non‐fluorinated parent compounds (**PM1−3**), allowing a thorough comparison across the entire library. The systematic structural variation allowed controlled modulation of the intramolecular charge‐transfer (ICT) character, providing a platform to evaluate how subtle changes in the degree of fluorination affect the linear and nonlinear photophysical properties. The target fluorophores were obtained as orange‐to‐red amorphous or semicrystalline solids with high solubility in solvents of medium and low polarity and with distinct glass transitions or melting processes, reflecting their amorphous or semicrystalline nature. Thermal stability was generally high, with the ─CF_3_ and ─SO_2_CF_3_‐functionalized derivatives **2a**−**c** and **4b** displaying the greatest robustness. In general, peripheral F‐functionalization can greatly enhance thermal stability. Electrochemical measurements revealed relatively steady HOMO localized at the central TPA unit, while the LUMO energies were strongly modulated by the electron‐withdrawing strength of the peripheral substituents. According to the LUMO energies, the electron withdrawing power of the investigated (per)fluoro groups follows the order: OCF_3_ ≈ H < ─SCF_3_ ≈ ─F < ─CF_3_< ─SF_5_ ≈ ─OSO_2_CF_3_< ─SO_2_CF_3_< ─COCF_3_. Thus, the trifluoromethoxy group acts as an electronically neutral substituent, whereas the trifluoroacetyl proved to be a highly potent electron acceptor. The steady‐state absorption spectra were largely insensitive to structural variations, whereas the emission properties exhibited a strong dependence on the extent of ICT and solvent polarity, with emission maxima tunable from 521 to 678 nm in THF and fluorescence lifetimes ranging from 0.3 to 2.2 ns. Hence, a radiative relaxation can be effectively controlled through the peripheral substitution. The twisted arrangement of the *ortho*‐oriented CF_3_ groups in **2c** imparts different photophysics in solution and enhanced solid‐state fluorescence, further illustrating the critical role of substituent orientation. The experimentally measured two‐photon absorption cross‐sections ranged from 500 to 1300 GM in THF and toluene, while fluorophore **2e**, bearing two meta‐positioned ─SF_5_ groups, demonstrated an exceptional 2PA cross‐section of 1930 GM. In contrast, the two‐photon activity of the trifluoroacetyl derivative **4a** was nearly zero in THF. Thus, taking the steady central donor and the conjugated system, the peripheral F‐substitution can either boost or quench the NLO activity through controlling the extent of ICT. These results demonstrate that the interplay between the polarizability of the particular chromophoric units, the substituent electronic nature, and the molecular symmetry is crucial for enabling efficient two‐photon transitions. This study establishes general principles showing that a careful selection and positioning of the F‐substituents can finely modulate ICT and tailor both linear and nonlinear photophysical properties of TPA‐centered tripodal fluorophores with perspective applications across 2PA bioimaging, optical data storage, and other nonlinear photonic technologies, where a precise control over the ICT and the two‐photon response is essential.

## Conflicts of Interest

The authors declare no conflict of interest.

## Supporting information



The authors have cited additional references within the Supporting Information [81, 82, 83, 84, 85, 86, 87, 88, 89, 90, 91, 92, 93, 94, 95, 96, 97]. The data supporting this article have been included as part of the SI, and the dataset available at https://doi.org/10.6084/m9.figshare.30646283. Crystallographic data for **2aL** has been deposited at the [CCDC] under [2502796] and can be obtained from [https://www.ccdc.cam.ac.uk].

**Supporting File 1**: chem70633‐sup‐0001‐SuppMat.pdf.

**Supporting File 2**: chem70633‐sup‐0002‐Data.zip.

## Data Availability

The data that support the findings of this study are openly available in Figshare at https://doi.org/10.6084/m9.figshare.30646283, reference number 30646283.
