# Supplementary material for: Modulating the Optoelectronic Properties of Tripodal Fluorophores Through Fluorine‐Substituted Peripheral Phenyls
Source: Chemistry. 2025 Dec 24;32(6):e03470. doi: 10.1002/chem.202503470 (PMC12887642; doi:10.1002/chem.202503470)
Supplement: Supplementary file 1 — The authors have cited additional references within the Supporting Information [81, 82, 83, 84, 85, 86, 87, 88, 89, 90, 91, 92, 93, 94, 95, 96, 97]. The data supporting this article have been included as part of the SI, and the dataset available at https://doi.org/10.6084/m9.figshare.30646283. Crystallographic data for 2aL has been deposited at the [CCDC] under [2502796] and can be obtained from [https://www.ccdc.cam.ac.uk]. [file CHEM-32-e03470-s001.pdf]

Supporting Information

Table of contents **Page**

1. Experimental section 2
2. Solubility and characterization of target fluorophores 23
3. X-Ray analysis 24
4. Thermal properties 26
5. Electrochemistry 35
6. Linear optical properties 40
7. DFT calculations 55
8. Non-linear absorption properties (2PA) 71
9. ^{1}H, ^{13}C and ^{19}F NMR spectra 76
10. HR-MALDI-MS spectra 104
11. Literature 113

1. Experimental section

General Methods

All target chromophores **1–4** and **PM1–4** are new compounds. All intermediates **16–29** (except **16a**, **17a**, **20a**) are completely new compounds as well. The starting 2-bromothiophene **5**, triphenylamine **8**, 4-bromo-*N*,*N*-dimethylaniline **30**, and all bromo or iodobenzenes **10–14** (expect **12c–d**) are commercially available. The starting tris(4-iodophenyl)amine **9** was prepared by a well-known protocol.[1] Starting from commercially available 1,3,5-tris(trifluoro)methylbenzene and 4-(pentafluoro-λ^6-sulfanyl)aniline, the corresponding 2-iodo-1,3,5-tris(trifluoromethyl)benzene **12c** and 1-iodo-4-(pentafluoro-λ^6-sulfanyl)benzene **12d** were prepared according to the modified literature procedures.[2,3] Particular preparations and characterizations are given below. All commercial chemicals, reagents and solvents were purchased from Apollo Scientific, Aldrich (Merck), Acros, TCI, and Penta and were used as received. Column chromatography was carried out with silicagel 60 (particle size 0.040–0.063 mm, 230–400 mesh; Merck) and commercially available solvents. Thin-layer chromatography (TLC) was conducted on aluminum sheets coated with silica gel 60 F254, obtained from Merck, with visualization by a UV lamp (254 or 360nm). Melting points were determined in open capillaries on a Buchi B-540 apparatus. Thermal properties were determined by differential scanning calorimetry DSC with a Mettler-Toledo STARe System DSC 2/700 equipped with FRS 6 ceramic sensor and cooling system HUBER TC100-MT RC 23. Thermal behaviour of the target compounds was measured in aluminous crucibles sealed with a holed lid under N_2 inert atmosphere (60 ml×min-1). DSC curves were recorded with a scan rate of 5 °C/min within the range 25–500 °C. Temperature of decomposition were estimated as intersection of the baseline and tangent of the peak (onset point). ^{1}H, ^{13}C, and ^{19}F NMR spectra were recorded at 500, 125, and 470 MHz, respectively, with a Bruker Ascend TM 500 at 25 °C. Chemical shifts are reported in ppm relative to the signal of Me_4Si. The residual solvent signal in the ^{1}H and ^{13}C NMR spectra was used as an internal reference ($CDCl_3$ δ = 7.25 and 77.23 ppm). C_6F_6 was used as an internal standard for ^{19}F NMR experiments ($\delta = -164.9$ ppm). Apparent resonance multiplicities are described as s (singlet), d (doublet), dd (doublet of doublet), t (triplet), dt (doublet of triplet), q (quartet), kv (quintet), m (multiplet), and dm (doublet of multiplet). Apparent coupling constants of multiplets (*J*) are given in Hz. Phenyl and thienyl signals are marked as CH_{Ph} and CH_{Th}, respectively. Mass spectra were measured with a GC–MS configuration comprised of an Agilent Technologies 6890N gas chromatograph equipped with a 5973 Network MS detector (EI 70 eV, mass range 33–550 Da) or an Agilent 7890B Series GC Custom equipped with a 5977B EI MSD Bundle detector (EI 70 eV, mass range 50–550 Da). High-resolution MALDI MS spectra were measured with a MALDI mass spectrometer LTQ Orbitrap XL (Thermo Fisher Scientific, Bremen, Germany) equipped with nitrogen UV laser (337 nm, 60 Hz). The LTQ Orbitrap instrument was operated in positive-ion mode over a normal mass range (m/z 50–2000) with resolution 100 000 at m/z = 400. The survey crystal positioning system (survey CPS) was set for the random choice of shot position by automatic crystal recognition. 2,5-Dihydroxybenzoic acid (DHB) was used as a matrix. Mass spectra were averaged over the whole MS record for all measured samples. Voltammetric measurements were performed by using an integrated potentiostat system ER466 (eDAQ Europe, Warszawa, Poland) operated with EChem Electrochemistry software. Absorption and fluorescence spectra were measured on a Duetta^{TM} HORIBA spectrophotometer in toluene, THF, and acetonitrile at concentration 5×10^{-6} M.

Compounds were excited at their absorption maxima (band of lowest energy) to record the emission spectra. Φ^F values were calculated by a well-known procedure with perylene as internal standard (Φ^F = 0.94 in cyclohexane).[4] The Time Correlated Single Photon Counting (TCSPC) method was employed to detect fluorescence decays in the ps-ns timescale. The excitation was made by means of a pulsed diode laser at 400 nm and the temporal resolution was 80 ps. The detection was made by a microchannel plate photomultiplier. Magic angle conditions were used while the samples were dilute solutions of the compounds with an optical density of ~0.1 at the excitation wavelength.[5,6] The measurement of the two-photon absorption cross sections was realized by a two-photon excited fluorescence (TPEF) measurements using a Ti:Sapphire mode-locked laser as the excitation source.[7,8] The laser emits 80 fs pulses tuned from 730 to 870 nm. The excitation light was focused on the samples by a microscope objective lens and the emitted fluorescence was collected backward. It was separated from the excitation beam by a dichroic mirror and a set of filters. The TPEF intensity was measured as a function of the excitation power and the scattered light from the solvent was subtracted. A solution of Rhodamine 6G in MeOH, which has a well-characterized TPA spectrum, was used as a reference. The square-law dependence was always verified before calculating the TPA cross sections.

General Procedure for the first Suzuki-Miyaura cross-coupling reaction (i):

2-(Thiophen-2-yl)vinylboronic acid pinacol ester **7** (519 mg; 2.2 mmol) and the corresponding bromo or iodobenzene **10–14** (2 mmol) were dissolved in the mixture of THF:H_2O (8:2 mL). Argon was bubbled through the solution for 15 min, whereupon [$PdCl_2(PPh_3)_2$] (28 mg; 0.04 mmol) and K_2CO_3 (415 mg; 3 mmol) were added. The reaction mixture was stirred under argon atmosphere at 60 °C for 18 hours. The reaction mixture was cooled to room temperature, poured into aq. NH_4Cl (50 mL) and extracted with EtOAc (3×25 mL). The combined organic extracts were dried (Na_2SO_4) and the solvents were evaporated *in vacuo*. The crude product was purified by column chromatography (SiO_2; CH_2Cl_2/hexane 1:4).

General Procedure for the bromination (ii):

In a 100 mL round-bottom flask, particular thiophene derivative **15–19** (1.5 mmol) and *N*-bromosuccinimide (NBS; 280 mg; 1.575 mmol) were dissolved in the mixture of $CHCl_3$:glacial AcOH (30:15 mL). The reaction mixture was stirred under condenser at 80 °C for 18 hours. The reaction mixture was cooled to room temperature, poured into water (50 mL) and extracted with EtOAc (2×25 mL). The combined organic extracts were washed with sat. aq. Na_2CO_3 and brine. The organic phase was dried (Na_2SO_4) and the solvents were evaporated *in vacuo*. The crude product was purified by column chromatography (SiO_2; CH_2Cl_2/hexane 1:4).

General Procedure for the Heck cross-coupling reaction (iii):

Vinylboronic acid pinacol ester **6** (231 mg; 1.5 mmol) and corresponding 2-bromothiophene **20–24** (1 mmol) were dissolved in dry toluene (6 mL). DIPEA (0.35 mL; 2 mmol) and tri-*tert*-butylphosphine (10 mg; 50 μmol; 1 M solution in toluene) were added and argon was bubbled through the solution for 15 min, whereupon [$Pd_2(dba)_3$] (9 mg; 10 μmol) were finally added. The reaction mixture was stirred under argon atmosphere at 95 °C for 18 hours. The reaction mixture was cooled to room temperature, poured into aq. NH_4Cl (50 mL) and extracted with EtOAc (3×25 mL). The combined organic extracts were dried (Na_2SO_4) and the solvents were

evaporated *in vacuo*. The crude product was purified by column chromatography (SiO_2; CH_2Cl_2/hexane 4:3 to CH_2Cl_2).

General Procedure for the final threefold Suzuki-Miyaura cross-coupling reaction (iv):

Tris(4-iodophenyl)amine **9** (94 mg; 0.15 mmol) and particular boronic acid pinacol ester **25–29** (0.54 mmol) were dissolved in the mixture of THF:H_2O (8:2 mL). Argon was bubbled through the solution for 15 min, whereupon [$PdCl_2(PPh_3)_2$] (6 mg; 9 µmol) and K_2CO_3 (104 mg; 0.75 mmol) were added. The reaction mixture was stirred under argon atmosphere at 60 °C for 18 hours. The reaction mixture was cooled to room temperature, poured into aq. NH_4Cl (50 mL) and extracted with EtOAc (3×25 mL). The combined organic extracts were dried (Na_2SO_4) and the solvents were evaporated *in vacuo*. The crude product was purified by column chromatography (SiO_2; CH_2Cl_2/hexane 1:1). Pre-purified product was finally precipitated from a CH_2Cl_2/hexane mixture.

(E)-2-(Thiophen-2-yl)vinylboronic acid pinacol ester 7

The title compound was synthesized from 2-bromothiophene **5** (4.89 g, 30 mmol) and vinylboronic acid pinacol ester **6** (6.93 g, 45 mmol) by following the general procedure *iii*. Yield: 6.58 g (93 %); white-off solid (solidified slowly); R_f = 0.5 (SiO_2, CH_2Cl_2/hex 4:3); m.p. = 39–44 °C. EI/MS (70 eV): *m/z* (%): 236 (M^+, 75), 221 (18), 163 (25), 151 (42), 136 (100), 120 (19), 111 (29). ^1H NMR (500 MHz, $CDCl_3$, 25 °C): δ = 7.46 (d, J = 18.1 Hz, 1H, CH), 7.23 (d, J = 5.3 Hz, 1H, CH_{Th}), 7.07 (d, J = 3.5 Hz, 1H, CH_{Th}), 6.97 (dd, J_1 = 5.3 Hz, J_2 = 3.5 Hz, 1H, CH_{Th}), 5.90 (d, J = 18.1 Hz, 1H, CH), 1.29 ppm (s, 12H, CH_3). The analytical data are consistent with the literature.[9]

2-Iodo-1,3,5-tris(trifluoromethyl)benzene ***12c***

iPr_2NH (445 mg; 4.4 mmol) was dissolved in dry THF (50 mL) and the solution was cooled to −78 °C. Argon was bubbled through the solution for 15 min, whereupon *n*BuLi (4.4 mmol; 1.6 M in hexane) was added dropwise. The reaction mixture was stirred under argon atmosphere at −78 °C for 15 min. The reaction mixture was heated to 0 °C and a solution of 1,3,5-tris(trifluoromethyl)benzene (1.13 g; 4 mmol) in dry THF (5 mL) was added dropwise. The reaction mixture was stirred at 0 °C for 75 min. The iodine (1.52 g; 6 mmol) was added and the reaction mixture was stirred at 0 °C for 5 min and further 15 min at room temperature. The reaction mixture was poured into sat. aq. Na_2SO_3 (50 mL) and extracted with Et_2O (1×25 mL). The organic phase was washed with brine (50 mL) and 1 M HCl (50 mL) and aqueous phase after acidic workup was washed with Et_2O (2×30 mL). The combined organic extracts were dried (Na_2SO_4) and the solvents were evaporated *in vacuo*. The crude product was purified by column chromatography (SiO_2; hexane). The iodo-derivative **12c** was isolated as a pale-yellow solid. Yield: 1.08 g (66 %); R_f = 0.9 (SiO_2, hexane). EI/MS (70 eV): *m/z* (%): 408 (M^+, 100), 389 (21), 281 (50), 262 (18), 231 (14), 212 (12), 162 (10). ^1H NMR (500 MHz, $CDCl_3$, 25 °C): δ = 8.03 ppm (s, 2H, CH_{Ph}). The analytical data are consistent with the literature.[2]

1-Iodo-4-(pentafluorosulfanyl)benzene ***12d***

Into a 250-mL round-bottom flask, 4-(pentafluorosulfanyl)aniline (3.5 g; 16 mmol) was dissolved in conc. HCl (15 mL) and ice (30 g) was added to the solution. The flask was placed in an ice bath and aqueous solution of $NaNO_2$ (16.8 mmol in 30 mL) was slowly added to the

vigorously stirred reaction mixture. The reaction mixture was stirred at 0 °C for further 5 min, whereupon aqueous solution of KI (16 mmol in 60 mL) was added. The reaction mixture was stirred at 0 °C for 30 min and further 1 h at room temperature. The resulting suspension was extracted with CH_2Cl_2 (4×20 mL). The organic extracts were combined and extracted with sat. aq. $NaHCO_3$. The separated organic phase was dried (Na_2SO_4) and the solvent was evaporated *in vacuo*. The crude product was purified by column chromatography (SiO_2, hexane). The iodo-derivative **12d** was isolated as a white solid. Yield: 4.44 g (84 %); R_f = 0.9 (SiO_2, hexane). EI/MS (70 eV): *m/z* (%): 330 (M^+, 100), 222 (19), 203 (21), 95 (39), 76 (29). ^{1}H NMR (500 MHz, $CDCl_3$, 25 °C): δ = 7.81 (d, J = 8.1 Hz, 2H, CH_{Ph}), 7.47 ppm (d, J = 8.8 Hz, 2H, CH_{Ph}). The analytical data are consistent with the literature.[3]

2-[(E)-2-Phenylethen-1-yl]thiophene ***15a***

The title compound was synthesized from boronic acid pinacol ester **7** (519 mg) and iodobenzene **10a** (408 mg) by following the general procedure *i*. Yield: 300 mg (81 %); pale yellow solid; R_f = 0.6 (SiO_2, CH_2Cl_2/hex 1:4); m.p. = 104–107 °C. EI/MS (70 eV): *m/z* (%): 186 (M^+, 100), 171 (15), 152 (20), 141 (19), 115 (10). ^{1}H NMR (500 MHz, $CDCl_3$, 25 °C): δ = 7.46 (d, J = 7.6 Hz, 2H, CH_{Ph}), 7.34 (t, J = 7.5 Hz, 2H, CH_{Ph}), 7.25–7.18 (m, 3H, CH+CH_{Ph}+CH_{Th}), 7.06 (d, J = 3.4 Hz, 1H, CH_{Th}), 6.99 (dd, J_1 = 5.0 Hz, J_2 = 3.4 Hz, 1H, CH_{Th}), 6.92 ppm (d, J = 16.0 Hz, 1H, CH). ^{13}C NMR (125 MHz, $CDCl_3$, 25 °C): δ = 143.09, 137.16, 128.91, 128.53, 127.80, 126.50, 126.32, 124.55, 121.98 ppm. The analytical data are consistent with the literature.[10]

2-[(E)-2-(4-Methoxyphenyl)ethen-1-yl]thiophene ***15b***

The title compound was synthesized from boronic acid pinacol ester **7** (519 mg) and 4-bromoanisole **10b** (374 mg) by following the general procedure *i*. Yield: 340 mg (79 %); white solid; R_f = 0.5 (SiO_2, CH_2Cl_2/hex 1:4); m.p. = 134–136 °C. EI/MS (70 eV): *m/z* (%): 216 (M^+, 100), 201 (32), 171 (18), 129 (15). ^{1}H NMR (500 MHz, $CDCl_3$, 25 °C): δ = 7.39 (d, J = 8.6 Hz, 2H, CH_{Ph}), 7.17 (d, J = 5.0 Hz, 1H, CH_{Th}), 7.09 (d, J = 16.2 Hz, 1H, CH), 7.02–6.97 (m, 2H, CH_{Th}), 6.89–6.86 (m, 3H, CH+CH_{Ph}), 3.82 ppm (s, 3H, OCH_3). ^{13}C NMR (125 MHz, $CDCl_3$, 25 °C): δ = 159.46, 143.46, 129.94, 128.15, 127.75, 127.72, 125.62, 123.94, 119.99, 114.35, 55.54 ppm. The analytical data are consistent with the literature.[11]

1-{4-[(E)-2-(Thiophen-2-yl)ethen-1-yl]phenyl}ethan-1-one ***15c***

The title compound was synthesized from boronic acid pinacol ester **7** (519 mg) and 4′-bromoacetophenone **10c** (398 mg) by following the general procedure *i*. Yield: 409 mg (90 %); pale yellow solid; R_f = 0.5 (SiO_2, CH_2Cl_2/hex 2:1); m.p. = 123–128 °C. EI/MS (70 eV): *m/z* (%): 228 (M^+, 80), 213 (100), 184 (48), 152 (20), 141 (10), 92 (10). ^{1}H NMR (500 MHz, $CDCl_3$, 25 °C): δ = 7.93 (d, J = 8.3 Hz, 2H, CH_{Ph}), 7.52 (d, J = 8.3 Hz, 2H, CH_{Ph}), 7.34 (d, J = 16.3 Hz, 1H, CH), 7.24 (d, J = 4.8 Hz, 1H, CH_{Th}), 7.12 (d, J = 3.2 Hz, 1H, CH_{Th}), 7.02 (dd, J_1 = 5.2 Hz, J_2 = 3.4 Hz 1H, CH_{Th}), 6.93 (d, J = 16.3 Hz, 1H, CH), 2.60 ppm (s, 3H, CH_3). ^{13}C NMR (125 MHz, $CDCl_3$, 25 °C): δ = 197.66, 142.46, 141.89, 136.05, 129.11, 128.01, 127.50, 127.12, 126.45, 125.62, 124.63, 26.81 ppm. The analytical data are consistent with the literature.[12]

2-[(E)-2-(4-Fluorophenyl)ethen-1-yl]thiophene ***16a***

The title compound was synthesized from boronic acid pinacol ester **7** (519 mg) and 1-fluoro-4-iodobenzene **11a** (444 mg) by following the general procedure *i*. Yield: 360 mg (88 %); pale yellow solid; R_f = 0.7 (SiO_2, CH_2Cl_2/hex 1:4); m.p. = 99–102 °C. EI/MS (70 eV): *m/z* (%): 204 (M^+, 100), 189 (9), 170 (19), 159 (19), 133 (11). ^1H NMR (500 MHz, $CDCl_3$, 25 °C): δ = 7.43–7.40 (m, 2H, CH_{Ph}), 7.18 (d, J = 5.1 Hz, 1H, CH_{Th}), 7.13 (d, J = 16.2 Hz, 1H, CH), 7.05–6.99 (m, 4H, CH_{Ph}+CH_{Th}), 6.88 ppm (d, J = 16.0 Hz, 1H, CH). ^{13}C NMR (125 MHz, $CDCl_3$, 25 °C): δ = 162.49 (d, $^1J_{CF}$ = 247.3 Hz), 142.87, 133.34 (d, $^4J_{CF}$ = 3.3 Hz), 127.97 (d, $^3J_{CF}$ = 7.8 Hz), 127.83, 127.30, 126.32, 124.55, 121.78 (d, $^5J_{CF}$ = 2.1 Hz), 115.88 ppm (d, $^2J_{CF}$ = 21.6 Hz). ^{19}F NMR (470 MHz, 25 °C, $CDCl_3$): δ = −112.50 to −112.56 ppm (m). The analytical data are consistent with the literature.[13]

2-[(E)-2-(3,5-Difluorophenyl)ethen-1-yl]thiophene ***16b***

The title compound was synthesized from boronic acid pinacol ester **7** (519 mg) and 1,3-difluoro-5-iodobenzene **11b** (468 mg) by following the general procedure *i*. Yield: 410 mg (92 %); pale yellow solid; R_f = 0.8 (SiO_2, CH_2Cl_2/hex 1:4); m.p. = 45–49 °C. EI/MS (70 eV): *m/z* (%): 222 (M^+, 100), 202 (12), 189 (18), 177 (25), 151 (10). ^1H NMR (500 MHz, $CDCl_3$, 25 °C): δ = 7.25–7.20 (m, 2H, CH+CH_{Th}), 7.10 (d, J = 3.3 Hz, 1H, CH_{Th}), 7.01 (dd, J_1 = 4.8 Hz, J_2 = 3.6 Hz, 1H, CH_{Th}), 6.97–6.93 (m, 2H, CH_{Ph}), 6.80 (d, J = 16.2 Hz, 1H, CH), 6.70–6.65 ppm (m, 1H, CH_{Ph}). ^{13}C NMR (125 MHz, $CDCl_3$, 25 °C): δ = 163.54 (d, $^1J_{CF}$ = 247.4 Hz), 163.44 (d, $^1J_{CF}$ = 248.0 Hz), 141.92, 140.60 (t, $^3J_{CF}$ = 9.7 Hz), 127.99, 127.57, 126.14 (t, $^4J_{CF}$ = 2.9 Hz), 125.64, 124.47, 109.11–108.90 (m), 102.88 ppm (t, $^2J_{CF}$ = 25.5 Hz). ^{19}F NMR (470 MHz, 25 °C, $CDCl_3$): δ = −108.53 to −108.56 ppm (m).

2-[(E)-2-(2,4,6-Trifluorophenyl)ethen-1-yl]thiophene ***16c***

The title compound was synthesized from boronic acid pinacol ester **7** (519 mg) and 1,3,5-trifluoro-2-iodobenzene **11c** (516 mg) by following the general procedure *i*. Yield: 370 mg (77 %); pale yellow liquid; R_f = 0.8 (SiO_2, CH_2Cl_2/hex 1:4); m.p. = 20–25 °C. EI/MS (70 eV): *m/z* (%): 240 (M^+, 100), 220 (38), 195 (11), 169 (10), 156 (10). ^1H NMR (500 MHz, $CDCl_3$, 25 °C): δ = 7.47 (d, J = 16.3 Hz, 1H, CH), 7.24 (d, J = 5.3 Hz, 1H, CH_{Th}), 7.10 (d, J = 3.4 Hz, 1H, CH_{Th}), 7.01 (dd, J_1 = 5.3 Hz, J_2 = 3.8 Hz, 1H, CH_{Th}), 6.84 (d, J = 16.8 Hz, 1H, CH), 6.70–6.67 ppm (m, 2H, CH_{Ph}). ^{13}C NMR (125 MHz, $CDCl_3$, 25 °C): δ = 162.29–159.98 (2×dm, 2×C–F), 143.07, 127.90, 127.86–127.77 (m), 127.22, 125.48, 113.85, 111.54–111.25 (m), 101.00–100.49 ppm (m). ^{19}F NMR (470 MHz, 25 °C, $CDCl_3$): δ = −107.79 to −107.85 (m, 1F), −108.07 to −108.10 ppm (m, 2F).

2-[(E)-2-(2,3,4,5,6-Pentafluorophenyl)ethen-1-yl]thiophene ***16d***

The title compound was synthesized from boronic acid pinacol ester **7** (519 mg) and 1,2,3,4,5-pentafluoro-6-iodobenzene **11d** (588 mg) by following the general procedure *i*. Yield: 300 mg (54 %); pale yellow solid; R_f = 0.8 (SiO_2, CH_2Cl_2/hex 1:4); m.p. = 96–99 °C. EI/MS (70 eV): *m/z* (%): 276 (M^+, 100), 256 (56), 225 (13), 207 (17). ^1H NMR (500 MHz, $CDCl_3$, 25 °C): δ = 7.53 (d, J = 16.5 Hz, 1H, CH), 7.30 (d, J = 5.0 Hz, 1H, CH_{Th}), 7.15 (d, J = 3.5 Hz, 1H, CH_{Th}), 7.03 (dd, J_1 = 5.0 Hz, J_2 = 3.5 Hz, 1H, CH_{Th}), 6.78 ppm (d, J = 16.5 Hz, 1H, CH). ^{13}C NMR (125 MHz, $CDCl_3$, 25 °C): δ = 144.87 (dm), 142.12, 139.90 (dm), 137.90 (dm), 130.32–130.17 (m), 128.42, 128.09, 126.58, 112.04–112.01 ppm (m). ^{19}F NMR (470 MHz,

25 °C, $CDCl_3$): $\delta = -141.20$ (dd, $J_1 = 21.3$ Hz, $J_2 = 7.2$ Hz, 2F), −155.06 (t, $J = 20.5$ Hz, 1F), −161.34 ppm (dt, $J_1 = 21.4$ Hz, $J_2 = 7.0$ Hz, 2F).

2-{(E)-2-[4-(Trifluoromethyl)phenyl]ethen-1-yl}thiophene ***17a***

The title compound was synthesized from boronic acid pinacol ester **7** (519 mg) and 1-iodo-4-(trifluoromethyl)benzene **12a** (544 mg) by following the general procedure *i*. Yield: 440 mg (87 %); white solid; $R_f = 0.7$ (SiO_2, CH_2Cl_2/hex 1:4); m.p. = 144–148 °C. EI/MS (70 eV): *m/z* (%): 254 (M^+, 100), 233 (13), 209 (13), 184 (47), 152 (15). ^{1}H NMR (500 MHz, $CDCl_3$, 25 °C): $\delta = 7.59$–7.53 (m, 4H, CH_{Ph}), 7.30 (d, $J = 16.1$ Hz, 1H, CH), 7.24 (d, $J = 5.2$ Hz, 1H, CH_{Th}), 7.11 (d, $J = 3.1$ Hz, 1H, CH_{Th}), 7.02 (dd, $J_1 = 4.9$ Hz, $J_1 = 3.5$ Hz, 1H, CH_{Th}), 6.92 ppm (d, $J = 16.1$ Hz, 1H, CH). ^{13}C NMR (125 MHz, $CDCl_3$, 25 °C): $\delta = 142.32$, 140.66, 129.36 (q, $^2J_{CF} = 32.6$ Hz), 127.97, 127.40, 126.78, 126.52, 125.86 (q, $^3J_{CF} = 3.9$ Hz), 125.50, 124.41 (q, $^1J_{CF} = 272.5$ Hz), 124.37 ppm.^{19}F NMR (470 MHz, 25 °C, $CDCl_3$): $\delta = -60.83$ ppm. The analytical data are consistent with the literature.[14]

2-{(E)-2-[3,5-Bis(trifluoromethyl)phenyl]ethen-1-yl}thiophene ***17b***

The title compound was synthesized from boronic acid pinacol ester **7** (519 mg) and 1-iodo-3,5-bis(trifluoromethyl)benzene **12b** (680 mg) by following the general procedure *i*. Yield: 600 mg (93 %); pale yellow solid; $R_f = 0.8$ (SiO_2, CH_2Cl_2/hex 1:4); m.p. = 79–82 °C. EI/MS (70 eV): *m/z* (%): 322 (M^+, 100), 301 (16), 252 (16), 184 (12). ^{1}H NMR (500 MHz, $CDCl_3$, 25 °C): $\delta = 7.85$ (s, 2H, CH_{Ph}), 7.71 (s, 1H, CH_{Ph}), 7.37 (d, $J = 15.8$ Hz, 1H, CH), 7.28 (d, $J = 5.4$ Hz, 1H, CH_{Th}), 7.16 (d, $J = 3.5$ Hz, 1H, CH_{Th}), 7.04 (dd, $J_1 = 4.9$ Hz, $J_2 = 3.5$ Hz, 1H, CH_{Th}), 6.94 ppm (d, $J = 16.3$ Hz, 1H, CH). ^{13}C NMR (125 MHz, $CDCl_3$, 25 °C): $\delta = 141.59$, 139.30, 132.24 (q, $^2J_{CF} = 33.2$ Hz), 128.16, 128.11, 126.15, 126.06 (q, $^3J_{CF} = 2.9$ Hz), 125.63, 125.04, 123.52 (q, $^1J_{CF} = 272.4$ Hz), 120.87–120.78 ppm (m). ^{19}F NMR (470 MHz, 25 °C, $CDCl_3$): $\delta = -61.37$ ppm.

2-{(E)-2-[2,4,6-Tris(trifluoromethyl)phenyl]ethen-1-yl}thiophene ***17c***

The title compound was synthesized from boronic acid pinacol ester **7** (519 mg) and 2-iodo-1,3,5-tris(trifluoromethyl)benzene **12c** (816 mg) by following the general procedure *i*, using 1,4-dioxane/H_20 (8:2 mL) was used as a solvent system and raising the reaction temperature to 100 °C. Yield: 577 mg (74 %); white solid; $R_f = 0.8$ (SiO_2, CH_2Cl_2/hex 1:4); m.p. = 108–110 °C. EI/MS (70 eV): *m/z* (%): 390 (M^+, 100), 371 (17), 301 (56), 252 (48). ^{1}H NMR (500 MHz, $CDCl_3$, 25 °C): $\delta = 8.14$ (s, 2H, CH_{Ph}), 7.31 (d, $J = 5.1$ Hz, 1H, CH_{Th}), 7.11 (d, $J = 3.4$ Hz, 1H, CH_{Th}), 7.04-7.00 (m, 2H, CH+CH_{Th}), 6.81 ppm (d, $J = 16.5$ Hz, 1H, CH). ^{13}C NMR (125 MHz, $CDCl_3$, 25 °C): $\delta = 141.10$, 140.94, 132.33 (q, $^2J_{CF} = 30.6$ Hz), 131.28, 130.28 (q, $^2J_{CF} = 35.4$ Hz), 128.14, 127.94, 126.78, 126.47, 122.92 (q, $^1J_{CF} = 273.4$ Hz), 118.29 ppm. ^{19}F NMR (470 MHz, 25 °C, $CDCl_3$): $\delta = -57.15$ (s, 6F), −61.37 ppm (s, 3F).

2-{(E)-2-[4-(Pentafluorosulfanyl)phenyl]ethen-1-yl}thiophene ***17d***

The title compound was synthesized from boronic acid pinacol ester **7** (519 mg) and 1-iodo-4-(pentafluorosulfanyl)benzene **12d** (660 mg) by following the general procedure *i*. Yield: 550 mg (88 %); white solid; $R_f = 0.8$ (SiO_2, CH_2Cl_2/hex 1:4); m.p. = 151–154 °C. EI/MS (70 eV): *m/z* (%): 312 (M^+, 100), 184 (44), 152 (10). ^{1}H NMR (500 MHz, $CDCl_3$, 25 °C): $\delta = 7.70$ (d, $J = 8.9$ Hz, 2H, CH_{Ph}), 7.50 (d, $J = 8.3$ Hz, 2H, CH_{Ph}), 7.30 (d, $J = 16.2$ Hz, 1H, CH), 7.25 (d, $J = 4.9$ Hz, 1H, CH_{Th}), 7.12 (d, $J = 3.3$ Hz, 1H, CH_{Th}), 7.02 (dd, $J_1 = 4.9$ Hz,

J_1 = 3.6 Hz, 1H, CH_{Th}), 6.89 ppm (d, J = 16.2 Hz, 1H, CH). ^{13}C NMR (125 MHz, $CDCl_3$, 25 °C): δ = 142.09, 140.48, 128.02, 127.72, 126.62–126.54 (m), 126.26, 125.98, 125.78, 125.10 ppm. ^{19}F NMR (470 MHz, 25 °C, $CDCl_3$): δ = 86.61 (kv, J = 150.4 Hz, 1F, SF_5), 64.70 ppm (d, J = 150.4 Hz, 4F, SF_5).

2-{(E)-2-[3,5-Bis(pentafluorosulfanyl)phenyl]ethen-1-yl}thiophene ***17e***

The title compound was synthesized from boronic acid pinacol ester **7** (519 mg) and 1-bromo-3,5-bis(pentafluorosulfanyl)benzene **12e** (818 mg) by following the general procedure *i*. Yield: 820 mg (94 %); pale yellow solid; R_f = 0.8 (SiO_2, CH_2Cl_2/hex 1:4); m.p. = 174–178 °C. EI/MS (70 eV): *m/z* (%): 438 (M^+, 100), 311 (12), 202 (14), 184 (19), 139 (10). ^{1}H NMR (500 MHz, $CDCl_3$, 25 °C): δ = 7.96–7.94 (m, 3H, CH_{Ph}), 7.35 (d, J = 16.1 Hz, 1H, CH), 7.30 (d, J = 5.1 Hz, 1H, CH_{Th}), 7.19 (d, J = 3.6 Hz, 1H, CH_{Th}), 7.05 (dd, J_1 = 4.9 Hz, J_1 = 3.6 Hz, 1H, CH_{Th}), 6.91 ppm (d, J = 16.1 Hz, 1H, CH). ^{13}C NMR (125 MHz, $CDCl_3$, 25 °C): δ = 154.40–154.10 (m, CSF_5), 141.16, 139.56, 128.70, 128.21, 126.64, 126.57, 126.36–126.30 (m), 124.31, 122.46–122.31 ppm (m). ^{19}F NMR (470 MHz, 25 °C, $CDCl_3$): δ = 83.61 (kv, J = 152.4 Hz, 1F, SF_5), 64.47 ppm (d, J = 150.8 Hz, 4F, SF_5).

2-{(E)-2-[4-(Trifluoromethoxy)phenyl]ethen-1-yl}thiophene ***18a***

The title compound was synthesized from boronic acid pinacol ester **7** (519 mg) and 1-iodo-4-(trifluoromethoxy)benzene **13a** (576 mg) by following the general procedure *i*. Yield: 420 mg (78 %); pale yellow solid; R_f = 0.8 (SiO_2, CH_2Cl_2/hex 1:4); m.p. = 97–100 °C. EI/MS (70 eV): *m/z* (%): 270 (M^+, 100), 185 (21), 171 (14). ^{1}H NMR (500 MHz, $CDCl_3$, 25 °C): δ = 7.46 (d, J = 8.4 Hz, 2H, CH_{Ph}), 7.21–7.17 (m, 4H, CH+CH_{Ph}+CH_{Th}), 7.08 (d, J = 3.4 Hz, 1H, CH_{Th}), 7.01 (dd, J_1 = 5.0 Hz, J_1 = 3.7 Hz, 1H, CH_{Th}), 6.89 ppm (d, J = 15.9 Hz, 1H, CH). ^{13}C NMR (125 MHz, $CDCl_3$, 25 °C): δ = 148.62, 142.59, 135.95, 127.89, 127.64, 126.83, 126.79, 124.97, 122.94, 121.43, 120.68 ppm (q, $^1J_{CF}$ = 257.2 Hz).^{19}F NMR (470 MHz, 25 °C, $CDCl_3$): δ = −56.15 ppm.

2-[(E)-2-{4-[(Trifluoromethyl)sulfanyl]phenyl}ethen-1-yl]thiophene ***18b***

The title compound was synthesized from boronic acid pinacol ester **7** (519 mg) and 1-iodo-4-[(trifluoromethyl)sulfanyl]benzene **13b** (514 mg) by following the general procedure *i*. Yield: 385 mg (67 %); pale yellow solid; R_f = 0.7 (SiO_2, CH_2Cl_2/hex 1:4); m.p. = 99–101 °C. EI/MS (70 eV): *m/z* (%): 286 (M^+, 100), 217 (40), 184 (59). ^{1}H NMR (500 MHz, $CDCl_3$, 25 °C): δ = 7.60 (d, J = 8.2 Hz, 2H, CH_{Ph}), 7.48 (d, J = 8.2 Hz, 2H, CH_{Ph}), 7.29 (d, J = 16.1 Hz, 1H, CH), 7.24 (d, J = 5.0 Hz, 1H, CH_{Th}), 7.11 (d, J = 3.2 Hz, 1H, CH_{Th}), 7.02 (dd, J_1 = 5.0 Hz, J_1 = 3.8 Hz, 1H, CH_{Th}), 6.90 ppm (d, J = 15.8 Hz, 1H, CH). ^{13}C NMR (125 MHz, $CDCl_3$, 25 °C): δ = 142.38, 139.86, 136.90, 127.98, 127.36, 127.28, 126.79, 125.49, 124.29, 122.93 ppm. The CF_3 quartet is missing. ^{19}F NMR (470 MHz, 25 °C, $CDCl_3$): δ = −41.17 ppm.

2,2,2-Trifluoro-1-{4-[(E)-2-(thiophen-2-yl)ethen-1-yl]phenyl}ethan-1-one ***19a***

The title compound was synthesized from boronic acid pinacol ester **7** (519 mg) and 1-(4-bromophenyl)-2,2,2-trifluoroethan-1-one **14a** (506 mg) by following the general procedure *i*. Yield: 490 mg (87 %); yellow solid; R_f = 0.4 (SiO_2, CH_2Cl_2/hex 1:4); m.p. = 122–124 °C. EI/MS (70 eV): *m/z* (%): 282 (M^+, 100), 213 (85), 184 (42), 152 (14). ^{1}H NMR (500 MHz, $CDCl_3$, 25 °C): δ = 8.04 (d, J = 8.3 Hz, 2H, CH_{Ph}), 7.59 (d, J = 8.3 Hz, 2H, CH_{Ph}), 7.42 (d, J = 16.1 Hz, 1H, CH), 7.29 (d, J = 5.0 Hz, 1H, CH_{Th}), 7.17 (d, J = 3.3 Hz,

1H, CH_{Th}), 7.04 (dd, J_1 = 4.8 Hz, J_1 = 3.5 Hz, 1H, CH_{Th}), 6.94 ppm (d, J = 16.1 Hz, 1H, CH). ^{13}C NMR (125 MHz, $CDCl_3$, 25 °C): δ = 179.86 (q, $^2J_{CF}$ = 35.0 Hz), 144.41, 142.04, 130.96, 130.94 128.58, 128.34, 128.16, 126.75, 126.40, 126.38, 116.99 ppm (q, $^1J_{CF}$ = 292.5 Hz). ^{19}F NMR (470 MHz, 25 °C, $CDCl_3$): δ = −69.55 ppm.

2-{(E)-2-[4-(Trifluoromethanesulfonyl)phenyl]ethen-1-yl}thiophene ***19b***

The title compound was synthesized from boronic acid pinacol ester **7** (519 mg) and 1-bromo-4-(trifluoromethanesulfonyl)benzene **14b** (578 mg) by following the general procedure *i*. Yield: 550 mg (86 %); yellow solid; R_f = 0.4 (SiO_2, CH_2Cl_2/hex 1:4); m.p. = 90–93 °C. EI/MS (70 eV): *m/z* (%): 318 (M^+, 100), 201 (24), 185 (85), 152 (29), 141 (16). ^{1}H NMR (500 MHz, $CDCl_3$, 25 °C): δ = 7.97 (d, J = 8.1 Hz, 2H, CH_{Ph}), 7.68 (d, J = 8.3 Hz, 2H, CH_{Ph}), 7.45 (d, J = 16.2 Hz, 1H, CH), 7.32 (d, J = 5.0 Hz, 1H, CH_{Th}), 7.19 (d, J = 3.0 Hz, 1H, CH_{Th}), 7.05 (dd, J_1 = 5.0 Hz, J_1 = 3.0 Hz, 1H, CH_{Th}), 6.94 ppm (d, J = 15.9 Hz, 1H, CH). ^{13}C NMR (125 MHz, $CDCl_3$, 25 °C): δ = 145.68, 141.59, 131.48, 128.89, 128.84, 128.23, 127.52, 127.26, 126.85, 125.39, 120.05 ppm (q, $^1J_{CF}$ = 325.9 Hz). ^{19}F NMR (470 MHz, 25 °C, $CDCl_3$): δ = −76.82 ppm.

4-[(E)-2-(thiophen-2-yl)ethen-1-yl]phenyl trifluoromethanesulfonate ***19c***

The title compound was synthesized from boronic acid pinacol ester **7** (519 mg) and 4-bromophenyl trifluoromethanesulfonate **14c** (610 mg) by following the general procedure *i*. Yield: 620 mg (93 %); yellow solid; R_f = 0.5 (SiO_2, CH_2Cl_2/hex 1:4); m.p. = 56–60 °C. EI/MS (70 eV): *m/z* (%): 334 (M^+, 48), 201 (100), 171 (21), 129 (23). ^{1}H NMR (500 MHz, $CDCl_3$, 25 °C): δ = 7.50 (d, J = 8.6 Hz, 2H, CH_{Ph}), 7.25–7.21 (m, 4H, CH_{Ph}+CH_{Th}+CH), 7.10 (d, J = 3.4 Hz, 1H, CH_{Th}), 7.01 (dd, J_1 = 5.0 Hz, J_1 = 3.9 Hz, 1H, CH_{Th}), 6.88 ppm (d, J = 16.2 Hz, 1H, CH). ^{13}C NMR (125 MHz, $CDCl_3$, 25 °C): δ = 148.71, 142.25, 137.66, 127.95, 127.92, 127.25, 126.17, 125.37, 123.98, 121.85, 118.94 ppm (q, $^1J_{CF}$ = 320.9 Hz). ^{19}F NMR (470 MHz, 25 °C, $CDCl_3$): δ = −71.11 ppm.

2-Bromo-5-[(E)-2-phenylethen-1-yl]thiophene ***20a***

The title compound was synthesized from thiophene derivative **15a** (279 mg) and NBS (280 mg) by following the general procedure *ii*. Yield: 306 mg (77 %); pale yellow solid; R_f = 0.7 (SiO_2, CH_2Cl_2/hex 1:4); m.p. = 82–85 °C. EI/MS (70 eV): *m/z* (%): 266 (M^+, 63, ^{81}Br), 264 (M^+, 63, ^{79}Br), 184 (59), 152 (100), 141 (21), 115 (15), 92 (25). ^{1}H NMR (500 MHz, $CDCl_3$, 25 °C): δ = 7.44 (d, J = 7.3 Hz, 2H, CH_{Ph}), 7.34 (t, J = 7.3 Hz, 2H, CH_{Ph}), 7.26 (t, J = 7.5 Hz, 1H, CH_{Ph}), 7.10 (d, J = 16.0 Hz, 1H, CH), 6.94 (d, J = 3.8 Hz, 1H, CH_{Th}), 6.82–6.79 ppm (m, 2H, CH+CH_{Th}). ^{13}C NMR (125 MHz, $CDCl_3$, 25 °C): δ = 144.72, 136.76, 130.68, 128.97, 128.09, 126.55, 126.48, 121.38, 111.32 ppm. The analytical data are consistent with the literature.[15]

2-Bromo-5-[(E)-2-(4-methoxyphenyl)ethen-1-yl]thiophene ***20b***

The title compound was synthesized from thiophene derivative **15b** (324 mg) and NBS (280 mg) by following the general procedure *ii*, nevertheless, the reaction temperature was maintained at 25 °C. Yield: 376 mg (85 %); pale yellow solid; R_f = 0.3 (SiO_2, CH_2Cl_2/hex 1:4); m.p. = 123–126 °C. EI/MS (70 eV): *m/z* (%): 296 (M^+, 100), 279 (24), 171 (53). ^{1}H NMR (500 MHz, $CDCl_3$, 25 °C): δ = 7.37 (d, J = 8.6 Hz, 2H, CH_{Ph}), 6.96 (d, J = 16.1 Hz, 1H, CH), 6.92 (d, J = 3.8 Hz, 1H, CH_{Th}), 6.87 (d, J = 8.6 Hz, 2H, CH_{Ph}), 6.77–6.74 (m, 2H, CH+CH_{Th}),

3.82 ppm (s, 3H, OCH_3). ^{13}C NMR (125 MHz, $CDCl_3$, 25 °C): δ = 159.68, 145.11, 130.60, 129.53, 128.62, 127.81, 125.71, 119.37, 114.40, 110.55, 55.53 ppm.

1-{4-[(E)-2-(5-Bromothiophen-2-yl)ethen-1-yl]phenyl}ethan-1-one ***20c***

The title compound was synthesized from thiophene derivative **15c** (342 mg) and NBS (280 mg) by following the general procedure *ii*. Yield: 345 mg (75 %); yellow solid; R_f = 0.7 (SiO_2, CH_2Cl_2/hex 2:1); m.p. = 132–137 °C. EI/MS (70 eV): *m/z* (%): 308 (M^+, 100), 293 (89), 184 (66), 152 (21), 139 (28). ^1H NMR (500 MHz, $CDCl_3$, 25 °C): δ = 7.92 (d, J = 8.3 Hz, 2H, CH_{Ph}), 7.50 (d, J = 8.3 Hz, 2H, CH_{Ph}), 7.21 (d, J = 16.1 Hz, 1H, CH), 6.97 (d, J = 3.7 Hz, 1H, CH_{Th}), 6.85 (d, J = 3.7 Hz, 1H, CH_{Th}), 6.81 (d, J = 16.1 Hz, 1H, CH), 2.59 ppm (s, 3H, CH_3). ^{13}C NMR (125 MHz, $CDCl_3$, 25 °C): δ = 197.61, 144.06, 141.44, 136.26, 130.89, 129.13, 127.66, 127.53, 126.51, 123.94, 112.59, 26.81 ppm.

2-Bromo-5-[(E)-2-(4-fluorophenyl)ethen-1-yl]thiophene ***21a***

The title compound was synthesized from thiophene derivative **16a** (306 mg) and NBS (280 mg) by following the general procedure *ii*. Yield: 361 mg (85 %); yellow solid; R_f = 0.8 (SiO_2, CH_2Cl_2/hex 1:4); m.p. = 67–70 °C. EI/MS (70 eV): *m/z* (%): 282 (M^+, 98), 202 (87), 170 (100), 159 (49), 146 (15), 133 (27), 101 (41). ^1H NMR (500 MHz, $CDCl_3$, 25 °C): δ = 7.41–7.38 (m, 2H, CH_{Ph}), 7.04–6.99 (m, 3H, CH+CH_{Ph}), 6.94 (d, J = 3.7 Hz, 1H, CH_{Th}), 6.78–6.74 ppm (m, 2H, CH+CH_{Th}). ^{13}C NMR (125 MHz, $CDCl_3$, 25 °C): δ = 162.63 (d, $^1J_{CF}$ = 247.8 Hz), 144.49, 132.96 (d, $^4J_{CF}$ = 3.1 Hz), 130.69, 128.06 (d, $^3J_{CF}$ = 7.8 Hz), 127.74, 126.48, 121.17 (d, $^5J_{CF}$ = 2.6 Hz), 115.96 (d, $^2J_{CF}$ = 21.9 Hz), 111.33 ppm. ^{19}F NMR (470 MHz, 25 °C, $CDCl_3$): δ = −111.93 to −111.99 ppm (m).

2-Bromo-5-[(E)-2-(3,5-difluorophenyl)ethen-1-yl]thiophene ***21b***

The title compound was synthesized from thiophene derivative **16b** (333 mg) and NBS (280 mg) by following the general procedure *ii*. Yield: 375 mg (83 %); pale yellow solid; R_f = 0.8 (SiO_2, CH_2Cl_2/hex 1:4); m.p. = 65–70 °C. EI/MS (70 eV): *m/z* (%): 302 (M^+, 100), 220 (90), 188 (75), 177 (57), 151 (31), 110 (25). ^1H NMR (500 MHz, $CDCl_3$, 25 °C): δ = 7.08 (d, J=16.1 Hz, 1H, CH), 6.96 (d, J = 3.7 Hz, 1H, CH_{Th}), 6.93–6.92 (m, 2H, CH_{Ph}), 6.83 (d, J = 3.7 Hz, 1H, CH_{Th}), 6.70–6.67 ppm (m, 2H, CH+CH_{Ph}). ^{13}C NMR (125 MHz, $CDCl_3$, 25 °C): δ = 163.55 (d, $^1J_{CF}$ = 248.1 Hz), 163.44 (d, $^1J_{CF}$ = 248.0 Hz), 143.50, 140.18 (t, $^3J_{CF}$ = 9.4 Hz), 130.87, 127.75, 126.54 (t, $^4J_{CF}$ = 2.9 Hz), 123.81, 112.64, 109.18–108.98 (m), 103.18 ppm (t, $^2J_{CF}$ = 25.8 Hz). ^{19}F NMR (470 MHz, 25 °C, $CDCl_3$): δ = −108.30 to −108.33 ppm (m).

2-Bromo-5-[(E)-2-(2,4,6-trifluorophenyl)ethen-1-yl]thiophene ***21c***

The title compound was synthesized from thiophene derivative **16c** (360 mg) and NBS (280 mg) by following the general procedure *ii*. Yield: 330 mg (69 %); pale yellow solid; R_f = 0.8 (SiO_2, CH_2Cl_2/hex 1:4); m.p. = 53–58 °C. EI/MS (70 eV): *m/z* (%): 320 (M^+, 100), 238 (23), 220 (20), 195 (25). ^1H NMR (500 MHz, $CDCl_3$, 25 °C): δ = 7.34 (d, J = 16.5 Hz, 1H, CH), 6.96 (d, J = 3.6 Hz, 1H, CH_{Th}), 6.82 (d, J = 3.8 Hz, 1H, CH_{Th}), 6.74–6.66 ppm (m, 3H, CH+CH_{Ph}). ^{13}C NMR (125 MHz, $CDCl_3$, 25 °C): δ = 162.49–159.99 (2×dm, 2×C–F), 144.66, 130.80, 127.44, 127.24–127.08 (m), 114.30, 112.50, 111.24–110.95 (m), 101.08–100.49 ppm

(m). ^{19}F NMR (470 MHz, 25 °C, $CDCl_3$): δ = −107.13 to −107.19 (m, 1F), −107.89 to −107.94 ppm (m, 2F).

*2-Bromo-5-[(E)-2-(pentafluorophenyl)ethen-1-yl]thiophene **21d***

The title compound was synthesized from thiophene derivative **16d** (414 mg) and NBS (334 mg; 1.875 mmol) by following the general procedure *ii*. Yield: 340 mg (64 %); yellow solid; R_f = 0.8 (SiO_2, CH_2Cl_2/hex 1:4); m.p. = 103–107 °C. EI/MS (70 eV): *m/z* (%): 356 (M^+, 100), 336 (12), 275 (18), 256 (49), 231 (29), 225 (43), 211 (17). ^1H NMR (500 MHz, $CDCl_3$, 25 °C): δ = 7.41 (d, J = 16.5 Hz, 1H, CH), 6.99 (d, J = 3.7 Hz, 1H, CH_{Th}), 6.88 (d, J = 3.4 Hz, 1H, CH_{Th}), 6.66 ppm (d, J = 16.5 Hz, 1H, CH). ^{13}C NMR (125 MHz, $CDCl_3$, 25 °C): δ = 144.89 (dm), 143.66, 139.93 (dm), 137.95 (dm), 131.01, 129.59–129.43 (m), 128.69, 113.84, 112.79, 112.16–111.91 ppm (m). ^{19}F NMR (470 MHz, 25 °C, $CDCl_3$): δ = −141.07 (d, J = 17.7 Hz, 2F), −154.48 (t, J = 20.9 Hz, 1F), −161.17 ppm (dt, J_1 = 21.1 Hz, J_2 = 8.5 Hz, 2F).

*2-Bromo-5-{(E)-2-[4-(trifluoromethyl)phenyl]ethen-1-yl}thiophene **22a***

The title compound was synthesized from thiophene derivative **17a** (381 mg) and NBS (280 mg) by following the general procedure *ii*. Yield: 385 mg (77 %); pale yellow solid; R_f = 0.8 (SiO_2, CH_2Cl_2/hex 1:4); m.p. = 101–103 °C. EI/MS (70 eV): *m/z* (%): 334 (M^+, 100), 252 (30), 233 (20), 220 (38), 209 (20) 184 (59). ^1H NMR (500 MHz, $CDCl_3$, 25 °C): δ = 7.57 (d, J = 8.2 Hz, 2H, CH_{Ph}), 7.51 (d, J = 8.2 Hz, 2H, CH_{Ph}), 7.17 (d, J = 16.1 Hz, 1H, CH), 6.96 (d, J = 3.9 Hz, 1H, CH_{Th}), 6.84 (d, J = 3.9 Hz, 1H, CH_{Th}), 6.80 ppm (d, J = 16.1 Hz, 1H, CH). ^{13}C NMR (125 MHz, $CDCl_3$, 25 °C): δ = 143.91, 140.25, 130.85, 129.63 (q, $^2J_{CF}$ = 32.7 Hz), 127.57, 127.19, 126.58, 125.91 (q, $^3J_{CF}$ = 3.9 Hz), 124.34 (q, $^1J_{CF}$ = 271.5 Hz), 123.71, 112.47 ppm. ^{19}F NMR (470 MHz, 25 °C, $CDCl_3$): δ = −60.88 ppm.

*2-{(E)-2-[3,5-Bis(trifluoromethyl)phenyl]ethen-1-yl}-5-bromothiophene **22b***

The title compound was synthesized from thiophene derivative **17b** (483 mg) and NBS (280 mg) by following the general procedure *ii*. Yield: 439 mg (73 %); pale yellow solid; R_f = 0.8 (SiO_2, CH_2Cl_2/hex 1:4); m.p. = 75–78 °C. EI/MS (70 eV): *m/z* (%): 400 (M^+, 100), 320 (13), 301 (21), 288 (13), 252 (46). ^1H NMR (500 MHz, $CDCl_3$, 25 °C): δ = 7.84 (s, 2H, CH_{Ph}), 7.73 (s, 1H, CH_{Ph}), 7.23 (d, J = 16.1 Hz, 1H, CH), 6.99 (d, J = 3.9 Hz, 1H, CH_{Th}), 6.90 (d, J = 3.8 Hz, 1H, CH_{Th}), 6.82 ppm (d, J = 16.2 Hz, 1H, CH). ^{13}C NMR (125 MHz, $CDCl_3$, 25 °C): δ = 143.14, 138.93, 132.33 (q, $^2J_{CF}$ = 33.3 Hz), 131.02, 128.39, 126.12 (q, $^3J_{CF}$ = 3.0 Hz), 125.43, 124.97, 123.46 (q, $^1J_{CF}$ = 272.4 Hz), 121.19–121.06 (m), 113.30 ppm. ^{19}F NMR (470 MHz, 25 °C, $CDCl_3$): δ = −61.44 ppm.

*2-Bromo-5-{(E)-2-[2,4,6-tris(trifluoromethyl)phenyl]ethen-1-yl}thiophene **22c***

The title compound was synthesized from thiophene derivative **17c** (585 mg) and NBS (294 mg; 1.65 mmol) by following the general procedure *ii*, while the reaction time was extended to 60 h. Yield: 542 mg (77 %); white solid; R_f = 0.8 (SiO_2, CH_2Cl_2/hex 1:4); m.p. = 74–78 °C. EI/MS (70 eV): *m/z* (%): 470 (M^+, 65), 320 (100). ^1H NMR (500 MHz, $CDCl_3$, 25 °C): δ = 8.13 (s, 2H, CH_{Ph}), 6.98 (d, J = 3.9 Hz, 1H, CH_{Th}), 6.91 (d, J = 16.4 Hz, 1H, CH), 6.84 (d, J = 3.9 Hz, 1H, CH_{Th}), 6.69 ppm (d, J = 16.4 Hz, 1H, CH). ^{13}C NMR (125 MHz, $CDCl_3$, 25 °C): δ = 142.37, 140.62, 132.29 (q, $^2J_{CF}$ = 30.7 Hz), 130.82, 130.61, 130.35, 128.48, 126.87–126.74 (m), 122.86 (q, $^1J_{CF}$ = 275.1 Hz), 118.74, 113.75 ppm. ^{19}F NMR (470 MHz, 25 °C, $CDCl_3$): δ = −57.17 (s, 6F), −61.39 ppm (s, 3F).

2-Bromo-5-{(E)-2-[4-(pentafluorosulfanyl)phenyl]ethen-1-yl}thiophene ***22d***

The title compound was synthesized from thiophene derivative **17d** (468 mg) and NBS (280 mg) by following the general procedure *ii*. Yield: 440 mg (75 %); yellow solid; R_f = 0.9 (SiO_2, CH_2Cl_2/hex 1:4); m.p. = 115–121 °C. EI/MS (70 eV): *m/z* (%): 392 (M^+, 100), 202 (10), 184 (54), 139 (13). ^{1}H NMR (500 MHz, $CDCl_3$, 25 °C): δ = 7.70 (d, J = 8.6 Hz, 2H, CH_{Ph}), 7.48 (d, J = 8.3 Hz, 2H, CH_{Ph}), 7.17 (d, J = 16.3 Hz, 1H, CH), 6.97 (d, J = 4.2 Hz, 1H, CH_{Th}), 6.86 (d, J = 3.8 Hz, 1H, CH_{Th}), 6.77 ppm (d, J = 15.9 Hz, 1H, CH). ^{13}C NMR (125 MHz, $CDCl_3$, 25 °C): δ = 143.67, 140.08, 130.91, 127.91, 126.68–126.61 (m), 126.40, 126.34, 124.43, 112.82 ppm. ^{19}F NMR (470 MHz, 25 °C, $CDCl_3$): δ = 86.54 (kv, J = 152.9 Hz, 1F, SF_5), 64.59 ppm (d, J = 150.6 Hz, 4F, SF_5).

2-Bromo-5-{(E)-2-[3,5-bis(pentafluorosulfanyl)phenyl]ethen-1-yl}thiophene ***22e***

The title compound was synthesized from thiophene derivative **17e** (657 mg) and NBS (280 mg) by following the general procedure *ii*. Yield: 597 mg (77 %); yellow solid; R_f = 0.9 (SiO_2, CH_2Cl_2/hex 1:4); m.p. = 162–168 °C. EI/MS (70 eV): *m/z* (%): 518 (M^+, 100), 391 (10), 202 (24), 183 (14), 157 (10), 139 (15). ^{1}H NMR (500 MHz, $CDCl_3$, 25 °C): δ = 7.97–7.92 (m, 3H, CH_{Ph}), 7.22 (d, J = 16.2 Hz, 1H, CH), 7.00 (d, J = 3.8 Hz, 1H, CH_{Th}), 6.93 (d, J = 4.0 Hz, 1H, CH_{Th}), 6.79 ppm (d, J = 16.0 Hz, 1H, CH). ^{13}C NMR (125 MHz, $CDCl_3$, 25 °C): δ = 154.43–154.04 (m, CSF_5), 142.67, 139.17, 131.12, 128.93, 126.45–126.30 (m), 125.87, 124.68, 122.71–122.56 (m), 113.87 ppm. ^{19}F NMR (470 MHz, 25 °C, $CDCl_3$): δ = 83.49 (kv, J = 152.4 Hz, 1F, SF_5), 64.49 ppm (d, J = 150.4 Hz, 4F, SF_5).

2-Bromo-5-{(E)-2-[4-(trifluoromethoxy)phenyl]ethen-1-yl}thiophene ***23a***

The title compound was synthesized from thiophene derivative **18a** (405 mg) and NBS (280 mg) by following the general procedure *ii*. Yield: 508 mg (97 %); yellow solid; R_f = 0.8 (SiO_2, CH_2Cl_2/hex 1:4); m.p. = 42–45 °C. EI/MS (70 eV): *m/z* (%): 350 (M^+, 100), 268 (13), 236 (34), 225 (13), 184 (33), 171 (40), 139 (19), 69 (22). ^{1}H NMR (500 MHz, $CDCl_3$, 25 °C): δ = 7.43 (d, J = 8.3 Hz, 2H, CH_{Ph}), 7.17 (d, J = 8.5 Hz, 2H, CH_{Ph}), 7.05 (d, J = 16.0 Hz, 1H, CH), 6.95 (d, J = 3.9 Hz, 1H, CH_{Th}), 6.80 (d, J = 3.9 Hz, 1H, CH_{Th}), 6.77 ppm (d, J = 16.0 Hz, 1H, CH). ^{13}C NMR (125 MHz, $CDCl_3$, 25 °C): δ = 148.80, 144.19, 135.54, 130.75, 127.71, 127.25, 126.96, 122.30, 121.45, 120.67 (q, $^1J_{CF}$ = 256.0 Hz), 111.82 ppm.^{19}F NMR (470 MHz, 25 °C, $CDCl_3$): δ = −56.21 ppm.

2-Bromo-5-[(E)-2-{4-[(trifluoromethyl)sulfanyl]phenyl}ethen-1-yl]thiophene ***23b***

The title compound was synthesized from thiophene derivative **18b** (429 mg) and NBS (280 mg) by following the general procedure *ii*. Yield: 508 mg (93 %); yellow solid; R_f = 0.8 (SiO_2, CH_2Cl_2/hex 1:4); m.p. = 59–61 °C. EI/MS (70 eV): *m/z* (%): 366 (M^+, 100), 297 (35), 216 (14), 184 (40), 171 (28), 139 (14). ^{1}H NMR (500 MHz, $CDCl_3$, 25 °C): δ = 7.60 (d, J = 8.1 Hz, 2H, CH_{Ph}), 7.46 (d, J = 8.1 Hz, 2H, CH_{Ph}), 7.15 (d, J = 16.2 Hz, 1H, CH), 6.96 (d, J = 3.7 Hz, 1H, CH_{Th}), 6.84 (d, J = 3.7 Hz, 1H, CH_{Th}), 6.78 ppm (d, J = 16.2 Hz, 1H, CH). ^{13}C NMR (125 MHz, $CDCl_3$, 25 °C): δ = 143.96, 139.44, 136.90, 130.85, 129.70 (q, $^1J_{CF}$ = 308.6 Hz), 127.53, 127.31, 127.21, 123.62, 123.32, 112.45 ppm. ^{19}F NMR (470 MHz, 25 °C, $CDCl_3$): δ = −41.12 ppm.

1-{4-[(E)-2-(5-Bromothiophen-2-yl)ethen-1-yl]phenyl}-2,2,2-trifluoroethan-1-one ***24a***

The title compound was synthesized from thiophene derivative **19a** (423 mg) and NBS (280 mg) by following the general procedure *ii*. Yield: 433 mg (80 %); yellow solid; R_f = 0.6 (SiO_2, CH_2Cl_2/hex 1:4); m.p. = 72–77 °C. EI/MS (70 eV): *m/z* (%): 362 (M^+, 90), 293 (84), 184 (100), 152 (24), 139 (43), 92 (28). ^1H NMR (500 MHz, $CDCl_3$, 25 °C): δ = 8.03 (d, J = 8.0 Hz, 2H, CH_{Ph}), 7.56 (d, J = 8.3 Hz, 2H, CH_{Ph}), 7.28 (d, J = 16.1 Hz, 1H, CH), 6.99 (d, J = 3.8 Hz, 1H, CH_{Th}), 6.90 (d, J = 3.7 Hz, 1H, CH_{Th}), 6.82 ppm (d, J = 16.0 Hz, 1H, CH). ^{13}C NMR (125 MHz, $CDCl_3$, 25 °C): δ = 179.86 (q, $^2J_{CF}$ = 35.4 Hz), 143.94, 143.61, 131.05, 130.97, 128.80, 128.51, 126.81, 126.76, 125.63, 116.96 (q, $^1J_{CF}$ = 291.7 Hz), 113.53 ppm. ^{19}F NMR (470 MHz, 25 °C, $CDCl_3$): δ = −69.60 ppm.

2-Bromo-5-{(E)-2-[4-(trifluoromethanesulfonyl)phenyl]ethen-1-yl}thiophene ***24b***

The title compound was synthesized from thiophene derivative **19b** (477 mg) and NBS (280 mg) by following the general procedure *ii*. Yield: 453 mg (76 %); yellow solid; R_f = 0.3 (SiO_2, CH_2Cl_2/hex 1:4); m.p. = 105–110 °C. EI/MS (70 eV): *m/z* (%): 398 (M^+, 94), 281 (16), 265 (60), 184 (100), 152 (29), 139 (36). ^1H NMR (500 MHz, $CDCl_3$, 25 °C): δ = 7.97 (d, J = 8.4 Hz, 2H, CH_{Ph}), 7.67 (d, J = 8.4 Hz, 2H, CH_{Ph}), 7.31 (d, J = 16.0 Hz, 1H, CH), 7.00 (d, J = 3.8 Hz, 1H, CH_{Th}), 6.92 (d, J = 3.8 Hz, 1H, CH_{Th}),6.82 ppm (d, J = 16.4 Hz, 1H, CH). ^{13}C NMR (125 MHz, $CDCl_3$, 25 °C): δ = 145.24, 143.14, 131.52, 131.14, 129.23, 129.05, 127.33, 126.76, 125.78, 120.03 (q, $^1J_{CF}$ = 325.8 Hz), 114.09 ppm. ^{19}F NMR (470 MHz, 25 °C, $CDCl_3$): δ = −76.79 ppm.

4-[(E)-2-(5-Bromothiophen-2-yl)ethen-1-yl]phenyl trifluoromethanesulfonate ***24c***

The title compound was synthesized from thiophene derivative **19c** (501 mg) and NBS (280 mg) by following the general procedure *ii*. Yield: 477 mg (77 %); pale yellow viscous oil; R_f = 0.6 (SiO_2, CH_2Cl_2/hex 1:4). EI/MS (70 eV): *m/z* (%): 414 (M^+, 40), 281 (98), 171 (100), 69 (24). ^1H NMR (500 MHz, $CDCl_3$, 25 °C): δ = 7.48 (d, J = 8.7 Hz, 2H, CH_{Ph}), 7.23 (d, J = 8.7 Hz, 2H, CH_{Ph}), 7.09 (d, J = 16.1 Hz, 1H, CH), 6.96 (d, J = 3.7 Hz, 1H, CH_{Th}), 6.83 (d, J = 3.9 Hz, 1H, CH_{Th}), 6.76 ppm (d, J = 16.1 Hz, 1H, CH). ^{13}C NMR (125 MHz, $CDCl_3$, 25 °C): δ = 148.88, 143.84, 137.27, 130.84, 128.01, 127.45, 126.60, 123.34, 121.93, 118.94 (q, $^1J_{CF}$ = 320.8 Hz), 112.34 ppm. ^{19}F NMR (470 MHz, 25 °C, $CDCl_3$): δ = −71.11 ppm.

Boronic acid pinacol ester ***25a***

The title compound was synthesized from 2-bromothiophene derivative **20a** (265 mg) and vinylboronic acid pinacol ester **6** (231 mg) by following the general procedure *iii*. Yield: 247 mg (73 %); pale orange solid; R_f = 0.6 (SiO_2, CH_2Cl_2/hex 4:3); m.p. = 99–106 °C. EI/MS (70 eV): *m/z* (%): 338 (M^+, 100), 222 (32), 207 (18), 178 (24). ^1H NMR (500 MHz, $CDCl_3$, 25 °C): δ = 7.45 (d, J = 7.4 Hz, 2H, CH_{Ph}), 7.41 (d, J = 17.9 Hz, 1H, CH), 7.34 (t, J = 7.4 Hz, 2H, CH_{Ph}), 7.24 (t, J = 7.4 Hz, 1H, CH_{Ph}), 7.15 (d, J = 15.9 Hz, 1H, CH), 6.96–6.90 (m, 3H, CH+CH_{Th}), 5.87 (d, J = 18.1 Hz, 1H, CH), 1.30 ppm (s, 12H, CH_3). ^{13}C NMR (125 MHz, $CDCl_3$, 25 °C): δ = 143.97, 142.88, 141.99, 136.96, 129.44, 129.02, 128.93, 128.02, 127.20, 126.63, 121.94, 83.60, 25.01 ppm.

Boronic acid pinacol ester **25b**

The title compound was synthesized from 2-bromothiophene derivative **20b** (295 mg) and vinylboronic acid pinacol ester **6** (231 mg) by following the general procedure *iii*. Yield: 287 mg (78 %); orange solid; R_f = 0.5 (SiO_2, CH_2Cl_2/hex 4:3); m.p. = 95–101 °C. EI/MS (70 eV): *m/z* (%): 368 (M^+, 100), 252 (12). ^{1}H NMR (500 MHz, $CDCl_3$, 25 °C): δ = 7.42–7.39 (m, 3H, CH+CH_{Ph}), 7.01 (d, J = 16.0 Hz, 1H, CH), 6.94 (d, J = 3.6 Hz, 1H, CH_{Th}), 6.89–6.86 (m, 4H, CH+CH_{Th}+CH_{Ph}), 5.85 (d, J = 17.9 Hz, 1H, CH), 3.82 (s, 3H, OCH_3), 1.30 ppm (s, 12H, CH_3). 13C NMR (125 MHz, CDCl3, 25 °C): δ = 159.67, 144.46, 142.28, 142.09, 137.28, 129.76, 129.13, 129.08, 127.91, 126.47, 119.94, 114.40, 83.57, 55.54, 25.01 ppm.

Boronic acid pinacol ester **25c**

The title compound was synthesized from 2-bromothiophene derivative **20c** (307 mg) and vinylboronic acid pinacol ester **6** (231 mg) by following the general procedure *iii*. Yield: 300 mg (79 %); orange solid; R_f = 0.6 (SiO_2, CH_2Cl_2); m.p. = 116–124 °C. EI/MS (70 eV): *m/z* (%): 380 (M^+, 100), 265 (14). ^{1}H NMR (500 MHz, $CDCl_3$, 25 °C): δ = 7.91 (d, J = 8.3 Hz, 2H, CH_{Ph}), 7.51 (d, J = 7.9 Hz, 2H, CH_{Ph}), 7.40 (d, J = 18.0 Hz, 1H, CH), 7.25 (d, J = 16.2 Hz, 1H, CH), 6.99–6.97 (m, 2H, CH_{Th}), 6.91 (d, J = 16.2 Hz, 1H, CH), 5.90 (d, J = 18.1 Hz, 1H, CH), 2.58 (s, 3H, $COCH_3$), 1.29 ppm (s, 12H, CH_3). ^{13}C NMR (125 MHz, $CDCl_3$, 25 °C): δ = 197.59, 143.86, 143.17, 141.75, 141.64, 136.13, 129.09, 129.01, 128.38, 127.91, 126.55, 124.53, 83.65, 26.79, 24.99 ppm.

Boronic acid pinacol ester **26a**

The title compound was synthesized from 2-bromothiophene derivative **21a** (283 mg) and vinylboronic acid pinacol ester **6** (231 mg) by following the general procedure *iii*. Yield: 263 mg (74 %); slowly solidified orange solid; R_f = 0.6 (SiO_2, CH_2Cl_2/hex 4:3); m.p. = 105–115 °C. EI/MS (70 eV): *m/z* (%): 356 (M^+, 100), 256 (11), 240 (22). ^{1}H NMR (500 MHz, $CDCl_3$, 25 °C): δ = 7.47–7.42 (m, 3H, CH+CH_{Ph}), 7.10–7.04 (m, 3H, CH+CH_{Ph}), 6.99 (d, J = 3.8 Hz, 1H, CH_{Th}), 6.95 (d, J = 3.6 Hz, 1H, CH_{Th}), 6.90 (d, J = 16.1 Hz, 1H, CH), 5.90 (d, J = 18.1 Hz, 1H, CH), 1.33 ppm (s, 12H, CH_3). ^{13}C NMR (125 MHz, $CDCl_3$, 25 °C): δ = 162.61 (d, $^1J_{CF}$ = 247.9 Hz), 143.73, 142.87, 141.95, 133.16 (d, $^4J_{CF}$ = 3.3 Hz), 129.00, 128.19, 128.13 (d, $^3J_{CF}$ = 8.2 Hz), 127.20, 121.73 (d, $^5J_{CF}$ = 2.5 Hz), 115.93 (d, $^2J_{CF}$ = 21.9 Hz), 83.62, 25.01 ppm. ^{19}F NMR (470 MHz, 25 °C, $CDCl_3$): δ = −112.04 to −112.10 ppm (m).

Boronic acid pinacol ester **26b**

The title compound was synthesized from 2-bromothiophene derivative **21b** (301 mg) and vinylboronic acid pinacol ester **6** (231 mg) by following the general procedure *iii*. Yield: 251 mg (67 %); orange solid; R_f = 0.6 (SiO_2, CH_2Cl_2/hex 4:3); m.p. = 86–93 °C. EI/MS (70 eV): *m/z* (%): 374 (M^+, 100), 301 (11), 274 (13), 258 (25), 214 (11). ^{1}H NMR (500 MHz, $CDCl_3$, 25 °C): δ = 7.40 (d, J =17.8 Hz, 1H, CH), 7.13 (d, J =16.0 Hz, 1H, CH), 6.97–6.93 (m, 4H, CH_{Ph}+CH_{Th}), 6.79 (d, J =16.0 Hz, 1H, CH), 6.69–6.65 (m, 1H, CH_{Ph}), 5.89 (d, J = 18.0 Hz, 1H, CH), 1.30 ppm (s, 12H, CH_3). ^{13}C NMR (125 MHz, $CDCl_3$, 25 °C): δ = 163.53 (d, $^1J_{CF}$ = 247.7 Hz), 163.43 (d, $^1J_{CF}$ = 247.7 Hz), 143.92, 142.61, 141.72, 140.39 (t, $^3J_{CF}$ = 9.6 Hz), 128.95, 128.45, 126.93 (t, $^4J_{CF}$ = 3.0 Hz), 124.40, 112.64, 109.21–109.01 (m), 103.07 (t, $^2J_{CF}$ = 25.8 Hz), 83.68, 25.01. ^{19}F NMR (470 MHz, 25 °C, $CDCl_3$): δ = −108.42 to −108.46 ppm (m).

*Boronic acid pinacol ester **26c***

The title compound was synthesized from 2-bromothiophene derivative **21c** (319 mg) and vinylboronic acid pinacol ester **6** (231 mg) by following the general procedure *iii*. Yield: 243 mg (62 %); orange solid; R_f = 0.6 (SiO_2, CH_2Cl_2/hex 4:3); m.p. = 118–126 °C. EI/MS (70 eV): *m/z* (%): 392 (M^+, 100), 319 (12), 292 (21), 276 (41), 253 (16), 207 (54). ^{1}H NMR (500 MHz, $CDCl_3$, 25 °C): δ = 7.42–7.37 (m, 2H, 2×CH), 6.96 (s, 2H, CH_{Ph}), 6.83 (d, J = 16.6 Hz, 1H, CH), 6.70–6.66 (m, 2H, CH_{Th}), 5.89 (d, J = 18.0 Hz, 1H, CH), 1.29 ppm (s, 12H, CH_3). ^{13}C NMR (125 MHz, $CDCl_3$, 25 °C): δ = 162.42–160.02 (2×dm, 2×C–F), 143.79, 141.81, 135.00, 129.18, 128.93, 128.61, 128.09, 127.80–127.66 (m), 114.69, 111.41–111.17 (m), 101.06–100.61 (m), 83.64, 25.00 ppm. ^{19}F NMR (470 MHz, 25 °C, $CDCl_3$): δ = −107.32 to −107.38 (m, 1F), −107.73 to −107.80 ppm (m, 2F).

*Boronic acid pinacol ester **26d***

The title compound was synthesized from 2-bromothiophene derivative **21d** (355 mg) and vinylboronic acid pinacol ester **6** (231 mg) by following the general procedure *iii*. Yield: 287 mg (67 %); orange solid; R_f = 0.7 (SiO_2, CH_2Cl_2/hex 4:3); m.p. = 150–157 °C. EI/MS (70 eV): *m/z* (%): 428 (M^+, 100), 355 (10), 328 (30), 312 (35), 207 (15). ^{1}H NMR (500 MHz, $CDCl_3$, 25 °C): δ = 7.46 (d, J = 16.5 Hz, 1H, CH), 7.40 (d, J = 17.9 Hz, 1H, CH), 7.02 (d, J = 3.7 Hz, 1H, CH_{Th}), 6.98 (d, J = 4.0 Hz, 1H, CH_{Th}), 6.76 (d, J = 16.5 Hz, 1H, CH), 5.92 (d, J = 18.0 Hz, 1H, CH), 1.29 ppm (s, 12H, CH_3). ^{13}C NMR (125 MHz, $CDCl_3$, 25 °C): δ = 144.89 (dm), 144.84, 142.66, 141.54, 139.94 (dm), 137.97 (dm), 130.20–130.06 (m), 129.32, 128.89, 112.80, 112.35–112.09 (m), 83.72, 25.01 ppm. ^{19}F NMR (470 MHz, 25 °C, $CDCl_3$): δ = −140.86 (dd, J_1 = 21.2 Hz, J_2 = 7.5 Hz 2F), −154.60 (t, J = 20.7 Hz, 1F), −161.14 ppm (dt, J_1 = 21.1 Hz, J_2 = 7.3 Hz, 2F).

*Boronic acid pinacol ester **27a***

The title compound was synthesized from 2-bromothiophene derivative **22a** (333 mg) and vinylboronic acid pinacol ester **6** (231 mg) by following the general procedure *iii*. Yield: 288 mg (71 %); slowly solidified orange solid; R_f = 0.6 (SiO_2, CH_2Cl_2/hex 4:3); m.p. = 82–87 °C. EI/MS (70 eV): *m/z* (%): 406 (M^+, 100), 333 (10), 306 (13), 290 (26). ^{1}H NMR (500 MHz, $CDCl_3$, 25 °C): δ = 7.57 (d, J = 8.1 Hz, 2H, CH_{Ph}), 7.53 (d, J = 8.3 Hz, 2H, CH_{Ph}), 7.41 (d, J = 18.0 Hz, 1H, CH), 7.22 (d, J = 16.2 Hz, 1H, CH), 6.99–6.96 (m, 2H, CH_{Th}), 6.91 (d, J = 16.2 Hz, 1H, CH), 5.90 (d, J = 18.0 Hz, 1H, CH), 1.30 ppm (s, 12H, CH_3). ^{13}C NMR (125 MHz, $CDCl_3$, 25 °C): δ = 143.78, 143.07, 141.77, 140.45, 129.49 (q, $^2J_{CF}$ = 32.7 Hz), 128.97, 128.28, 127.58, 126.63, 125.87 (q, $^3J_{CF}$ = 3.9 Hz), 124.37 (q, $^1J_{CF}$ = 271.6 Hz), 124.30, 83.67, 25.01 ppm. ^{19}F NMR (470 MHz, 25 °C, $CDCl_3$): δ = −61.10 ppm.

*Boronic acid pinacol ester **27b***

The title compound was synthesized from 2-bromothiophene derivative **22b** (401 mg) and vinylboronic acid pinacol ester **6** (231 mg) by following the general procedure *iii*. Yield: 313 mg (66 %); slowly solidified orange solid; R_f = 0.8 (SiO_2, CH_2Cl_2/hex 4:3); m.p. = 118–128 °C. EI/MS (70 eV): *m/z* (%): 474 (M^+, 100), 401 (13), 374 (30), 358 (19). ^{1}H NMR (500 MHz, $CDCl_3$, 25 °C): δ = 7.85 (s, 2H, CH_{Ph}), 7.71 (s, 1H, CH_{Ph}), 7.41 (d, J = 17.9 Hz, 1H, CH), 7.28 (d, J = 16.1 Hz, 1H, CH), 7.03 (d, J = 3.7 Hz, 1H, CH_{Th}), 6.99 (d, J = 3.8 Hz, 1H, CH_{Th}), 6.92 (d, J = 16.2 Hz, 1H, CH), 5.92 (d, J = 18.1 Hz, 1H, CH), 1.30 ppm (s, 12H, CH_3). ^{13}C NMR

(125 MHz, $CDCl_3$, 25 °C): δ = 144.42, 142.19, 141.60, 139.12, 132.27 (q, $^2J_{CF}$ = 33.2 Hz), 129.07, 128.96, 126.15 (q, $^3J_{CF}$ = 2.9 Hz), 125.78, 125.54, 123.49 (q, $^1J_{CF}$ = 273.3 Hz), 121.04–120.91 (m), 83.72, 25.00 ppm. ^{19}F NMR (470 MHz, 25 °C, $CDCl_3$): δ = −61.33 ppm.

*Boronic acid pinacol ester **27c***

The title compound was synthesized from 2-bromothiophene derivative **22c** (469 mg) and vinylboronic acid pinacol ester **6** (231 mg) by following the general procedure *iii*. Yield: 309 mg (57 %); pale orange viscous oil; R_f = 0.7 (SiO_2, CH_2Cl_2/hex 4:3). EI/MS (70 eV): *m/z* (%): 542 (M^+, 100), 469 (20), 457(19), 442 (37), 426 (39), 403 (16). ^1H NMR (500 MHz, $CDCl_3$, 25 °C): δ = 8.13 (s, 2H, CH_{Ph}), 7.40 (d, J = 18.1 Hz, 1H, CH), 7.01 (d, J = 16.5 Hz, 1H, CH), 6.98–6.96 (m, 2H, CH_{Th}), 6.74 (d, J = 16.3 Hz, 1H, CH), 5.93 (d, J = 18.1 Hz, 1H, CH), 1.29 ppm (s, 12H, CH_3). ^{13}C NMR (125 MHz, $CDCl_3$, 25 °C): δ = 144.84, 141.58, 141.37, 137.26, 132.28 (q, $^2J_{CF}$ = 30.5 Hz), 131.27, 130.33 (q, $^2J_{CF}$ = 34.5 Hz), 128.99, 128.69, 126.78, 122.88 (q, $^1J_{CF}$ = 274.8 Hz), 119.61, 83.69, 25.00 ppm. ^{19}F NMR (470 MHz, 25 °C, $CDCl_3$): δ = −57.16 (s, 6F), −61.41 ppm (s, 3F).

*Boronic acid pinacol ester **27d***

The title compound was synthesized from 2-bromothiophene derivative **22d** (391 mg) and vinylboronic acid pinacol ester **6** (231 mg) by following the general procedure *iii.* Yield: 255 mg (55 %); orange viscous oil; R_f = 0.7 (SiO_2, CH_2Cl_2/hex 4:3). EI/MS (70 eV): *m/z* (%): 464 (M^+, 100), 391 (10), 364 (10). ^1H NMR (500 MHz, $CDCl_3$, 25 °C): δ = 7.69 (d, J = 8.8 Hz, 2H, CH_{Ph}), 7.49 (d, J = 8.5 Hz, 2H, CH_{Ph}), 7.40 (d, J = 18.0 Hz, 1H, CH), 7.22 (d, J = 16.0 Hz, 1H, CH), 6.99 (d, J = 3.6 Hz, 1H, CH_{Th}), 6.97 (d, J = 3.6 Hz, 1H, CH_{Th}), 6.87 (d, J = 16.3 Hz, 1H, CH), 5.90 (d, J = 17.9 Hz, 1H, CH), 1.30 ppm (s, 12H, CH_3). ^{13}C NMR (125 MHz, $CDCl_3$, 25 °C): δ = 144.05, 142.76, 141.71, 140.28, 137.27, 130.79–130.75 (m), 128.97, 128.61, 126.76, 126.63–126.56 (m), 126.37, 125.02, 83.70, 25.01 ppm. ^{19}F NMR (470 MHz, 25 °C, $CDCl_3$): δ = 86.56 (kv, J = 151.1 Hz, 1F, SF_5), 64.69 ppm (d, J = 149.8 Hz, 4F, SF_5).

*Boronic acid pinacol ester **27e***

The title compound was synthesized from 2-bromothiophene derivative **22e** (517 mg) and vinylboronic acid pinacol ester **6** (231 mg) by following the general procedure *iii.* Yield: 195 mg (33 %); yellow solid; R_f = 0.7 (SiO_2, CH_2Cl_2/hex 4:3); m.p. = 195–208 °C. ^1H NMR (500 MHz, $CDCl_3$, 25 °C): δ = 7.96–7.93 (m, 3H, CH_{Ph}), 7.41 (d, J = 18.1 Hz, 1H, CH), 7.27 (d, J = 16.1 Hz, 1H, CH), 7.06 (d, J = 3.9 Hz, 1H, CH_{Th}), 7.00 (d, J = 3.7 Hz, 1H, CH_{Th}), 6.89 (d, J = 16.0 Hz, 1H, CH), 5.93 (d, J = 18.0 Hz, 1H, CH), 1.30 ppm (s, 12H, CH_3). ^{13}C NMR (125 MHz, $CDCl_3$, 25 °C): δ = 154.41–154.10 (m, CSF_5), 144.86, 141.68, 141.50, 139.38, 129.62, 128.97, 126.47, 126.43–126.33 (m), 124.99, 122.57–122.42 (m), 83.77, 25.02 ppm. ^{19}F NMR (470 MHz, 25 °C, $CDCl_3$): δ = 83.56 (kv, J = 150.8 Hz, 1F, SF_5), 64.48 ppm (d, J = 151.0 Hz, 4F, SF_5). HR-MALDI-MS (DHB): *m/z* calcd. for $C_{20}H_{21}BF_{10}O_2S_3$ $[M]^+$ 590.06315; found: 590.06451 (Δ = 2.29 ppm).

*Boronic acid pinacol ester **28a***

The title compound was synthesized from 2-bromothiophene derivative **23a** (349 mg) and vinylboronic acid pinacol ester **6** (231 mg) by following the general procedure *iii.* Yield: 333 mg (79 %); orange viscous oil; R_f = 0.7 (SiO_2, CH_2Cl_2/hex 4:3). EI/MS (70 eV): *m/z* (%): 422 (M^+, 100), 322 (13), 306 (23). ^1H NMR (500 MHz, $CDCl_3$, 25 °C): δ = 7.45 (d, J = 8.6 Hz, 2H,

CH_{Ph}), 7.40 (d, J = 18.0 Hz, 1H, CH), 7.17 (d, J = 8.3 Hz, 2H, CH_{Ph}), 7.11 (d, J = 15.9 Hz, 1H, CH), 6.96 (d, J = 3.8 Hz, 1H, CH_{Th}), 6.94 (d, J = 3.8 Hz, 1H, CH_{Th}), 6.88 (d, J = 16.1 Hz, 1H, CH), 5.88 (d, J = 18.0 Hz, 1H, CH), 1.30 ppm (s, 12H, CH_3). ^{13}C NMR (125 MHz, $CDCl_3$, 25 °C): δ = 148.75, 143.38, 143.28, 141.87, 137.27, 135.75, 128.99, 127.76, 127.68, 122.88, 121.43, 120.67 (q, $^1J_{CF}$ = 256.0 Hz), 83.64, 25.00 ppm. ^{19}F NMR (470 MHz, 25 °C, $CDCl_3$): δ = −56.16 ppm.

*Boronic acid pinacol ester **28b***

The title compound was synthesized from 2-bromothiophene derivative **23b** (364 mg) and vinylboronic acid pinacol ester **6** (231 mg) by following the general procedure *iii*. Yield: 315 mg (72 %); orange viscous oil; R_f = 0.7 (SiO_2, CH_2Cl_2/hex 4:3). EI/MS (70 eV): *m/z* (%): 438 (M^+, 100), 322 (12). ^{1}H NMR (500 MHz, $CDCl_3$, 25 °C): δ = 7.60 (d, J = 8.2 Hz, 2H, CH_{Ph}), 7.48 (d, J = 8.2 Hz, 2H, CH_{Ph}), 7.40 (d, J = 18.1 Hz, 1H, CH), 7.21 (d, J = 16.1 Hz, 1H, CH), 6.98–6.96 (m, 2H, CH_{Th}), 6.88 (d, J = 16.1 Hz, 1H, CH), 5.90 (d, J = 18.1 Hz, 1H, CH), 1.30 ppm (s, 12H, CH_3). ^{13}C NMR (125 MHz, $CDCl_3$, 25 °C): δ = 143.78, 143.10, 141.78, 139.65, 137.28, 136.88, 129.73 (q, $^1J_{CF}$ = 309.0 Hz), 128.99, 128.24, 127.61, 127.38, 124.22, 83.67, 25.01 ppm. ^{19}F NMR (470 MHz, 25 °C, $CDCl_3$): δ = −41.13 ppm.

*Boronic acid pinacol ester **29a***

The title compound was synthesized from 2-bromothiophene derivative **24a** (361 mg) and vinylboronic acid pinacol ester **6** (231 mg) by following the general procedure *iii*. Yield: 321 mg (74 %); red viscous oil; R_f = 0.5 (SiO_2, CH_2Cl_2/hex 4:3). EI/MS (70 eV): *m/z* (%): 434 (M^+, 100), 361 (10), 318 (17), 265 (16). ^{1}H NMR (500 MHz, $CDCl_3$, 25 °C): δ = 8.03 (d, J = 8.2 Hz, 2H, CH_{Ph}), 7.58 (d, J = 8.5 Hz, 2H, CH_{Ph}), 7.41 (d, J = 17.9 Hz, 1H, CH), 7.33 (d, J = 15.9 Hz, 1H, CH), 7.03 (d, J = 3.7 Hz, 1H, CH_{Th}), 6.99 (d, J = 3.7 Hz, 1H, CH_{Th}), 6.92 (d, J = 15.9 Hz, 1H, CH), 5.92 (d, J = 18.0 Hz, 1H, CH), 1.30 ppm (s, 12H, CH_3). ^{13}C NMR (125 MHz, $CDCl_3$, 25 °C): δ = 179.81 (q, $^2J_{CF}$ = 34.9 Hz), 144.63, 144.17, 142.66, 141.61, 137.26, 130.93, 129.25, 129.02, 127.13, 126.84, 126.27, 116.97 (q, $^1J_{CF}$ = 291.0 Hz), 83.72, 25.00 ppm. ^{19}F NMR (470 MHz, 25 °C, $CDCl_3$): δ = −69.56 ppm.

*Boronic acid pinacol ester **29b***

The title compound was synthesized from 2-bromothiophene derivative **24b** (397 mg) and vinylboronic acid pinacol ester **6** (231 mg) by following the general procedure *iii*. Yield: 301 mg (64 %); slowly solidified orange solid; R_f = 0.5 (SiO_2, CH_2Cl_2/hex 4:3); m.p. = 58–63 °C. EI/MS (70 eV): *m/z* (%): 470 (M^+, 84), 281 (45), 207 (100). ^{1}H NMR (500 MHz, $CDCl_3$, 25 °C): δ = 7.96 (d, J = 8.6 Hz, 2H, CH_{Ph}), 7.67 (d, J = 8.2 Hz, 2H, CH_{Ph}), 7.41 (d, J = 18.0 Hz, 1H, CH), 7.36 (d, J = 16.0 Hz, 1H, CH), 7.06 (d, J = 3.9 Hz, 1H, CH_{Th}), 7.00 (d, J = 3.5 Hz, 1H, CH_{Th}), 5.93 (d, J = 17.9 Hz, 1H, CH), 1.30 ppm (s, 12H, CH_3). ^{13}C NMR (125 MHz, $CDCl_3$, 25 °C): δ = 145.45, 145.05, 142.12, 141.48, 137.25, 131.45, 129.75, 128.99, 127.39, 127.34, 126.08, 120.03 (q, $^1J_{CF}$ = 326.0 Hz), 83.75, 24.99 ppm. ^{19}F NMR (470 MHz, 25 °C, $CDCl_3$): δ = −76.80 ppm.

*Boronic acid pinacol ester **29c***

The title compound was synthesized from 2-bromothiophene derivative **24c** (413 mg) and vinylboronic acid pinacol ester **6** (231 mg) by following the general procedure *iii*. Yield: 350 mg (72 %); slowly solidified orange solid; R_f = 0.7 (SiO_2, CH_2Cl_2/hex 4:3); m.p. = 62–68 °C. EI/MS

(70 eV): *m/z* (%): 486 (M^+, 22), 353 (100), 253 (12). ^{1}H NMR (500 MHz, $CDCl_3$, 25 °C): δ = 7.49 (d, *J* = 8.5 Hz, 2H, CH_{Ph}), 7.41 (d, *J* = 17.9 Hz, 1H, CH), 7.23 (d, *J* = 8.7 Hz, 2H, CH_{Ph}), 7.14 (d, *J* = 16.2 Hz, 1H, CH), 6.97–6.95 (m, 2H, CH_{Th}), 6.87 (d, *J* = 15.9 Hz, 1H, CH), 5.90 (d, *J* = 18.2 Hz, 1H, CH), 1.30 ppm (s, 12H, CH_3). ^{13}C NMR (125 MHz, $CDCl_3$, 25 °C): δ = 148.81, 143.66, 142.97, 141.76, 137.45, 128.96, 128.14, 128.04, 126.97, 123.91, 121.87, 118.93 (q, $^1J_{CF}$ = 321.2 Hz), 83. 66, 24.99 ppm. ^{19}F NMR (470 MHz, 25 °C, $CDCl_3$): δ = −71.12 ppm.

*Fluorophore **PM1***

The title target fluorophore was synthesized from tris(4-iodophenyl)amine **9** and boronic acid pinacol ester **25a** (183 mg) by following the general procedure *iv*. Yield: 93 mg (71 %); brick-orange solid; R_f = 0.7 (SiO_2, CH_2Cl_2/hex 1:1). ^{1}H NMR (500 MHz, $CDCl_3$, 25 °C): δ = 7.46 (d, *J* = 7.4 Hz, 6H, CH_{Ph}), 7.38–7.33 (m, 12H, CH_{Ph}), 7.26–7.23 (m, 3H, CH_{Ph}), 7.18 (d, *J* = 16.0 Hz, 3H; CH), 7.11–7.08 (m, 9H, CH_{Th}+CH_{Ph}), 6.95-6.85 ppm (m, 12H, CH_{Th}+CH). ^{13}C NMR (125 MHz, $CDCl_3$, 25 °C): δ = 146.77, 142.42, 141.77, 137.16, 132.07, 128.95, 128.56, 128.13, 127.84, 127.54, 127.46, 126.98, 126.52, 124.51, 122.10, 120.86 ppm. HR-MALDI-MS (DHB): *m/z* calcd. for $C_{60}H_{45}NS_3$ $[M]^+$ 875.2709; found: 875.2714 (Δ = 0.58 ppm).

*Fluorophore **PM2***

The title target fluorophore was synthesized from tris(4-iodophenyl)amine **9** and boronic acid pinacol ester **25b** (199 mg) by following the general procedure *iv*. Yield: 90 mg (62 %); orange solid; R_f = 0.5 (SiO_2, CH_2Cl_2/hex 1:1). ^{1}H NMR (500 MHz, $CDCl_3$, 25 °C): δ = 7.40–7.35 (m, 12H, CH_{Ph}), 7.10–7.03 (m, 12H, CH_{Ph}), 6.90–6.83 (m, 18H, CH+CH_{Th}), 3.82 ppm (s, 9H, CH_3). ^{13}C NMR (125 MHz, $CDCl_3$, 25 °C): δ = 159.54, 146.72, 142.22, 141.80, 132.12, 129.97, 128.25, 127.78, 127.48, 127.00, 126.69, 124.49, 120.93, 120.12, 114.42, 55.55 ppm. HR-MALDI-MS (DHB): *m/z* calcd. for $C_{63}H_{52}NO_3S_3$ $[M+H]^+$ 966.3104; found: 966.3080 (Δ = 2.43 ppm).

*Fluorophore **PM3***

The title target fluorophore was synthesized from tris(4-iodophenyl)amine **9** and boronic acid pinacol ester **25c** (205 mg) by following the general procedure *iv*. Yield: 83 mg (55 %); red solid; R_f = 0.7 (SiO_2, CH_2Cl_2/EtOAc 20:1). ^{1}H NMR (500 MHz, $CDCl_3$, 25 °C): δ = 7.92 (d, *J* = 7.9 Hz, 6H, CH_{Ph}), 7.51 (d, *J* = 8.0 Hz, 6H, CH_{Ph}), 7.36 (d, *J* = 8.1 Hz, 6H, CH_{Ph}), 7.28 (d, *J* = 16.2 Hz, 3H, CH), 7.11–7.06 (m, 9H, CH+CH_{Ph}), 7.00 (d, *J* = 3.6 Hz, 3H, CH_{Th}), 6.94 (d, *J* = 3.6 Hz, 3H, CH_{Th}), 6.87 (d, *J* = 16.1 Hz, 6H, CH+CH), 2.59 (s, 9H, CH_3). ^{13}C NMR (125 MHz, $CDCl_3$, 25 °C): δ = 197.59, 146.83, 143.48, 141.86, 141.07, 135.98, 131.96, 129.13, 128.72, 128.66, 127.64, 127.06, 127.04, 126.42, 124.69, 124.51, 120.70, 26.79 ppm. HR-MALDI-MS (DHB): *m/z* calcd. for $C_{66}H_{52}NO_3S_3$ $[M+H]^+$ 1002.3104; found: 1002.3075 (Δ = 2.85 ppm).

*Fluorophore **1a***

The title target fluorophore was synthesized from tris(4-iodophenyl)amine **9** and boronic acid pinacol ester **26a** (192 mg) by following the general procedure *iv*. Yield: 88 mg (63 %); brick-orange solid; R_f = 0.6 (SiO_2, CH_2Cl_2/hex 1:1). ^{1}H NMR (500 MHz, $CDCl_3$, 25 °C): δ = 7.43–7.40 (m, 6H, CH_{Ph}), 7.36 (d, *J* = 8.4 Hz, 6H, CH_{Ph}), 7.10–7.01 (m, 18H, CH+CH_{Ph}), 6.93–6.91 (m, 6H, CH_{Th}), 6.94 (d, *J* = 3.6 Hz, 3H, CH_{Th}), 6.88–6.83 ppm (m, 6H, CH). ^{13}C

NMR (125 MHz, $CDCl_3$, 25 °C): δ = 162.50 (d, $^1J_{CF}$ = 248.0 Hz), 146.76, 142.40, 141.53, 133.35 (d, $^4J_{CF}$ = 3.2 Hz), 132.04, 128.15, 127.98 (d, $^3J_{CF}$ = 8.0 Hz), 127.54, 127.44, 127.30, 126.98, 124.49, 121.87 (d, $^5J_{CF}$ = 1.7 Hz), 120.82, 115.92 ppm (d, $^2J_{CF}$ = 20.9 Hz). ^{19}F NMR (470 MHz, 25 °C, $CDCl_3$): δ = −112.54 ppm. HR-MALDI-MS (DHB): *m/z* calcd. for $C_{60}H_{43}F_3NS_3$ $[M+H]^+$ 930.2504; found: 930.2478 (Δ = 2.83 ppm).

*Fluorophore **1b***

The title target fluorophore was synthesized from tris(4-iodophenyl)amine **9** and boronic acid pinacol ester **26b** (202 mg) by following the general procedure *iv*. Yield: 94 mg (64 %); orange-red solid; R_f = 0.7 (SiO_2, CH_2Cl_2/hex 1:1). ^1H NMR (500 MHz, $CDCl_3$, 25 °C): δ = 7.37 (d, J = 8.4 Hz, 6H, CH_{Ph}), 7.17 (d, J = 16.0 Hz, 3H, CH), 7.11–7.08 (m, 9H, CH+CH_{Ph}), 6.99–6.94 (m, 12H, CH_{Ph}+CH_{Th}), 6.88 (d, J = 16.0 Hz, 3H, CH), 6.76 (d, J = 16.0 Hz, 3H, CH), 6.69–6.66 ppm (m, 3H, CH_{Ph}). ^{13}C NMR (125 MHz, $CDCl_3$, 25 °C): δ = 163.56 (d, $^1J_{CF}$ = 247.3 Hz), 163.46 (d, $^1J_{CF}$ = 247.3 Hz), 146.87, 143.51, 140.59 (t, $^3J_{CF}$ = 9.6 Hz), 140.49, 131.95, 128.76, 128.73, 127.65, 126.99, 126.05, 124.53, 120.67, 109.08–108.87 (m), 102.88 ppm (t, $^2J_{CF}$ = 25.6 Hz). ^{19}F NMR (470 MHz, 25 °C, $CDCl_3$): δ = −108.48 to −108.52 ppm (m). HR-MALDI-MS (DHB): *m/z* calcd. for $C_{60}H_{40}F_6NS_3$ $[M+H]^+$ 984.2222; found: 984.2198 (Δ = 2.43 ppm).

*Fluorophore **1c***

The title target fluorophore was synthesized from tris(4-iodophenyl)amine **9** and boronic acid pinacol ester **26c** (212 mg) by following the general procedure *iv*. Yield: 107 mg (69 %); orange solid; R_f = 0.7 (SiO_2, CH_2Cl_2/hex 1:1). ^1H NMR (500 MHz, $CDCl_3$, 25 °C): δ = 7.43 (d, J = 16.4 Hz, 3H, CH), 7.36 (d, J = 8.3 Hz, 6H, CH_{Ph}), 7.11–7.08 (m, 9H, CH+CH_{Ph}), 6.97 (d, J = 3.8 Hz, 3H, CH_{Th}), 6.93 (d, J = 3.5 Hz, 3H, CH_{Th}), 6.89 (d, J = 16.0 Hz, 3H, CH), 6.81 (d, J = 16.4 Hz, 3H, CH), 6.70–6.67 ppm (m, 6H, CH_{Ph}). ^{13}C NMR (125 MHz, $CDCl_3$, 25 °C): δ = 162.17–159.97 (2×dm, 2×C–F), 146.85, 143.38, 141.69, 132.00, 128.59, 128.48, 127.88, 127.61, 126.98, 124.53, 120.74, 113.79, 111.64–111.36 (m), 101.05–100.60 ppm (m). ^{19}F NMR (470 MHz, 25 °C, $CDCl_3$): δ = −107.72 to −107.75 (m, 3F), −107.99 to −108.02 ppm (m, 6F). HR-MALDI-MS (DHB): *m/z* calcd. for $C_{60}H_{37}F_9NS_3$ $[M+H]^+$ 1038.1939; found: 1038.1928 (Δ = 1.07 ppm).

*Fluorophore **1d***

The title target fluorophore was synthesized from tris(4-iodophenyl)amine **9** and boronic acid pinacol ester **26d** (231 mg) by following the general procedure *iv*. Yield: 93 mg (54 %); orange solid; R_f = 0.6 (SiO_2, CH_2Cl_2/hex 1:1). ^1H NMR (500 MHz, $CDCl_3$, 25 °C): δ = 7.49 (d, J = 16.6 Hz, 3H, CH), 7.37 (d, J = 8.6 Hz, 6H, CH_{Ph}), 7.11–7.08 (m, 9H, CH+CH_{Ph}), 7.03 (d, J = 3.7 Hz, 3H, CH_{Th}), 6.95 (d, J = 3.7 Hz, 3H, CH_{Th}), 6.91 (d, J = 16.1 Hz, 3H, CH), 6.73 ppm (d, J = 16.5 Hz, 3H, CH). ^{13}C NMR (125 MHz, $CDCl_3$, 25 °C): δ = 146.94, 144.86 (dm), 144.49, 140.64, 139.88 (dm), 137.88 (dm), 131.88, 130.29–130.16 (m), 129.75, 129.18, 127.72, 127.02, 124.55, 120.54, 112.57–112.33 (m), 111.83 ppm. ^{19}F NMR (470 MHz, 25 °C, $CDCl_3$): δ = −141.13 (dd, J_1 = 21.3 Hz, J_2 = 7.5 Hz, 6F), = −155.01 (t, J = 20.6 Hz, 3F), −161.22 ppm (dt, J_1 = 21.3 Hz, J_2 = 7.2 Hz, 6F). HR-MALDI-MS (DHB): *m/z* calcd. for $C_{60}H_{31}F_{15}NS_3$ $[M+H]^+$ 1146.1374; found: 1146.1360 (Δ = 1.17 ppm).

*Fluorophore **2a***

The title target fluorophore was synthesized from tris(4-iodophenyl)amine **9** and boronic acid pinacol ester **27a** (219 mg) by following the general procedure *iv*. Yield: 92 mg (57 %); orange-red solid; R_f = 0.6 (SiO_2, CH_2Cl_2/hex 1:1). ^{1}H NMR (500 MHz, $CDCl_3$, 25 °C): δ = 7.58 (d, J = 8.3 Hz, 6H, CH_{Ph}), 7.53 (d, J = 8.3 Hz, 6H, CH_{Ph}), 7.25 (d, J = 15.8 Hz, 3H, CH), 7.12–7.05 (m, 9H, CH+CH_{Ph}), 6.99 (d, J = 3.6 Hz, 3H, CH_{Th}), 6.94 (d, J = 3.6 Hz, 3H, CH_{Th}), 6.90–6.86 ppm (m, 6H, CH). ^{13}C NMR (125 MHz, $CDCl_3$, 25 °C): δ = 146.85, 143.36, 140.91, 140.64, 131.97, 129.31 (q, $^2J_{CF}$ = 33.3 Hz), 128.63, 128.58, 127.63, 127.00, 126.70, 126.50, 125.88 (q, $^3J_{CF}$ = 3.8 Hz), 124.52, 124.43, 124.40 (q, $^1J_{CF}$ = 272.1 Hz), 120.71 ppm. ^{19}F NMR (470 MHz, 25 °C, $CDCl_3$): δ = −60.98 ppm. HR-MALDI-MS (DHB): *m/z* calcd. for $C_{63}H_{42}F_9NS_3$ $[M]^+$ 1079.2330; found: 1079.2321 (Δ = 0.88 ppm).

*Fluorophore **2b***

The title target fluorophore was synthesized from tris(4-iodophenyl)amine **9** and boronic acid pinacol ester **27b** (256 mg) by following the general procedure *iv*. Yield: 108 mg (56 %); orange solid; R_f = 0.8 (SiO_2, CH_2Cl_2/hex 1:1). ^{1}H NMR (500 MHz, $CDCl_3$, 25 °C): δ = 7.85 (s, 6H, CH_{Ph}), 7.70 (s, 3H, CH_{Ph}), 7.38 (d, J = 8.4 Hz, 6H, CH_{Ph}), 7.31 (d, J = 16.0 Hz, 3H, CH), 7.12–7.09 (m, 9H, CH+CH_{Ph}), 7.05 (d, J = 3.7 Hz, 3H, CH_{Th}), 6.96 (d, J = 3.7 Hz, 3H, CH_{Th}), 6.92–6.87 ppm (m, 6H, CH). ^{13}C NMR (125 MHz, $CDCl_3$, 25 °C): δ = 146.93, 144.03, 140.12, 139.31, 132.27 (q, $^2J_{CF}$ = 33.3 Hz), 131.90, 129.39, 129.03, 127.72, 127.01, 126.78, 126.02, 125.66, 124.88, 124.54, 123.52 (q, $^1J_{CF}$ = 274.2 Hz), 120.77, 120.58 ppm. ^{19}F NMR (470 MHz, 25 °C, $CDCl_3$): δ = −61.35 ppm. HR-MALDI-MS (DHB): *m/z* calcd. for $C_{66}H_{40}F_{18}NS_3$ $[M+H]^+$ 1284.2030; found: 1284.2008 (Δ = 1.74 ppm).

*Fluorophore **2c***

The title target fluorophore was synthesized from tris(4-iodophenyl)amine **9** and boronic acid pinacol ester **27c** (293 mg) by following the general procedure *iv*. Yield: 125 mg (56 %); orange solid; R_f = 0.8 (SiO_2, CH_2Cl_2/hex 1:1). ^{1}H NMR (500 MHz, $CDCl_3$, 25 °C): δ = 8.24–8.14 (2×s, 6H, CH_{Ph}), 7.39–7.33 (m, 3.55H), 7.27–7.22 (m, 2.24H), 7.12–6.87 (m, 17.51H), 6.81–6.77 (m, 4.26H), 6.59–6.48 ppm (m, 2.78H). ^{19}F NMR (470 MHz, 25 °C, $CDCl_3$): δ = −57.12, −59.28, −61.23, −61.36 ppm. HR-MALDI-MS (DHB): *m/z* calcd. for $C_{69}H_{36}F_{27}NS_3$ $[M]^+$ 1487.1573; found: 1487.1566 (Δ = 0.47 ppm).

*Fluorophore **2d***

The title target fluorophore was synthesized from tris(4-iodophenyl)amine **9** and boronic acid pinacol ester **27d** (251 mg) by following the general procedure *iv*. Yield: 103 mg (55 %); red solid; R_f = 0.8 (SiO_2, CH_2Cl_2/hex 1:1). ^{1}H NMR (500 MHz, $CDCl_3$, 25 °C): δ = 7.70 (d, J = 8.7 Hz, 6H, CH_{Ph}), 7.49 (d, J = 8.4 Hz, 6H, CH_{Ph}), 7.38–7.36 (m, 6H, CH_{Ph}), 7.26 (d, J = 16.1 Hz, 3H, CH), 7.12–7.06 (m, 9H, CH+CH_{Ph}), 7.01 (d, J = 3.5 Hz, 3H, CH_{Th}), 6.95 (d, J = 3.5 Hz, 3H, CH_{Th}), 6.88–6.83 ppm (m, 6H, CH). ^{13}C NMR (125 MHz, $CDCl_3$, 25 °C): δ = 52.8–152.5 (m), 146.88, 143.64, 140.65, 140.50, 131.93, 128.93, 128.78, 127.66, 127.03, 126.65–126.58 (m), 126.23, 125.88, 125.14, 124.53, 120.66 ppm. ^{19}F NMR (470 MHz, 25 °C, $CDCl_3$): δ = 86.68 (kv, J = 149.3 Hz, 3F, SF_5), 64.74 ppm (d, J = 149.8 Hz, 12F, SF_5). HR-MALDI-MS (DHB): *m/z* calcd. for $C_{60}H_{43}F_{15}NS_6$ $[M+H]^+$ 1254.1475; found: 1254.1471 (Δ = 0.33 ppm).

*Fluorophore **2e***

The title target fluorophore was synthesized from tris(4-iodophenyl)amine **9** and boronic acid pinacol ester **27e** (319 mg) by following the general procedure *iv*. Yield: 142 mg (58 %); orange-red solid; R_f = 0.8 (SiO_2, CH_2Cl_2/hex 1:1). ^{1}H NMR (500 MHz, $CDCl_3$, 25 °C): δ = 7.95–7.93 (m, 9H, CH_{Ph}), 7.39 (d, J = 8.5 Hz, 6H, CH_{Ph}), 7.30 (d, J = 16.0 Hz, 3H, CH), 7.12–7.08 (m, 12H, CH+CH_{Ph}+CH_{Th}), 6.97 (d, J = 3.5 Hz, 3H, CH_{Th}), 6.91 (d, J = 16.0 Hz, 3H, CH), 6.86 ppm (d, J = 16.0 Hz, 3H, CH). ^{13}C NMR (125 MHz, $CDCl_3$, 25 °C): δ = 154.42–154.12 (m, CSF_5), 146.98, 144.49, 139.64, 139.57, 131.86, 129.94, 129.30, 127.77, 127.04, 126.33–126.18 (m), 124.56, 124.11, 122.37–122.16 (m), 120.51 ppm. ^{19}F NMR (470 MHz, 25 °C, $CDCl_3$): δ = 83.63 (kv, J = 150.9 Hz, 3F, SF_5), 64.45 ppm (d, J = 150.0 Hz, 12F, SF_5). HR-MALDI-MS (DHB): *m/z* calcd. for $C_{60}H_{39}F_{30}NS_9$ $[M]^+$ 1631.0084; found: 1631.0097 (Δ = 0.78 ppm).

*Fluorophore **3a***

The title target fluorophore was synthesized from tris(4-iodophenyl)amine **9** and boronic acid pinacol ester **28a** (228 mg) by following the general procedure *iv*. Yield: 112 mg (66 %); orange solid; R_f = 0.8 (SiO_2, CH_2Cl_2/hex 1:1). ^{1}H NMR (500 MHz, $CDCl_3$, 25 °C): δ = 7.46 (d, J = 8.5 Hz, 6H, CH_{Ph}), 7.36 (d, J = 8.5 Hz, 6H, CH_{Ph}), 7.19–7.08 (m, 18H, CH+CH_{Ph}), 6.95 (d, J = 3.5 Hz, 3H, CH_{Th}), 6.92 (d, J = 3.7 Hz, 3H, CH_{Th}), 6.89–6.83 ppm (m, 6H, CH). ^{13}C NMR (125 MHz, $CDCl_3$, 25 °C): δ = 148.61, 146.82, 142.83, 141.23, 135.96, 132.02, 128.37, 127.95, 127.65, 127.59, 126.98, 126.81, 124.52, 123.02, 121.47, 120.77, 120.68 ppm (q, $^1J_{CF}$ = 256.3 Hz). ^{19}F NMR (470 MHz, 25 °C, $CDCl_3$): δ = −56.15 ppm. HR-MALDI-MS (DHB): *m/z* calcd. for $C_{63}H_{42}F_9NO_3S_3$ $[M]^+$ 1127.2178; found: 1127.2218 (Δ = 3.57 ppm).

*Fluorophore **3b***

The title target fluorophore was synthesized from tris(4-iodophenyl)amine **9** and boronic acid pinacol ester **28b** (237 mg) by following the general procedure *iv*. Yield: 116 mg (66 %); red solid; R_f = 0.8 (SiO_2, CH_2Cl_2/hex 1:1). ^{1}H NMR (500 MHz, $CDCl_3$, 25 °C): δ = 7.60 (d, J = 8.2 Hz, 6H, CH_{Ph}), 7.47 (d, J = 8.2 Hz, 6H, CH_{Ph}), 7.38–7.35 (m, 6H, CH_{Ph}), 7.24 (d, J = 15.8 Hz, 3H, CH), 7.11–7.06 (m, 9H, CH+CH_{Ph}), 6.99 (d, J = 3.7 Hz, 3H, CH_{Th}), 6.94 (d, J = 3.7 Hz, 3H, CH_{Th}), 6.90–6.84 ppm (m, 6H, CH). ^{13}C NMR (125 MHz, $CDCl_3$, 25 °C): δ = 146.85, 143.36, 140.97, 139.84, 136.91, 131.97, 129.74 (q, $^1J_{CF}$ = 308.4 Hz), 128.62, 128.54, 127.63, 127.25, 127.02, 126.72, 124.52, 124.35, 122.90, 120.71ppm. ^{19}F NMR (470 MHz, 25 °C, $CDCl_3$): δ = −41.17 ppm. HR-MALDI-MS (DHB): *m/z* calcd. for $C_{63}H_{42}F_9NS_6$ $[M]^+$ 1175.1492; found: 1175.1483 (Δ = 0.83 ppm).

*Fluorophore **4a***

The title target fluorophore was synthesized from tris(4-iodophenyl)amine **9** and boronic acid pinacol ester **29a** (234 mg) by following the general procedure *iv*. Yield: 110 mg (63 %); wine-red solid; R_f = 0.2 (SiO_2, CH_2Cl_2/hex 1:1). ^{1}H NMR (500 MHz, $CDCl_3$, 25 °C): δ = 8.03 (d, J = 8.0 Hz, 6H, CH_{Ph}), 7.57 (d, J = 8.4 Hz, 6H, CH_{Ph}), 7.39–7.35 (m, 9H, CH+CH_{Ph}), 7.12–7.05 (m, 12H, CH+CH_{Th}+CH_{Ph}), 6.97–6.87 ppm (m, 9H, CH+CH_{Th}). ^{13}C NMR (125 MHz, $CDCl_3$, 25 °C): δ = 179.78 (q, $^2J_{CF}$ = 34.8 Hz), 146.92, 144.38, 144.30, 140.61, 131.90, 130.97, 129.62, 129.08, 128.49, 127.72, 127.14, 126.70, 126.42, 126.24, 124.54, 120.61, 117.00 ppm (q, $^1J_{CF}$ = 290.6 Hz). ^{19}F NMR (470 MHz, 25 °C, $CDCl_3$): δ = −69.52 ppm. HR-MALDI-MS (DHB): *m/z* calcd. for $C_{66}H_{42}F_9NO_3S_3$ $[M]^+$ 1163.2178; found: 1163.2165 (Δ = 1.05 ppm).

*Fluorophore **4b***

The title target fluorophore was synthesized from tris(4-iodophenyl)amine **9** and boronic acid pinacol ester **29b** (254 mg) by following the general procedure *iv*. Yield: 109 mg (57 %); red solid; R_f = 0.5 (SiO_2, CH_2Cl_2/hex 1:1). ^{1}H NMR (500 MHz, $CDCl_3$, 25 °C): δ = 7.96 (d, J = 8.1 Hz, 6H, CH_{Ph}), 7.67 (d, J = 8.5 Hz, 6H, CH_{Ph}), 7.41–7.38 (m, 9H, CH+CH_{Ph}), 7.13–7.08 (m, 12H, CH+CH_{Th}+CH_{Ph}), 6.97 (d, J = 3.4 Hz, 3H, CH_{Th}), 6.94–6.87 ppm (m, 6H, CH). ^{13}C NMR (125 MHz, $CDCl_3$, 25 °C): δ = 146.97, 145.66, 144.75, 140.11, 131.86, 131.49, 130.15, 129.35, 128.72, 127.77, 127.53, 127.20, 127.14, 125.21, 124.57, 120.53, 120.06 ppm (q, $^1J_{CF}$ = 325.5 Hz). ^{19}F NMR (470 MHz, 25 °C, $CDCl_3$): δ = −76.81 ppm. HR-MALDI-MS (DHB): *m/z* calcd. for $C_{63}H_{43}F_9NO_6S_6$ $[M+H]^+$ 1272.1265; found: 1272.1245 (Δ = 1.58 ppm).

*Fluorophore **4c***

The title target fluorophore was synthesized from tris(4-iodophenyl)amine **9** and boronic acid pinacol ester **29c** (262 mg) by following the general procedure *iv*. Yield: 121 mg (61 %); red solid; R_f = 0.6 (SiO_2, CH_2Cl_2/hex 1:1). ^{1}H NMR (500 MHz, $CDCl_3$, 25 °C): δ = 7.50 (d, J = 8.4 Hz, 6H, CH_{Ph}), 7.37 (d, J = 8.7 Hz, 6H, CH_{Ph}), 7.25–7.23 (m, 6H, CH_{Ph}), 7.17 (d, J = 16.2 Hz, 3H, CH), 7.11–7.08 (m, 9H, CH+CH_{Ph}), 6.98 (d, J = 3.6 Hz, 3H, CH_{Th}), 6.94 (d, J = 3.6 Hz, 3H, CH_{Th}), 6.89–6.83 ppm (m, 6H, CH). ^{13}C NMR (125 MHz, $CDCl_3$, 25 °C): δ = 148.69, 146.84, 143.23, 140.85, 137.65, 131.96, 128.58, 128.45, 127.91, 127.62, 127.00, 126.11, 124.52, 124.05, 121.91, 120.70, 118.94 ppm (q, $^1J_{CF}$ = 320.1 Hz). ^{19}F NMR (470 MHz, 25 °C, $CDCl_3$): δ = −71.10 ppm. HR-MALDI-MS (DHB): *m/z* calcd. for $C_{63}H_{43}F_9NO_9S_6$ $[M+H]^+$ 1320.1113; found: 1320.1122 (Δ = 0.67 ppm).

*Fluorophore **2aL***

4-Bromo-*N*,*N*-dimethylaniline **30** (30 mg; 0.15 mmol) and boronic acid pinacol ester **27a** (67 mg; 0.165 mmol) were dissolved in the mixture of THF and H_2O (4:1 mL). Argon was bubbled through the solution for 15 min, whereupon $PdCl_2(PPh_3)_2$ (2.1 mg; 0.02 eq.) and K_2CO_3 (31 mg; 0.225 mmol; 1.5 eq.) were added. The reaction mixture was stirred under argon atmosphere at 60 °C for 2 hours. The reaction mixture was cooled to room temperature, poured into aq. NH_4Cl (50 mL) and extracted with EtOAc (3×25 mL). The combined organic extracts were dried (Na_2SO_4) and the solvents were evaporated in vacuo. The crude product was purified by column chromatography (SiO_2; CH_2Cl_2/hexane 1:1). Pre-purified product was finally precipitated from a CH_2Cl_2/hexane mixture. Yield: 37 mg (62 %); orange solid; R_f = 0.4 (SiO_2, CH_2Cl_2/hex 1:2); mp 231–233 °C. ^{1}H NMR (500 MHz, $CDCl_3$, 25 °C): δ = 7.57 (d, J = 8.3 Hz, 2H, CH_{Ph}), 7.52 (d, J = 8.3 Hz, 2H, CH_{Ph}), 7.36 (d, J = 8.7 Hz, 2H, CH_{Ph}), 7.24 (d, J = 16.0 Hz, 1H, CH), 7.00–6.96 (m, 2H, CH+CH_{Th}), 6.88–6.83 (m, 3H, 2×CH+CH_{Th}), 6.69 (d, J = 8.7 Hz, 2H, CH_{Ph}), 2.98 ppm (s, 6H, 2×CH_3). ^{13}C NMR (125 MHz, $CDCl_3$, 25 °C): δ = 150.44, 144.40, 140.85, 139.82, 129.83, 129.09 (q, $^2J_{CF}$ = 32.5 Hz), 128.65, 127.82, 126.41, 126.03, 125.85 (q, $^3J_{CF}$ = 3.8 Hz), 125.20, 124.63, 124.45 (q, $^1J_{CF}$ = 271.3 Hz), 117.62, 112.57, 40.61 ppm. ^{19}F NMR (470 MHz, 25 °C, $CDCl_3$): δ = −60.76 ppm.

2. Solubility and characterization of target fluorophores

Despite their high molecular weight, the extended π-system, and the planarity of the branches, it should be highlighted that prepared fluorophores **PM1–3** and **1–4** are extraordinarily soluble in common organic aprotic and less polar solvents. With increasing number of fluorine atoms, the solubility in non-polar solvents is apparently enhanced, for instance fluorophores carrying additional CF_3 groups or the SF_5-substituent ones (**2b–c**, **2d**) are soluble even in alkanes. The solubility of **2e** with six SF_5 groups in *meta* positions is rather inferior, which is consistent with our previous observation.[16] On the other hand, the solubility of whole series in protic and polar solvents is rather suppressed. The solubility of the linear analogue **2aL** is significantly reduced most likely due to a pronounced π-π stacking of the completely planar π-system.

The structure and purity of target fluorophores as well as all intermediates were verified by available analytical techniques such as ^{1}H, ^{13}C, and ^{19}F NMR spectroscopy and high resolution MALDI mass spectrometry. The amorphous nature of the most target fluorophores and abovementioned high solubility probably caused the failure to prepare a monocrystal suitable for X-ray analysis (except linear analogue **2aL**). Hence, the configurations of the double bonds were determined through a coupling constant in ^{1}H NMR spectroscopy and based on the results of X-ray analysis linear **2aL** (Fig. S1). The observed coupling constants range between ~16 Hz for the outer and ~18 Hz for the inner double bond, indicating only *E*-isomer.

NMR analysis of all fluorophores **PM1–3** and **1–4** afforded one desired pattern of signals in ^{1}H, ^{13}C and ^{19}F NMR spectra, however the NMR spectra of fluorophore **2c** showed two distinct sets of signals in the following spectra (Figures S107–S109), nevertheless the integration sum of all signals in ^{1}H NMR spectra (Fig. S107) corresponds to the number of hydrogen atoms in **2c** (36 H). This suggest that the variety of intramolecular rotamers within **2c** in the solution probably occurs due to the presence of the bulky CF_3-substituents in the *ortho*-positions. The number, splitting, and chemical shifts of the signals were clearly affected by the choice of the deuterated solvent (Figure S110), whereas only minor differences in signal patterns were observed when changing the temperature. In addition, the ratio of rotamers in $CDCl_3$ further depended on the light irradiation. The NMR spectra of **2c** prepared in the dark and that irradiated with Royal Blue LED for 1 h, are different (Figure S111) but prolonged irradiation caused a gradual degradation of **2c**. An analogical interaction between hydrogen atoms of the ethylene π-bridge and fluorine atoms of the *ortho*-positioned CF_3-groups[17] was also observed for the corresponding precursors **17c**, **22c**, and **27c**, where particular double bond signal was always significantly broadened in ^{1}H NMR spectra (Figures S83–S85). The published NMR studies of trifluoromethyl labeled stilbenes or retinal analogs support these deductions.[18–20] However, the structure and purity of fluorophore **2c** were further confirmed by HR-MALDI-MS, TLC, and preparative HPLC.

3. X-Ray analysis

A slow crystallization of linear fluorophore **2aL** from $CDCl_3$/hexane mixture afforded its single crystal suitable for XRD analysis; The molecular representation of **2aL** confirms its expected structure as well as the exclusive geometrical *E*-configuration and *s*-trans conformation of both double bonds within 2,5-divinylthiophene π-linker. The side view provided in Figure S1 reveals a slightly bent or bowl-shape molecular backbone of **2aL** in the solid state.

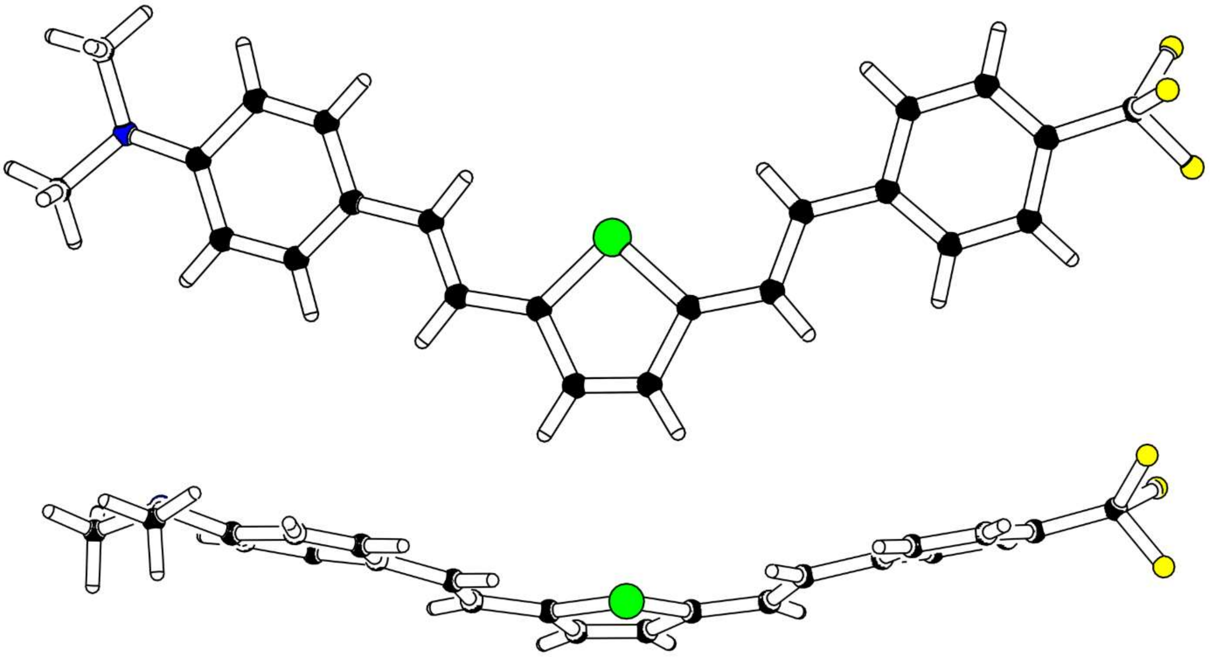

Figure S1. The molecular representations of **2aL** (front and side view). Disorder of $-NMe_2$ and $-CF_3$ groups has been omitted for clarity. Atom colors: carbon (black), hydrogen (white), nitrogen (blue), fluorine (yellow), sulfur (green).

Diffraction data (Table S1) for compound **2aL** (Fig. S1) were collected using a Bruker Venture D8 diffractometer at 150 K with graphite-monochromated Mo-Kα (0.7107 Å) radiation. The frames were integrated with the Bruker SAINT software package using a narrow frame algorithm. Data were corrected for absorption effects using the Multi-Scan method (SADABS). Obtained data were treated by XT-version 2014/5 and SHELXL-2019/1 software implemented in APEX4 v2021.10-0 (Bruker AXS) system. All non-hydrogen atoms were refined using anisotropic displacement parameters. Crystallographic data for the structural analyses have been deposited with the Cambridge Crystallographic Data Centre, CCDC nos. 2502796. Copies of this information may be obtained free of charge from The Director, CCDC, 12 Union Road, Cambridge CB2 1EZ, UK (Fax: +44-1223-336033; e-mail: deposit@ccdc.cam.ac.uk or http://www.ccdc.cam.ac.uk). The structure of **2aL** contains static disorder of $-NMe_2$ and $-CF_3$ groups, this disorder was treated with SHELXL software.[21]

Computer programs: Bruker Instrument Service vV6.2.3, APEX4 v2022.10-0 (Bruker AXS), SAINT V8.37A (Bruker AXS Inc., 2015), XT, VERSION 2014/5, SHELXL2019/1 (Sheldrick, 2019), PLATON (Spek, 2009).

Table S1. Experimental details of the X-Ray analysis.

Crystal data	
Chemical formula	$C_{23}H_{20}F_3NS$
M_r	399.46
Crystal system, space group	Monoclinic, *Pc*
Temperature (K)	150
a, *b*, *c* (Å)	21.849(10), 7.486(4), 5.924(3)
β (°)	97.75(2)
V (Å^3)	960.1(8)
Z	2
Radiation type	Mo *K*α
μ (mm^{-1})	0.21
Crystal size (mm)	0.57 × 0.09 × 0.02
**Data collection**	
Diffractometer	Bruker D8 - Venture
Absorption correction	Multi-scan<br>*SADABS2016*/2 - Bruker AXS area detector scaling and absorption correction
T_{min}, T_{max}	0.464, 0.745
No. of measured, independent and observed [$I > 2\sigma(I)$] reflections	17647, 2924, 1716
R_{int}	0.249
θ_{max} (°)	23.8
$(\sin \theta/\lambda)_{max}$ (Å^{-1})	0.567
**Refinement**	
$R[F^2 > 2\sigma(F^2)]$, $wR(F^2)$, S	0.152, 0.348, 1.23
No. of reflections	2924
No. of parameters	287
No. of restraints	288
H-atom treatment	H-atom parameters constrained
$\Delta\rho_{max}$, $\Delta\rho_{min}$ (e Å^{-3})	1.35, -0.56
Absolute structure	Refined as an inversion twin.
Absolute structure parameter	0.4(5)

4. Thermal properties

Thermal properties of fluorophores **PM1–PM3** and **1–4** were investigated by differential scanning calorimetry DSC. The DSC curves were recorded with a scan rate of 5 °C/min within the range 25–500 °C. The temperatures of decomposition (T_d) and glass transition (T_g) were determined as intersection of the baseline and the tangent of the peak/step (onset point). To properly evaluate a thermal effect, the heating-cooling-re-heating cycles were measured at a scan rate of 5–10 °C/min within the appropriate temperature window. Thermal decomposition was detected as a gradual or distinctive exothermic process going above the baseline.

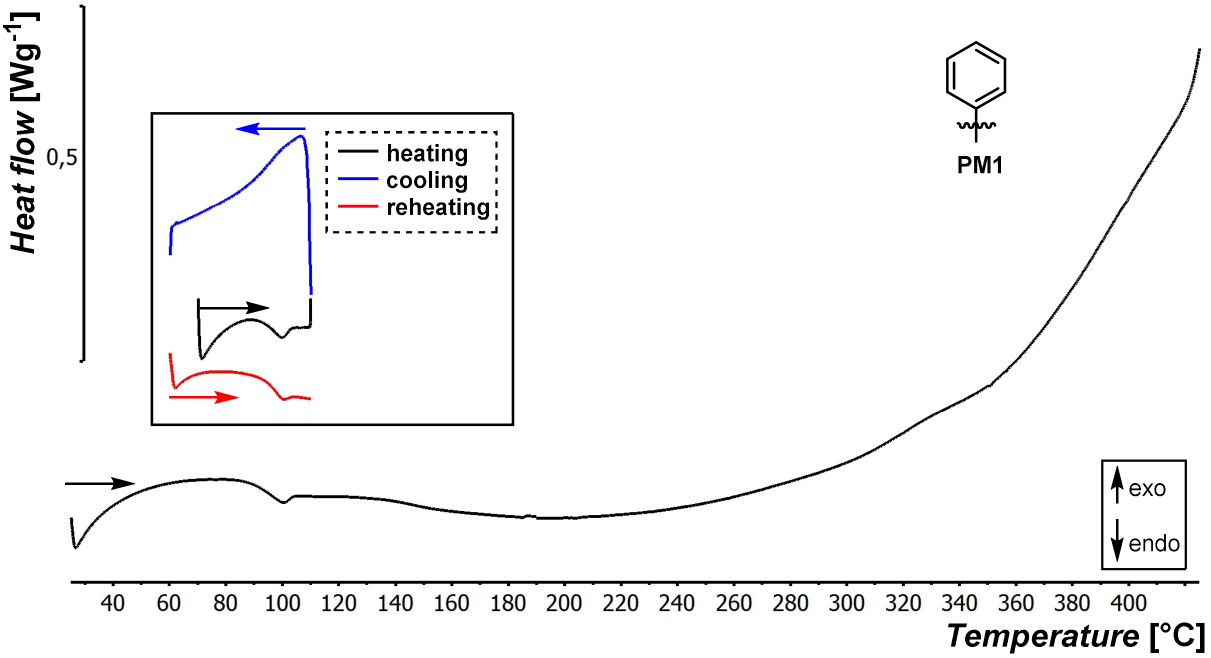

Figure S2. DSC thermogram of parent fluorophore **PM1** measured with a scan rate of 5 °C/min within the range 25–420 °C.

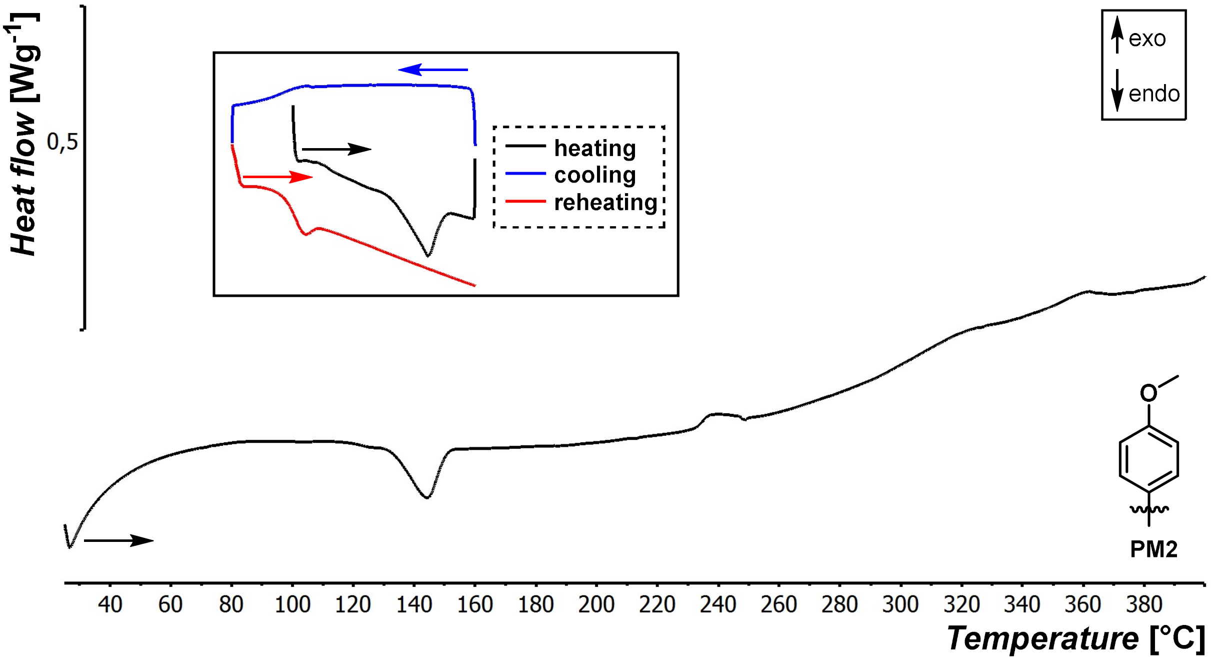

Figure S3. DSC thermogram of parent fluorophore **PM2** measured with a scan rate of 5 °C/min within the range 25–400 °C.

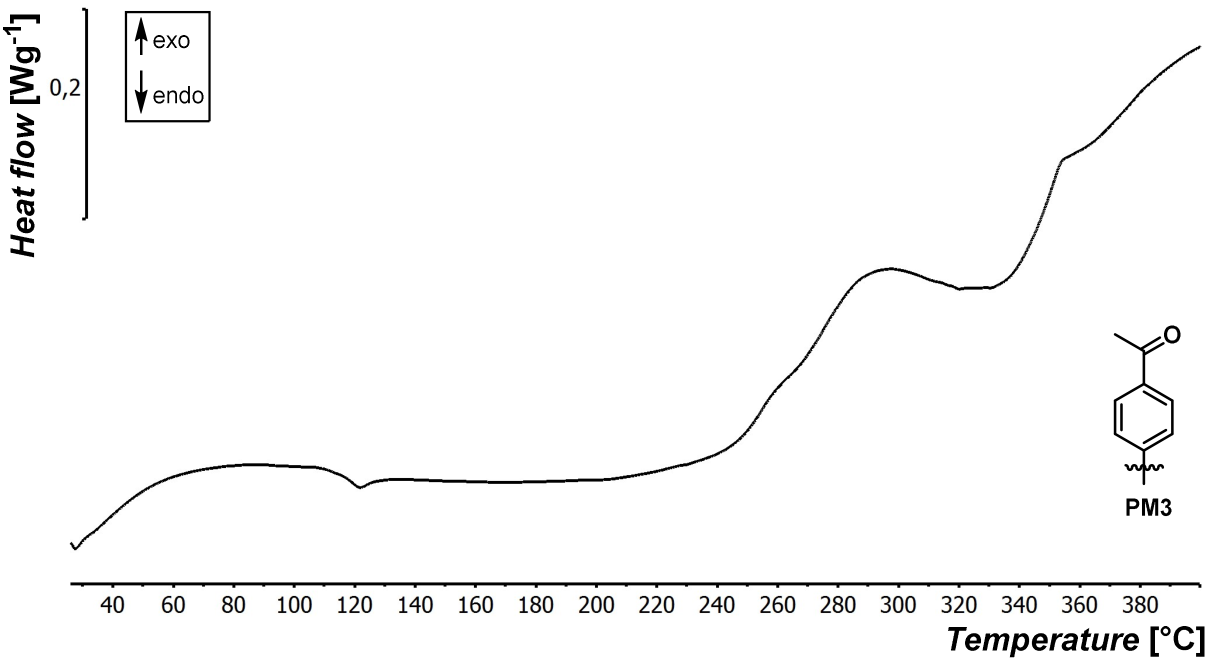

Figure S4. DSC thermogram of parent fluorophore **PM3** measured with a scan rate of 5 °C/min within the range 25–400 °C.

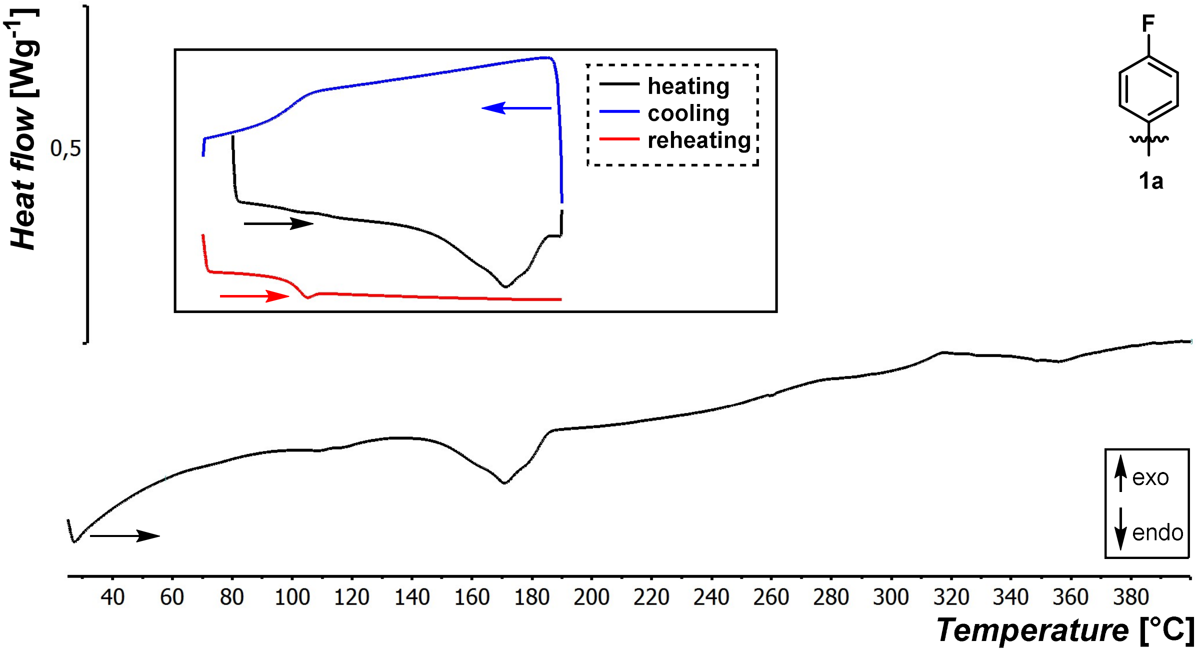

Figure S5. DSC thermogram of fluorophore **1a** measured with a scan rate of 5 °C/min within the range 25–400 °C.

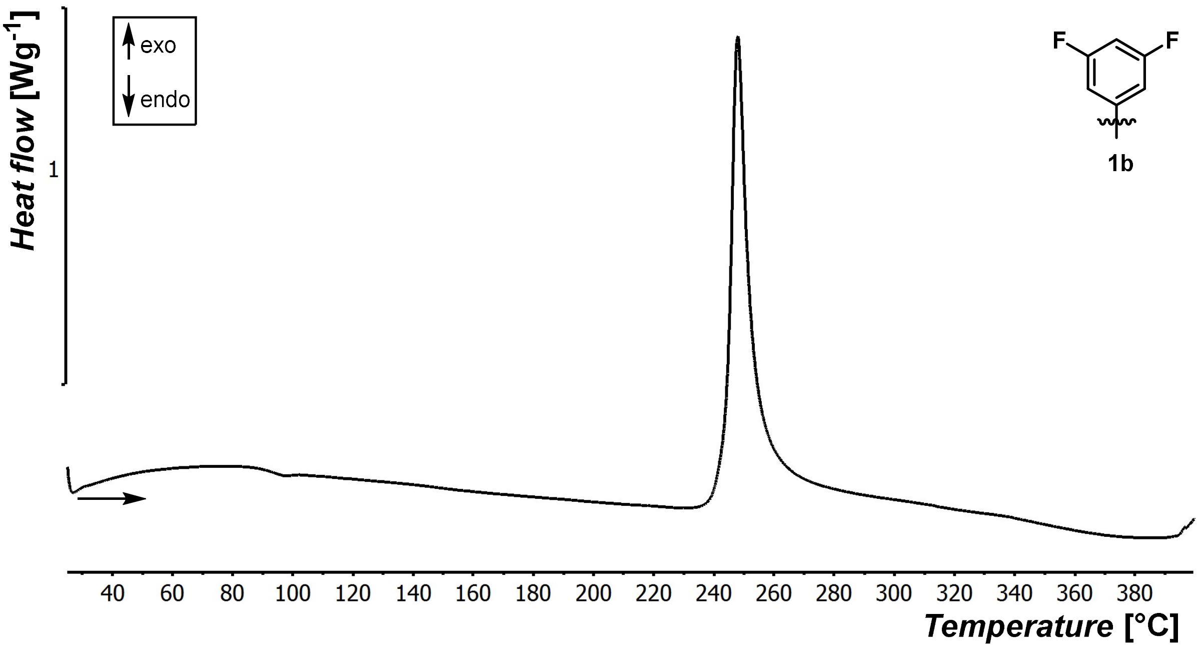

Figure S6. DSC thermogram of fluorophore **1b** measured with a scan rate of 5 °C/min within the range 25–400 °C.

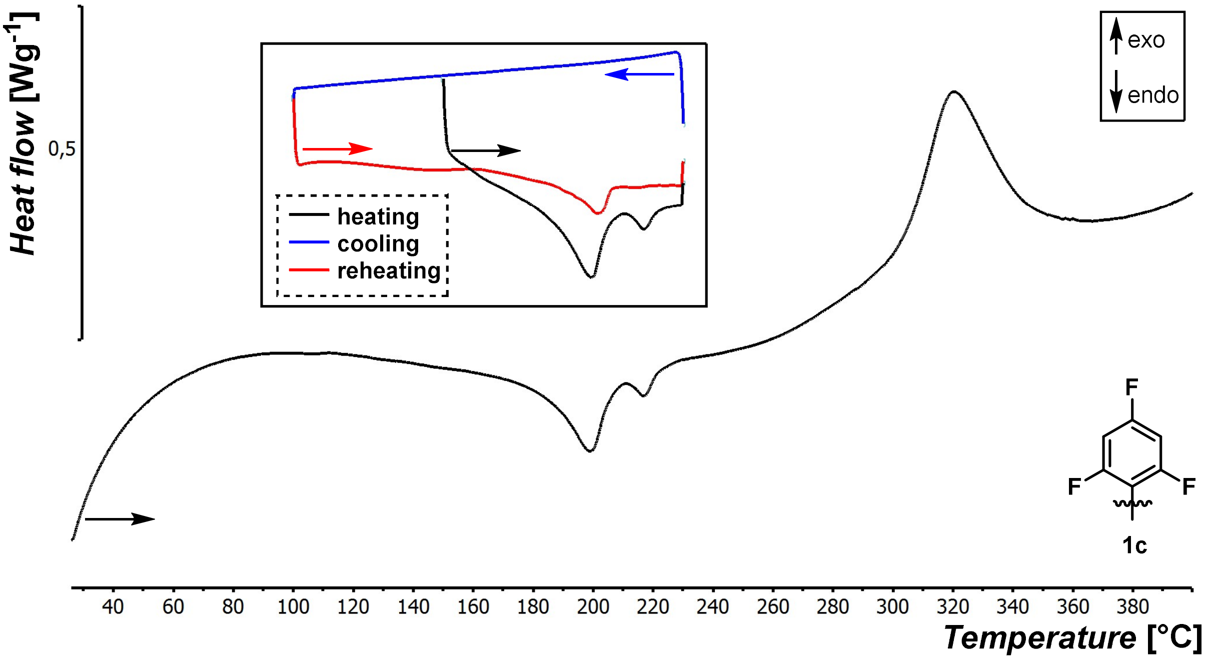

Figure S7. DSC thermogram of fluorophore **1c** measured with a scan rate of 5 °C/min within the range 25–400 °C.

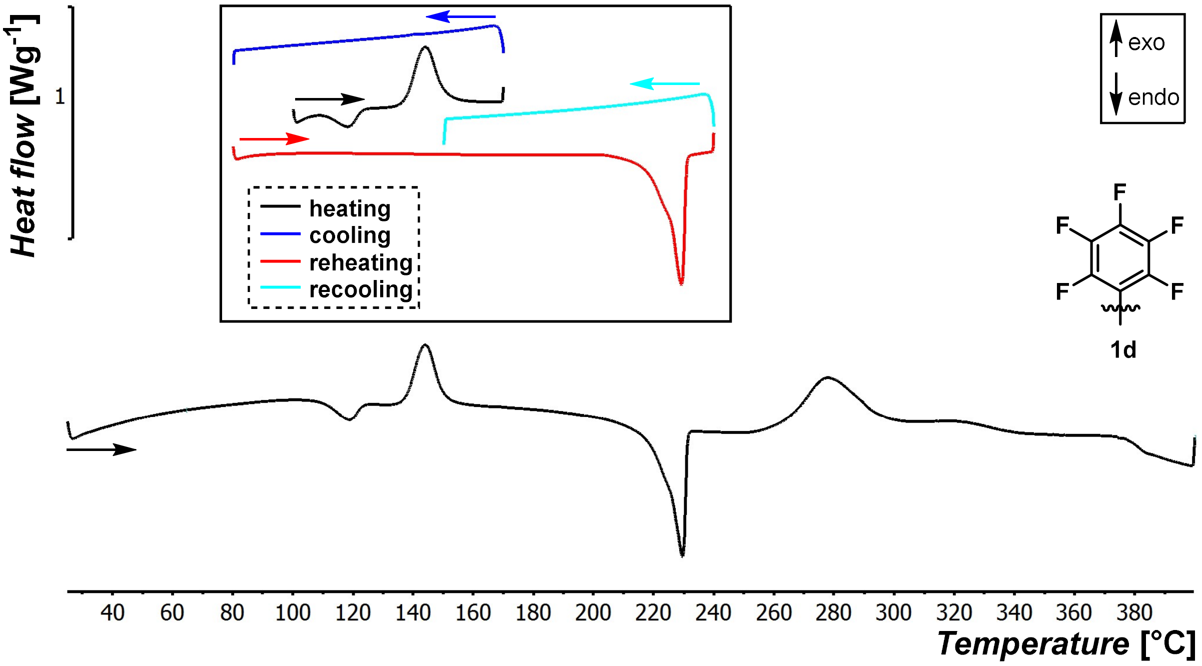

Figure S8. DSC thermogram of fluorophore **1d** measured with a scan rate of 5 °C/min within the range 25–400 °C.

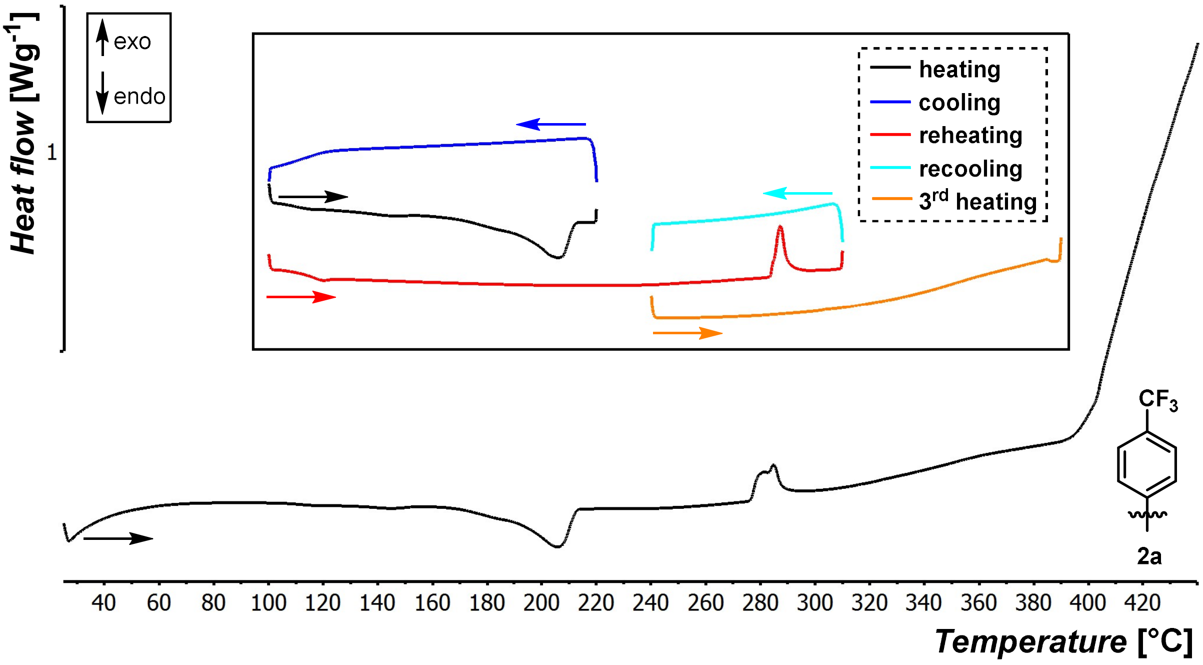

Figure S9. DSC thermogram of fluorophore **2a** measured with a scan rate of 5 °C/min within the range 25–440 °C.

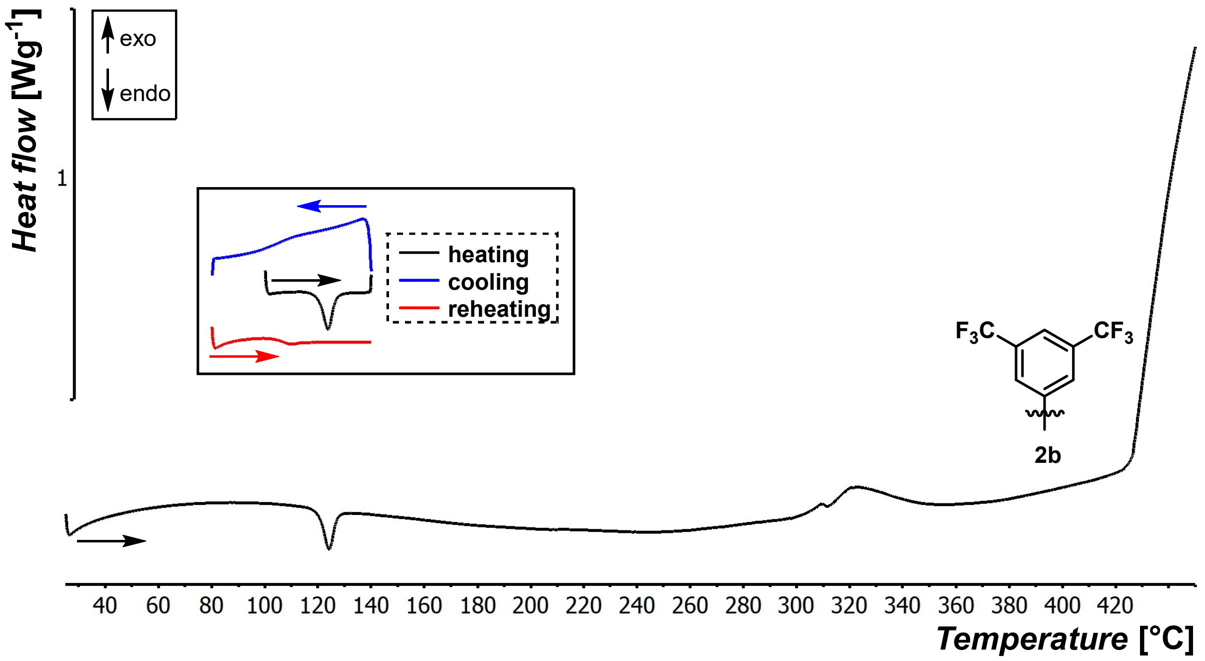

Figure S10. DSC thermogram of fluorophore **2b** measured with a scan rate of 5 °C/min within the range 25–450 °C.

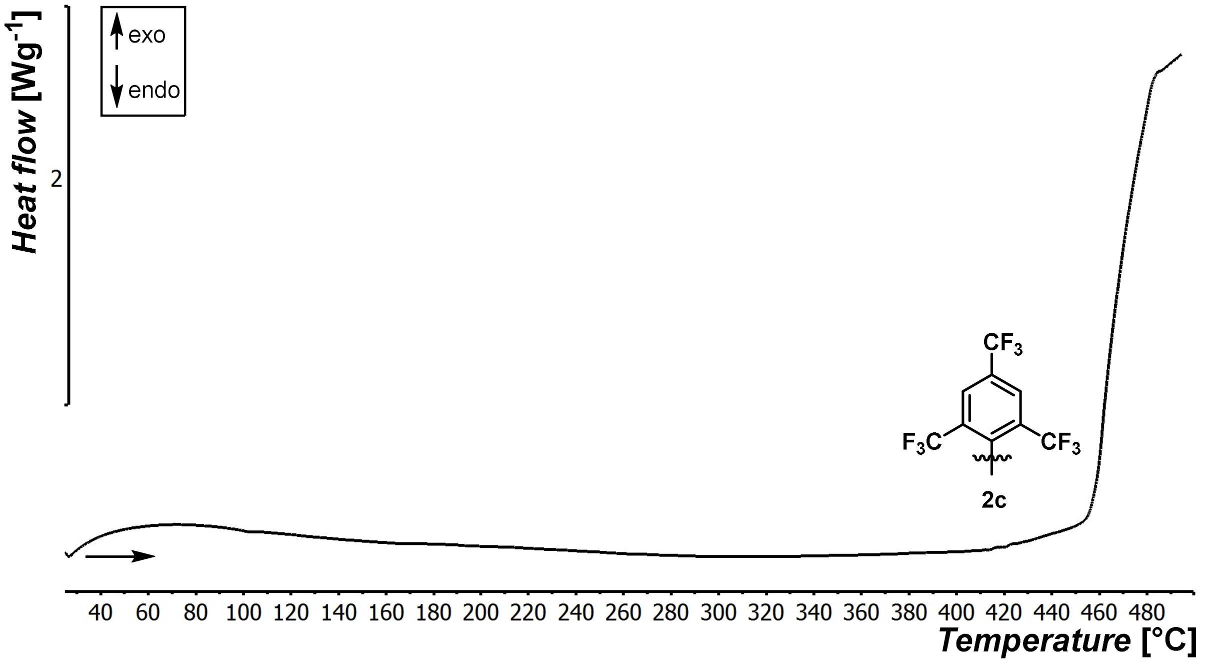

Figure S11. DSC thermogram of fluorophore **2c** measured with a scan rate of 5 °C/min within the range 25–500 °C.

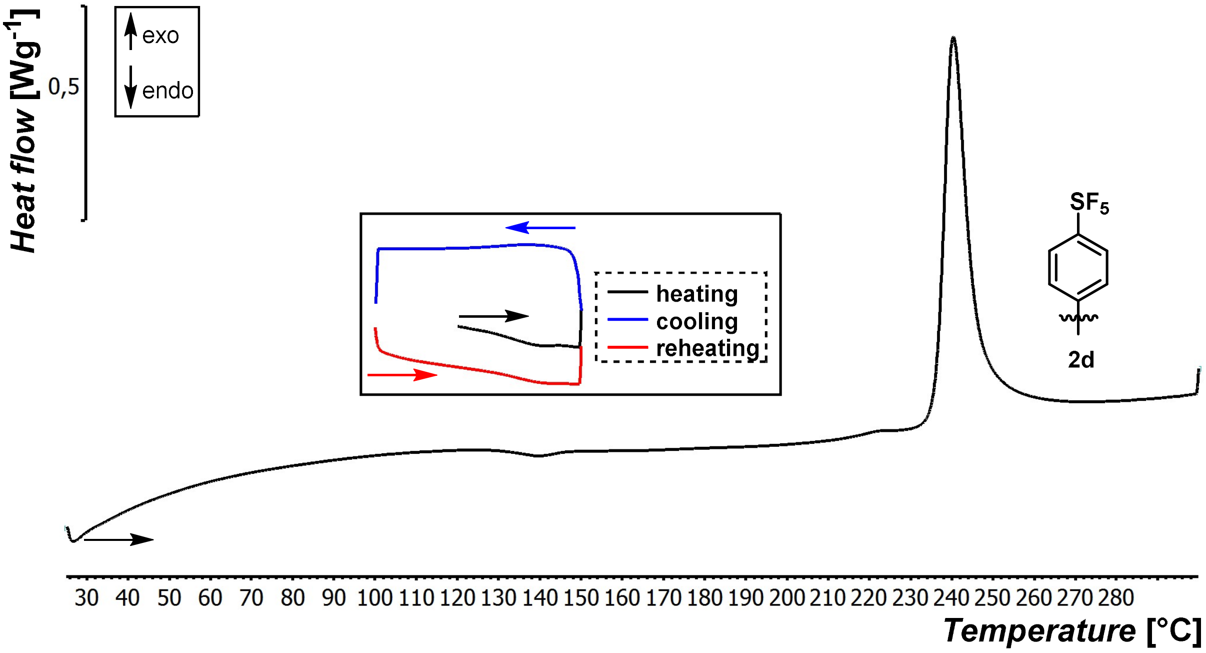

Figure S12. DSC thermogram of fluorophore **2d** measured with a scan rate of 5 °C/min within the range 25–300 °C.

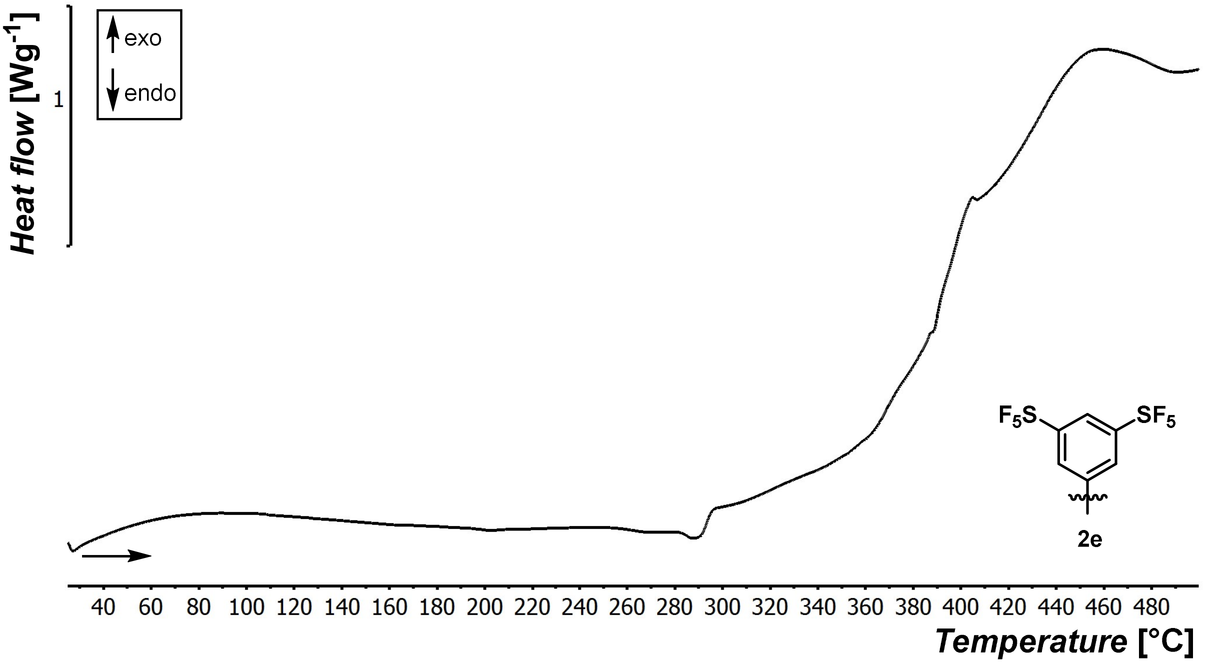

Figure S13. DSC thermogram of fluorophore **2e** measured with a scan rate of 5 °C/min within the range 25–500 °C.

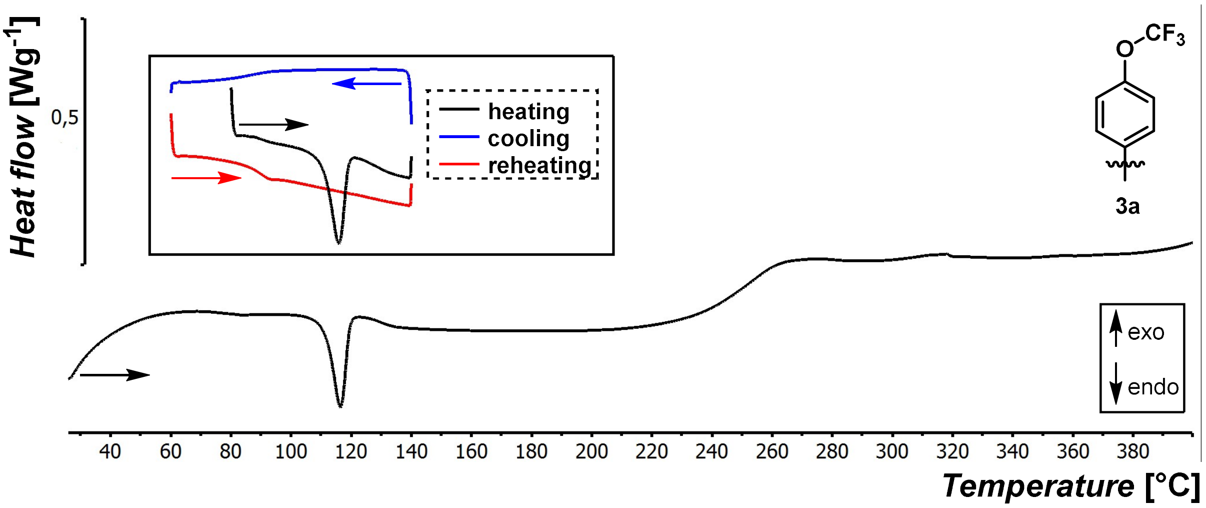

Figure S14. DSC thermogram of fluorophore **3a** measured with a scan rate of 5 °C/min within the range 25–400 °C.

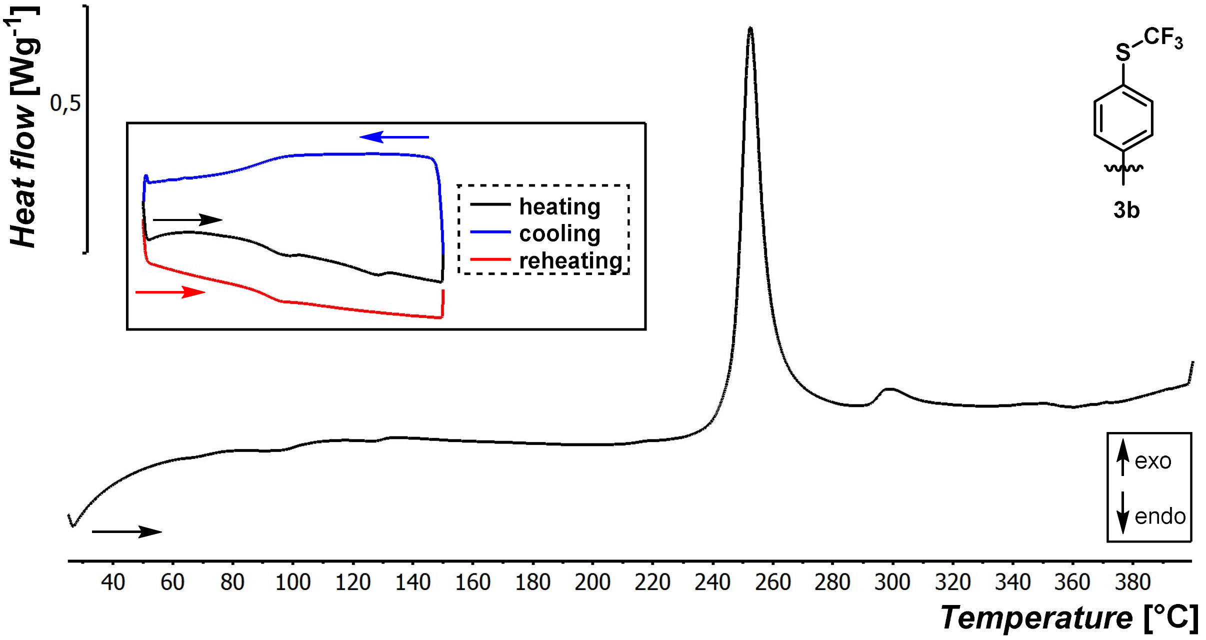

Figure S15. DSC thermogram of fluorophore **3b** measured with a scan rate of 5 °C/min within the range 25–400 °C.

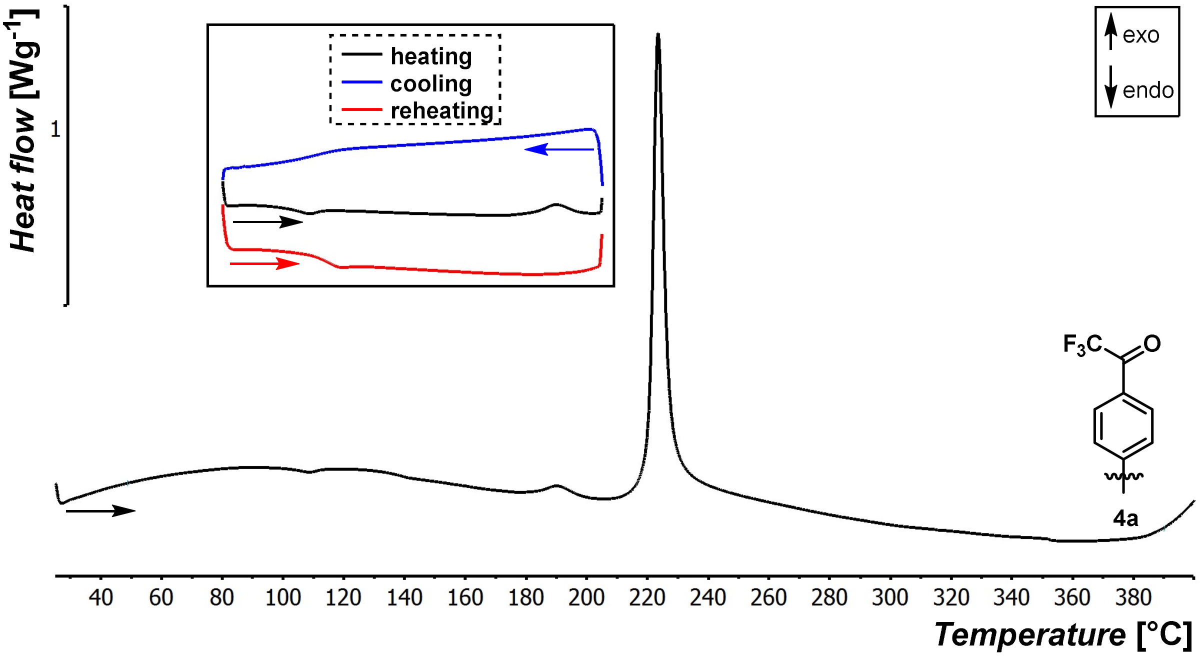

Figure S16. DSC thermogram of fluorophore **4a** measured with a scan rate of 5 °C/min within the range 25–400 °C.

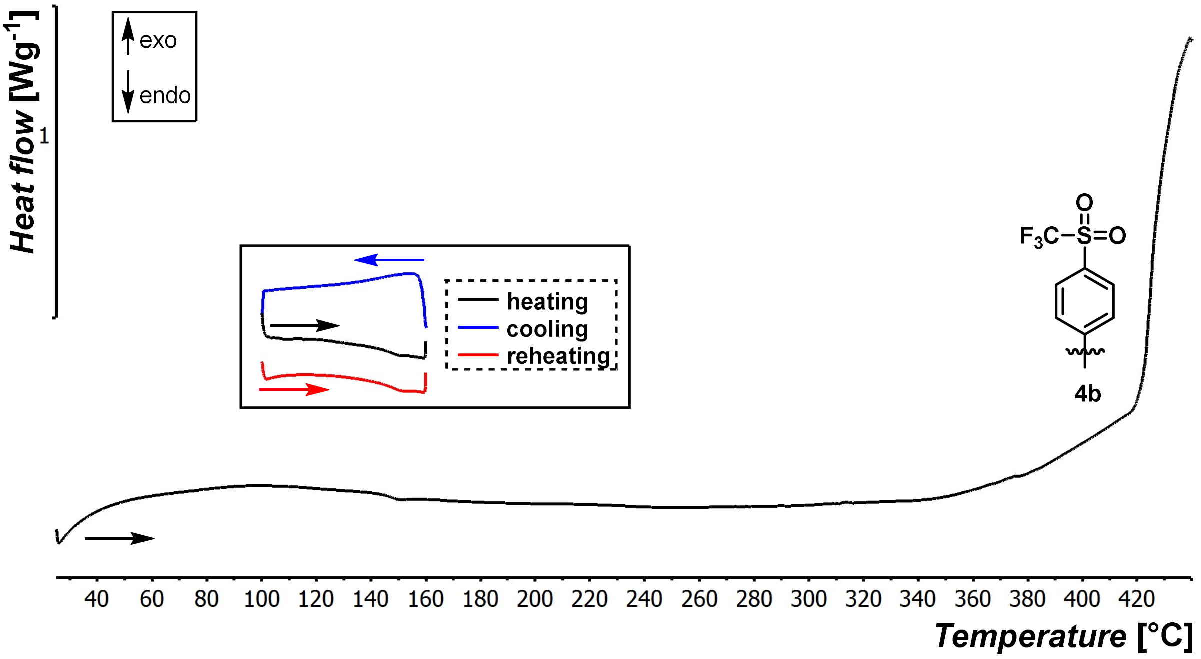

Figure S17. DSC thermogram of fluorophore **4b** measured with a scan rate of 5 °C/min within the range 25–440 °C.

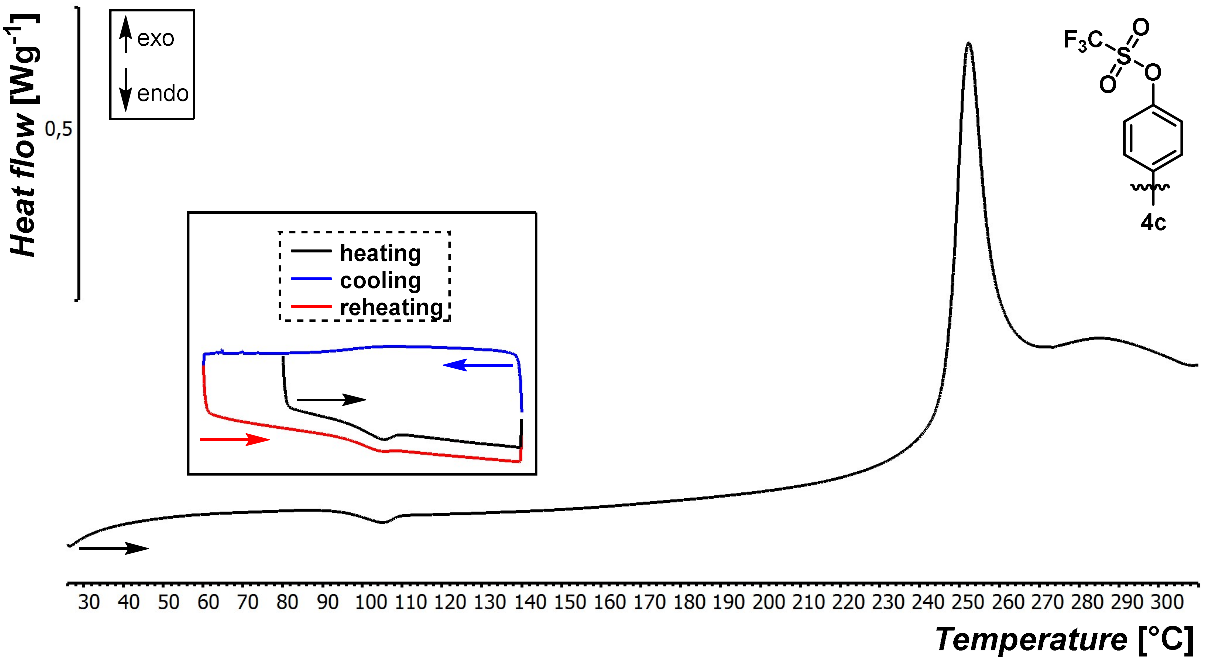

Figure S18. DSC thermogram of fluorophore **4c** measured with a scan rate of 5 °C/min within the range 25–310 °C.

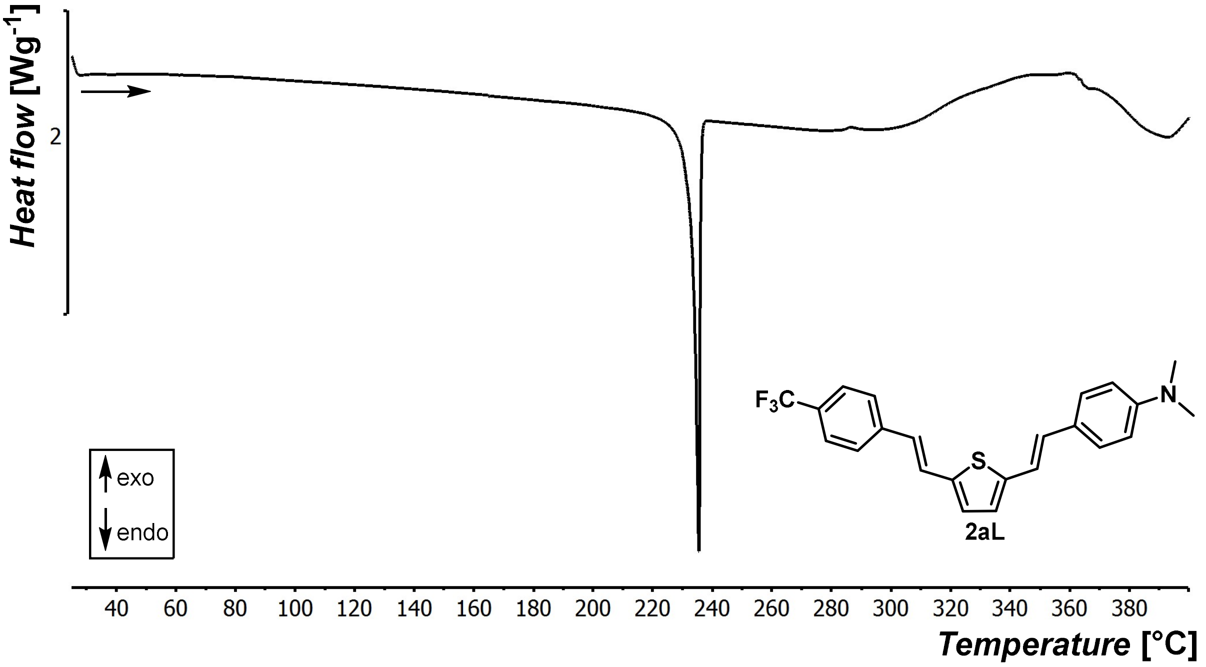

Figure S19. DSC thermogram of fluorophore **2aL** measured with a scan rate of 5 °C/min within the range 25–400 °C.

5. Electrochemistry

The electrochemical behavior of target fluorophores **PM1–3** and **1–4** was investigated in THF containing 0.1 M Bu_4NPF_6 in a three-electrode cell by cyclic voltammetry (CV). The working electrode was glassy carbon disk (1 mm in diameter). As the reference and auxiliary electrodes were used leakless Ag/AgCl electrode (SSCE) containing filling electrolyte (3.4 M KCl; +0.241 V vs. SHE) and titanium rod with a thick coating of platinum, respectively. All potential values were obtained from the first cycle at scan rate 100 $mV \cdot s^{-1}$ and are given *vs.* SSCE. The first oxidation a reduction processes were examined at scan rates ranging from 20 to 200 $mV \cdot s^{-1}$. Voltammetric measurements were performed by using an integrated potentiostat system ER466 (eDAQ Europe) operated with EChem Electrochemistry software. Although the measured solution was bubbled with argon before the voltammetric analysis, the residual peak of oxygen reversible reduction was recorded around −0.9 V.

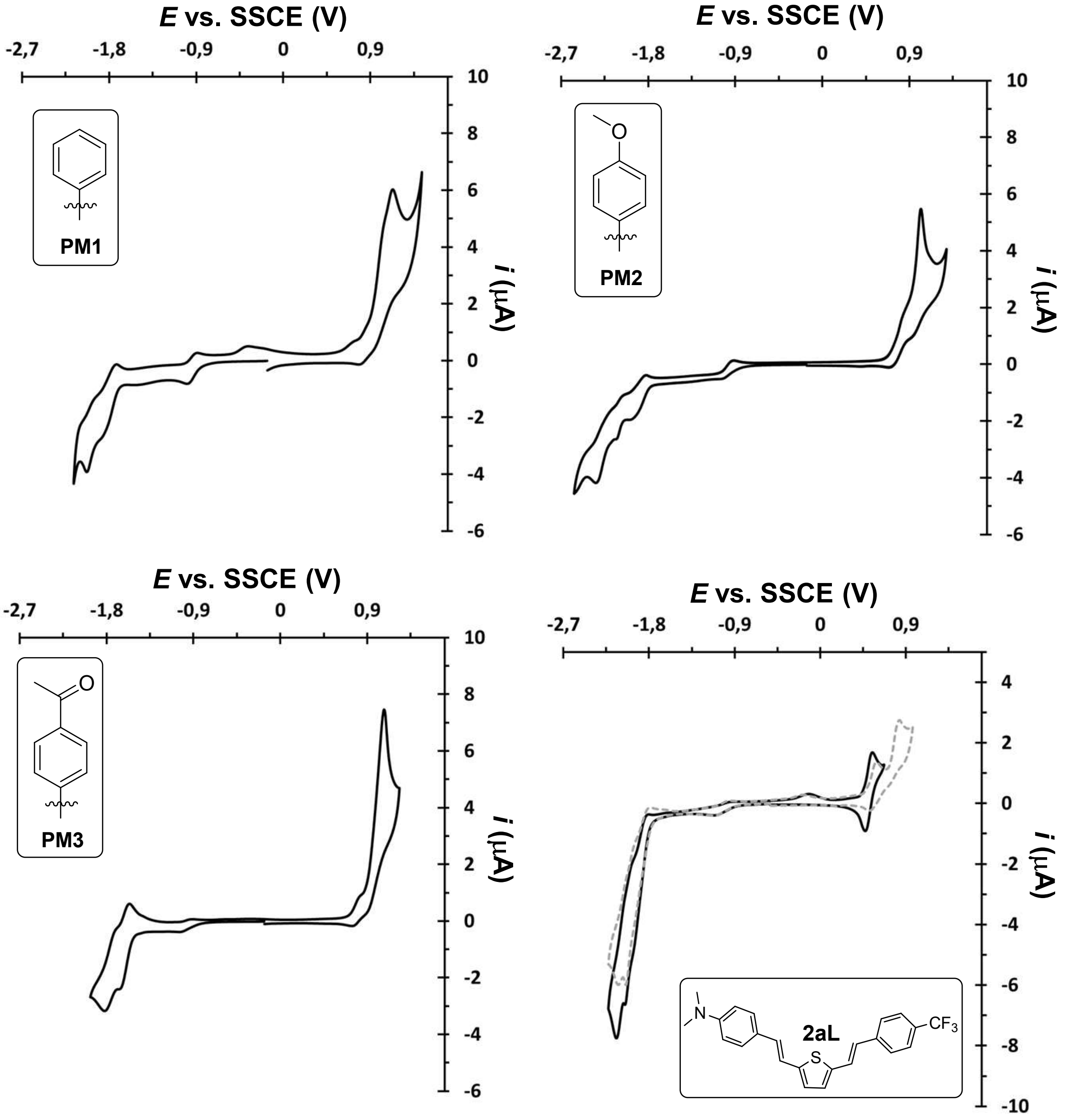

Figure S20. Cyclic voltammograms of parent fluorophores **PM1–PM3** and **2aL** measured in THF containing 0.1 M Bu_4NPF_6 at glassy carbon electrode; v = 100 mVs^{-1}.

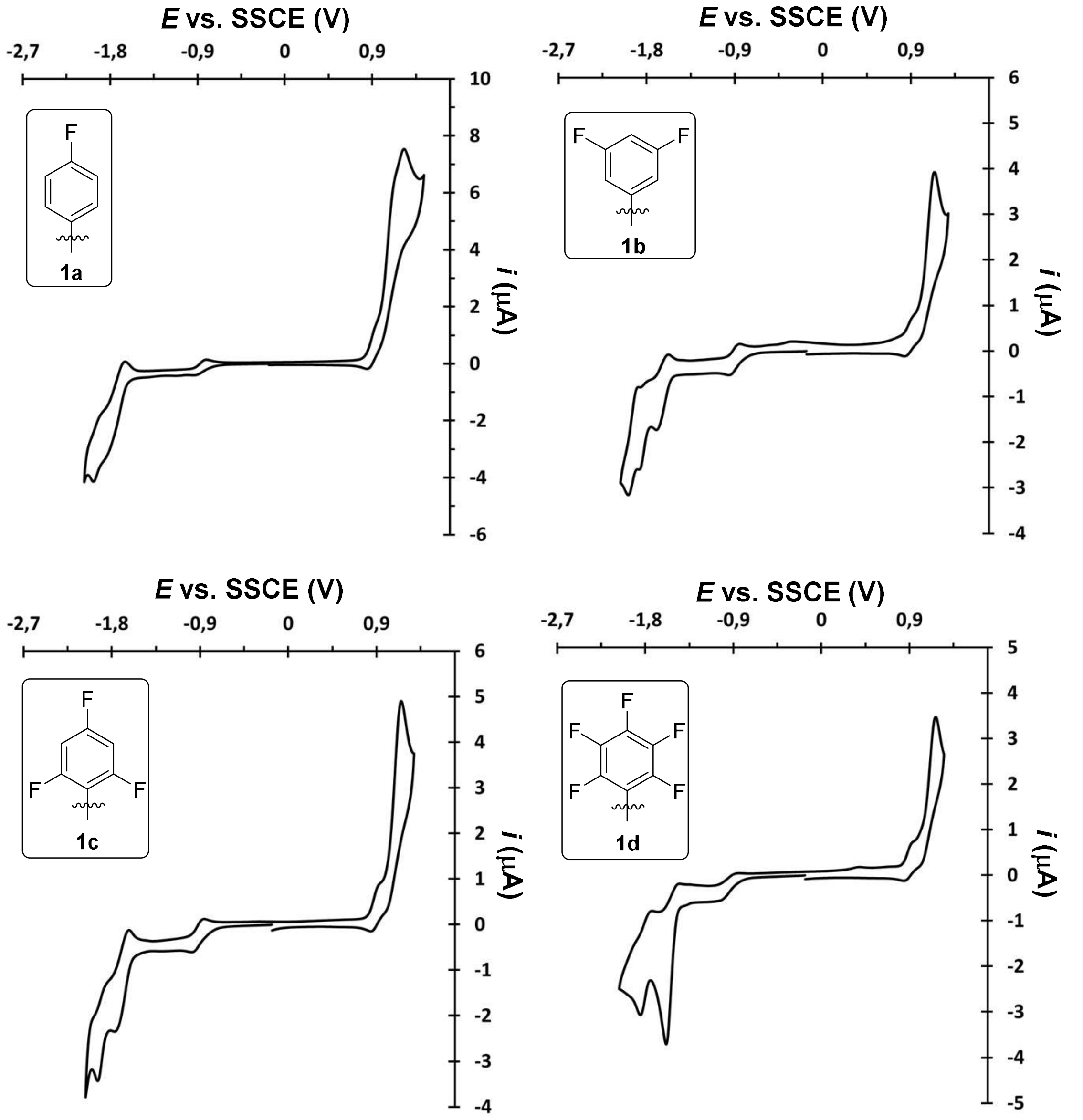

Figure S21. Cyclic voltammograms of fluorophores **1a–1d** measured in THF containing 0.1 M Bu_4NPF_6 at glassy carbon electrode; v = 100 mVs^{-1}.

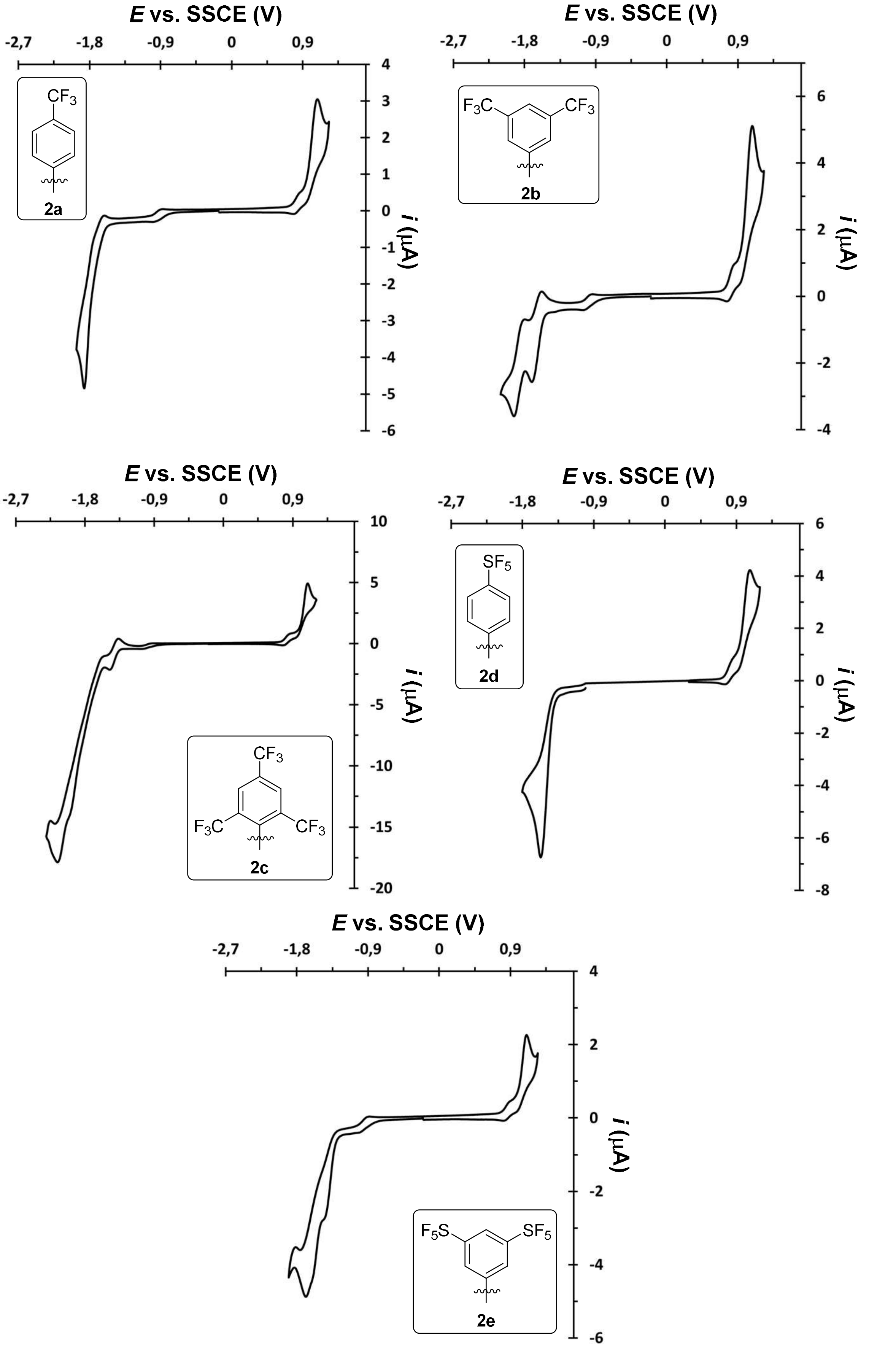

Figure S22. Cyclic voltammograms of fluorophores **2a**–**e** measured in THF containing 0.1 M Bu_4NPF_6 at glassy carbon electrode; v = 100 mVs^{-1}.

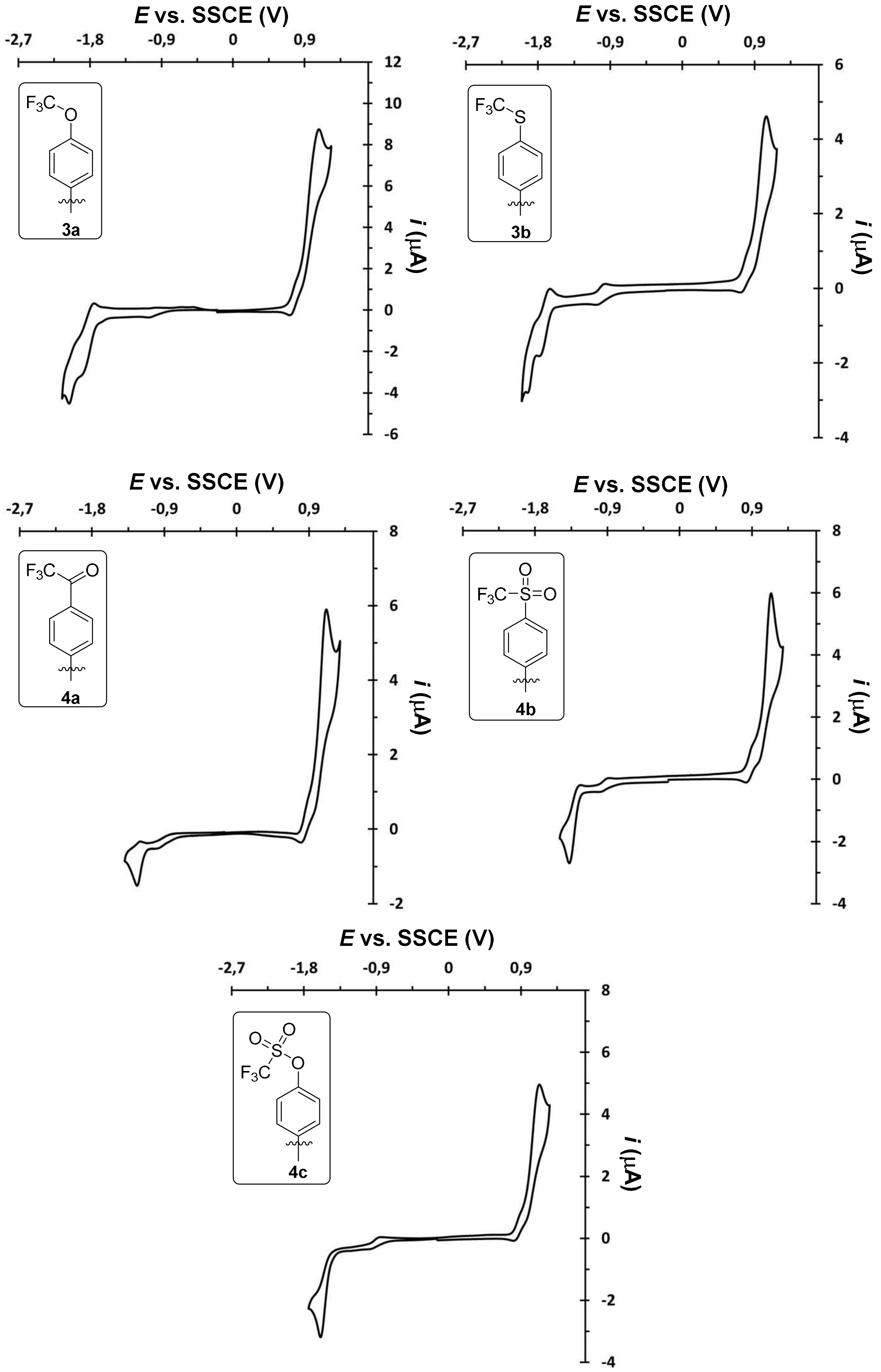

Figure S23. Cyclic voltammograms of fluorophores **3a**–**b** and **4a**–**c** measured in THF containing 0.1 M Bu_4NPF_6 at glassy carbon electrode; v = 100 mVs^{-1}.

A comparison of particular voltammograms for the tripodal fluorophore **2a** and the linear analogue **2aL** revealed several differences. The oxidation of the tripodal **2a** was recorded as a multi-electron process involving one-electron reversible oxidation of amino donor followed immediately by irreversible oxidations of the thiophene-based π-linker in each branch. Since there is only one branch in the linear molecule **2aL**, the first reversible single-electron oxidation of the amino donor is followed by only one-electron irreversible oxidation of the π-system (see Fig. S20). This oxidation feature is fully consistent with the Me_2N-(π-Th-π-FluoroA) and TPA-(π-Th-π-FluoroA)$_3$ linear/tripodal architecture. On the other hand, multi-electron reductions were determined in the case of both linear and tripodal fluorophores **2aL** and **2a**, where **2aL** showed gradually developed irreversible reductions with distinctive shoulder [$E_{p(red1)}$]. The multi-electron transfer is related to the number of branches as well as the number of appended fluorine atoms, respectively. The values $E_{HOMO/LUMO}^{CV}$ = –5.15/–2.64 eV with the energy gap 2.51 eV were revealed for tripodal fluorophore **2a**. These values are clearly elevated in the case of the linear molecule **2aL** ($E_{HOMO/LUMO}^{CV}$ = –4.82/–2.35 eV) with comparable energy gap of 2.47 eV. This behavior is a typical feature of linear and tripodal analogues.[16,22]

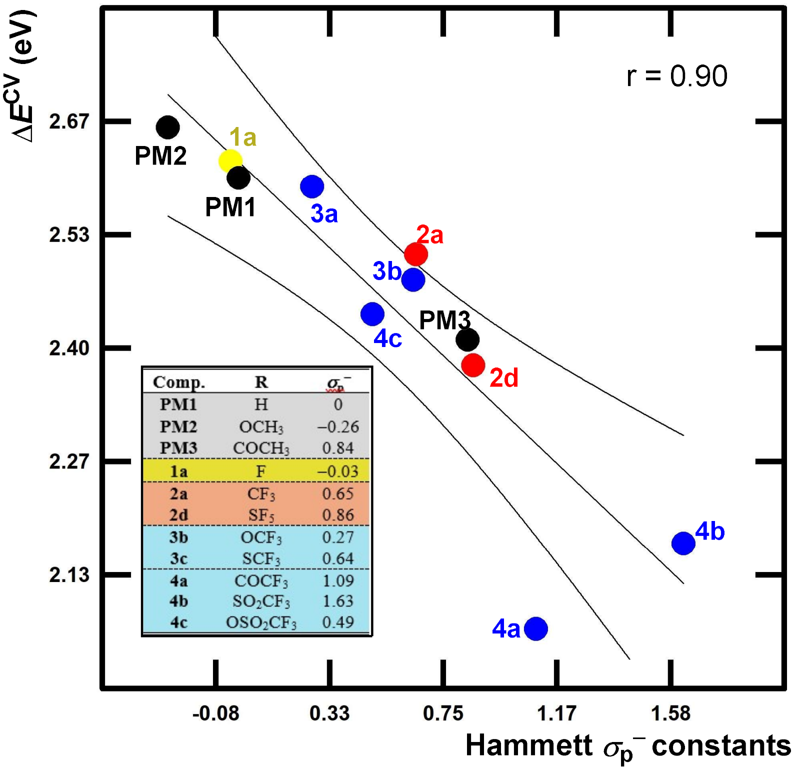

Figure S24. Correlation of the electrochemical gap ΔE^{CV} (recorded in THF) and the Hammett σ_p^- constants.[23]

6. Linear optical properties

Table S2: Linear and nonlinear optical data of fluorophores **PM1–3** and **1–4** in toluene.

	Comp.	λ_{max}^{A} [nm/eV][a]	ε_{max}^{A} [10^3 $M^{-1}cm^{-1}$][a]	λ_{max}^{E} [nm/eV][a]	Φ^{F} [-][b]	Stokes shift [cm^{-1}/eV]	$\delta_{2PA}/\lambda_{2PA}$ [GM/nm][d]
Parent series	**PM1**	437/2.84	120	501/2.48	0.45	2920/0.36	845/730
	**PM2**	440/2.82	131	499/2.49	0.49	2690/0.33	930/730
	**PM3**	453/2.74	107	526/2.36	0.50	3060/0.38	540/730
Fluoro-substituted	**1a**	445/2.79	107	500/2.48	0.48	2470/0.31	1380/750
	**1b**	451/2.75	120	511/2.43	0.44	2600/0.32	1150/730
	**1c**	448/2.77	120	506/2.45	0.45	2560/0.32	910/750
	**1d**	457/2.71	137	518/2.39	0.48	2580/0.32	815/740
CF_3- and SF_5-substituted	**2a**	452/2.74	140	513/2.42	0.47	2630/0.33	1020/740
	**2aL**	433/2.86	60.1	532/2.33	[c]	4300/0.53	[c]
	**2b**	457/2.71	115	519/2.39	0.45	2610/0.32	1260/740
	**2c**	436/2.84	94.3	594/2.09	0.14	6100/0.76	[e]
	**2d**	455/2.73	120	518/2.39	0.47	2670/0.33	1125/740
	**2e**	462/2.68	124	532/2.33	0.54	2850/0.35	1175/750
CF_3-funcional. D/A	**3a**	447/2.77	112	504/2.46	0.47	2530/0.31	1145/750
	**3b**	445/2.79	131	515/2.41	0.45	3050/0.38	960/750
	**4a**	479/2.59	109	573/2.16	0.53	3420/0.42	605/750
	**4b**	473/2.62	116	554/2.24	0.54	3090/0.38	520/740
	**4c**	441/2.81	92.5	509/2.44	0.44	3029/0.38	605/740

[a] Measured in toluene (Dimroth-Reichardt polarity parameter E_T^N = 0.099[24]) at concentration ≈ 5×10^{-6} M; emitted at the absorption maximum wavelength. [b] Fluorescence quantum yield (±10%) determined relative to perylene as a standard (Φ^{F} = 0.94 in cyclohexane).[4] [c] Not measured. [d] Measured in THF at concentration 5×10^{-6} M. [e] Under the detection limit.

Table S3: Linear optical data of fluorophores **PM1–3** and **1–4** in ACN.

	Comp.	λ_{max}^{A} [nm/eV][a]	ε_{max}^{A} [10^3 $M^{-1}cm^{-1}$][a]	λ_{max}^{E} [nm/eV][a]	Stokes shift [cm^{-1}/eV]
Parent series	**PM1**	437/2.84	144	584/2.12	5760/0.71
	**PM2**	440/2.82	113	572/2.17	5240/0.65
	**PM3**	453/2.74	107	660/1.88	6920/0.86
Fluoro-substituted	**1a**	436/2.84	74.8	582/2.13	5750/0.71
	**1b**	444/2.79	108	616/2.01	6290/0.78
	**1c**	439/2.82	47.3	591/2.10	5860/0.73
	**1d**	449/2.76	100	642/1.93	6700/0.83
CF_3- and SF_5-substituted	**2a**	443/2.80	59.6	619/2.00	6420/0.80
	**2aL**	424/2.92	43.5	626/1.98	7610/0.94
	**2b**	446/2.78	175	642/1.93	6850/0.85
	**2c**	432/2.87	178	-	-
	**2d**	452/2.74	138	635/1.95	6380/0.79
	**2e**	450/2.76	105	646/1.92	6742/0.84
CF_3-funcional. D/A	**3a**	440/2.82	119	594/2.09	5890/0.73
	**3b**	445/2.79	62.2	619/2.00	6320/0.78
	**4a**	469/2.64	98.9	-	-
	**4b**	463/2.68	78.1	-	-
	**4c**	441/2.81	92.5	604/2.05	6120/0.76

[a] Measured in acetonitrile (Dimroth-Reichardt polarity parameter E_T^N = 0.460[24]) at concentration ≈ 5×10^{-6} M; emitted at the absorption maximum wavelength.

Table S4: Fitting parameters of the nanosecond dynamics of **PM1–3** and **1–4** in THF measured at the peak of the fluorescence spectra.

	Comp.	λ_{det} [nm]	A_1	τ_1 [ns]	A_2	τ_2 [ns]	A_3	τ_3 [ns]	<τ> [ns]
Parent series	**PM1**	534	-	-	1	1.62	-	-	1.61
	**PM2**	529	-	-	1	1.39	-	-	1.39
	**PM3**	593	-	-	1	1.91	-	-	1.90
Fluoro-substituted	**1a**	536	-	-	1	1.66	-	-	1.65
	**1b**	562	-	-	0.29	1.41	0.71	2.14	1.93
	**1c**	555	-	-	0.89	1.84	0.11	2.71	1.92
	**1d**	588	-	-	-	-	1	2.23	2.23
CF_3- and SF_5-substituted	**2a**	569	-	-	0.20	1.25	0.80	2.08	1.91
	**2b**	586	-	-	0.23	1.43	0.77	2.26	2.06
	**2c**	709	1	0.32	-	-	-	-	0.32
	**2d**	578	-	-	0.22	1.27	0.78	2.13	1.94
	**2e**	612	-	-	0.29	1.41	0.71	2.10	1.90
CF_3-funcional. D/A	**3a**	554	-	-	0.86	1.67	0.14	2.36	1.76
	**3b**	575	-	-	0.20	1.21	0.80	2.04	1.87
	**4a**	689	0.97	0.31	0.03	1.22	-	-	0.34
	**4b**	669	1	0.64	-	-	-	-	0.64
	**4c**	556	-	-	0.53	1.56	0.47	2.15	1.83

Table S5: Fitting parameters of the nanosecond dynamics of **PM1–3** and **1–4** in toluene measured at the peak of the fluorescence spectra.

Series	Comp.	λ_{det} [nm]	A_1	τ_1 [ns]	A_2	τ_2 [ns]	<τ> [ns]
Parent series	**PM1**	502	0.25	0.40	0.75	1.03	0.87
	**PM2**	499	0.26	0.44	0.74	0.98	0.84
	**PM3**	527	0.27	0.47	0.73	1.13	0.95
Fluoro- substituted	**1a**	501	0.27	0.45	0.73	1.05	0.89
	**1b**	513	0.28	0.41	0.72	1.10	0.90
	**1c**	509	0.26	0.42	0.74	1.09	0.91
	**1d**	522	0.65	1.12	0.35	1.46	1.23
CF_3- and SF_5- substituted	**2a**	513	0.97	1.01	0.03	1.84	1.03
	**2b**	522	0.45	0.94	0.55	1.33	1.55
	**2c**	594	0.56	0.81	0.44	1.19	0.98
	**2d**	520	0.52	0.91	0.48	1.29	1.08
	**2e**	531	0.28	0.99	0.72	1.51	1.36
CF_3-funcional. D/A	**3a**	507	0.99	1.01	0.01	2.42	1.03
	**3b**	519	0.98	1.01	0.02	2.02	1.03
	**4a**	568	0.13	0.73	0.87	1.81	1.67
	**4b**	555	0.13	0.91	0.87	1.74	1.63
	**4c**	511	1	1.02	-	-	1.01

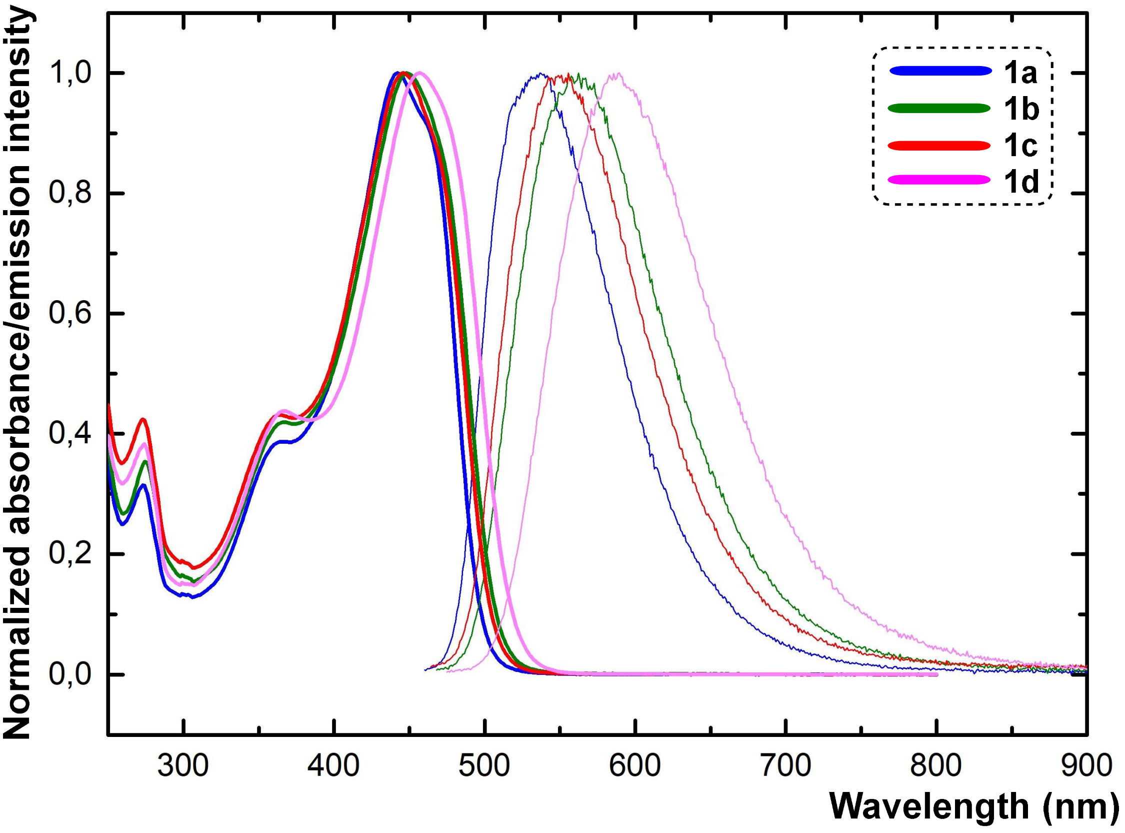

Figure S25. Normalized UV-Vis absorption (bold line) and emission spectra (thin line) of fluorophores **1a** (4-F), **1b** (3,5-F), **1c** (2,4,6-F), and **1d** (2,3,4,5,6-F) measured in THF ($c \approx 5\times10^{-6}$ M).

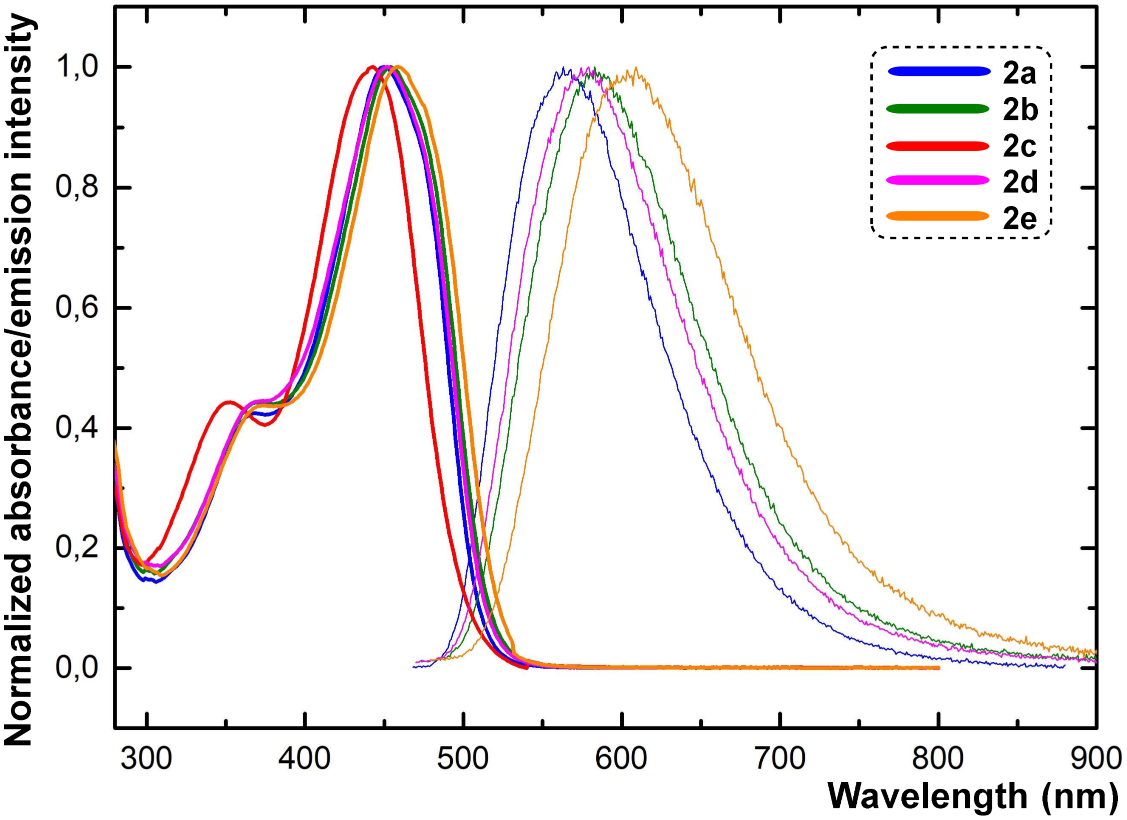

Figure S26. Normalized UV-Vis absorption (bold line) and emission spectra (thin line) of fluorophores **2a** (4-CF_3), **2b** (3,5-CF_3), **2c** (2,4,6-CF_3), **2d** (4-SF_5), and **2e** (3,5-SF_5) measured in THF ($c \approx 5\times10^{-6}$ M).

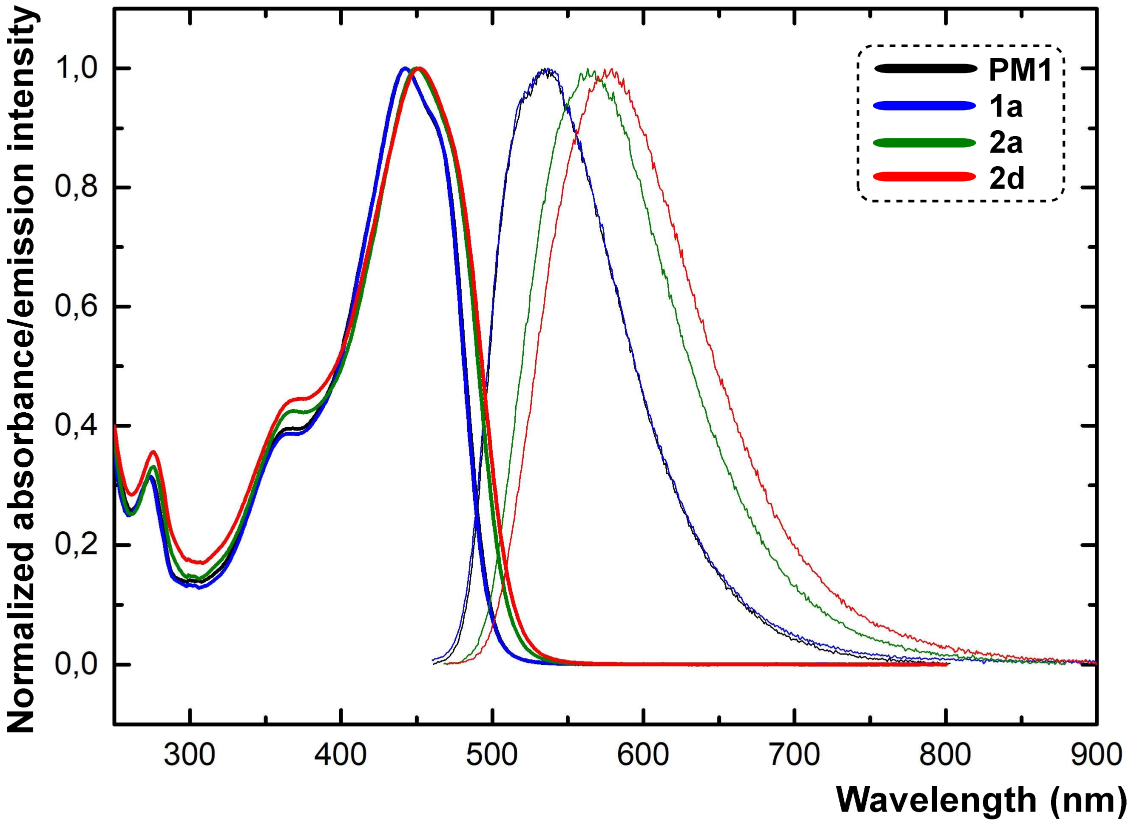

Figure S27. Normalized UV-Vis absorption (bold line) and emission spectra (thin line) of fluorophores **PM1** (4-H), **1a** (4-F), **2a** (4-CF_3), and **2d** (4-SF_5) measured in THF ($c \approx 5\times10^{-6}$ M).

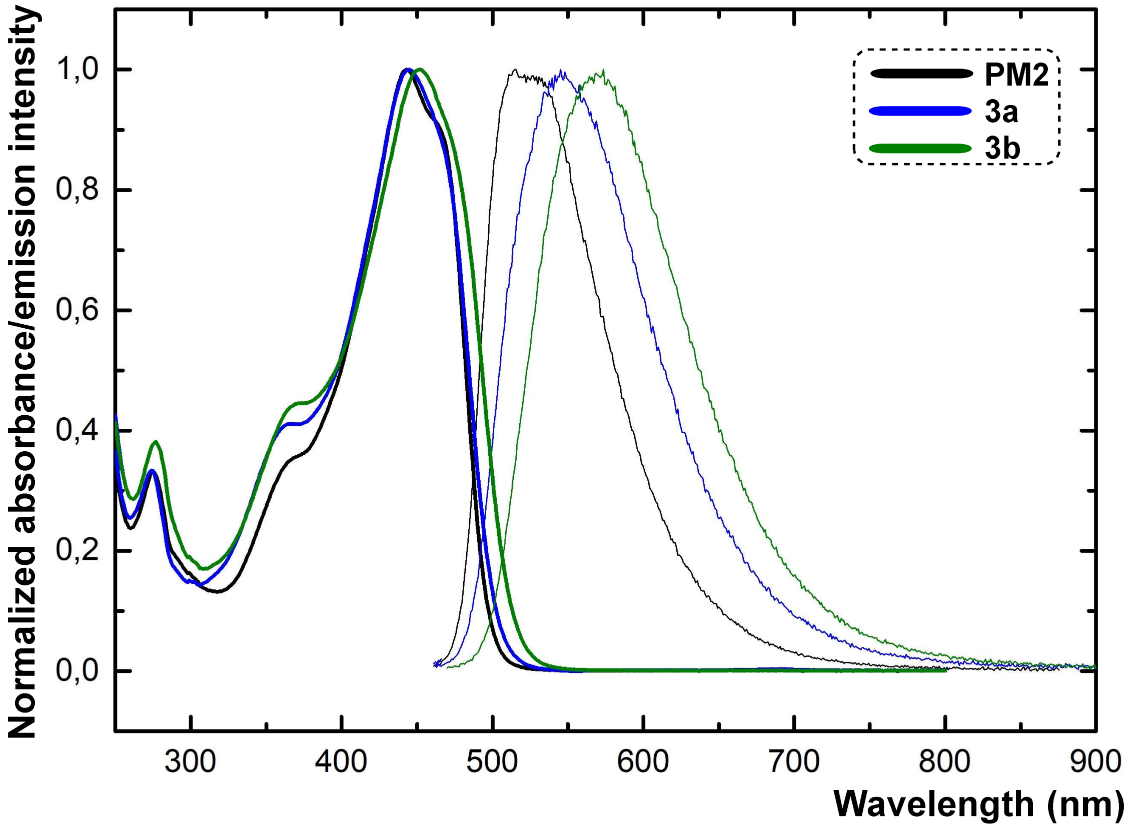

Figure S28. Normalized UV-Vis absorption (bold line) and emission spectra (thin line) of fluorophores **PM2** (4-OCH_3), **3a** (4-OCF_3), and **3b** (4-SCF_3) measured in THF ($c \approx 5\times10^{-6}$ M).

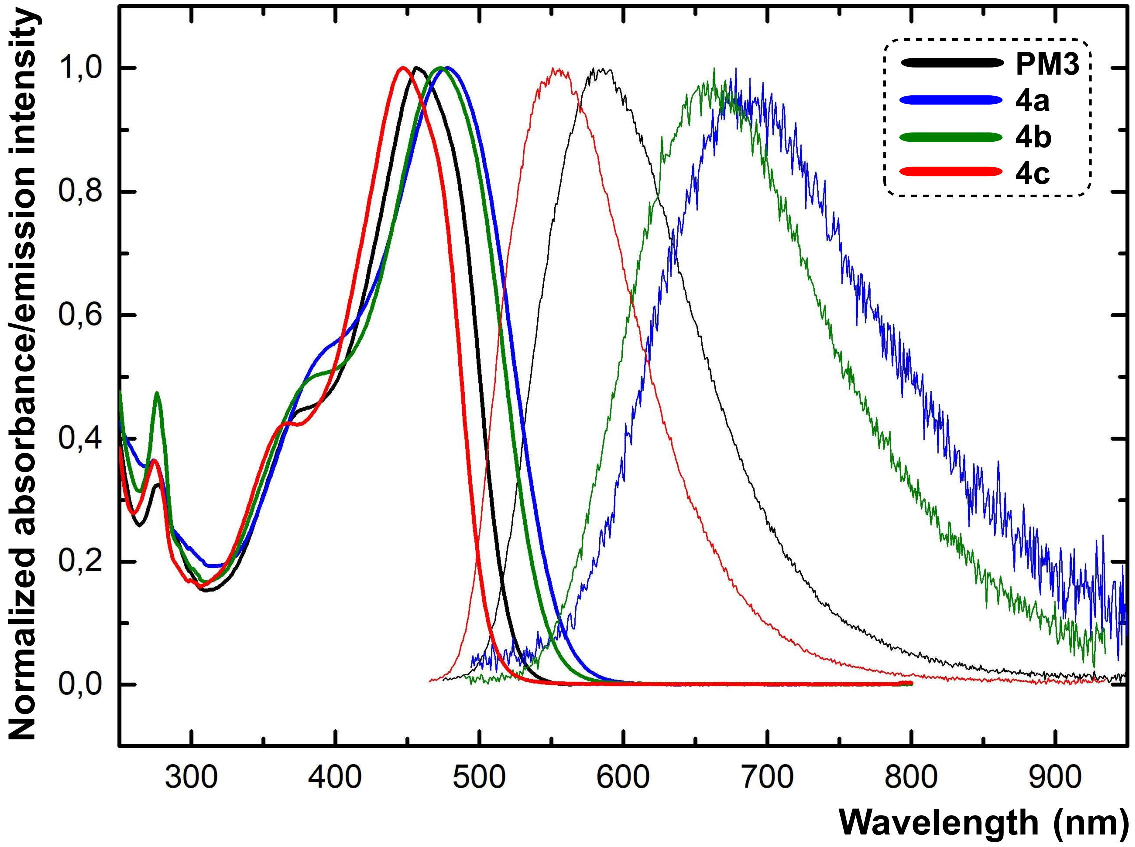

Figure S29. Normalized UV-Vis absorption (bold line) and emission spectra (thin line) of fluorophores **PM3** (4-Ac), **4a** (4-$COCF_3$), **4b** (4-SO_2CF_3), and **4c** (4-OSO_2CF_3) measured in THF ($c \approx 5\times10^{-6}$ M).

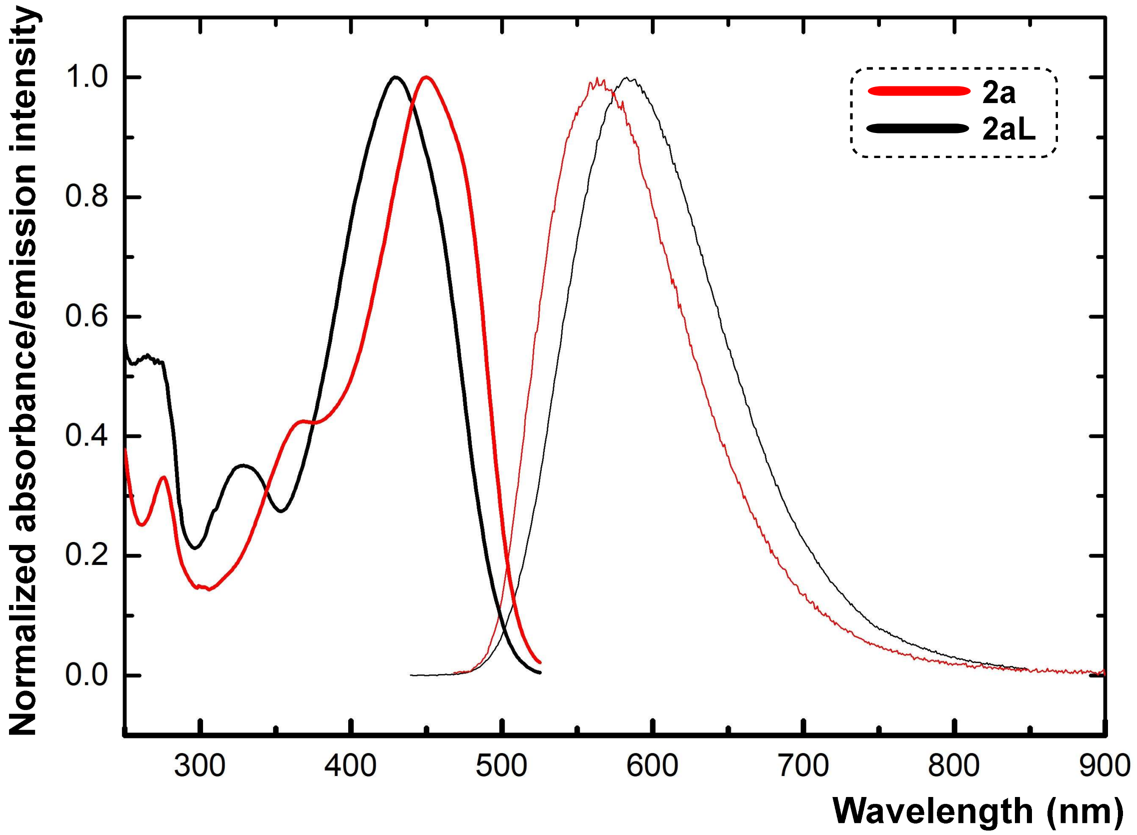

Figure S30. Normalized UV-Vis absorption (bold line) and emission spectra (thin line) of linear and tripodal fluorophores **2aL** and **2a** measured in THF ($c \approx 5\times10^{-6}$ M).

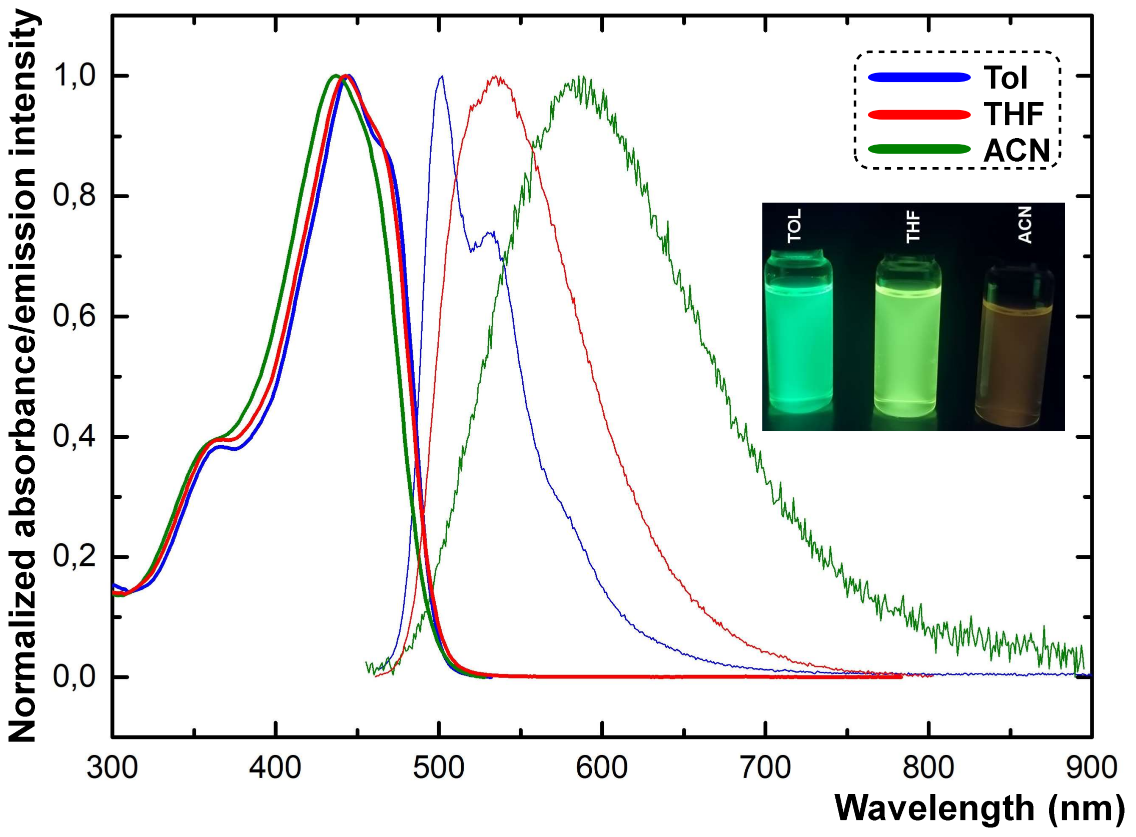

Figure S31. Normalized absorption (bold line) and emission spectra (thin line) of fluorophore **PM1** measured in three solvents of different polarity ($c \approx 5\times10^{-6}$ M). A photograph of **PM1** solutions under UV lamp irradiation (λ_{em} = 254 nm) is shown as an inset.

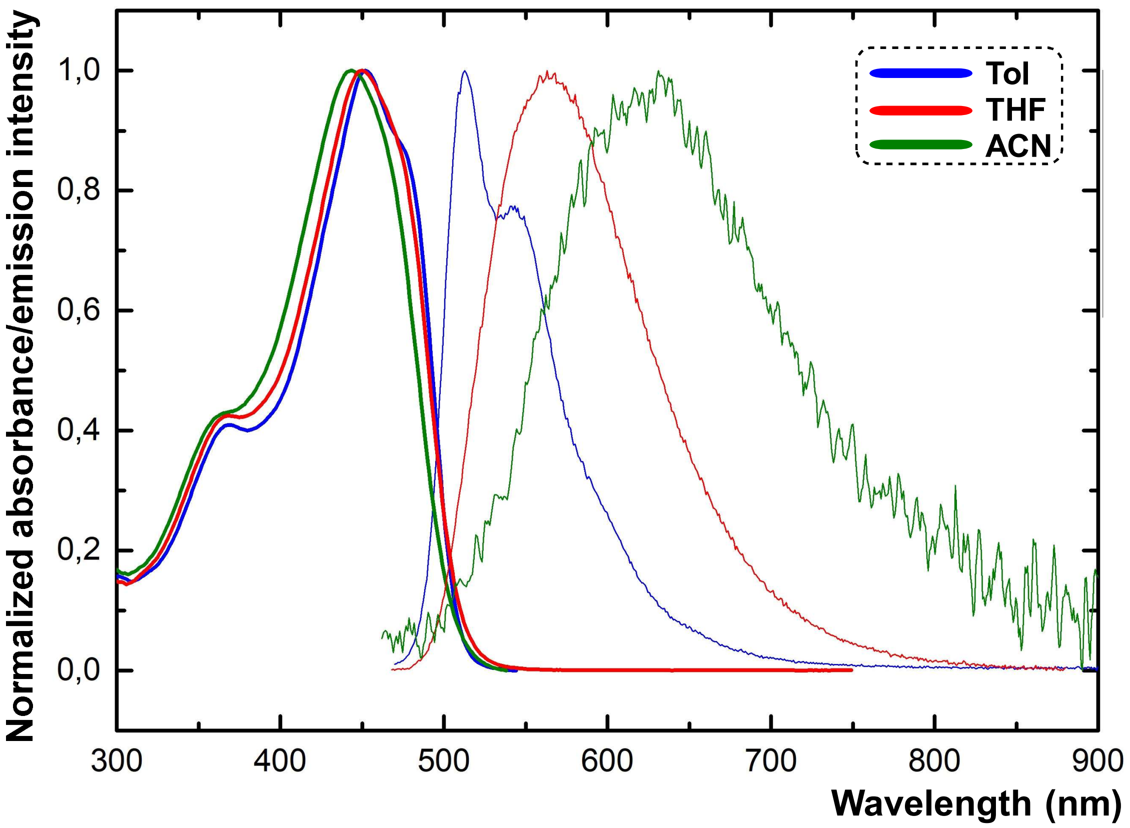

Figure S32. Normalized absorption (bold line) and emission spectra (thin line) of fluorophore **2a** measured in three solvents of different polarity ($c \approx 5{\times}10^{-6}$ M).

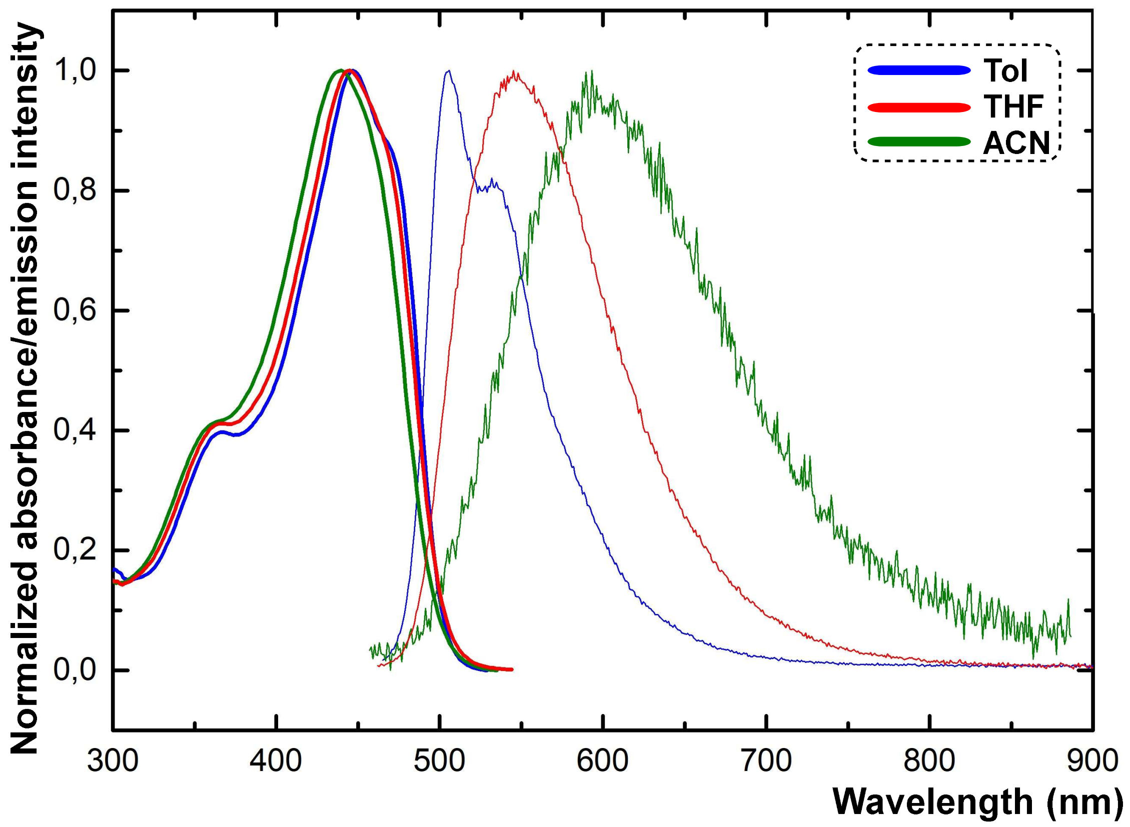

Figure S33. Normalized absorption (bold line) and emission spectra (thin line) of fluorophore **3a** measured in three solvents of different polarity ($c \approx 5{\times}10^{-6}$ M).

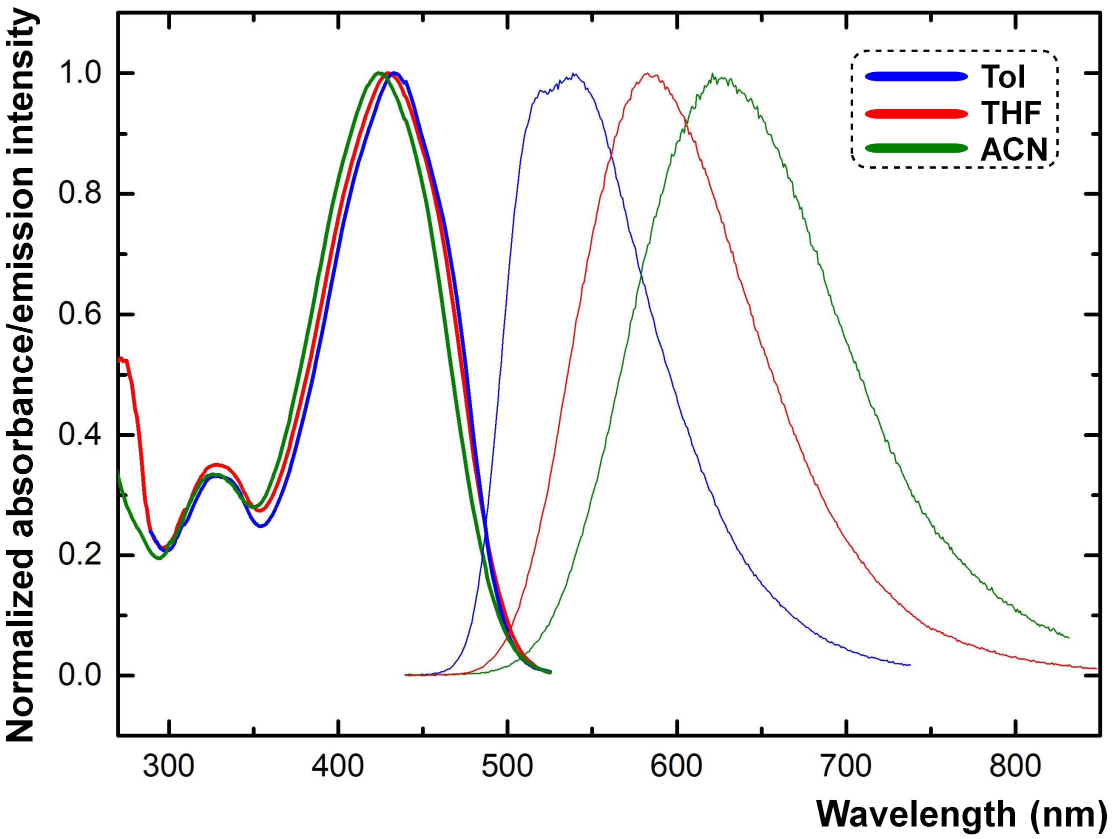

Figure S34. Normalized absorption (bold line) and emission spectra (thin line) of linear fluorophore **2aL** measured in three solvents of different polarity ($c \approx 5\times10^{-6}$ M).

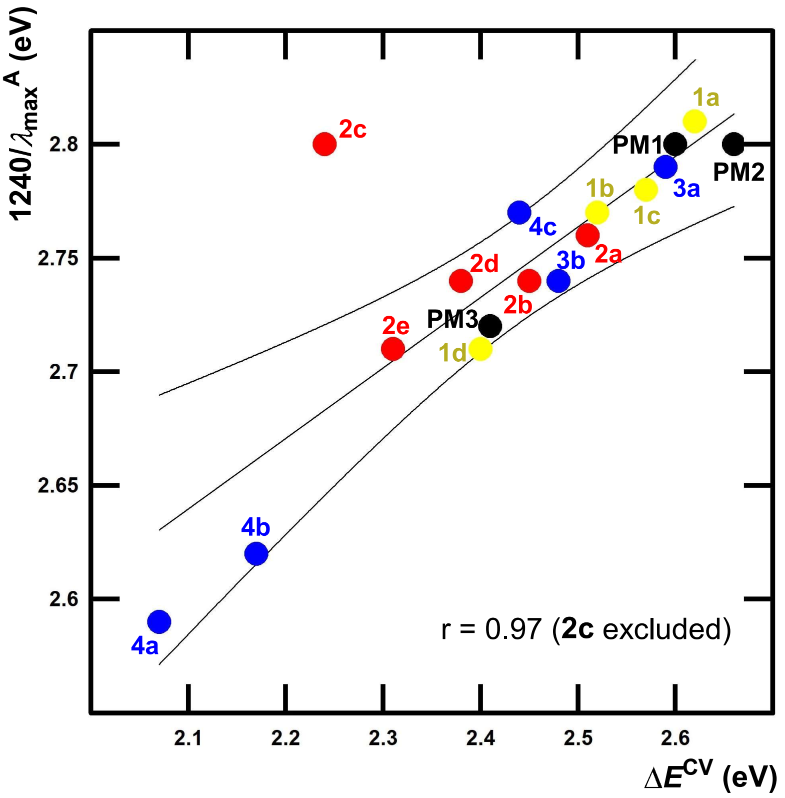

Figure S35. Correlation of the energy of the longest-wavelength absorption maxima $1240/\lambda_{max}^{A}$ and the electrochemical gap ΔE (both recorded in THF).

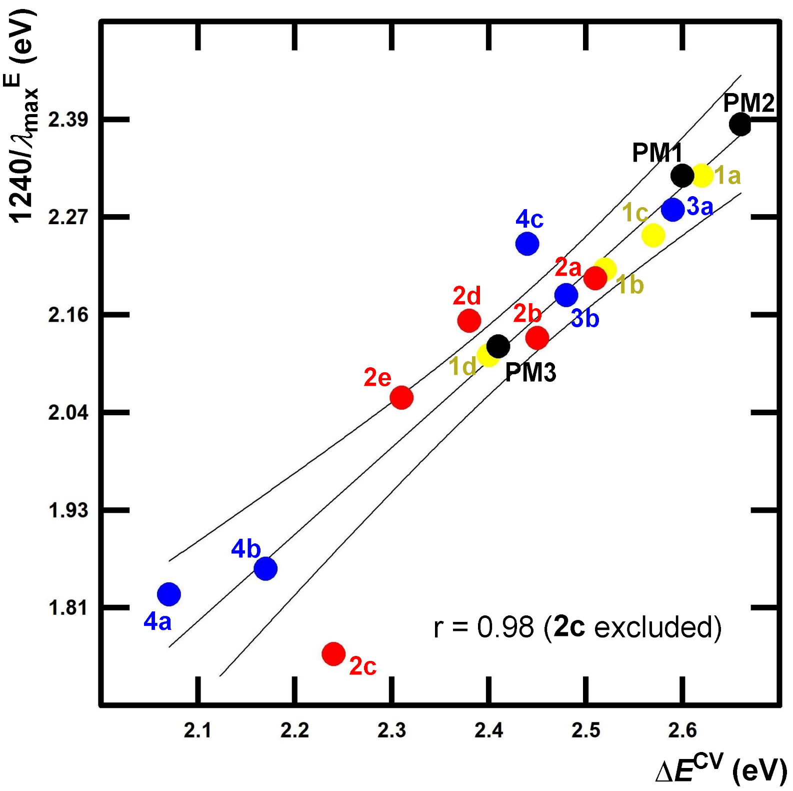

Figure S36. Correlation of the energy of the longest-wavelength emission maxima 1240/λ_{max}^{E} and the electrochemical gap ΔE (both recorded in THF).

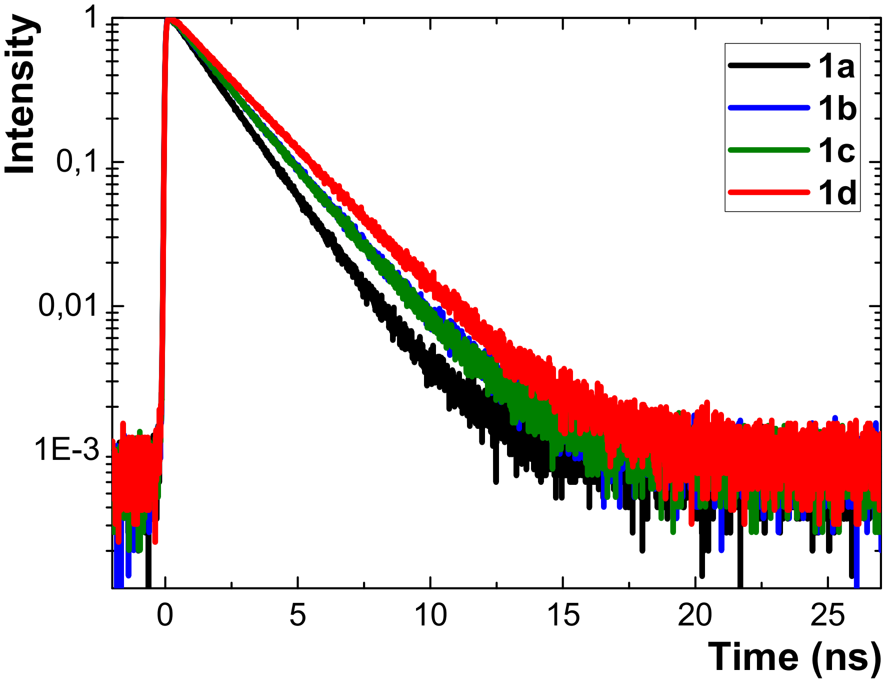

Figure S37. Fluorescence decays in the ns timescale for fluorophores **1a** (4-F), **1b** (3,5-F), **1c** (2,4,6-F), **1d** (2,3,4,5,6-F) measured in THF ($c \approx 5\times10^{-6}$ M).

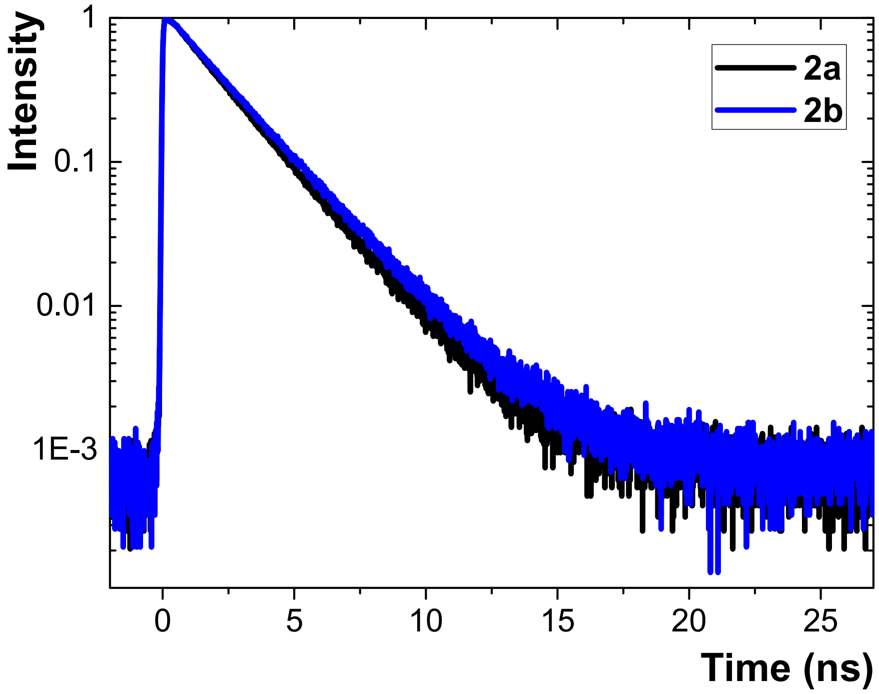

Figure S38. Fluorescence decays in the ns timescale for fluorophores **2a** (4-CF_3), **2b** (3,5-CF_3) measured in THF ($c \approx 5{\times}10^{-6}$ M).

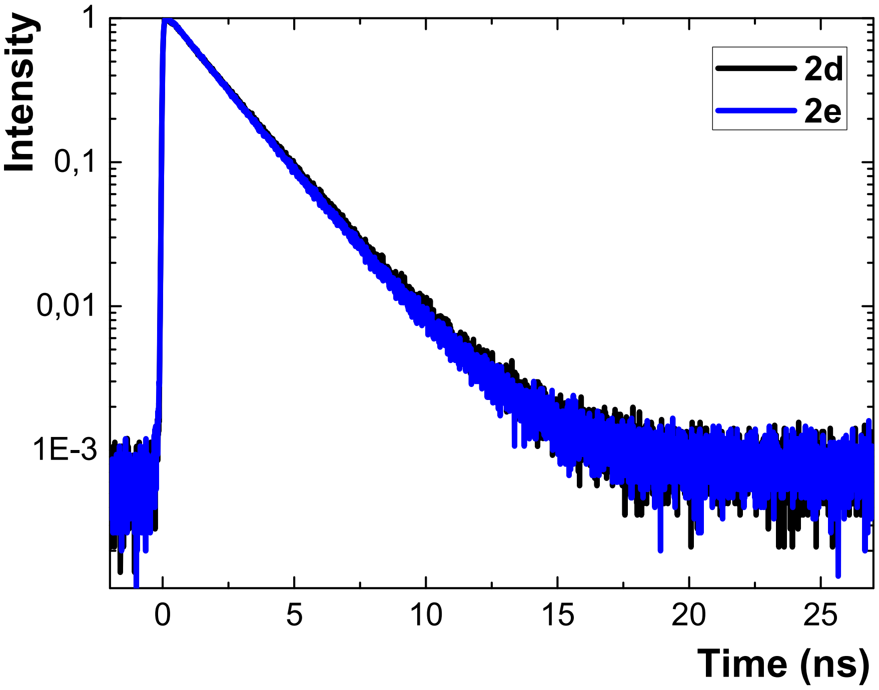

Figure S39. Fluorescence decays in the ns timescale for fluorophores **2d** (4-SF_5)**,** **2e** (3,5-SF_5) measured in THF ($c \approx 5{\times}10^{-6}$ M).

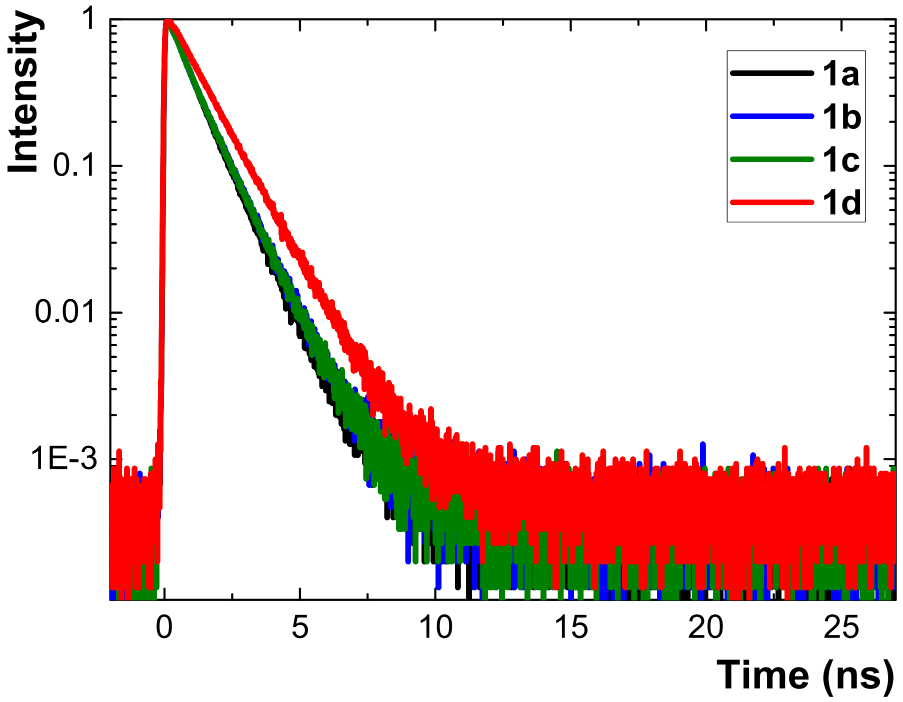

…

Figure S40. Fluorescence decays in the ns timescale for fluorophores **1a** (4-F), **1b** (3,5-F), **1c** (2,4,6-F), **1d** (2,3,4,5,6-F) measured in toluene ($c \approx 5\times10^{-6}$ M).

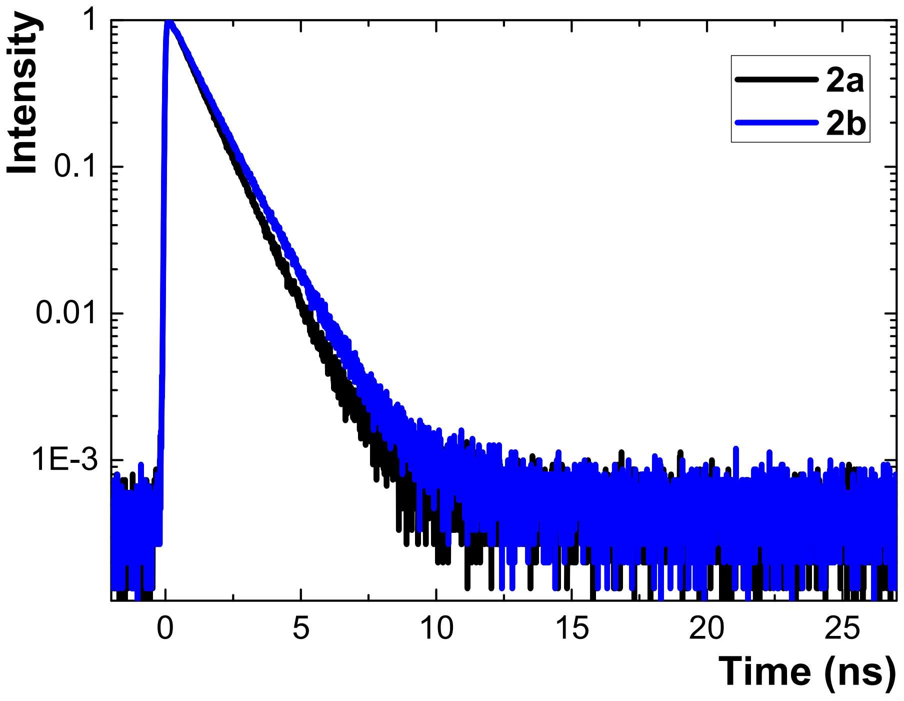

Figure S41. Fluorescence decays in the ns timescale for fluorophores **2a** (4-CF_3), **2b** (3,5-CF_3) measured in toluene ($c \approx 5\times10^{-6}$ M).

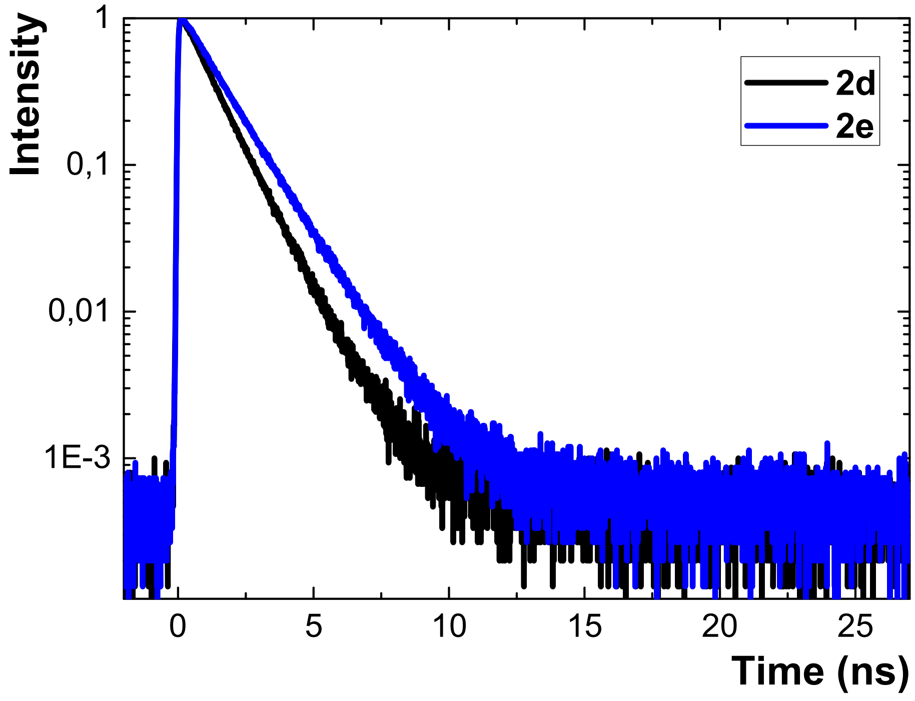

Figure S42. Fluorescence decays in the ns timescale for fluorophores **2d** (4-SF_5)**,** **2e** (3,5-SF_5) measured in toluene ($c \approx 5\times10^{-6}$ M).

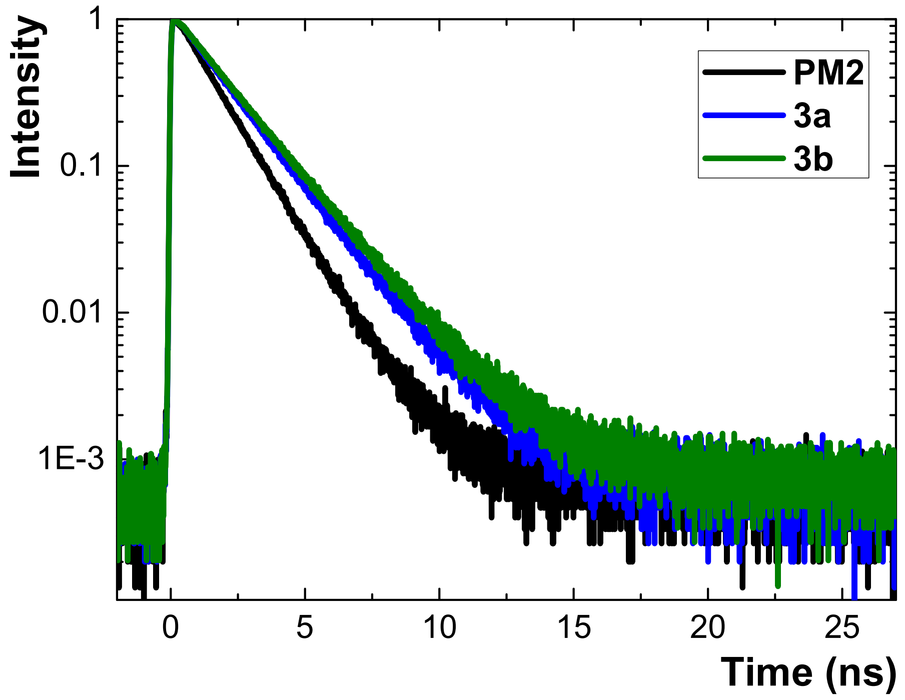

Figure S43. Fluorescence decays in the ns timescale for fluorophores **PM2** (4-OCH_3)**,** **3a** (4-OCF_3)**,** **3b** (4-SCF_3) in THF ($c \approx 5\times10^{-6}$ M).

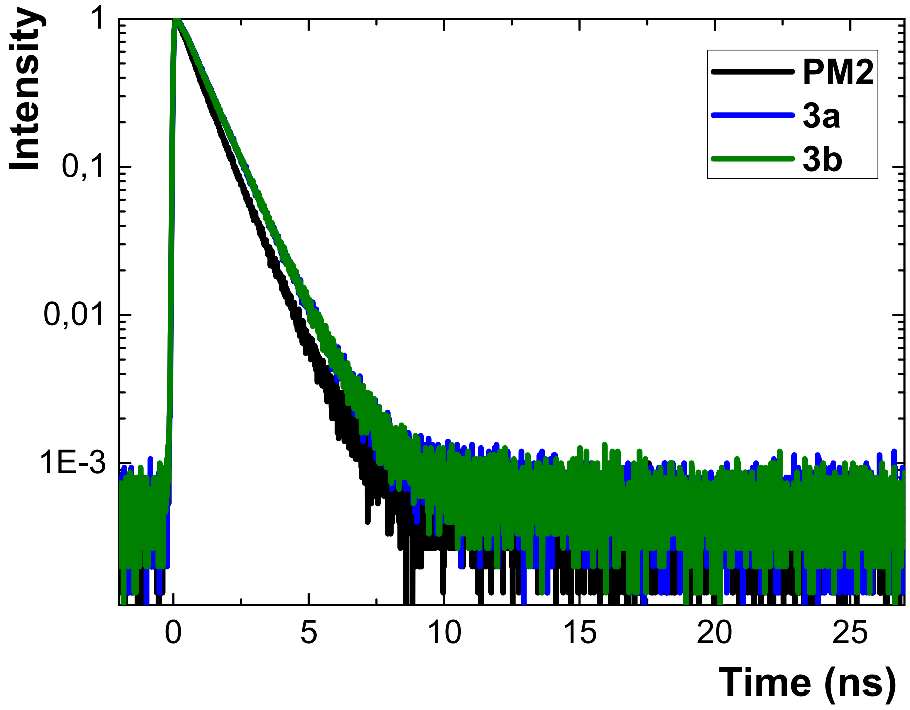

Figure S44. Fluorescence decays in the ns timescale for fluorophores **PM2** (4-OCH_3)**, 3a** (4-OCF_3)**, 3b** (4-SCF_3) in toluene ($c \approx 5\times10^{-6}$ M).

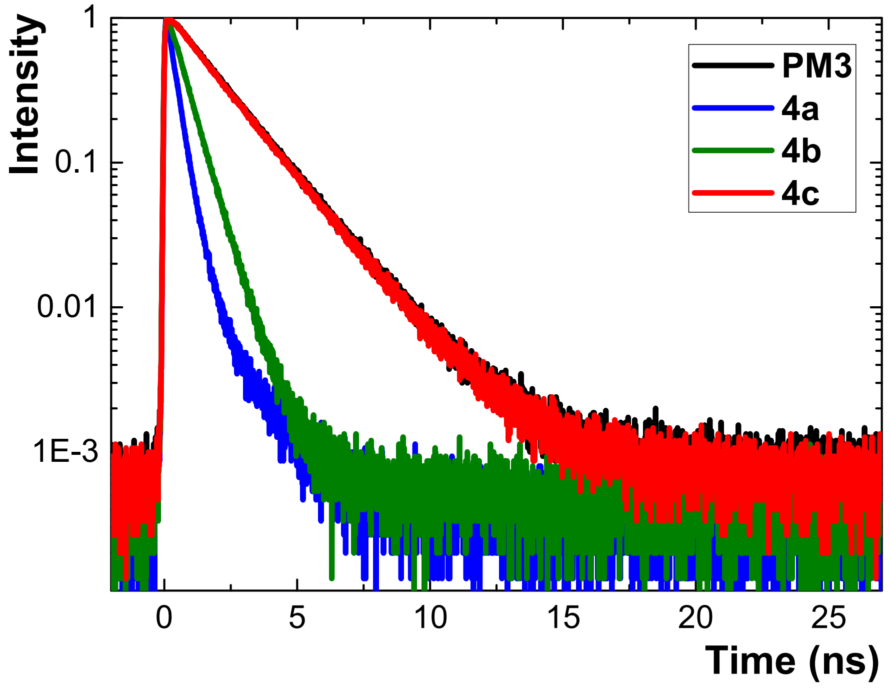

Figure S45. Fluorescence decays in the ns timescale for fluorophores **PM3** (4-$COCH_3$), **4a** (4-$COCF_3$), **4b** (4-SO_2CF_3), **4c** (4-OSO_2CF_3) in THF ($c \approx 5\times10^{-6}$ M).

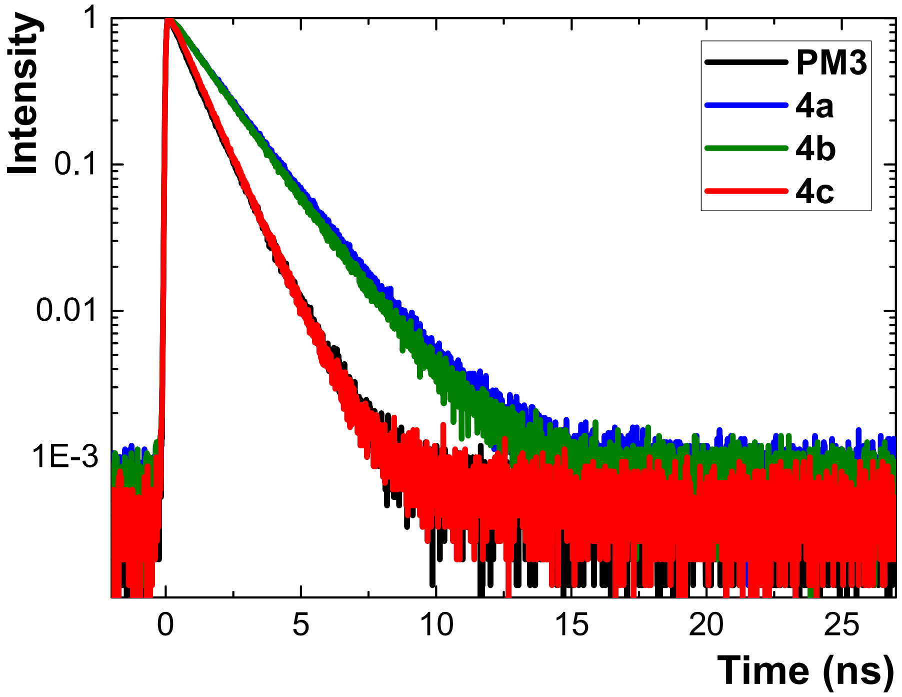

Figure S46. Fluorescence decays in the ns timescale for fluorophores **PM3** (4-$COCH_3$), **4a** (4-$COCF_3$), **4b** (4-SO_2CF_3), **4c** (4-OSO_2CF_3) in toluene ($c \approx 5{\times}10^{-6}$ M).

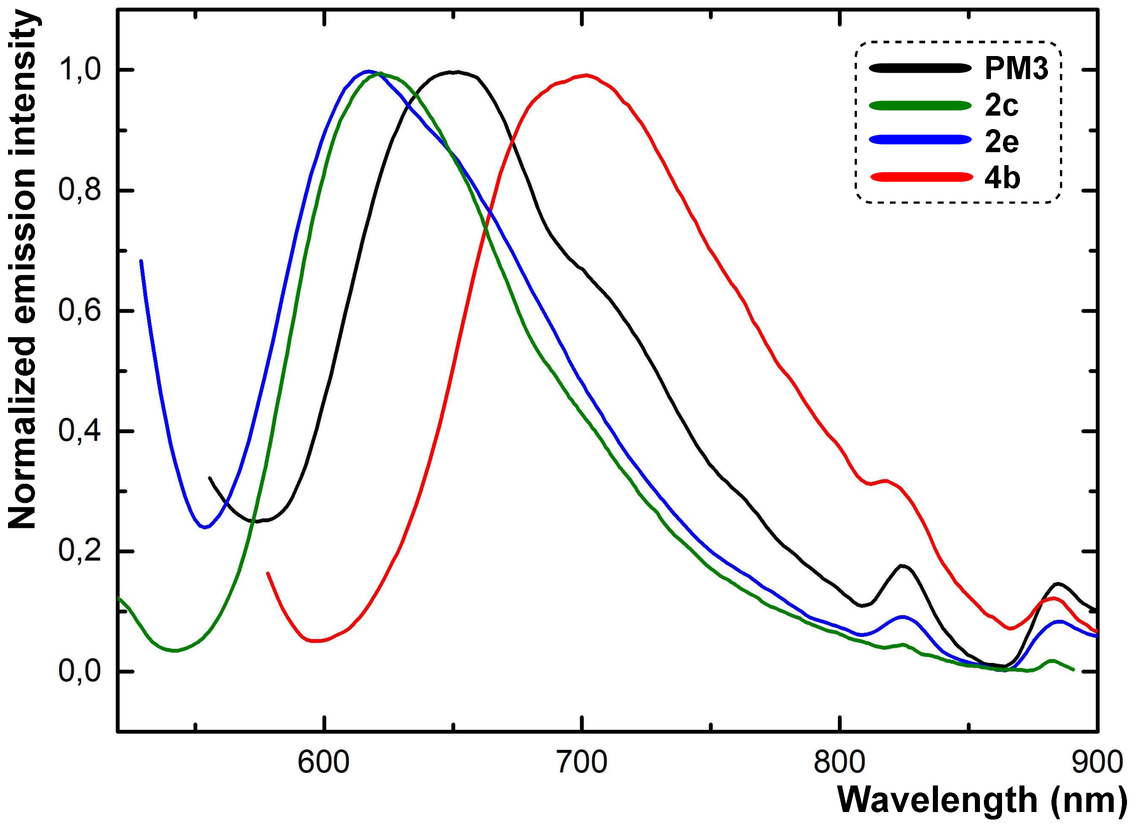

Figure S47. Normalized and smoothed emission spectra of fluorophores **PM3** (4-Ac), **2c** (2,4,6-CF_3), **2e** (3,5-SF_5), and **4b** (4-SO_2CF_3) in the solid state excited at their absorption maxima in THF.

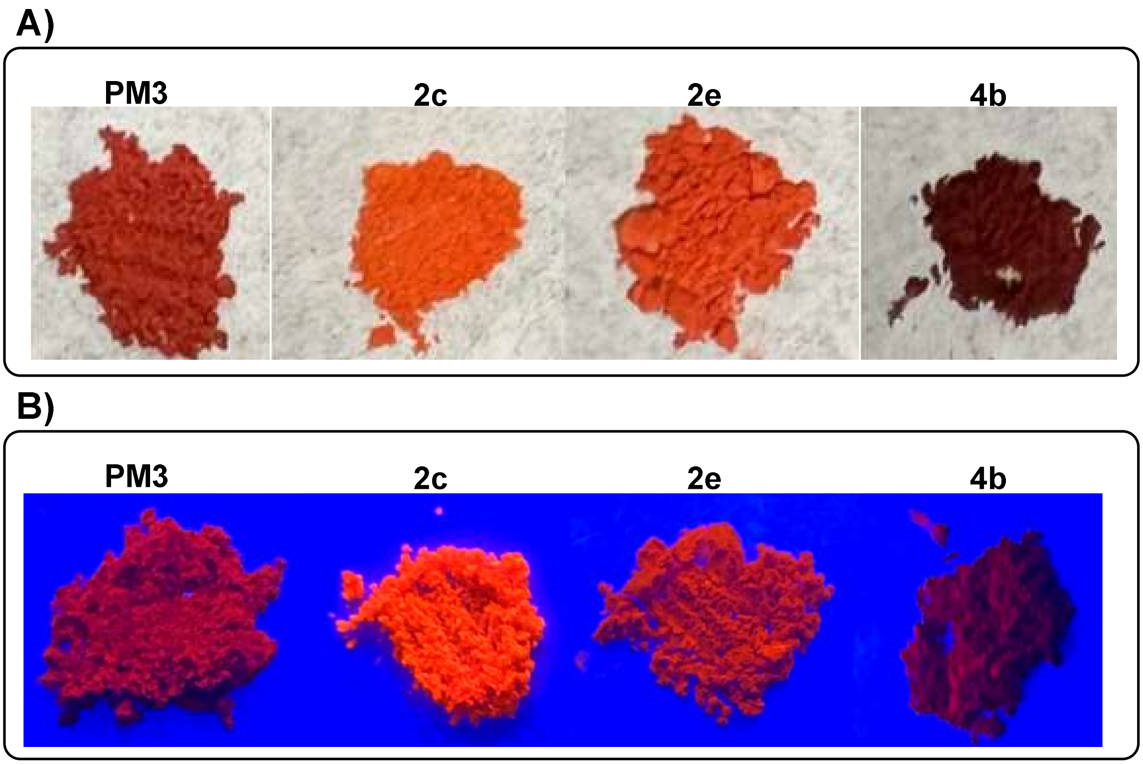

Figure S48. Photographs of the solid-state samples **PM3** (4-Ac), **2c** (2,4,6-CF_3), **2e** (3,5-SF_5), and **4b** (4-SO_2CF_3) taken under (A) daylight and (B) UV lamp irradiation (λ_{em} = 254 nm).

7. DFT calculations

Spatial and electronic properties of target fluorophores **PM1–3** and **1–4** were investigated using Gaussian®16W software package.[25] The initial geometries as well as energies of the frontier molecular orbitals were optimized/calculated by DFT B3LYP/6-311+G(2d,p) method in THF. The optimized geometries were used for all further calculations. The theoretical electronic absorption spectra were calculated at TD-DFT (nstates = 8) B3LYP/6-311++G(2d,p) and TD DFT (nstates = 8) CAM-B3LYP/6-311++G(2d,p) level in THF. All visualizations were prepared in OPChem[26] or OPStat.[27]

In addition to the NMR analysis, we have confirmed the conformations the geometrical arrangement around the two double bonds using DFT calculation. The lowest conformational energies were calculated for the s-trans/s-trans isomer (Fig. S49), which is probably due to an efficient conjugation of the aromatic thiophene and the double bond, the smaller steric interaction, and an eventual formation of a weak hydrogen bond between the thiophene sulfur atom and the hydrogen atom of the sp^2-hybridized double bond. These assumptions are consistent with the results ox X-Ray analysis of the linear analogue **2aL**, which confirms the s-trans configuration of both double bonds.

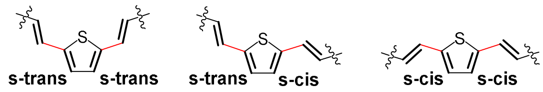

Figure S49. The possible spatial arrangement of double bonds including thiophene and two ethylene π-spacers.

Due to the π-extended branches and the expected C3-symmetry, achieving the minimum energy during optimization/symmetrization of the geometry was the main issue. To address this, we have employed a fixed number of optimization steps but even a slight deviation from the C3-symmetry have a significant effect on the calculated UV-VIS spectra. Hence, a deviation from the C3-symmetry and the chosen functional (B3LYP vs. CAM-B3LYP) fundamentally affect the shape (oscillator strength) and position of the calculated absorption bands.

Main differences between the tripodal and linear fluorophores 2a and 2aL:

Molecule **2a**, like all investigated tripodal fluorophores, possesses two pairs of energetically degenerated FMOs (HOMO–2/HOMO–1 and LUMO/LUMO+1), while these FMOs are not degenerated at all within the linear analogue **2aL** (see Table 3). Whereas the energy of the HOMO is almost identical for both fluorophores (E_{HOMO} = −5.00/–4.98 eV for **2a**/**2aL**), the LUMO is clearly elevated by 0.2 eV in the case of the linear analogue (E_{HOMO} = −2.58/–2.38 eV for **2a**/**2aL**) reflecting the mutual energy gap difference. In both cases, the HOMO is localized on the amino donor, however the HOMO−1 still occupies the amino donor within linear **2aL**, while this FMO is distributed over two π-thiophene branches within tripodal analogue **2a** (Figs. S55 and S60). The HOMO–2 is further spread over thiophene-based π-linker (**2a**) or directly localized on the thiophene´s sulfur atom (**2aL**). Whereas the LUMOs are centralized especially on the amino donor and thiophene-based π-systems within the particular branches (tripodal **2a**), the positions of LUMOs are shifted from thiophene π-linker to the terminal phenyl with attached CF_3-group as energy elevated (linear **2aL**).

These discrepancies in the discrete levels and the localization of FMOs in linear and tripodal analogues imply slight differences between their ground and excited states, which are reflected in the calculated UV-Vis spectra (B3LYP: Figs. S61 and S63; CAM-B3LYP: Figs. S65 and S67). Hence, the calculated CT-band of the linear **2aL** is significantly blue shifted in

comparison with the tripodal **2a** (B3LYP: λ_{max} = 598 → 534 nm; CAM-B3LYP: λ_{max} = 459 → 448 nm). Due to the energetically non-degenerated states, the CT-band of linear **2aL** is created only by one transition from the HOMO to the LUMO with the greatest oscillator strength (f = 1.49). The corresponding higher energy band (shoulder) is due to a single transition from the HOMO−1 to the LUMO with a significantly (≈ 5 times) lower oscillator strength (f = 0.33) compared to the tripodal derivative.

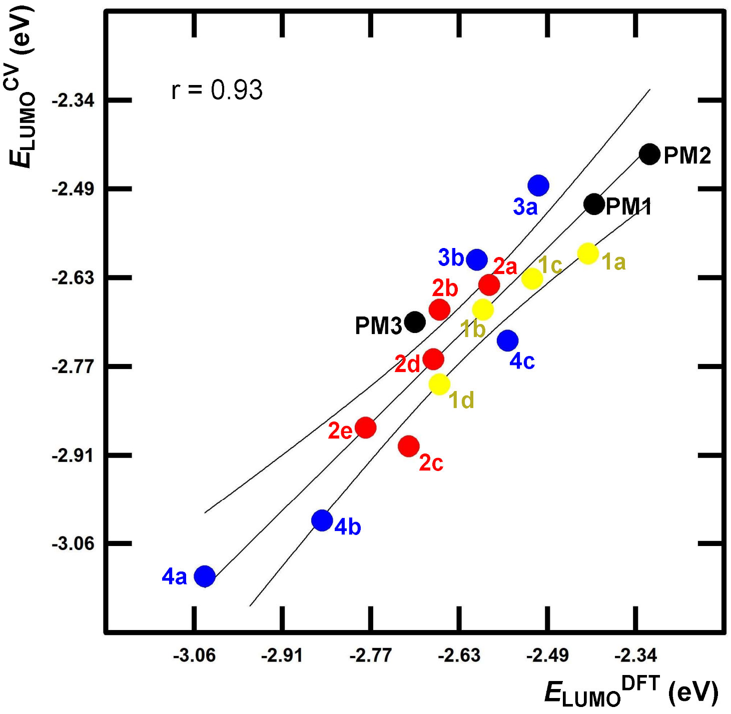

Figure S50. Correlation of the electrochemical E_{LUMO}^{CV} and the calculated E_{LUMO}^{DFT} (both recorded in THF).

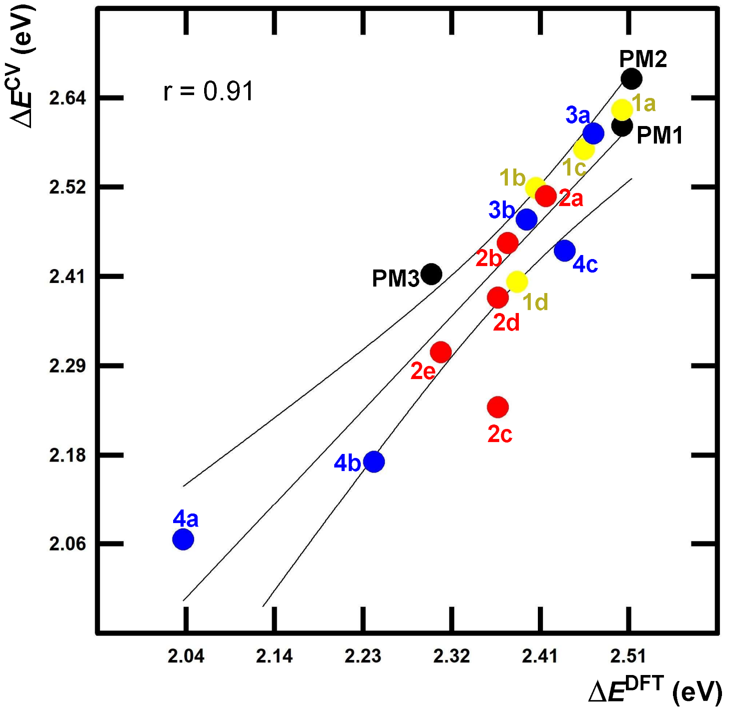

Figure S51. Correlation of the electrochemical H-L gap ΔE^{CV} and the calculated H-L gap ΔE^{DFT} (both recorded in THF).

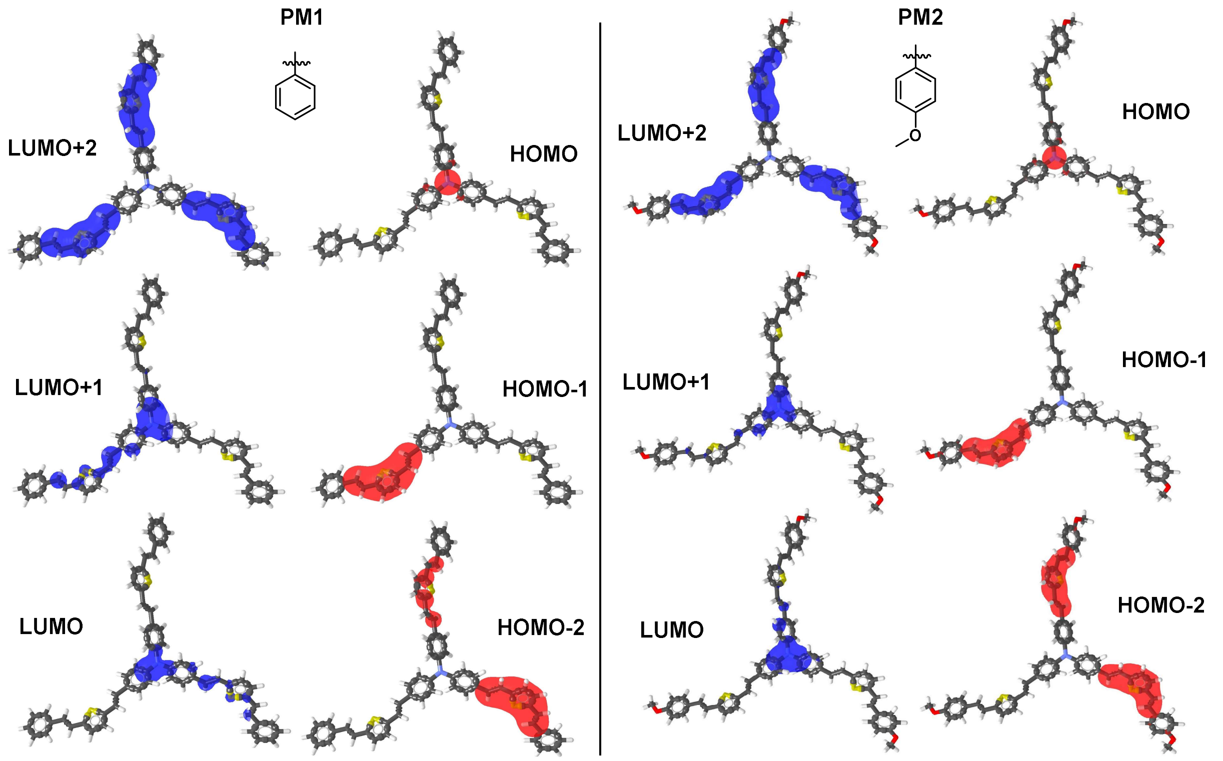

Figure S52. DFT visualization of the frontier molecular orbitals in fluorophores **PM1–PM2**. The HOMO(−1,−2) is shown in red, the LUMO(+1,+2) in blue.

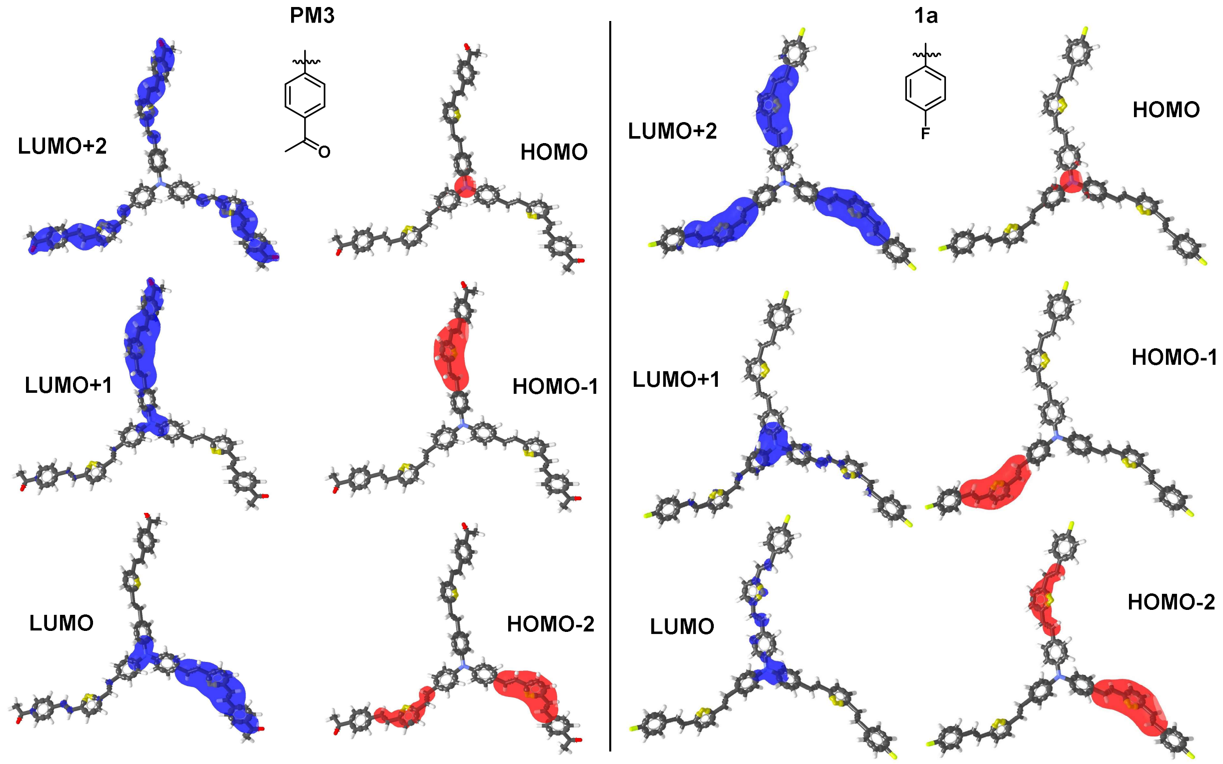

Figure S53. DFT visualization of the frontier molecular orbitals in fluorophores **PM3–1a**. The HOMO(−1,−2) is shown in red, the LUMO(+1,+2) in blue.

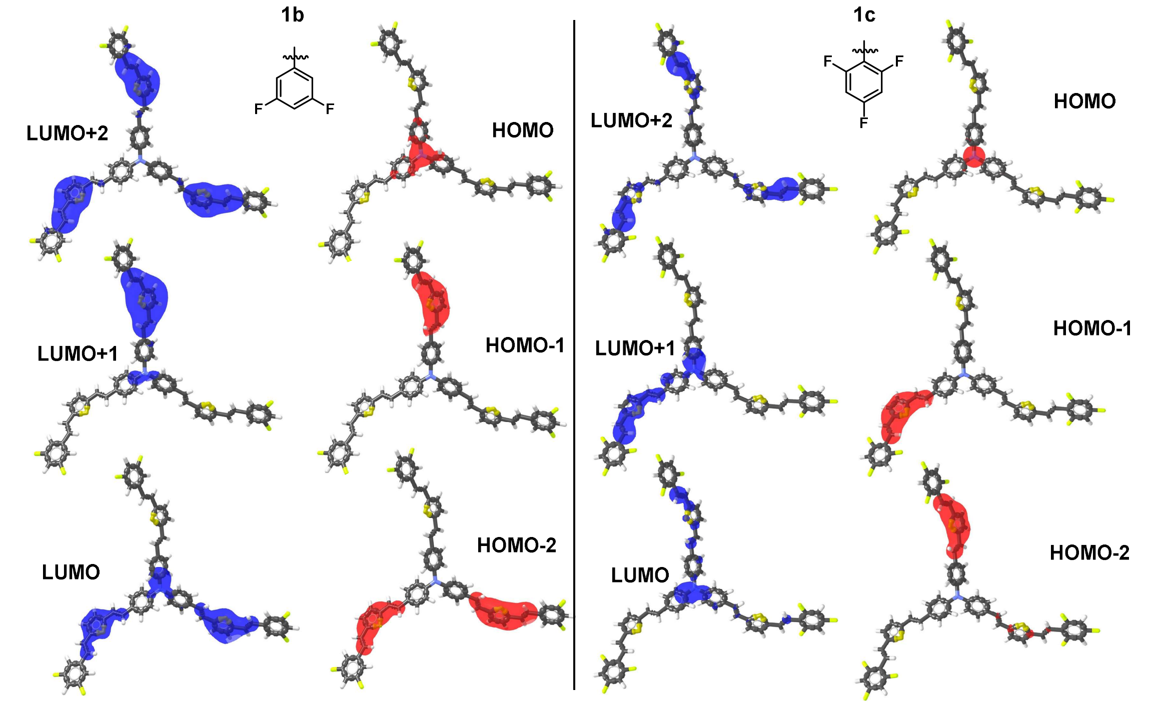

Figure S54. DFT visualization of the frontier molecular orbitals in fluorophores **1b–1c**. The HOMO(−1,−2) is shown in red, the LUMO(+1,+2) in blue.

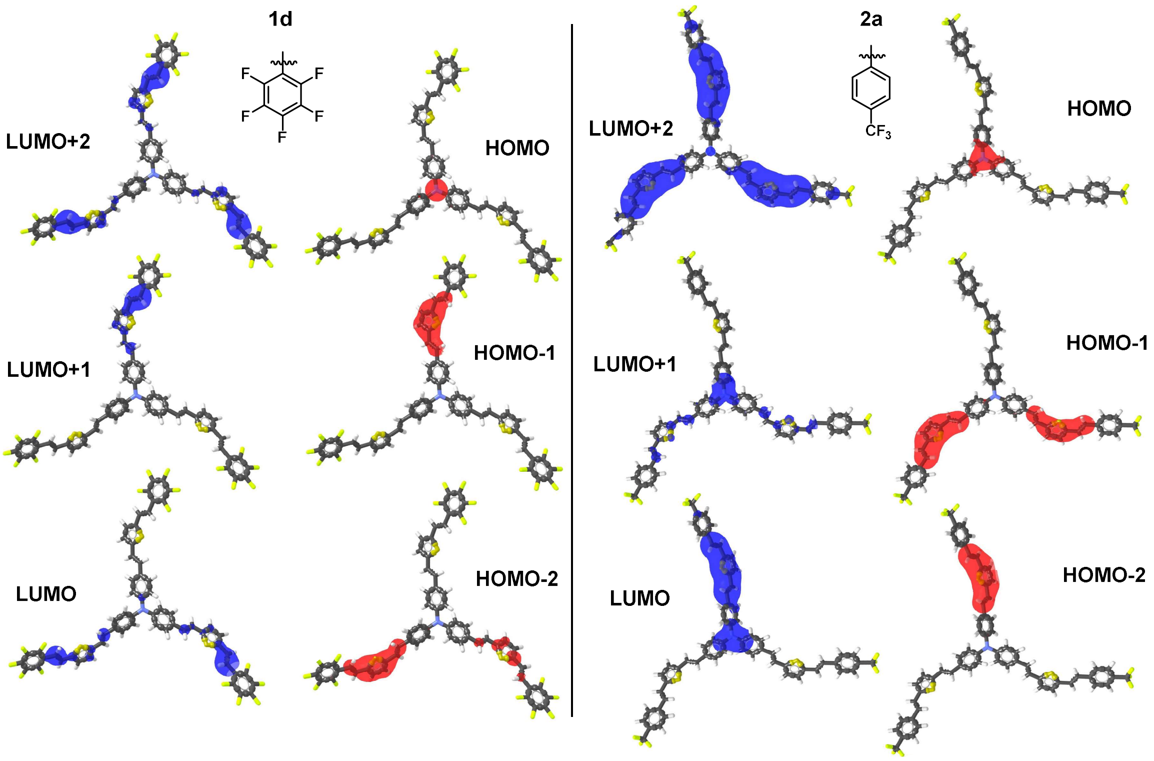

Figure S55. DFT visualization of the frontier molecular orbitals in fluorophores **1d–2a**. The HOMO(−1,−2) is shown in red, the LUMO(+1,+2) in blue.

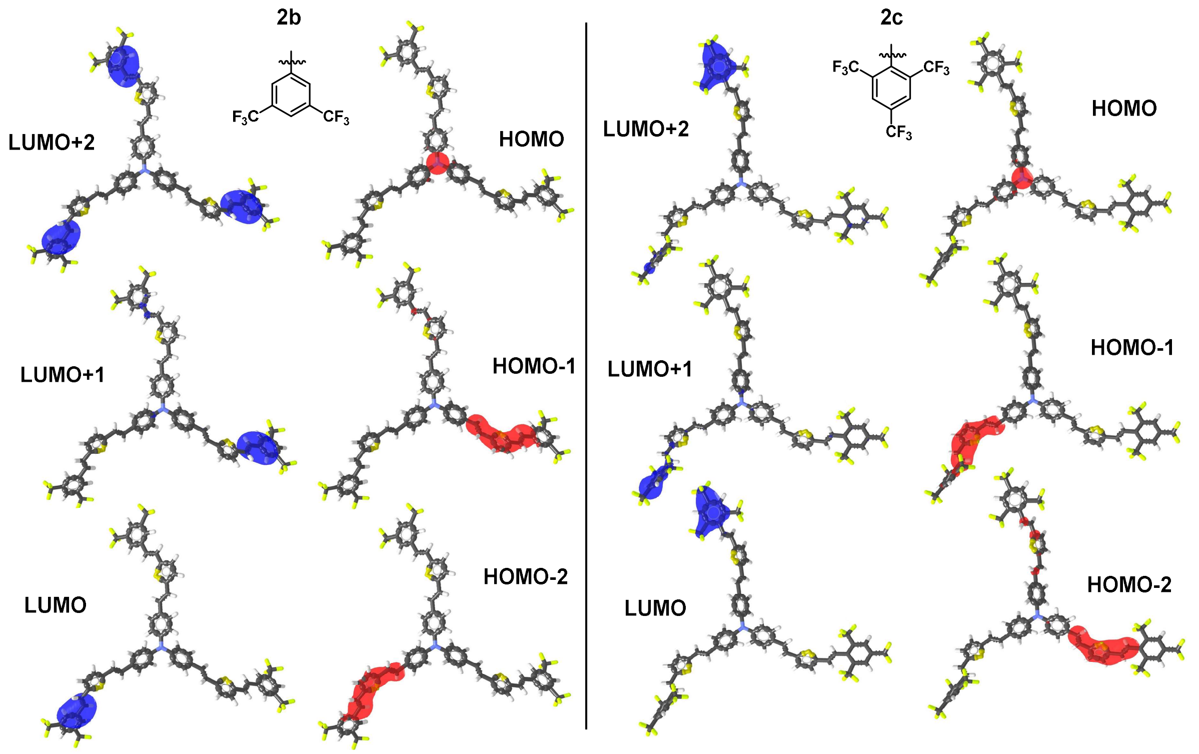

Figure S56. DFT visualization of the frontier molecular orbitals in fluorophores **2b**–**2c**. The HOMO(−1,–2) is shown in red, the LUMO(+1,+2) in blue.

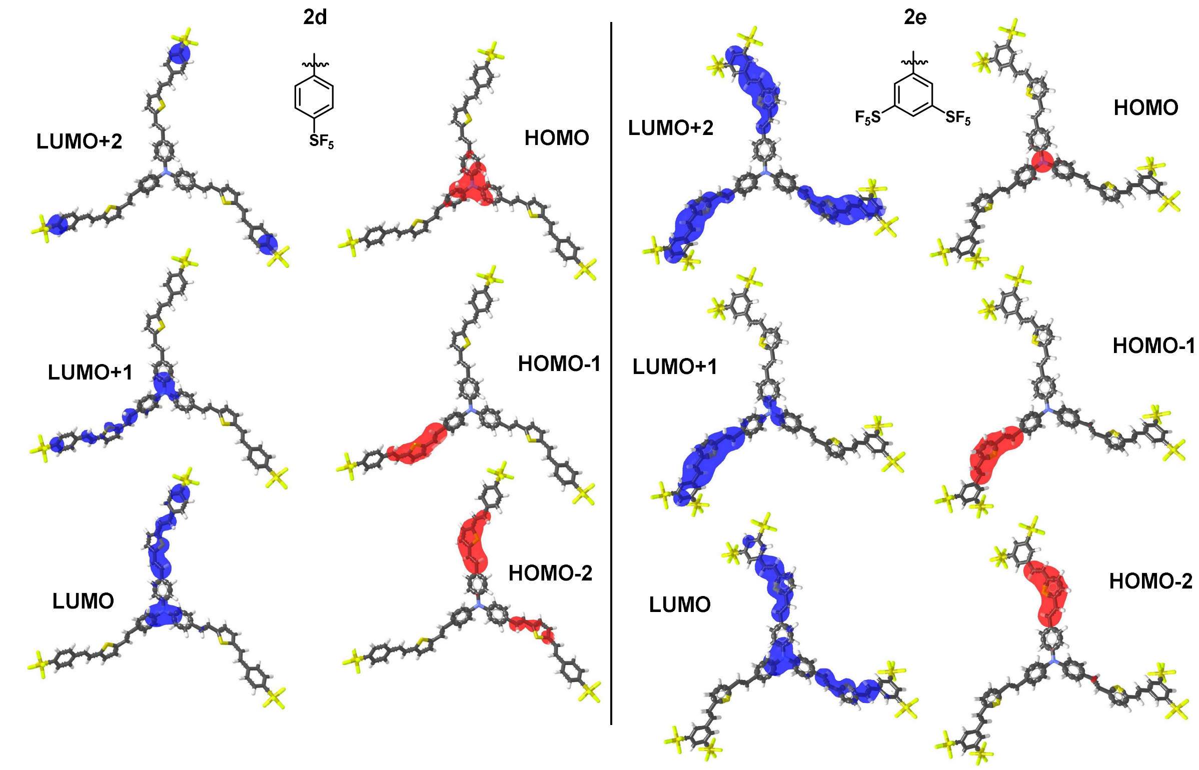

Figure S57. DFT visualization of the frontier molecular orbitals in fluorophores **2d**–**2e**. The HOMO(−1,–2) is shown in red, the LUMO(+1,+2) in blue.

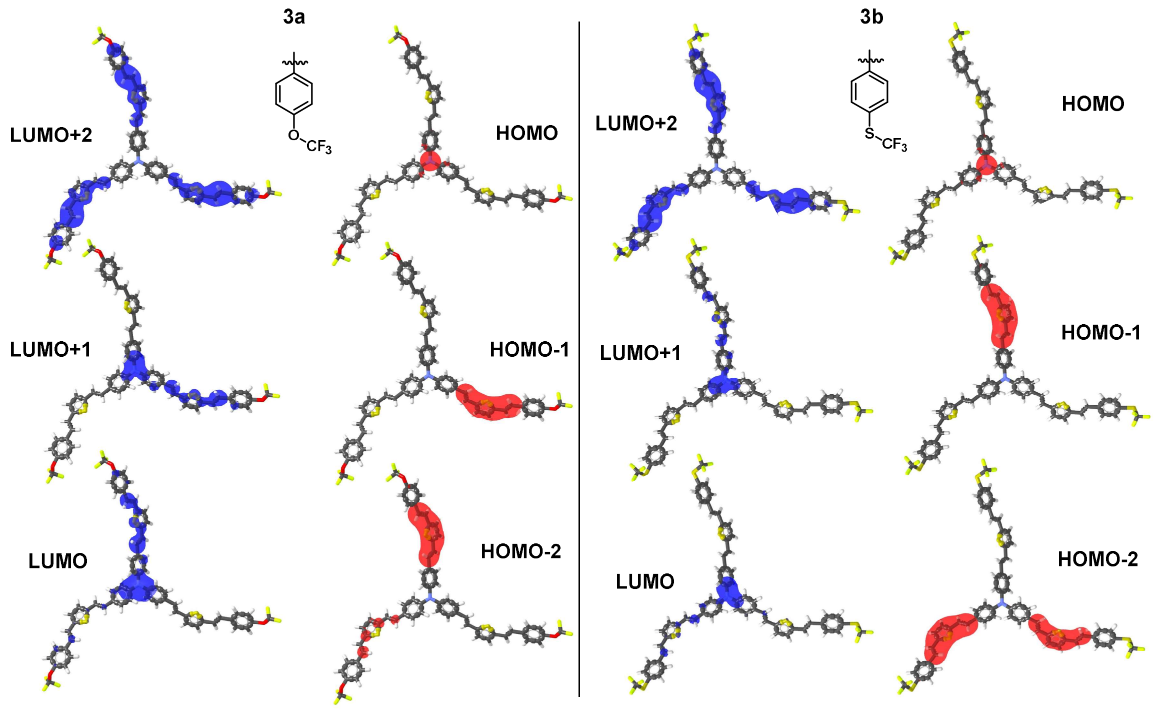

Figure S58. DFT visualization of the frontier molecular orbitals in fluorophores **3a**–**3b**. The HOMO(−1,−2) is shown in red, the LUMO(+1,+2) in blue.

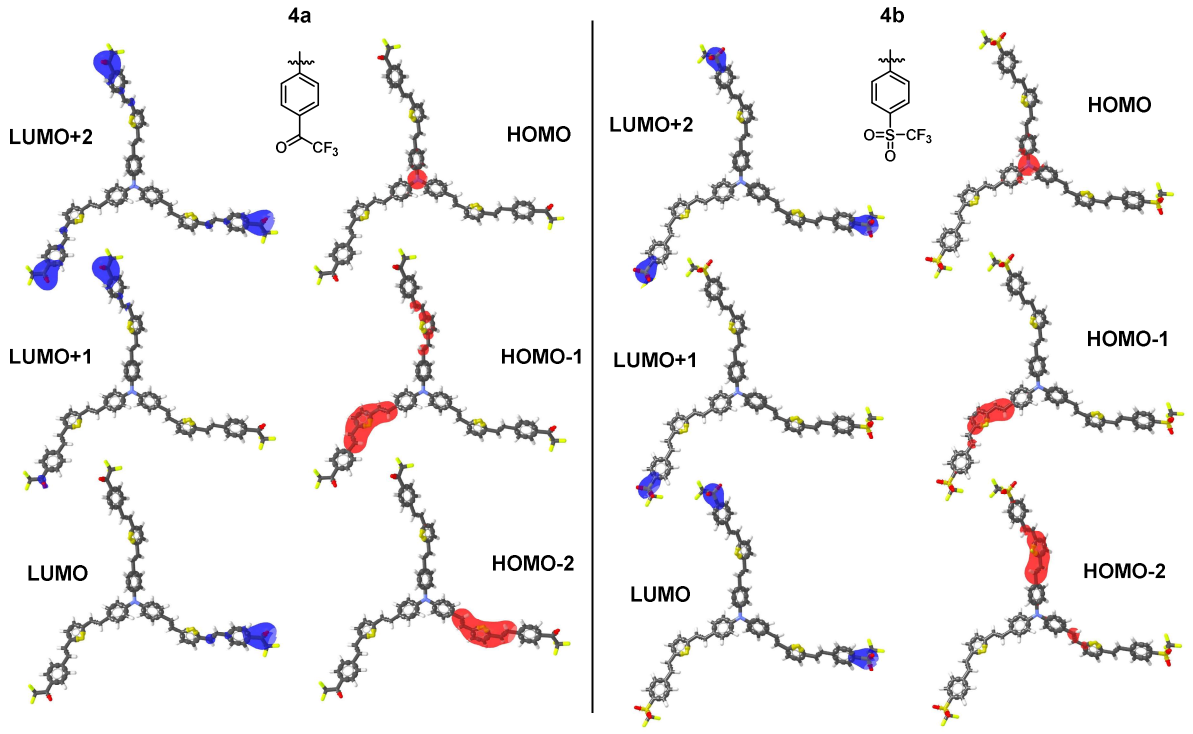

Figure S59. DFT visualization of the frontier molecular orbitals in fluorophores **4a**–**4b**. The HOMO(−1,−2) is shown in red, the LUMO(+1,+2) in blue.

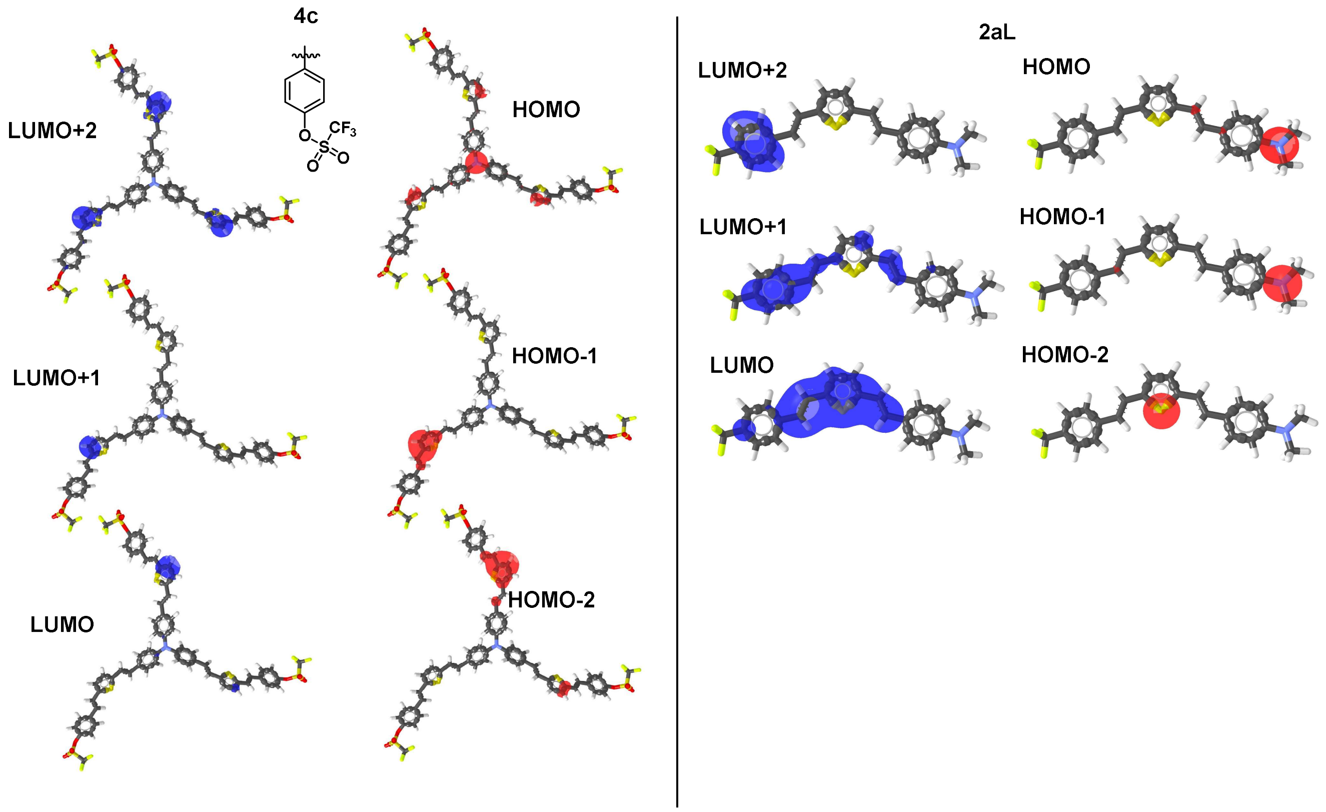

Figure S60. DFT visualization of the frontier molecular orbitals in fluorophores **4c** and **2aL**. The HOMO(−1,−2) is shown in red, the LUMO(+1,+2) in blue.

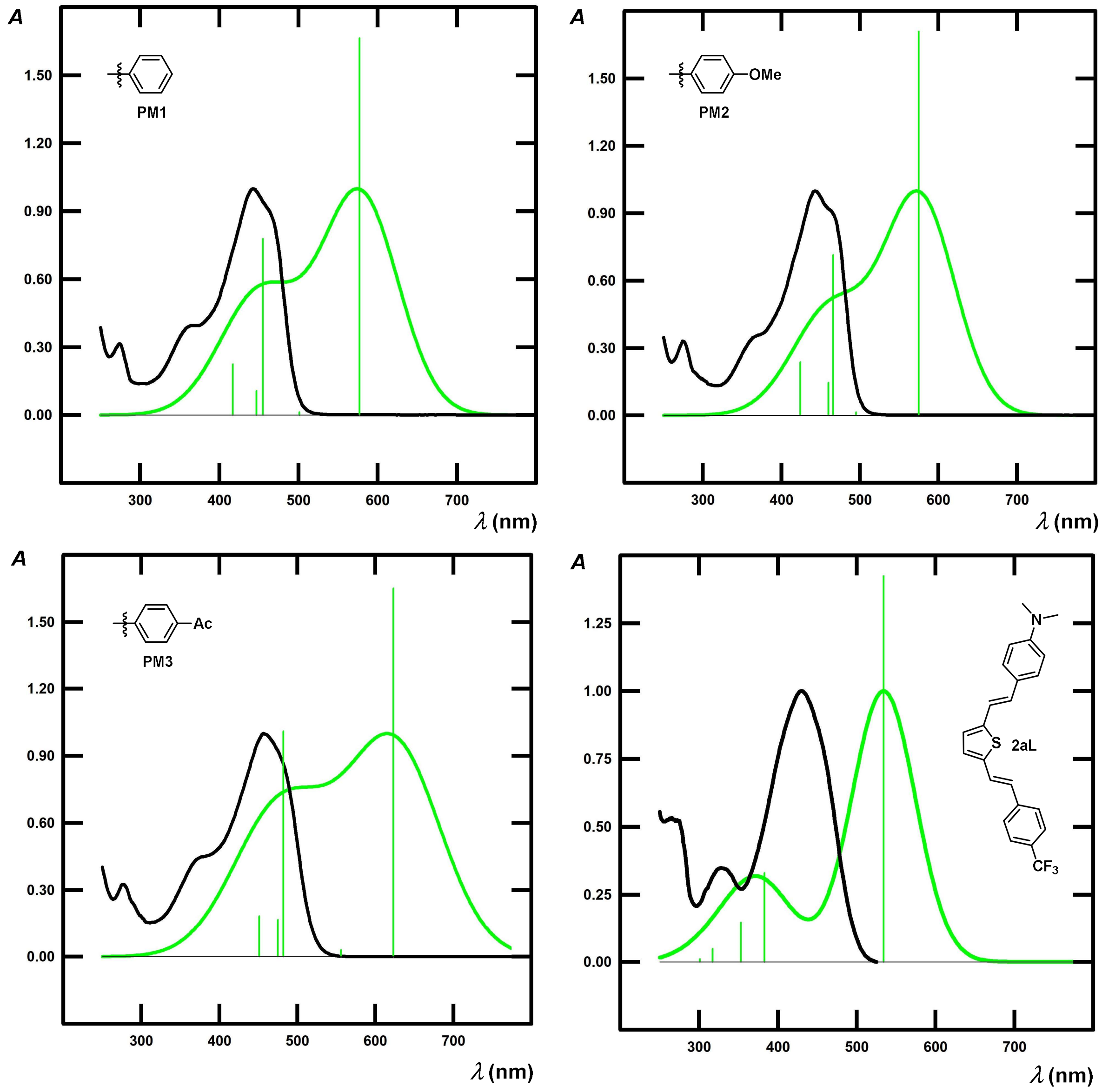

Figure S61. TD-DFT (nstates = 8) B3LYP/6-311++G(2d,p) calculated (green) UV-Vis spectra of fluorophores **PM1–3** and **2aL** in THF. Green vertical lines represent oscillator strengths (*f*). The black curves are experimentally obtained UV-Vis spectra in THF. Both spectra were overlapped and normalized to have maximal absorbance (*A*) of 1.

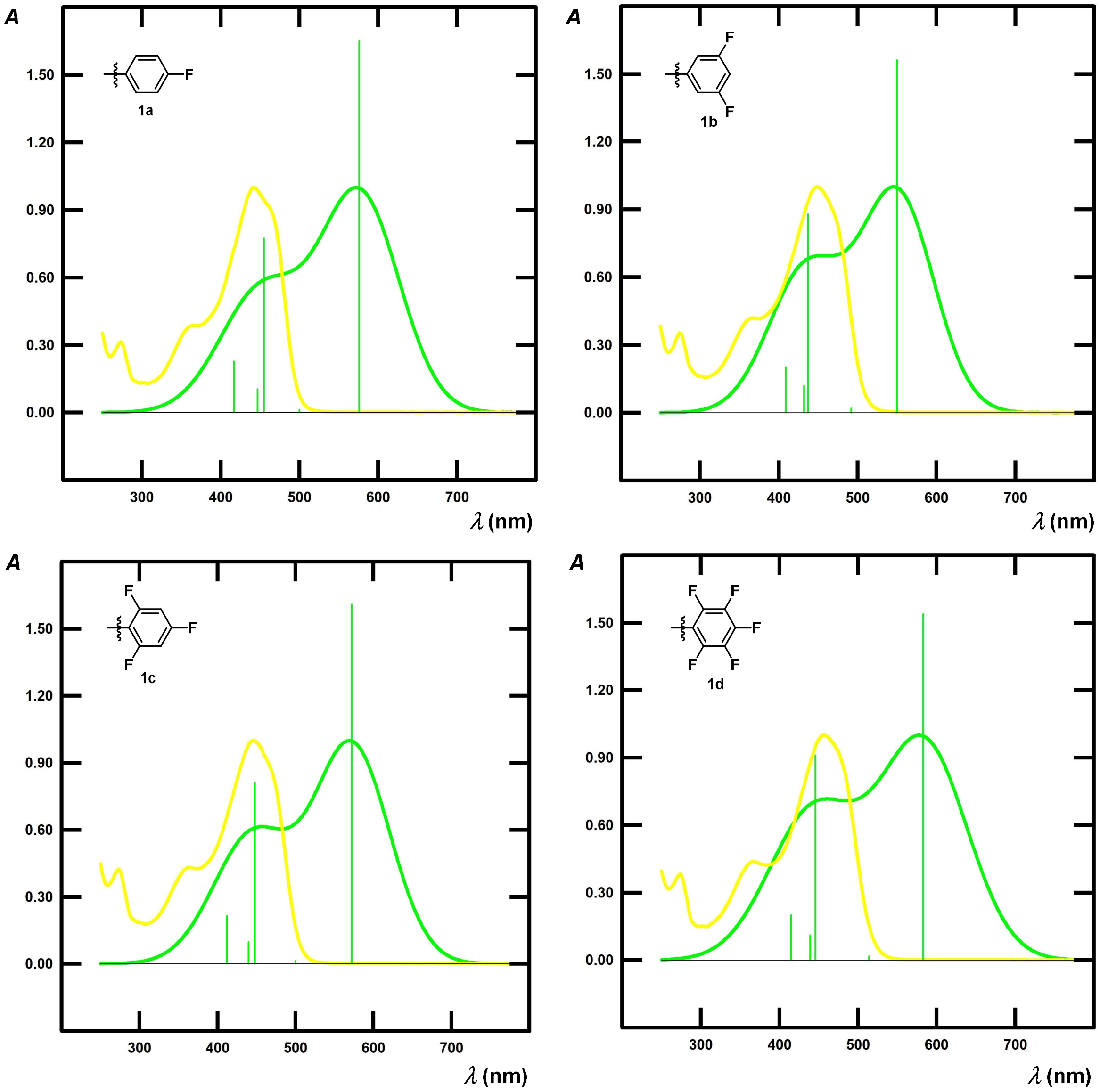

Figure S62. TD-DFT (nstates = 8) B3LYP/6-311++G(2d,p) calculated (green) UV-Vis spectra of fluorophores **1a–d** in THF. Green vertical lines represent oscillator strengths (*f*). The yellow curves are experimentally obtained UV-Vis spectra in THF. Both spectra were overlapped and normalized to have maximal absorbance (*A*) of 1.

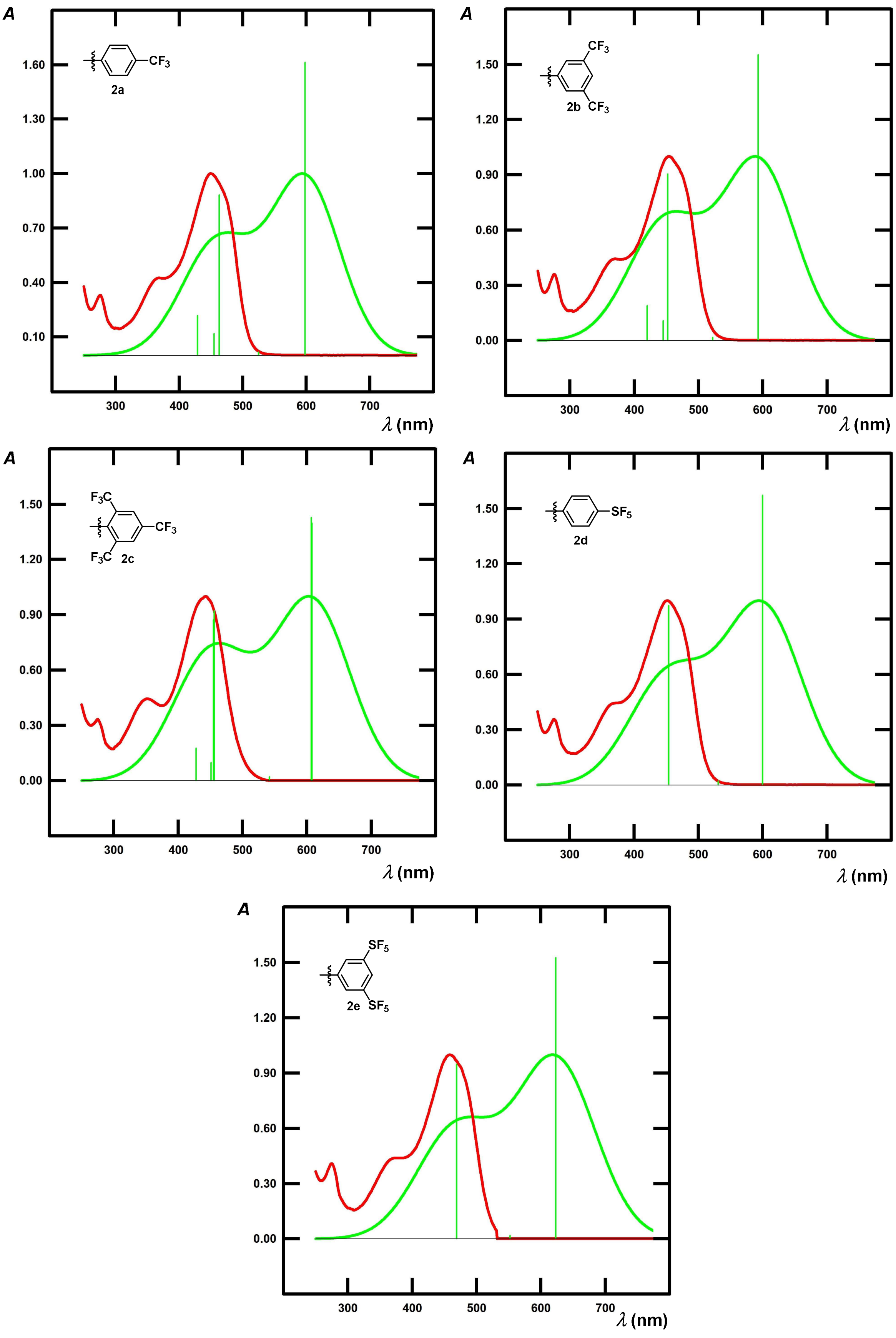

Figure S63. TD-DFT (nstates = 8) B3LYP/6-311++G(2d,p) calculated (green) UV-Vis spectra of fluorophores **2a–e** in THF. Green vertical lines represent oscillator strengths (*f*). The red curves are experimentally obtained UV-Vis spectra in THF. Both spectra were overlapped and normalized to have maximal absorbance (*A*) of 1.

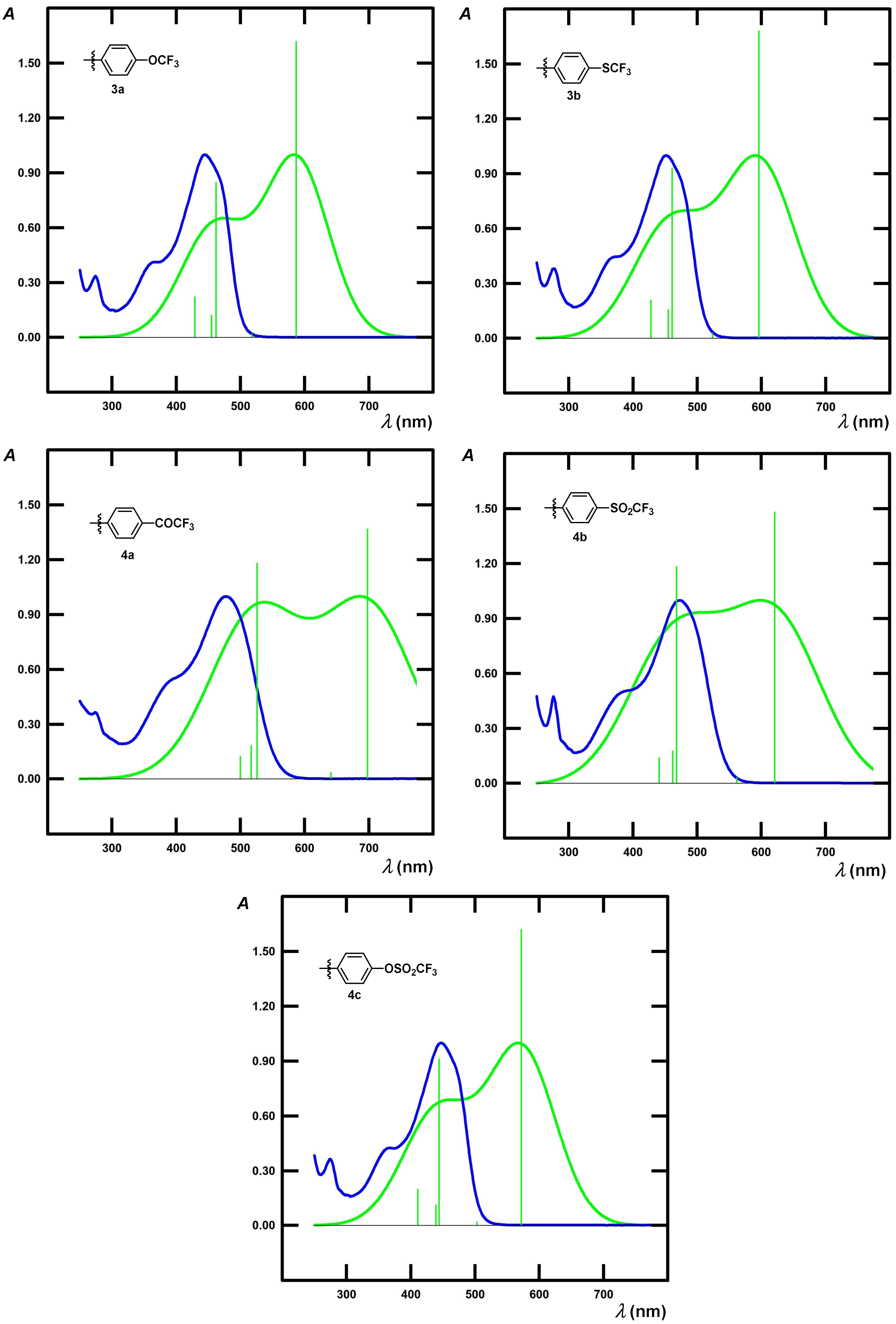

Figure S64. TD-DFT (nstates = 8) B3LYP/6-311++G(2d,p) calculated (green) UV-Vis spectra of fluorophores **3a**–**b** and **4a**–**c** in THF. Green vertical lines represent oscillator strengths (*f*). The blue curves are experimentally obtained UV-Vis spectra in THF. Both spectra were overlapped and normalized to have maximal absorbance (*A*) of 1.

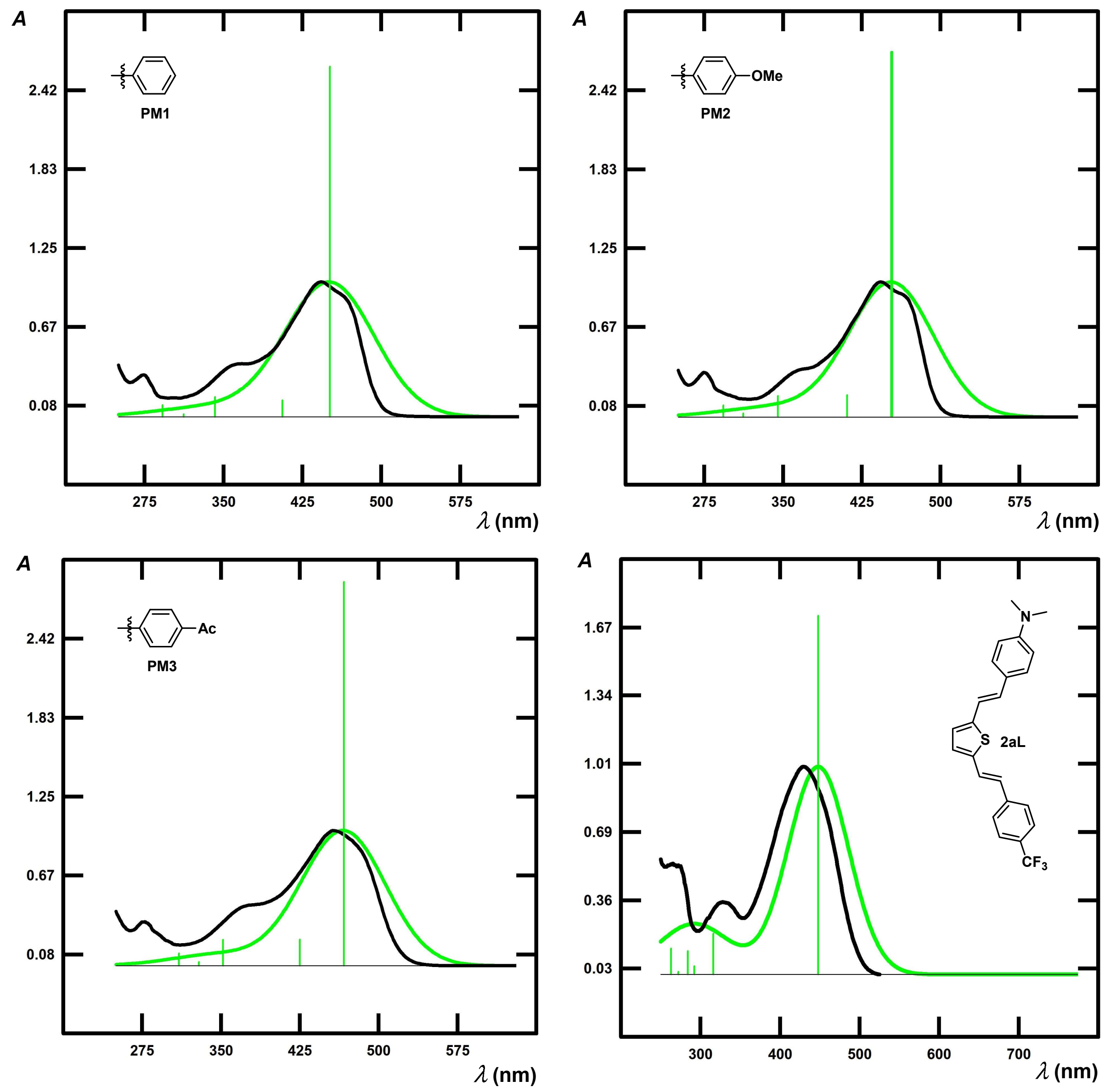

Figure S65. TD-DFT (nstates = 8) CAM-B3LYP/6-311++G(2d,p) calculated (green) UV-Vis spectra of fluorophores **PM1–3** in THF. Green vertical lines represent oscillator strengths (*f*). The black curves are experimentally obtained UV-Vis spectra in THF. Both spectra were overlapped and normalized to have maximal absorbance (*A*) of 1.

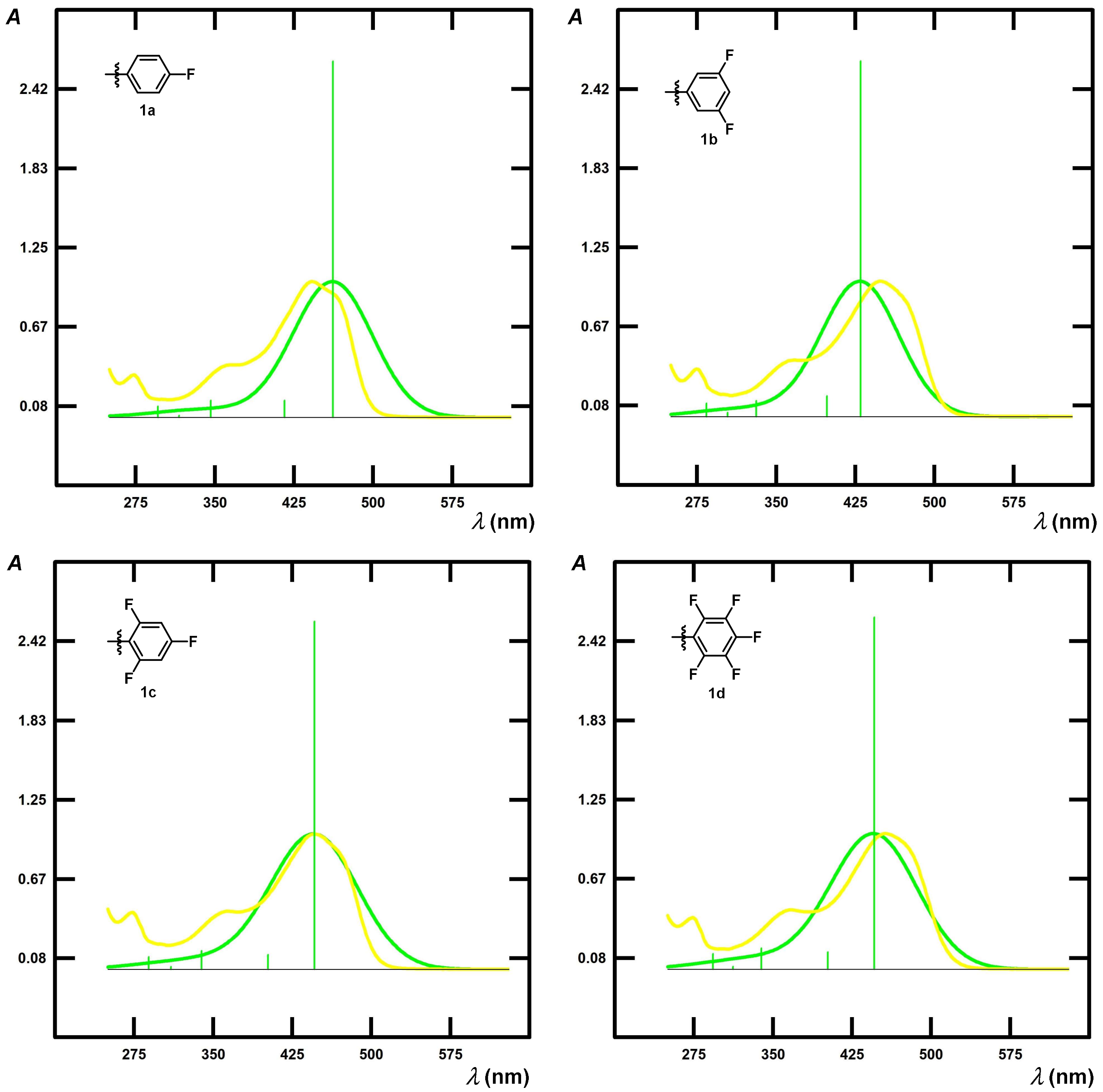

Figure S66. TD-DFT (nstates = 8) CAM-B3LYP/6-311++G(2d,p) calculated (green) UV-Vis spectra of fluorophores **1a–d** in THF. Green vertical lines represent oscillator strengths (*f*). The yellow curves are experimentally obtained UV-Vis spectra in THF. Both spectra were overlapped and normalized to have maximal absorbance (*A*) of 1.

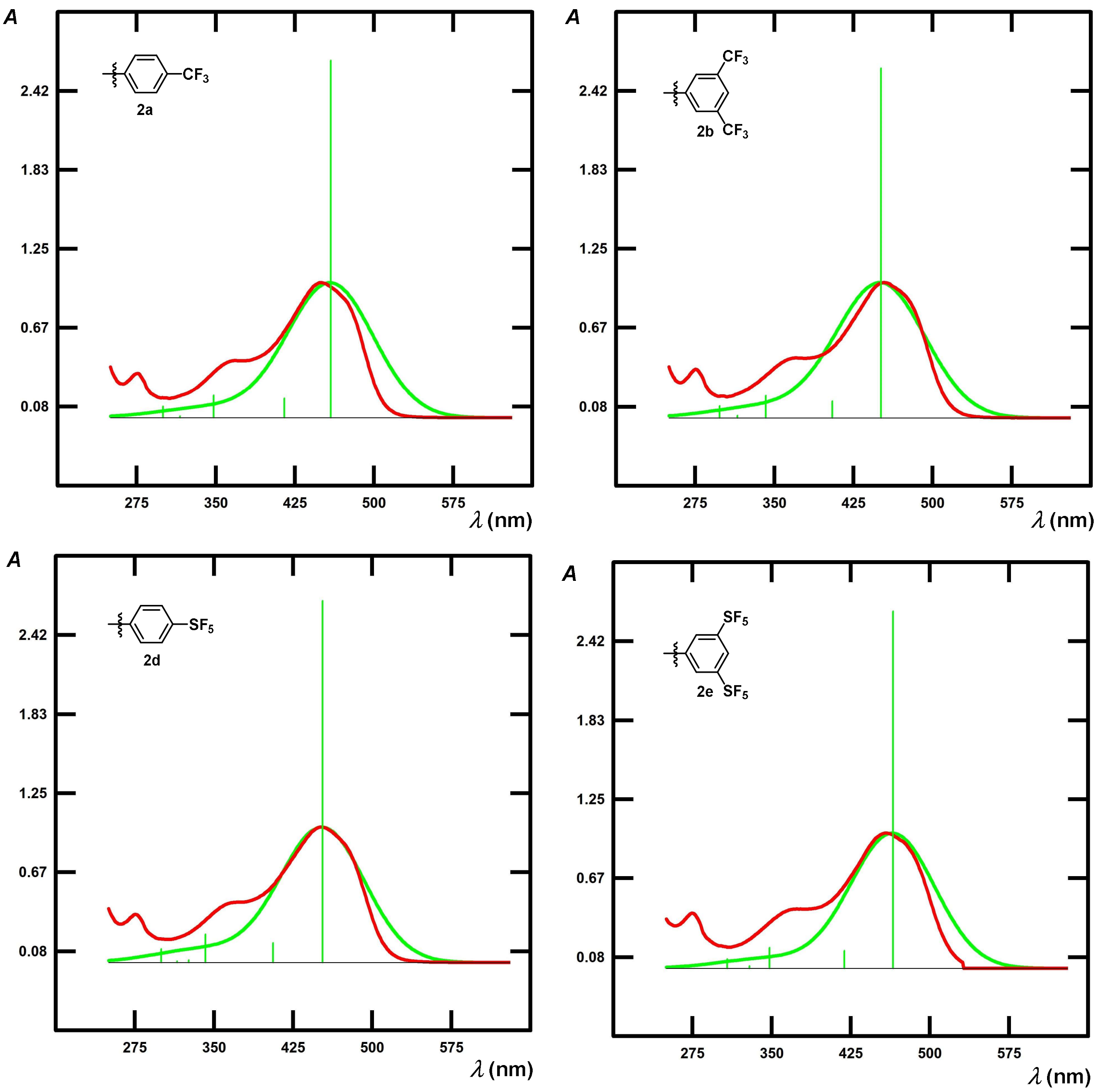

Figure S67. TD-DFT (nstates = 8) CAM-B3LYP/6-311++G(2d,p) calculated (green) UV-Vis spectra of fluorophores **2a**–**b** and **2d**–**e** in THF. Green vertical lines represent oscillator strengths (*f*). The red curves are experimentally obtained UV-Vis spectra in THF. Both spectra were overlapped and normalized to have maximal absorbance (*A*) of 1.

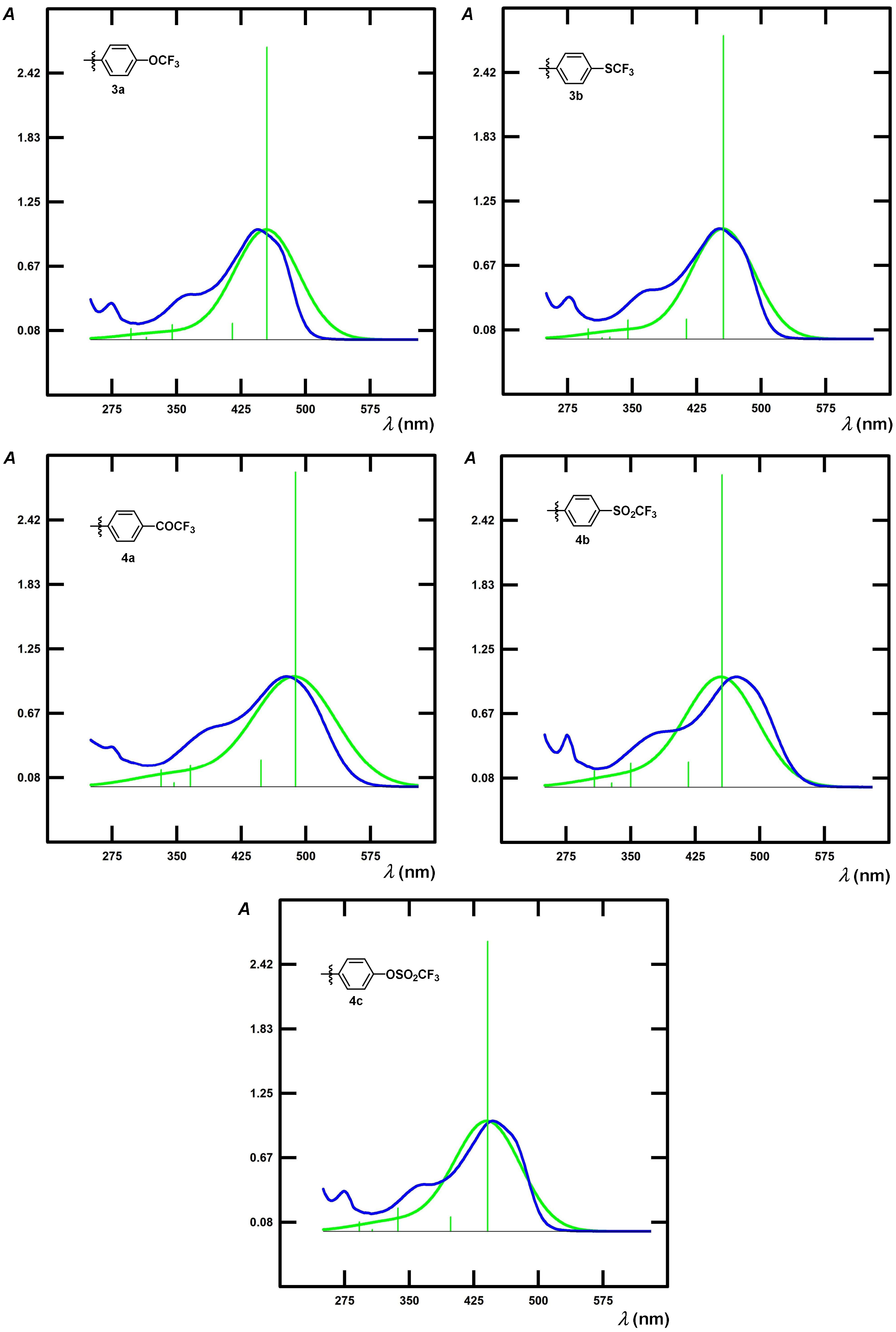

Figure S68. TD-DFT (nstates = 8) CAM-B3LYP/6-311++G(2d,p) calculated (green) UV-Vis spectra of fluorophores **3a–b** and **4a–c** in THF. Green vertical lines represent oscillator strengths (*f*). The blue curves are experimentally obtained UV-Vis spectra in THF. Both spectra were overlapped and normalized to have maximal absorbance (*A*) of 1.

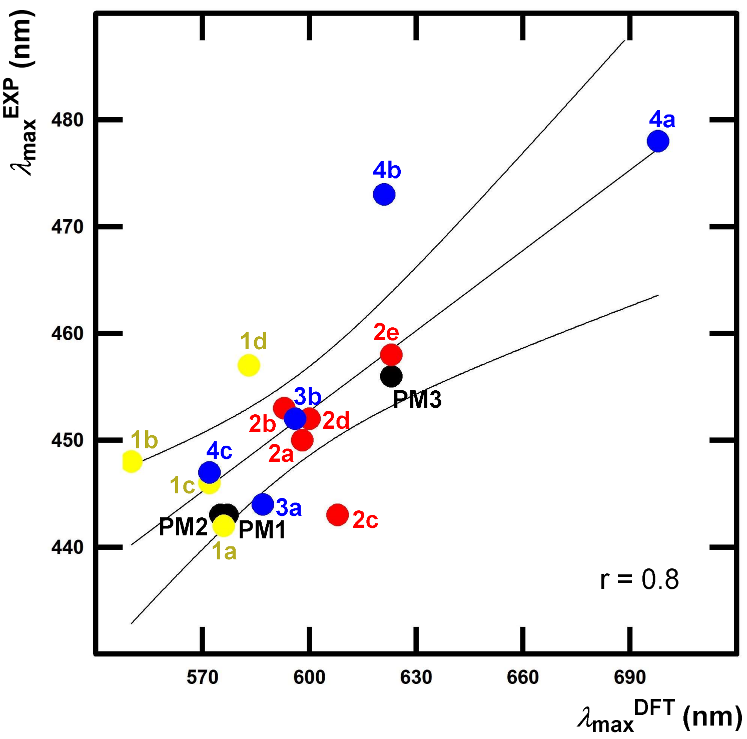

Figure S69. Correlation of the experimental absorption maxima λ_{max}^{EXP} and the calculated λ_{max}^{DFT} at TD-DFT B3LYP/6–311++G(2d,p) level (both in THF).

8. Non-linear absorption properties (2PA)

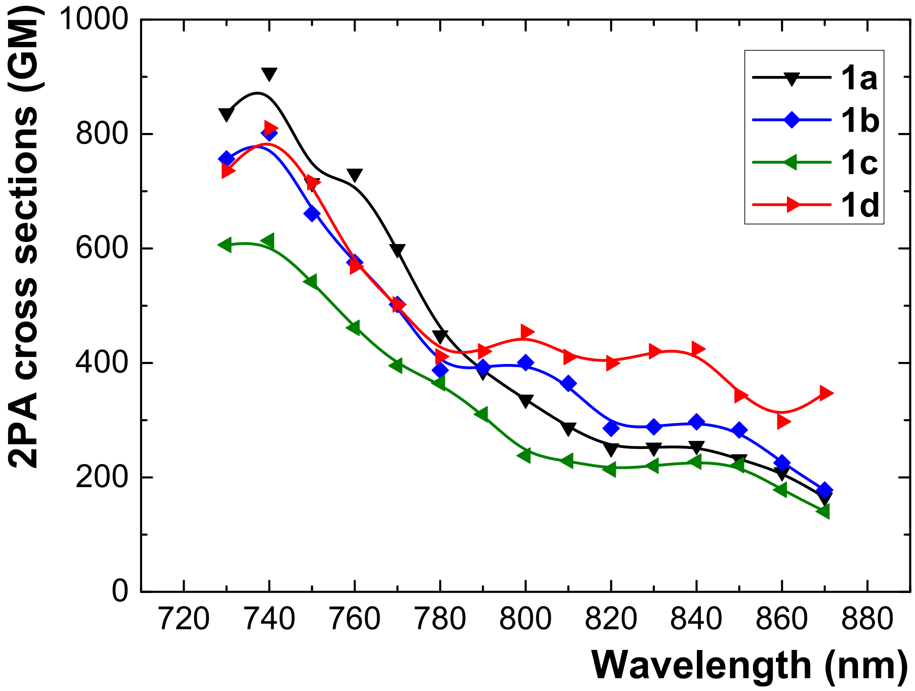

Figure S70. Two-photon absorption spectra for fluorophores **1a** (4-F)**, 1b** (3,5-F), **1c** (2,4,6-F)**, 1d** (2,3,4,5,6-F) measured in THF.

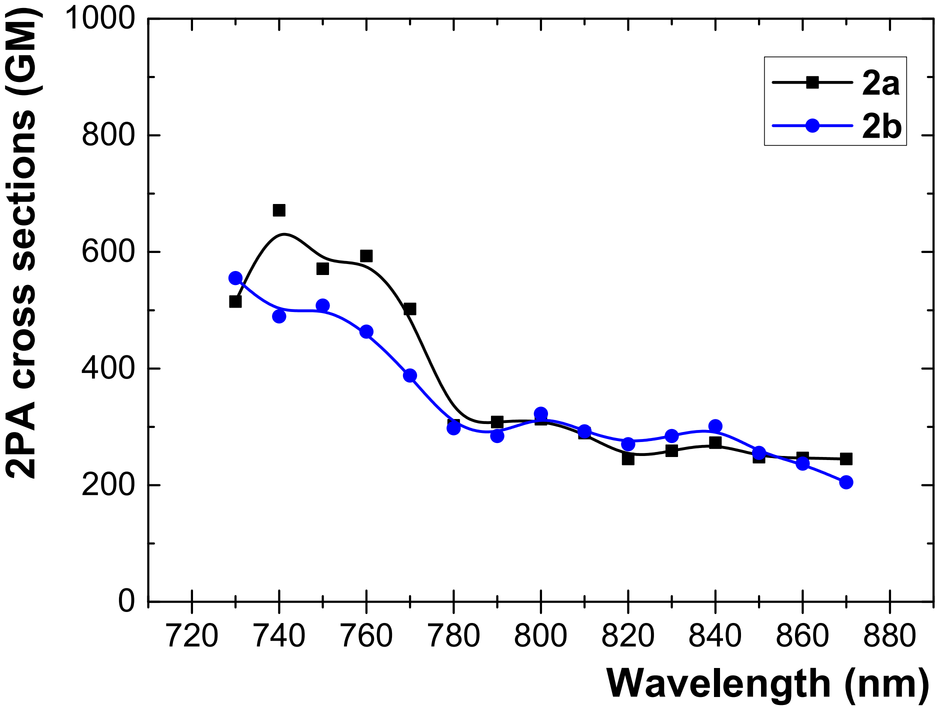

Figure S71. Two-photon absorption spectra for fluorophores **2a** (4-CF_3), **2b** (3,5-CF_3) measured in THF.

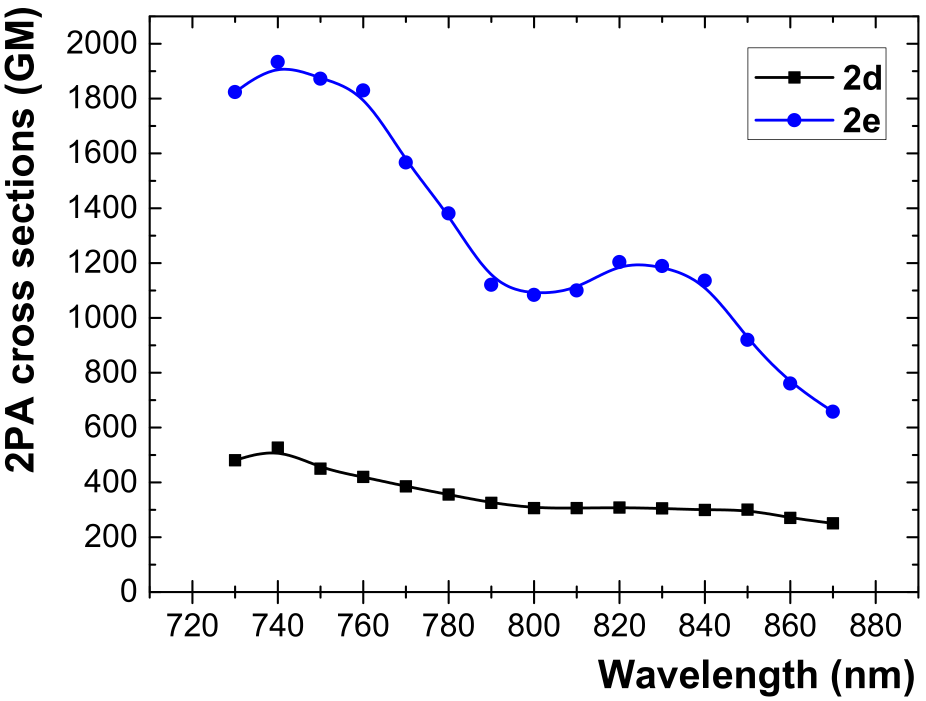

Figure S72. Two-photon absorption spectra for fluorophores **2d** (4-SF_5), **2e** (3,5-SF_5) measured in THF.

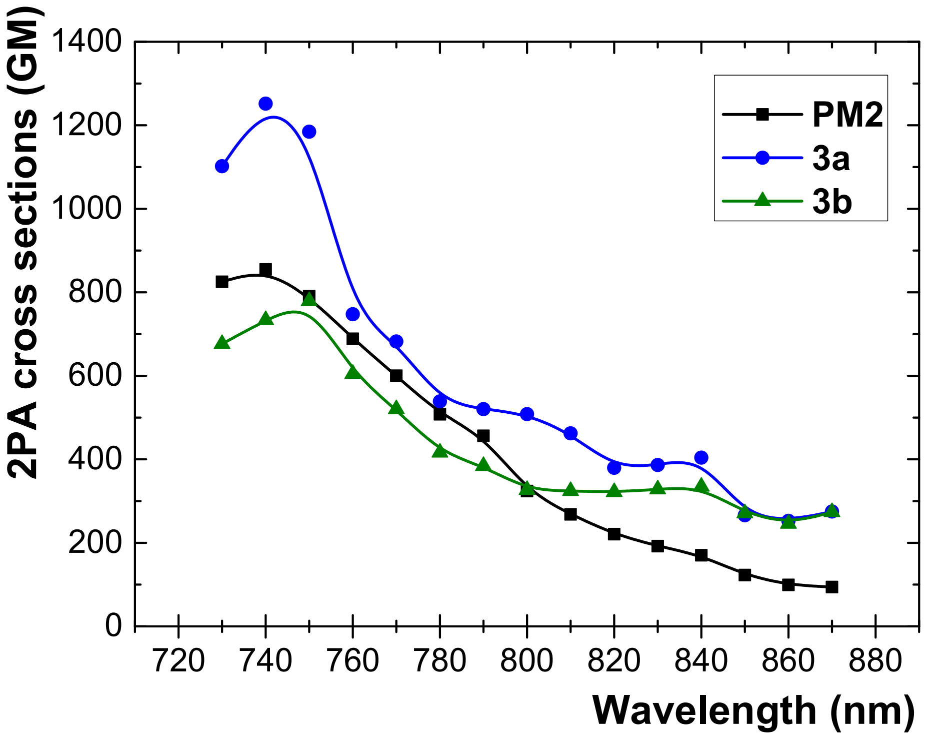

Figure S73. Two-photon absorption spectra for fluorophores **PM2** (4-OCH_3), **3a** (4-OCF_3), **3b** (4-SCF_3) measured in THF.

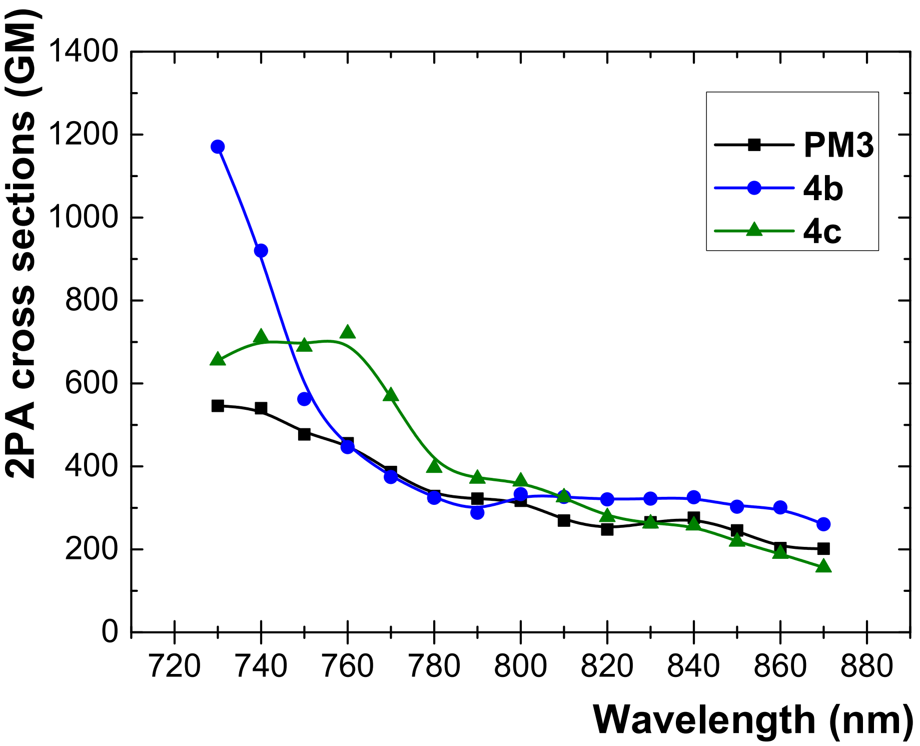

Figure S74. Two-photon absorption spectra for fluorophores **PM3** (4-$COCH_3$), **4b** (4-SO_2CF_3), **4c** (4-OSO_2CF_3) measured in THF.

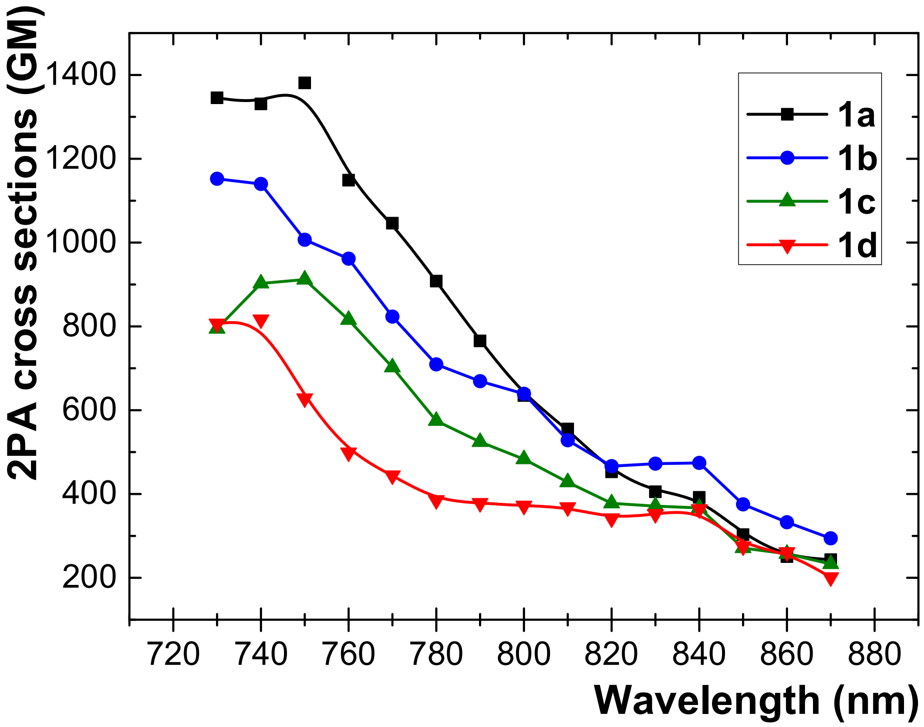

Figure S75. Two-photon absorption spectra for fluorophores **1a** (4-F), **1b** (3,5-F), **1c** (2,4,6-F), **1d** (2,3,4,5,6-F) measured in toluene.

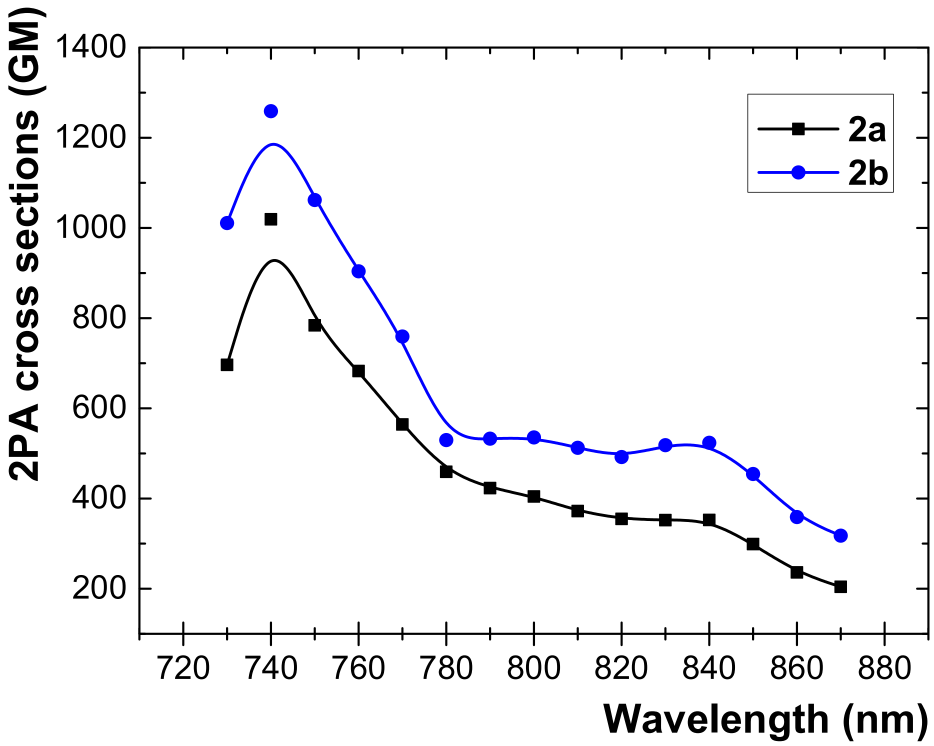

Figure S76. Two-photon absorption spectra for fluorophores **2a** (4-CF_3), **2b** (3,5-CF_3) measured in toluene.

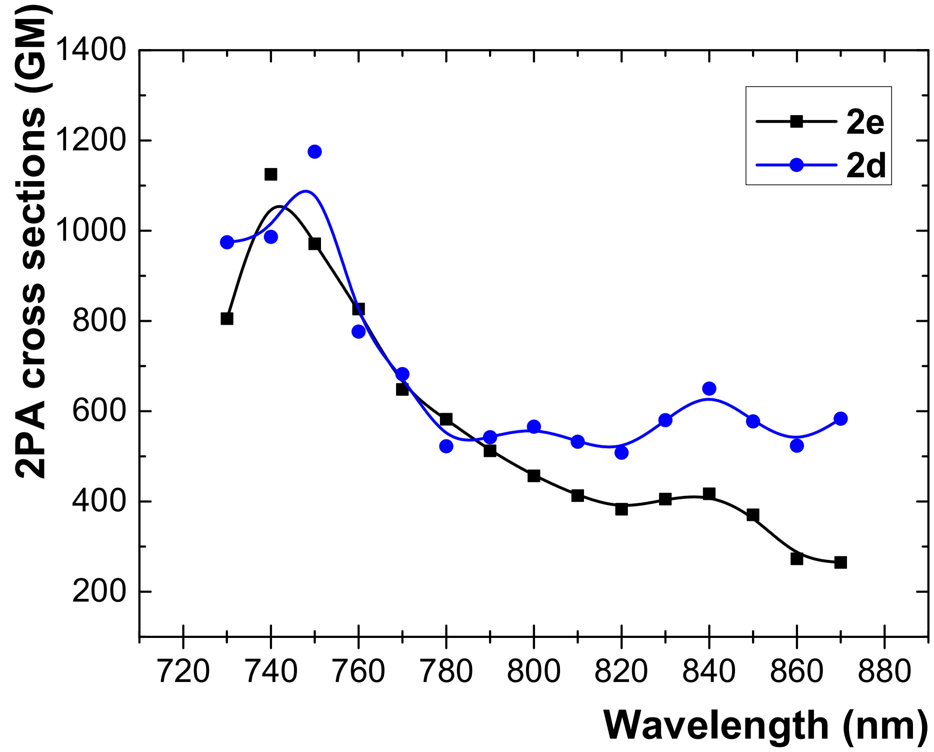

Figure S77. Two-photon absorption spectra for fluorophores **2d** (4-SF_5)**,** **2e** (3,5-SF_5) measured in toluene.

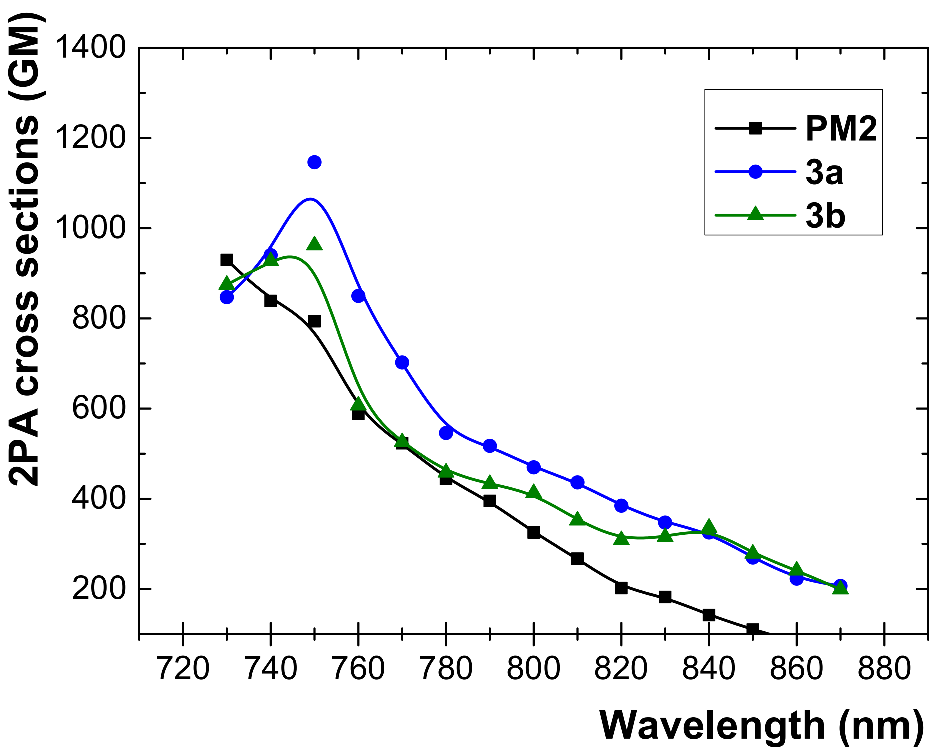

Figure S78. Two-photon absorption spectra for fluorophores **PM2** (4-OCH_3)**,** **3a** (4-OCF_3), **3b** (4-SCF_3) in toluene.

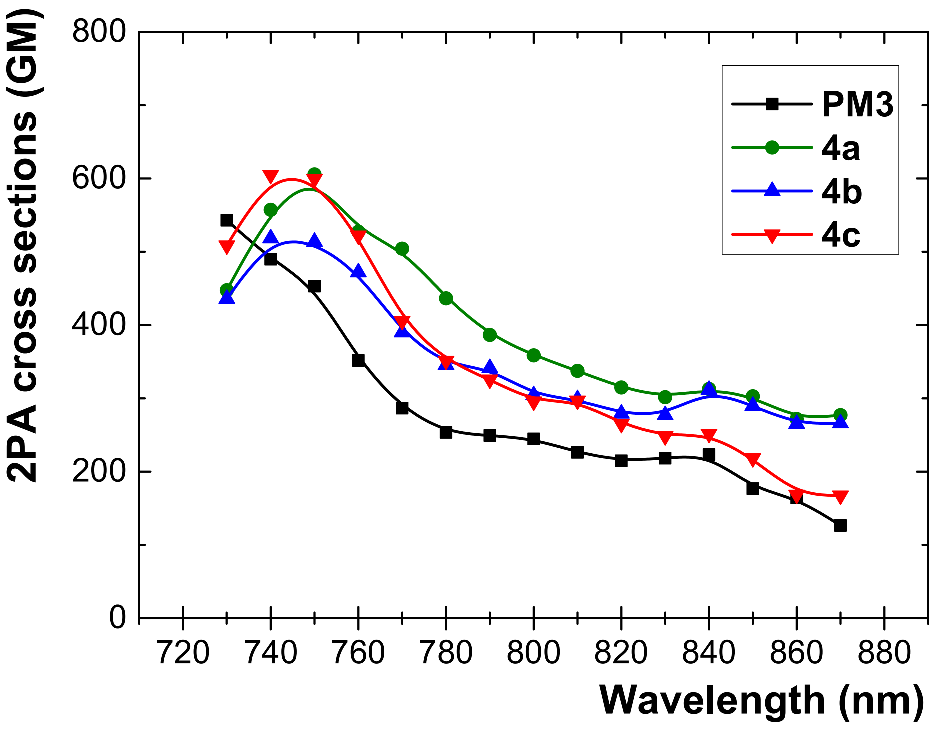

Figure S79. Two-photon absorption spectra for fluorophores **PM3** (4-$COCH_3$), **4b** (4-SO_2CF_3), **4c** (4-OSO_2CF_3) in toluene.

9. ^{1}H, ^{13}C and ^{19}F NMR spectra

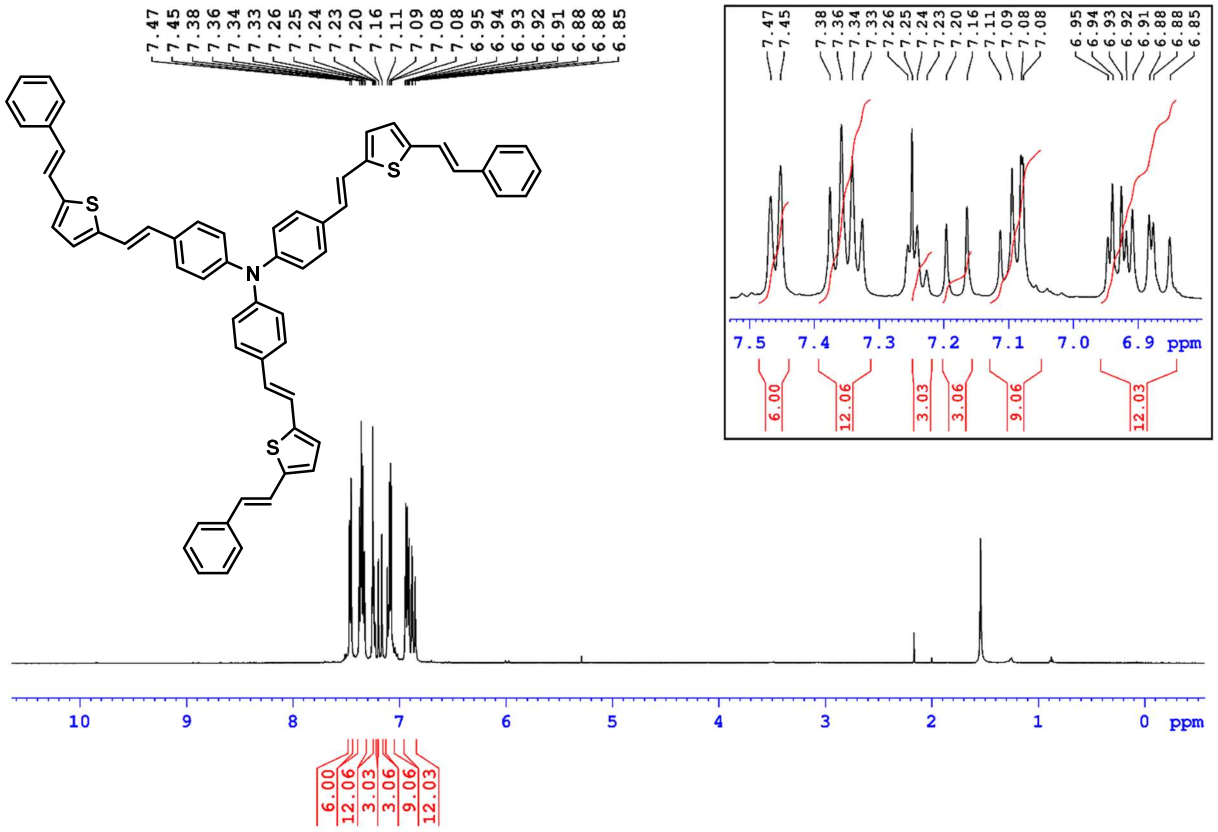

Figure S80. ^{1}H-NMR spectrum of fluorophore **PM1** (500 MHz, $CDCl_3$, 25 °C).

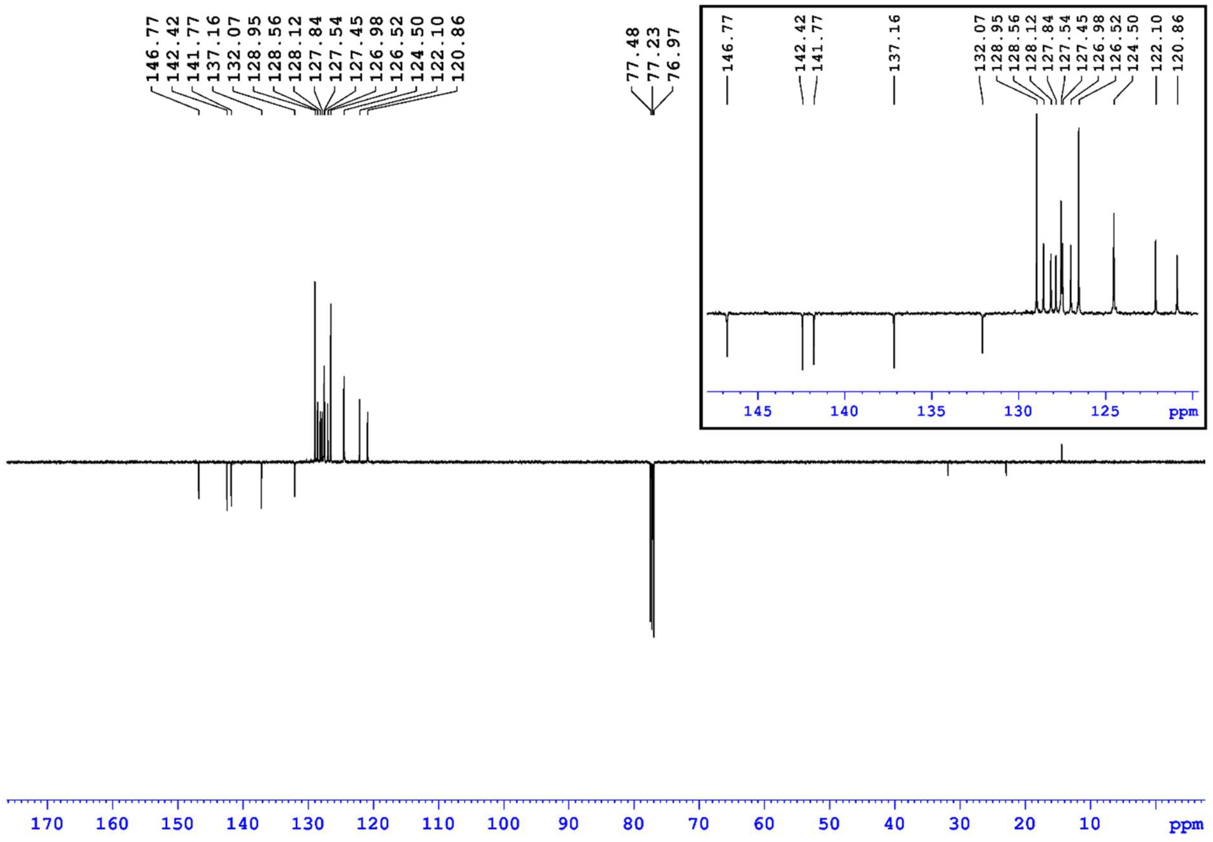

Figure S81. ^{13}C-NMR APT spectrum of fluorophore **PM1** (125 MHz, $CDCl_3$, 25 °C).

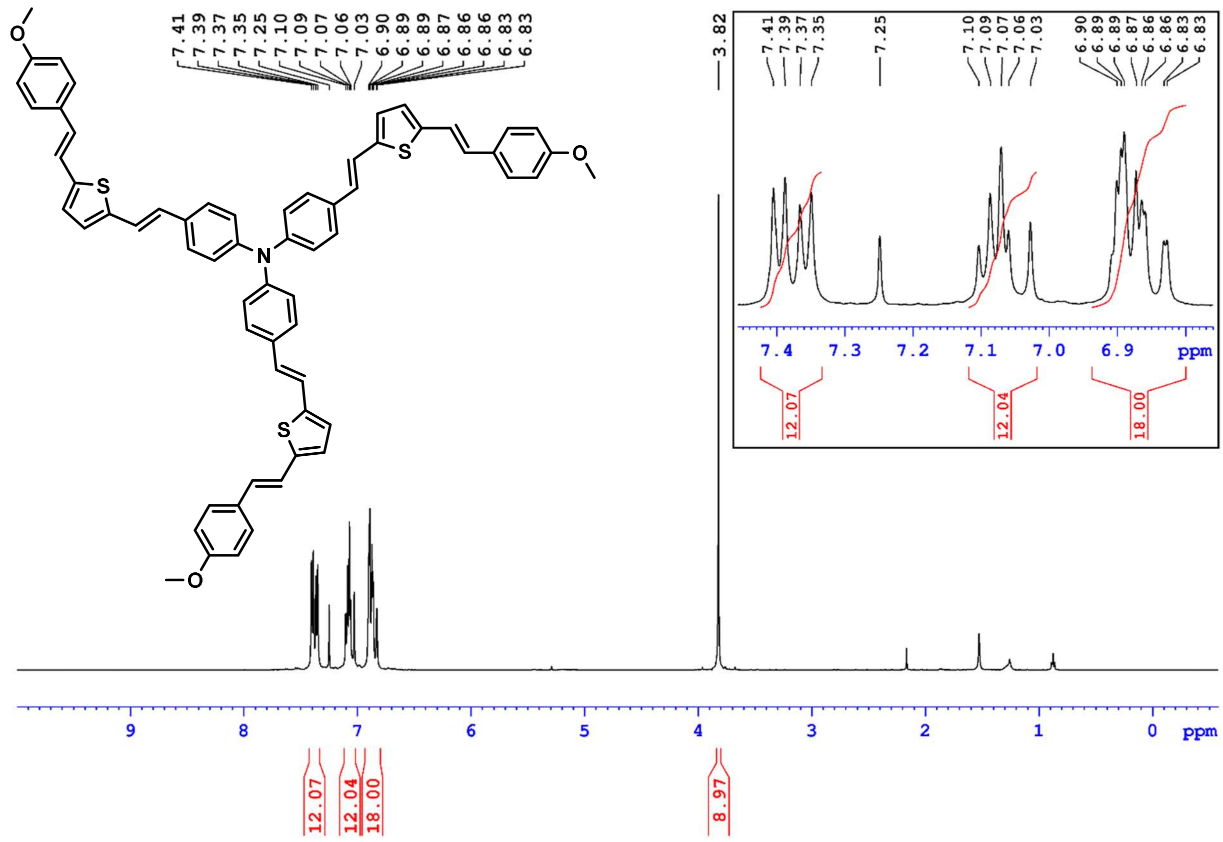

Figure S82. ^{1}H-NMR spectrum of fluorophore **PM2** (500 MHz, $CDCl_3$, 25 °C).

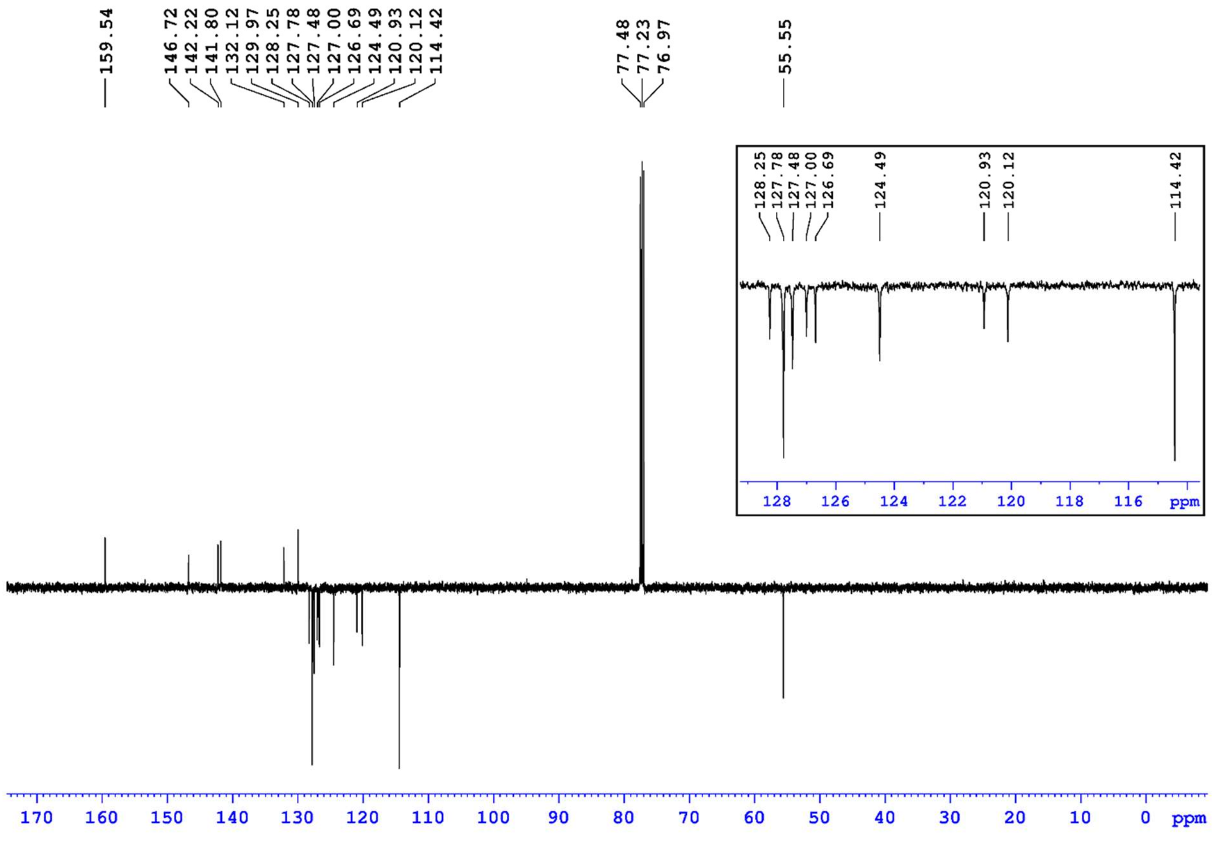

Figure S83. ^{13}C-NMR APT spectrum of fluorophore **PM2** (125 MHz, $CDCl_3$, 25 °C).

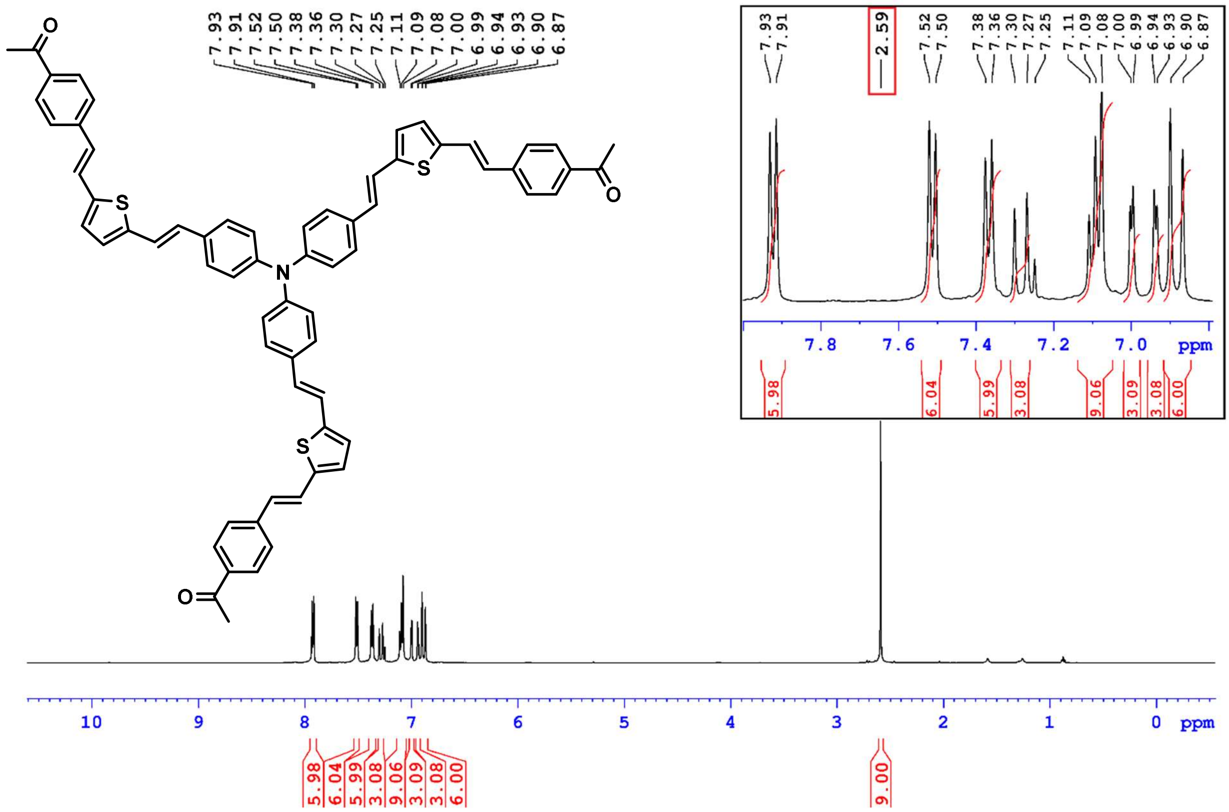

Figure S84. ^{1}H-NMR spectrum of fluorophore **PM3** (500 MHz, $CDCl_3$, 25 °C).

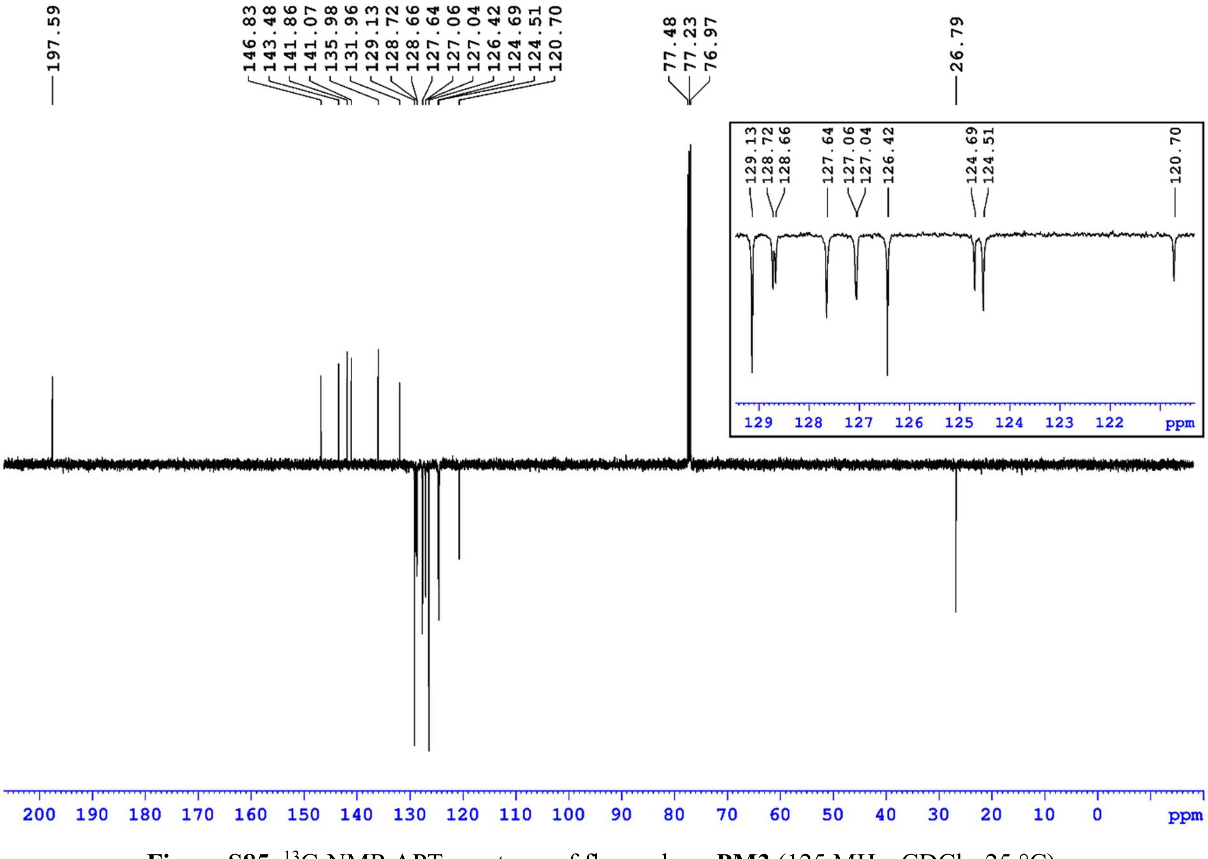

Figure S85. ^{13}C-NMR APT spectrum of fluorophore **PM3** (125 MHz, $CDCl_3$, 25 °C).

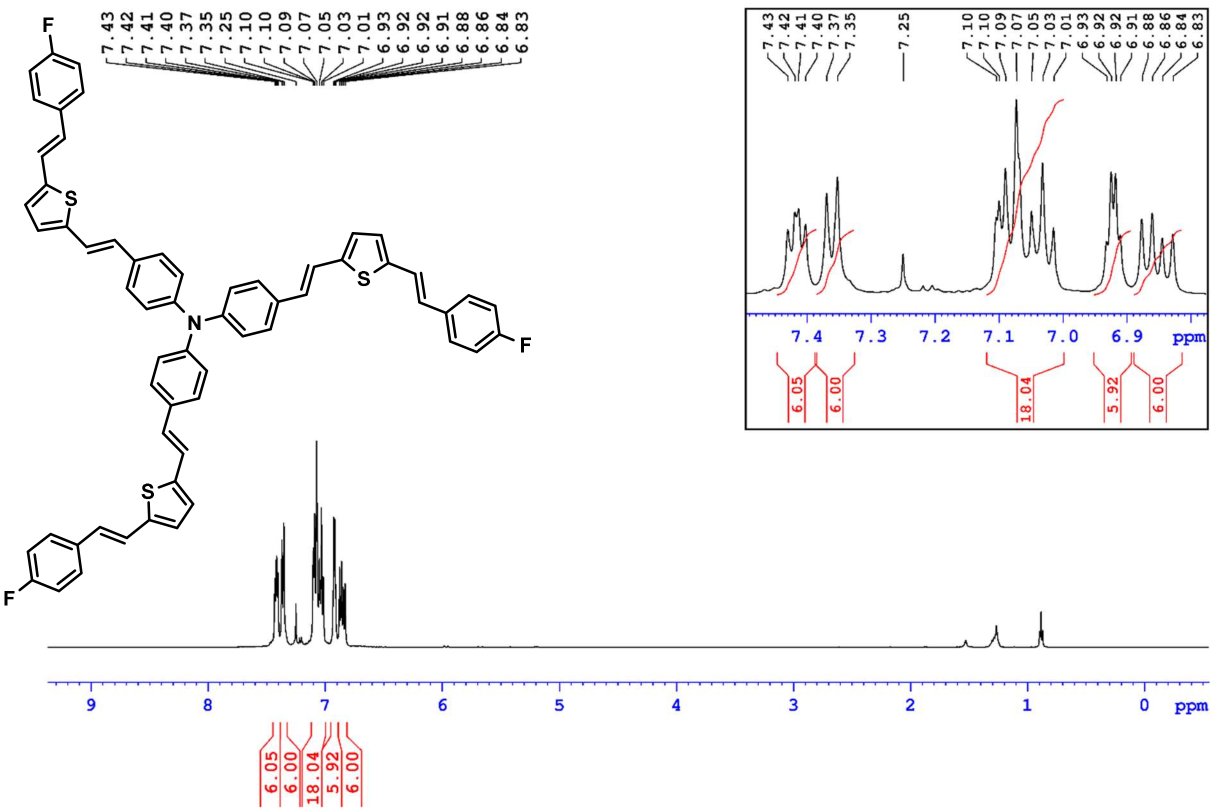

Figure S86. ^{1}H-NMR spectrum of fluorophore **1a** (500 MHz, $CDCl_3$, 25 °C).

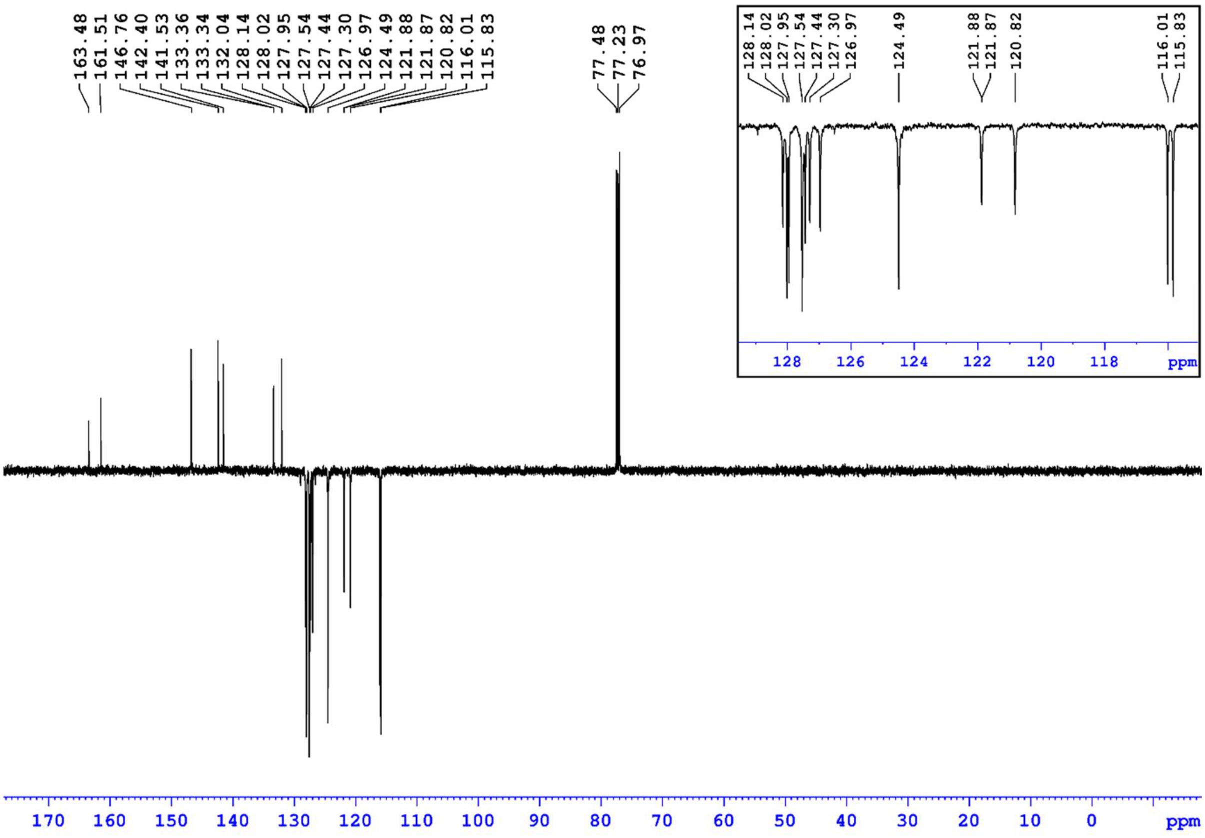

Figure S87. ^{13}C-NMR APT spectrum of fluorophore **1a** (125 MHz, $CDCl_3$, 25 °C).

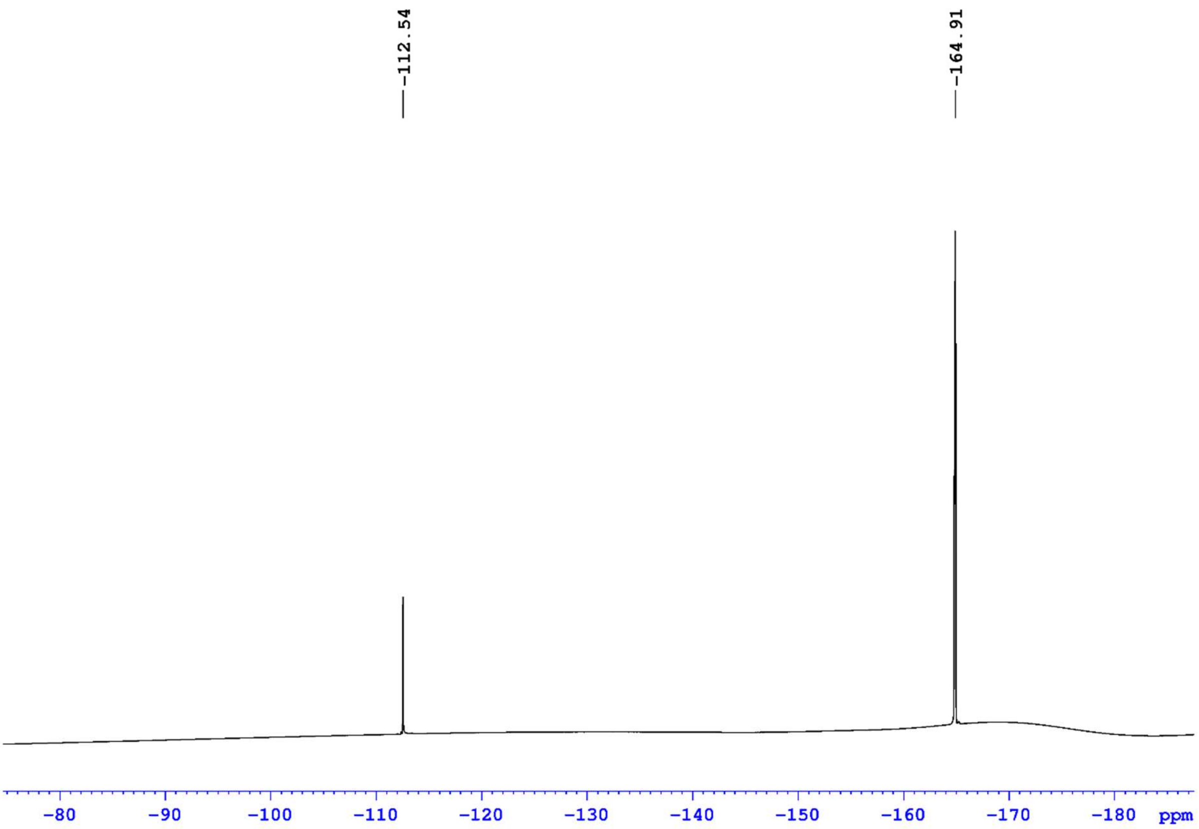

Figure S88. ^{19}F-NMR spectrum of fluorophore **1a** (470 MHz, $CDCl_3$, 25 °C).

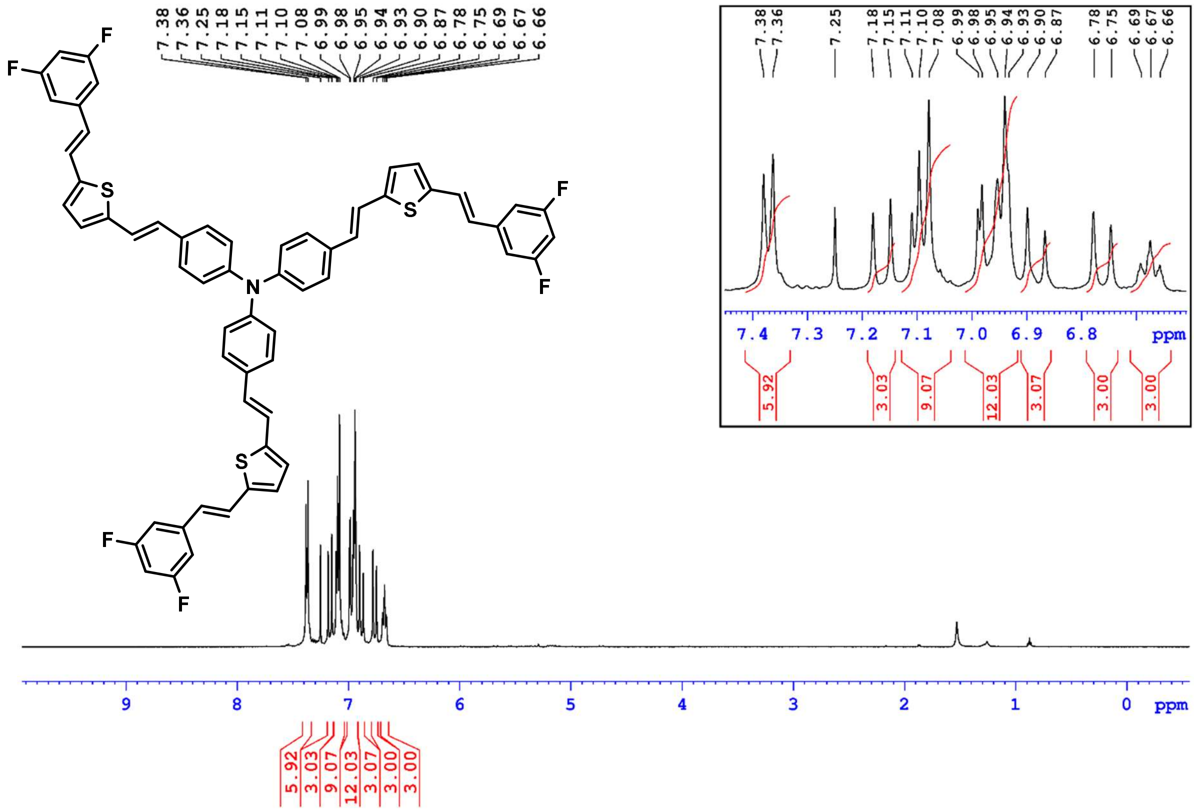

Figure S89. ^{1}H-NMR spectrum of fluorophore **1b** (500 MHz, $CDCl_3$, 25 °C).

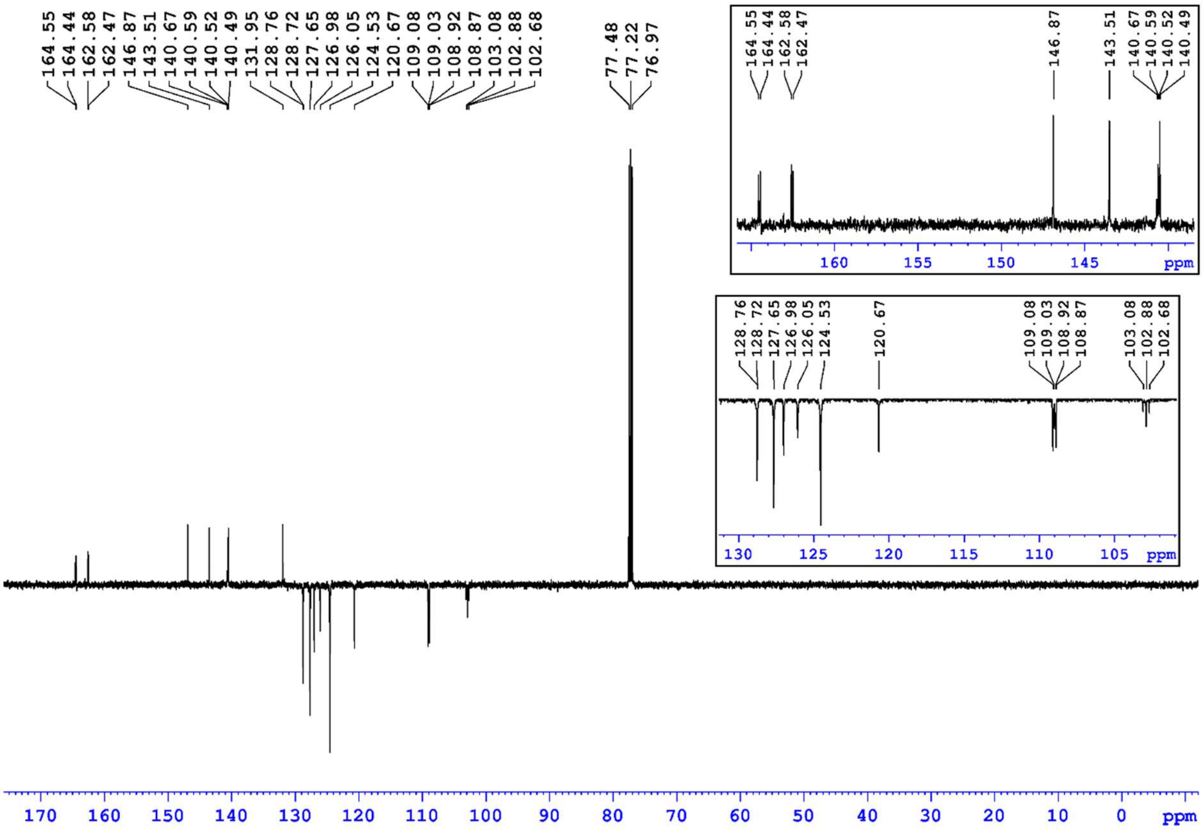

Figure S90. ^{13}C-NMR APT spectrum of fluorophore **1b** (125 MHz, $CDCl_3$, 25 °C).

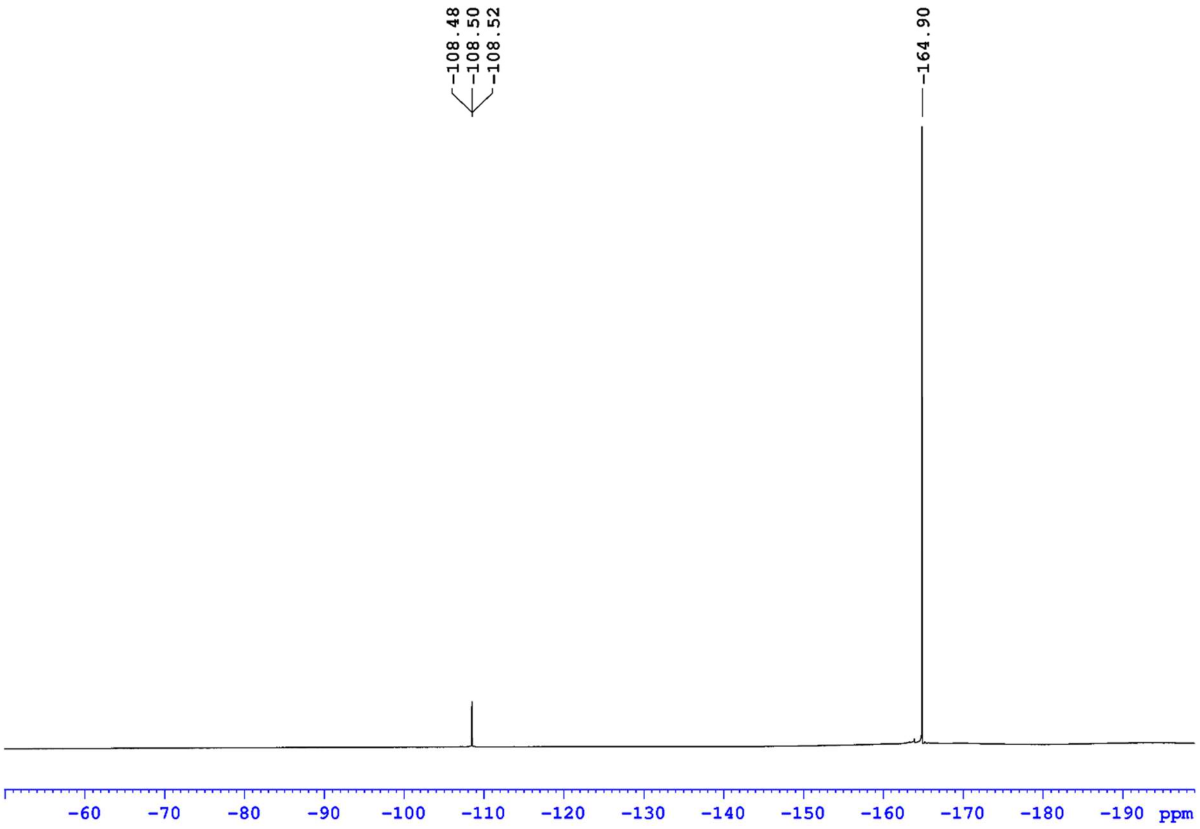

Figure S91. ^{19}F-NMR spectrum of fluorophore **1b** (470 MHz, $CDCl_3$, 25 °C).

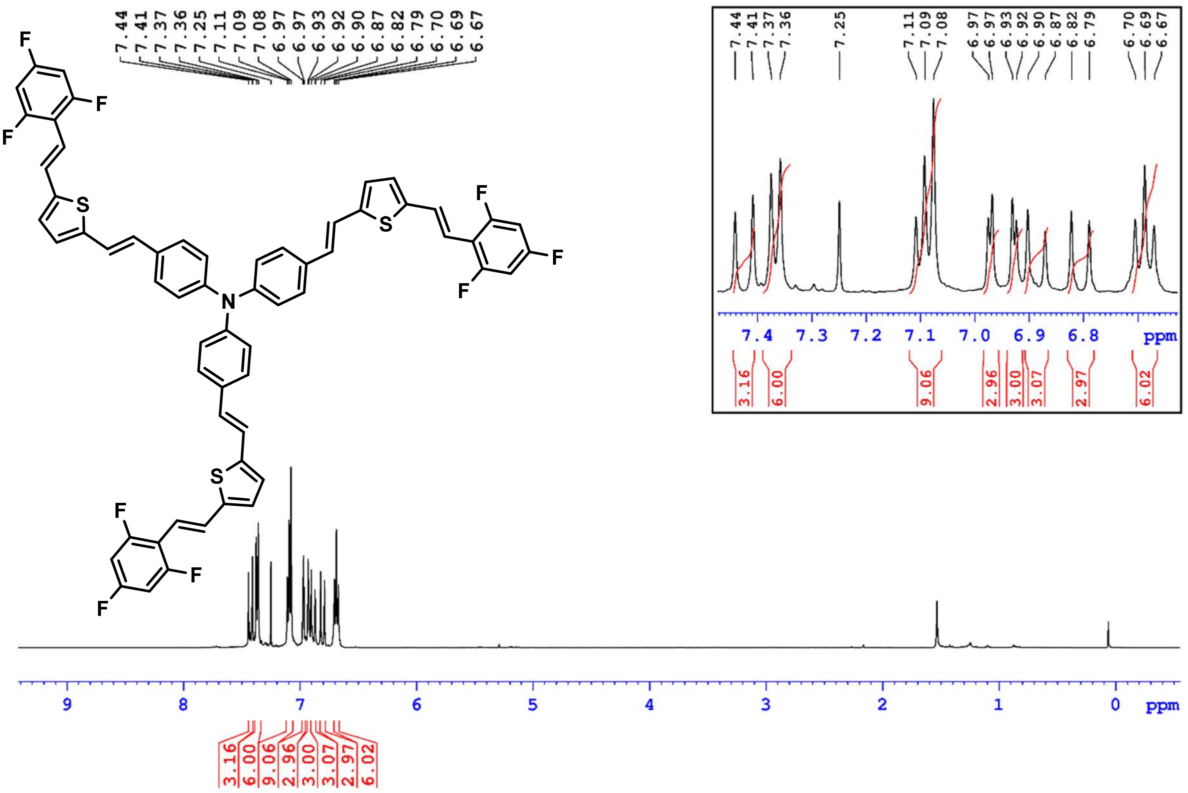

Figure S92. ^{1}H-NMR spectrum of fluorophore **1c** (500 MHz, $CDCl_3$, 25 °C).

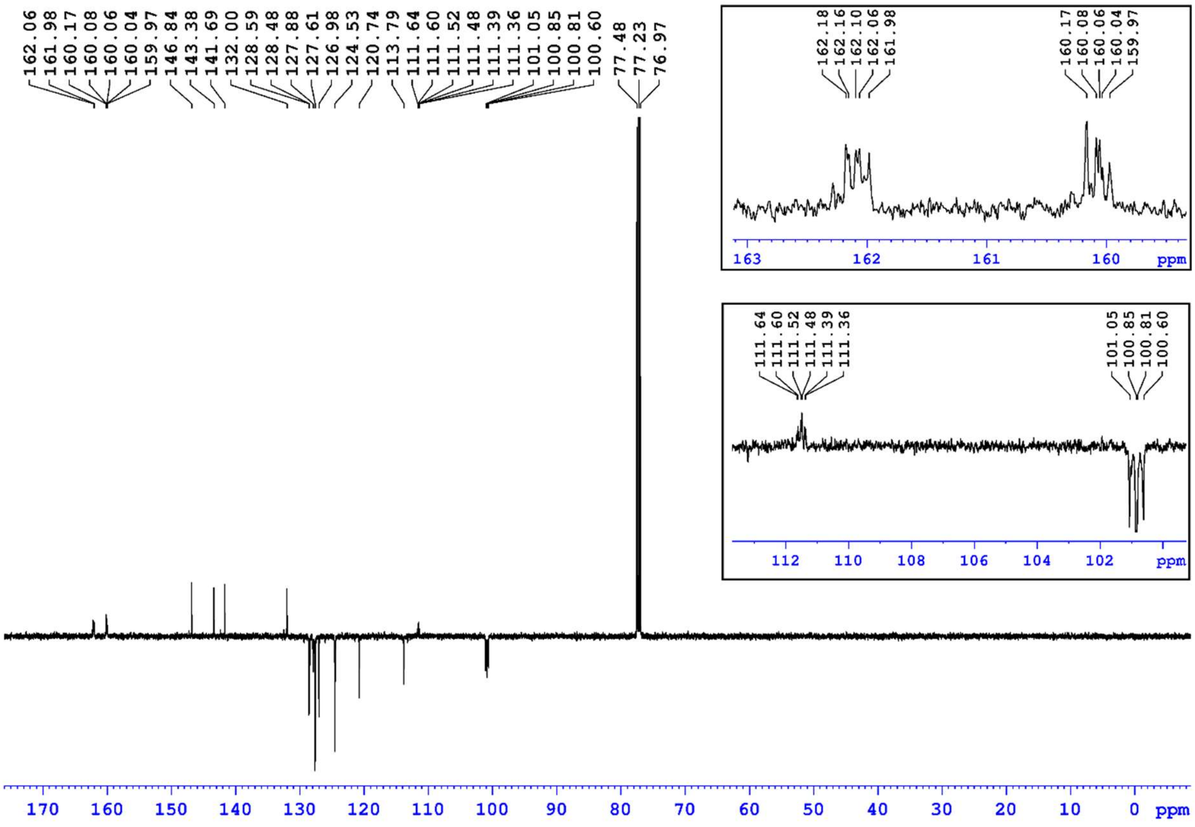

Figure S93. ^{13}C-NMR APT spectrum of fluorophore **1c** (125 MHz, $CDCl_3$, 25 °C).

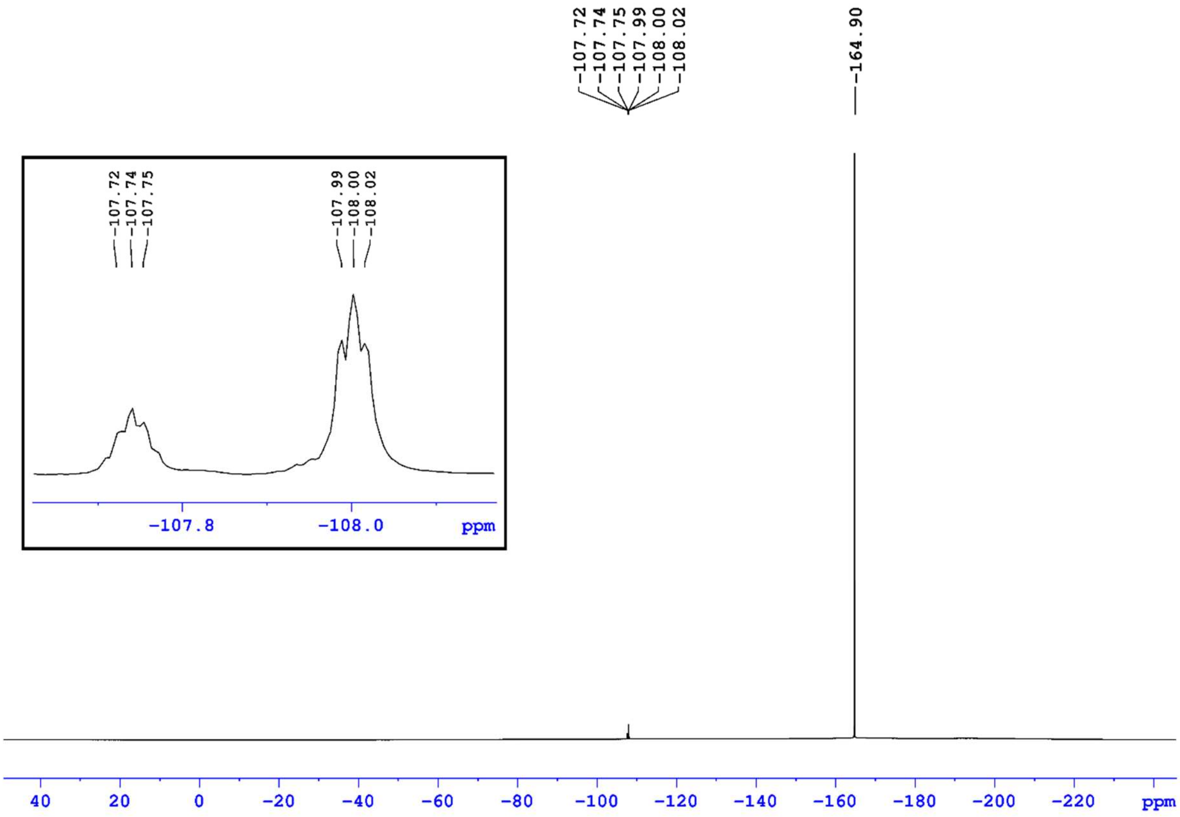

Figure S94. ^{19}F-NMR spectrum of fluorophore **1c** (470 MHz, $CDCl_3$, 25 °C).

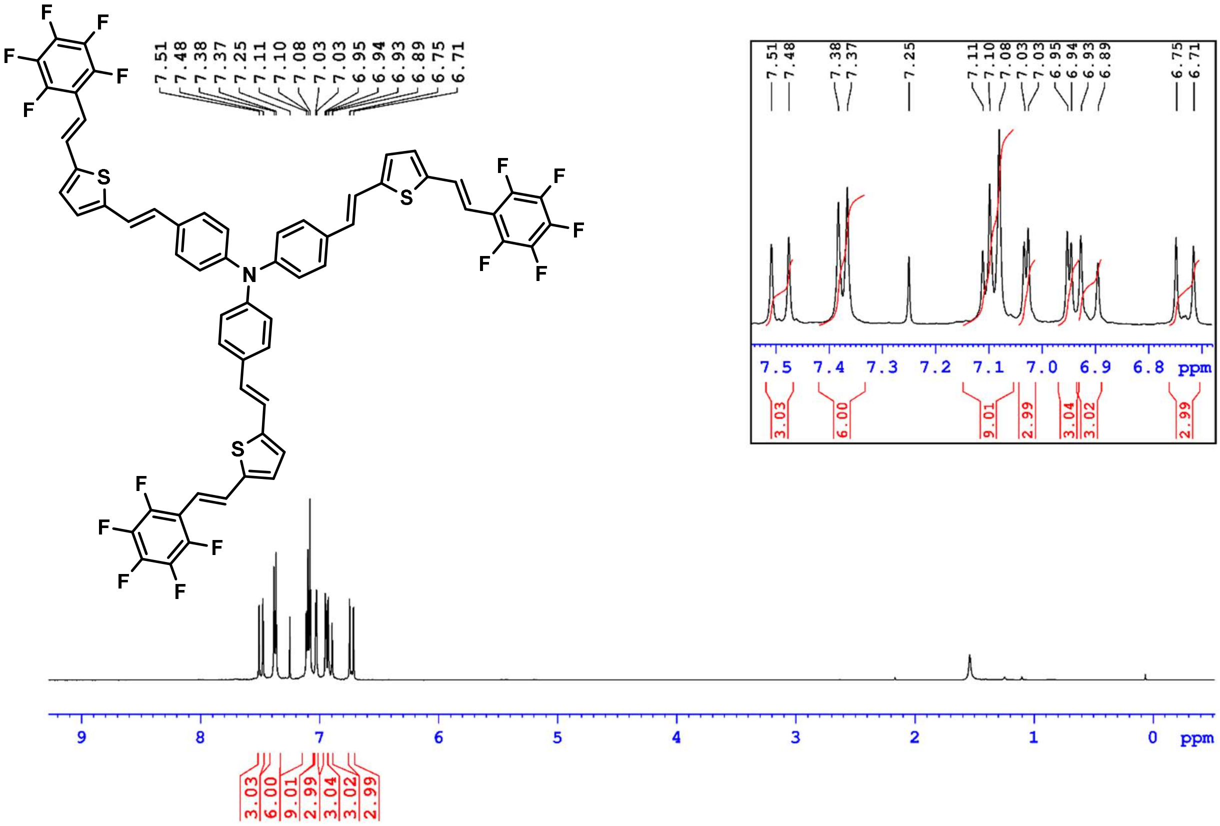

Figure S95. ^{1}H-NMR spectrum of fluorophore **1d** (500 MHz, $CDCl_3$, 25 °C).

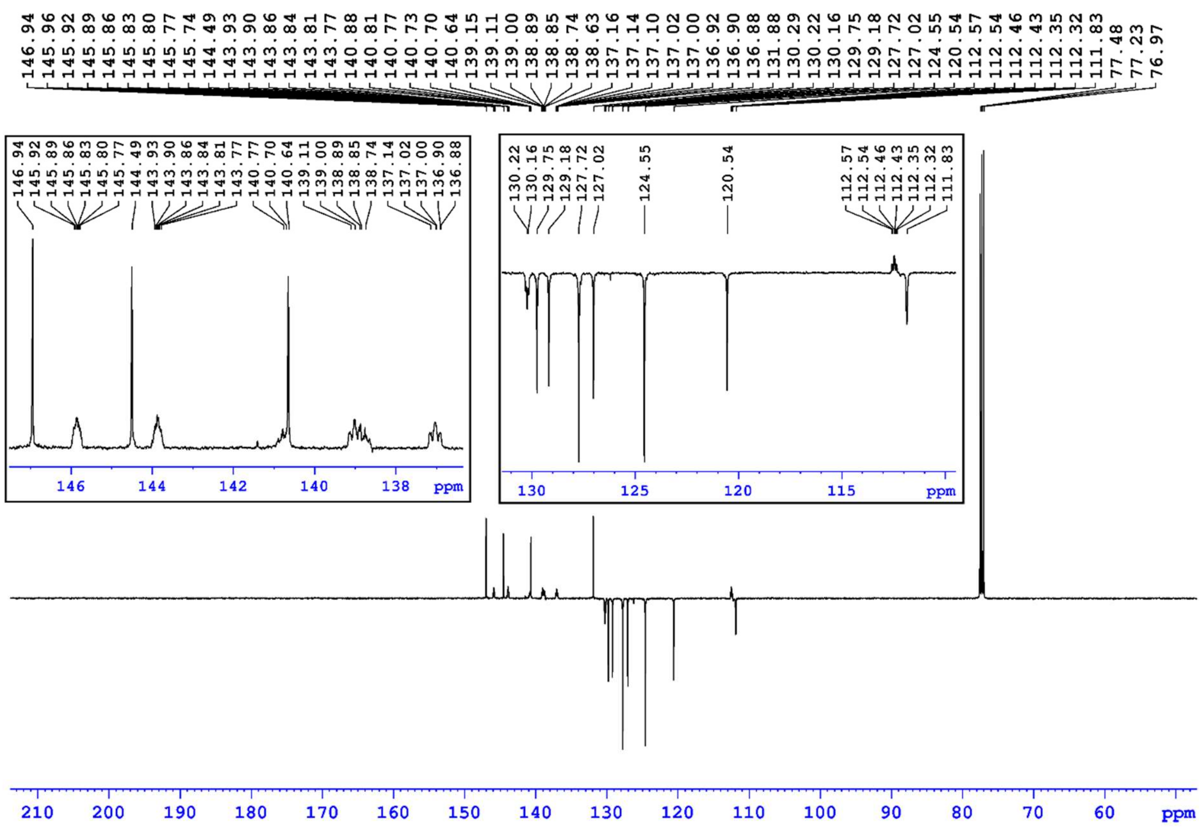

Figure S96. ^{13}C-NMR APT spectrum of fluorophore **1d** (125 MHz, $CDCl_3$, 25 °C).

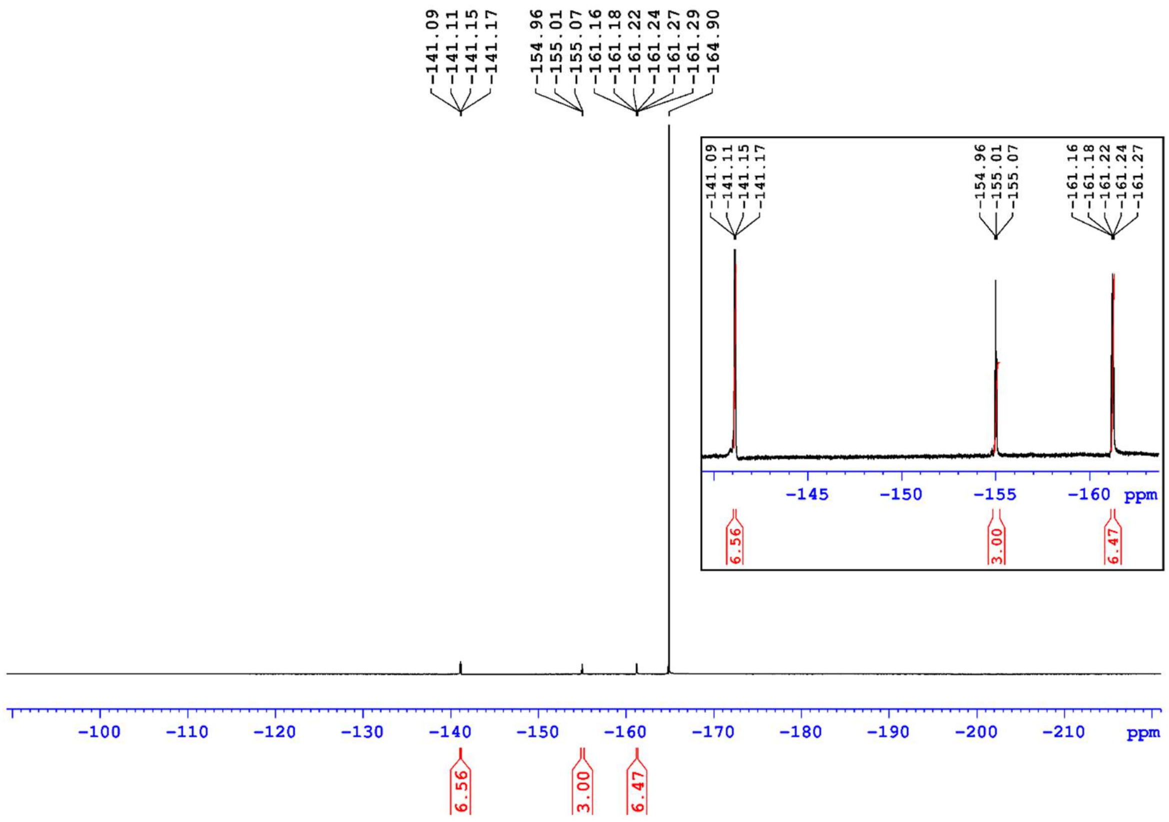

Figure S97. ^{19}F-NMR spectrum of fluorophore **1d** (470 MHz, $CDCl_3$, 25 °C).

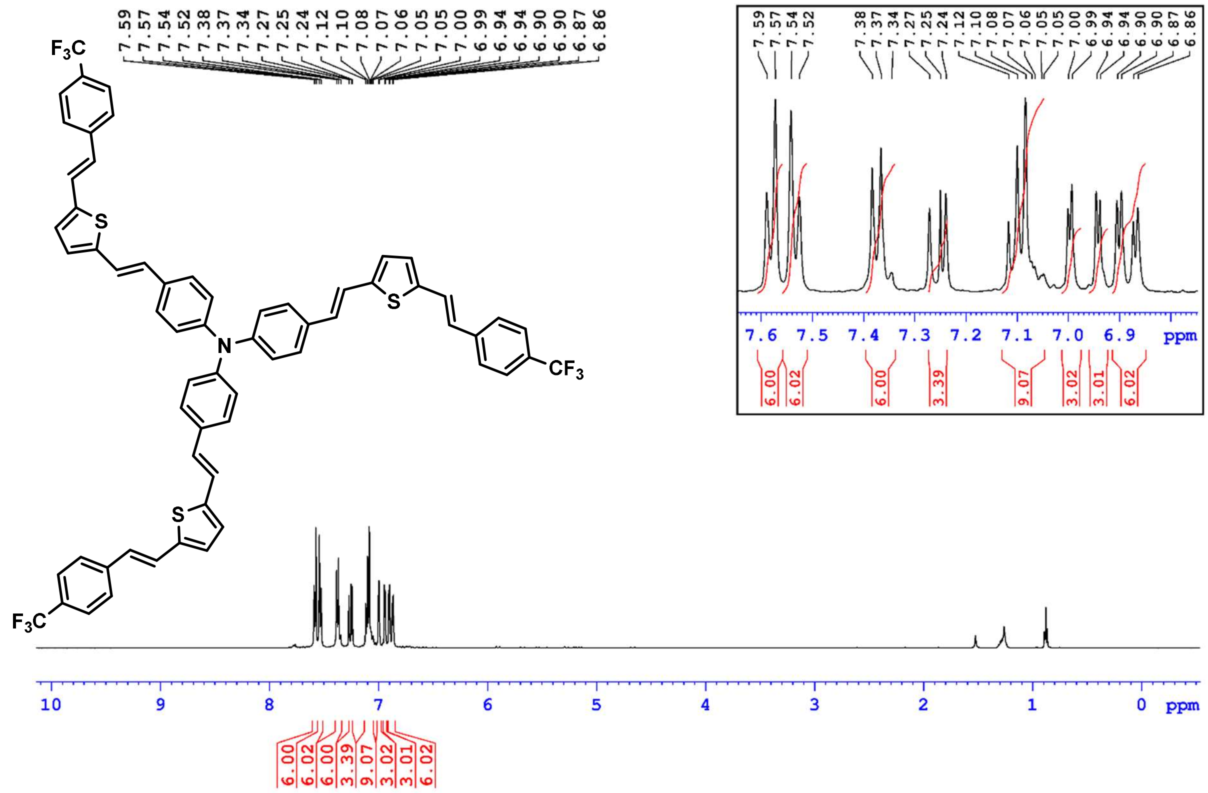

Figure S98. ^{1}H-NMR spectrum of fluorophore **2a** (500 MHz, $CDCl_3$, 25 °C).

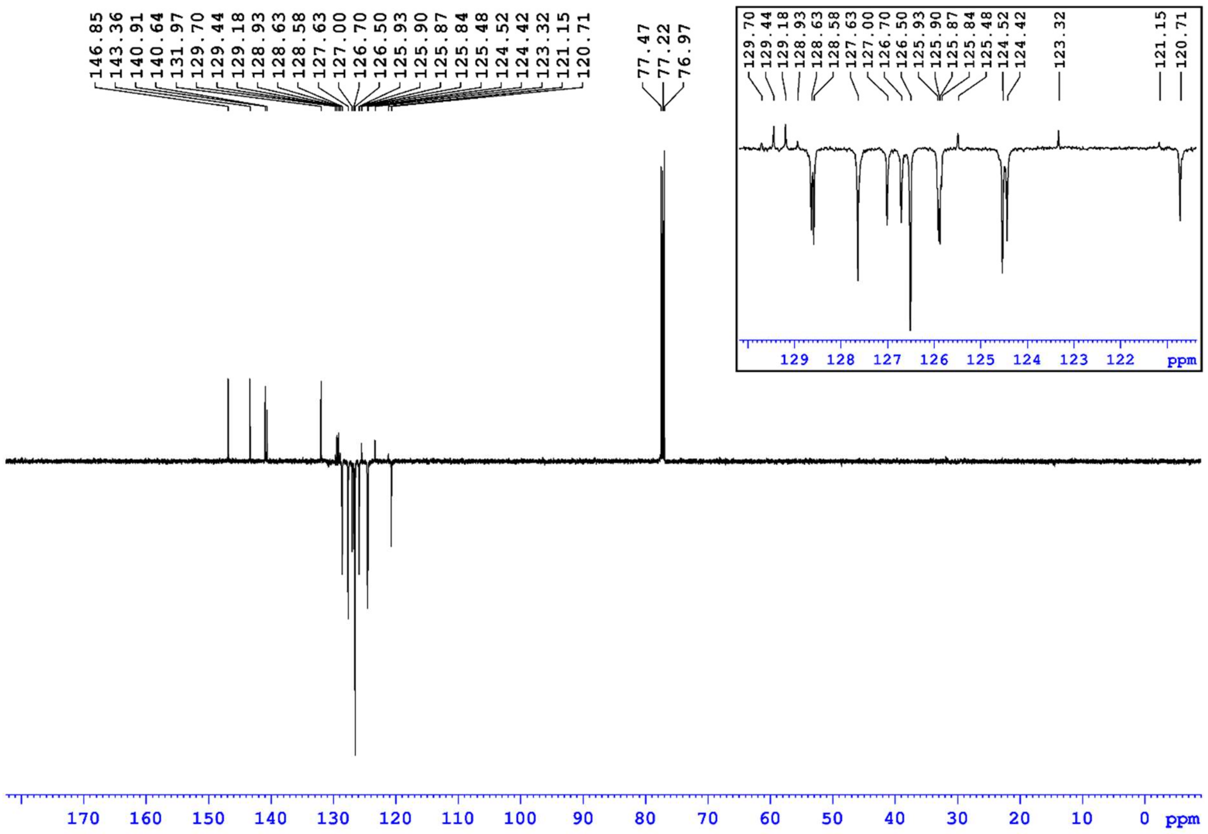

Figure S99. ^{13}C-NMR APT spectrum of fluorophore **2a** (125 MHz, $CDCl_3$, 25 °C).

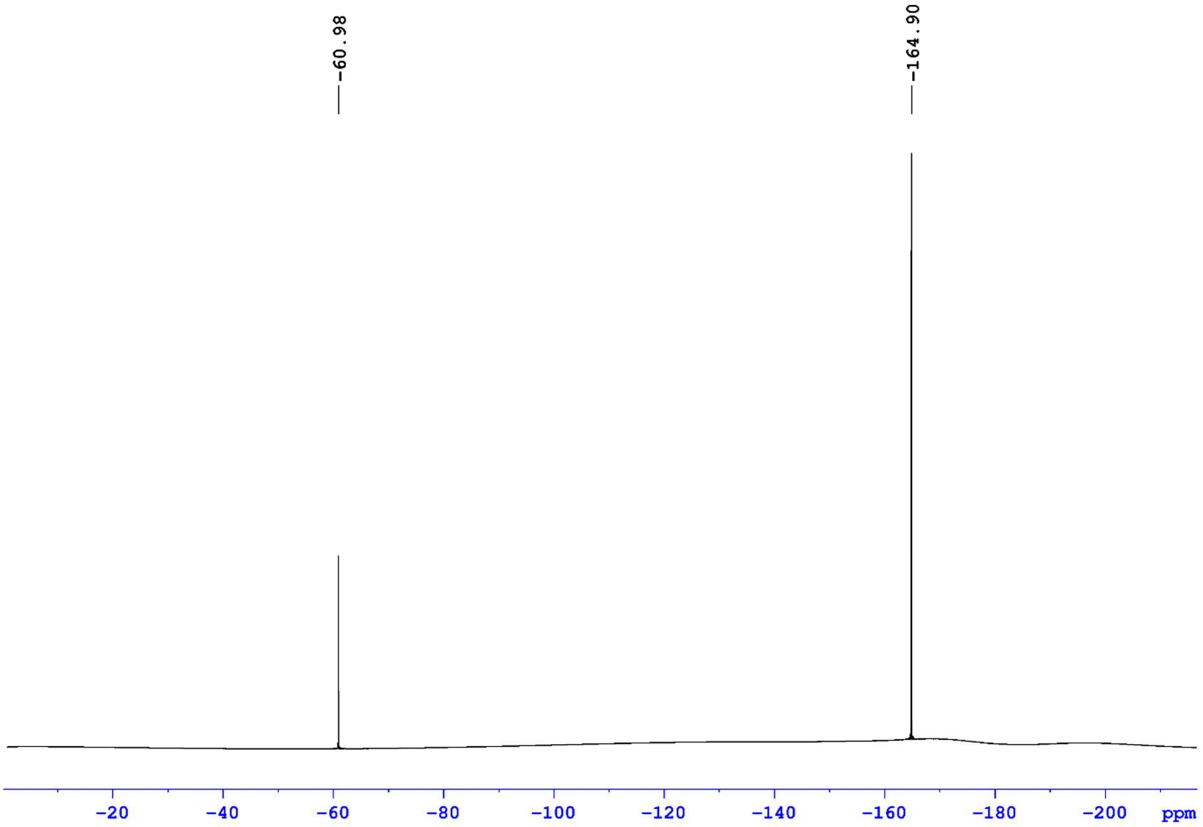

Figure S100. ^{19}F-NMR spectrum of fluorophore **2a** (470 MHz, $CDCl_3$, 25 °C).

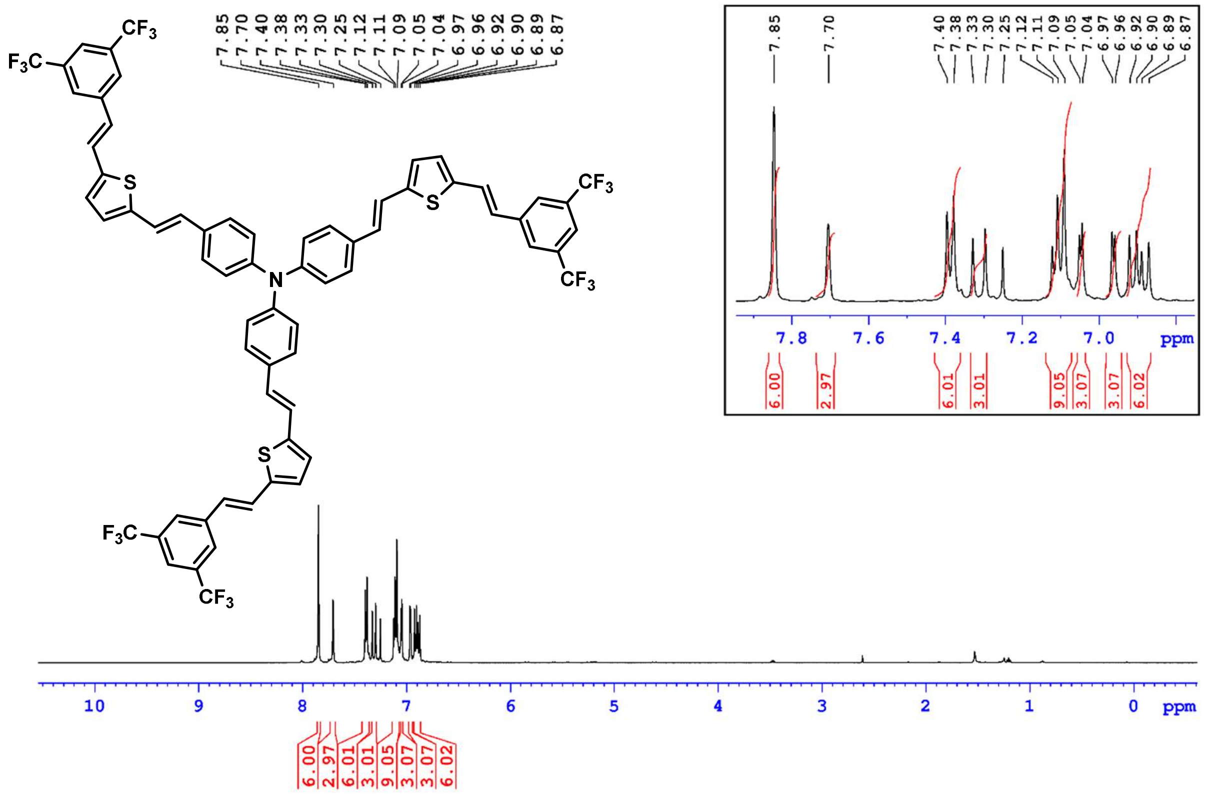

Figure S101. ^{1}H-NMR spectrum of fluorophore **2b** (500 MHz, $CDCl_3$, 25 °C).

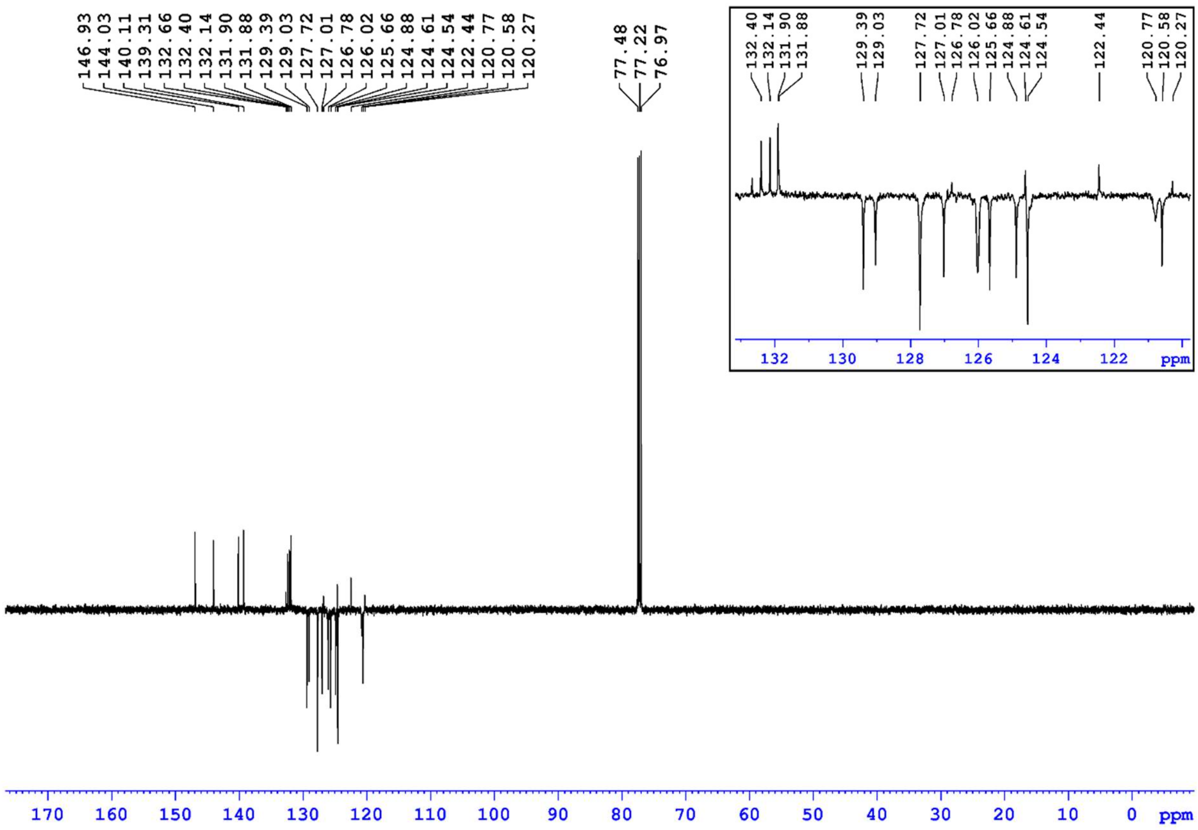

Figure S102. ^{13}C-NMR APT spectrum of fluorophore **2b** (125 MHz, $CDCl_3$, 25 °C).

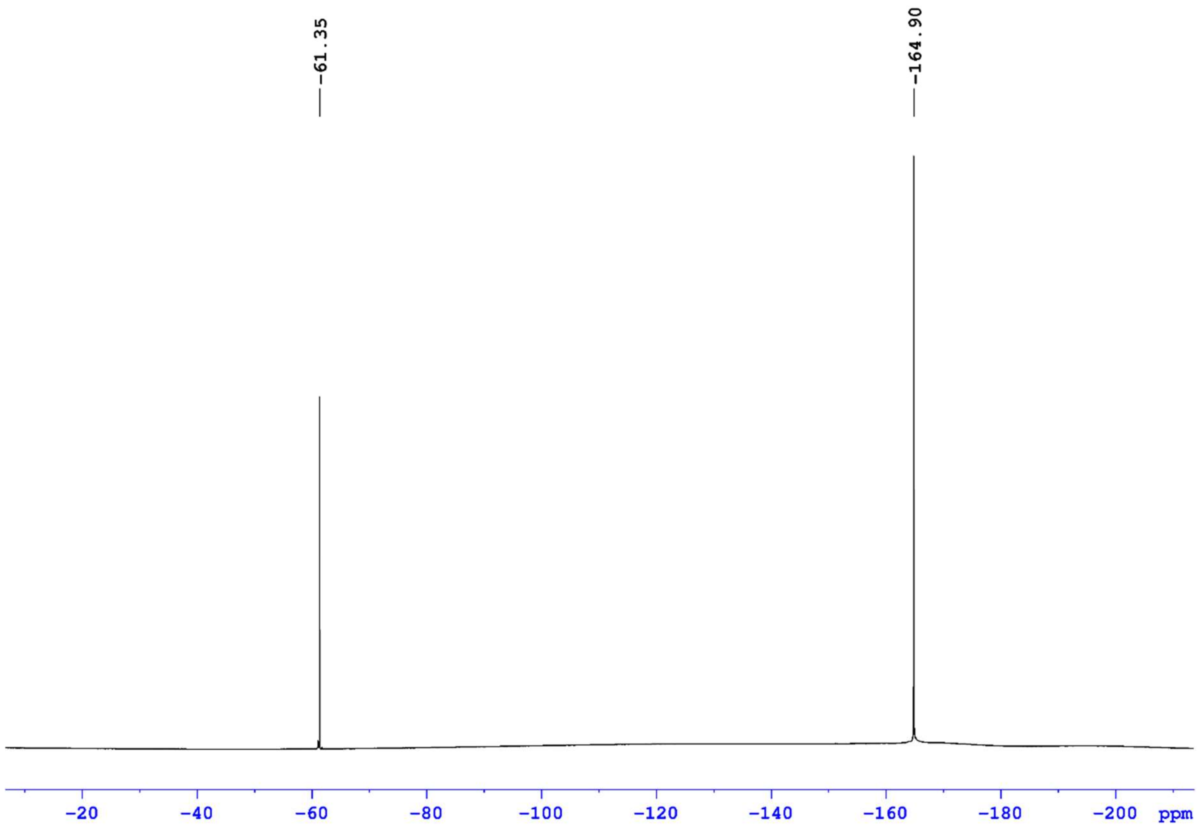

Figure S103. ^{19}F-NMR spectrum of fluorophore **2b** (470 MHz, $CDCl_3$, 25 °C).

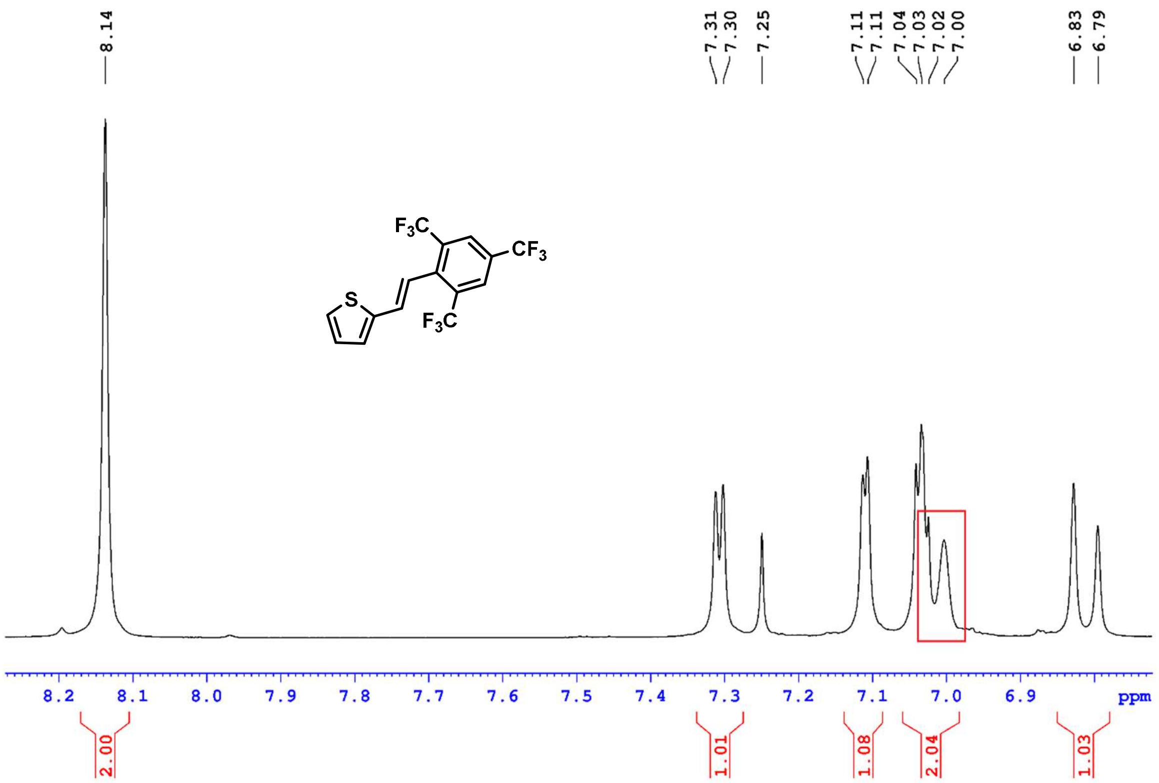

Figure S104. ^{1}H-NMR spectrum of thiophene derivative **17c** (500 MHz, $CDCl_3$, 25 °C). The red box highlights the broadened signal corresponding to the ethylene π-spacer.

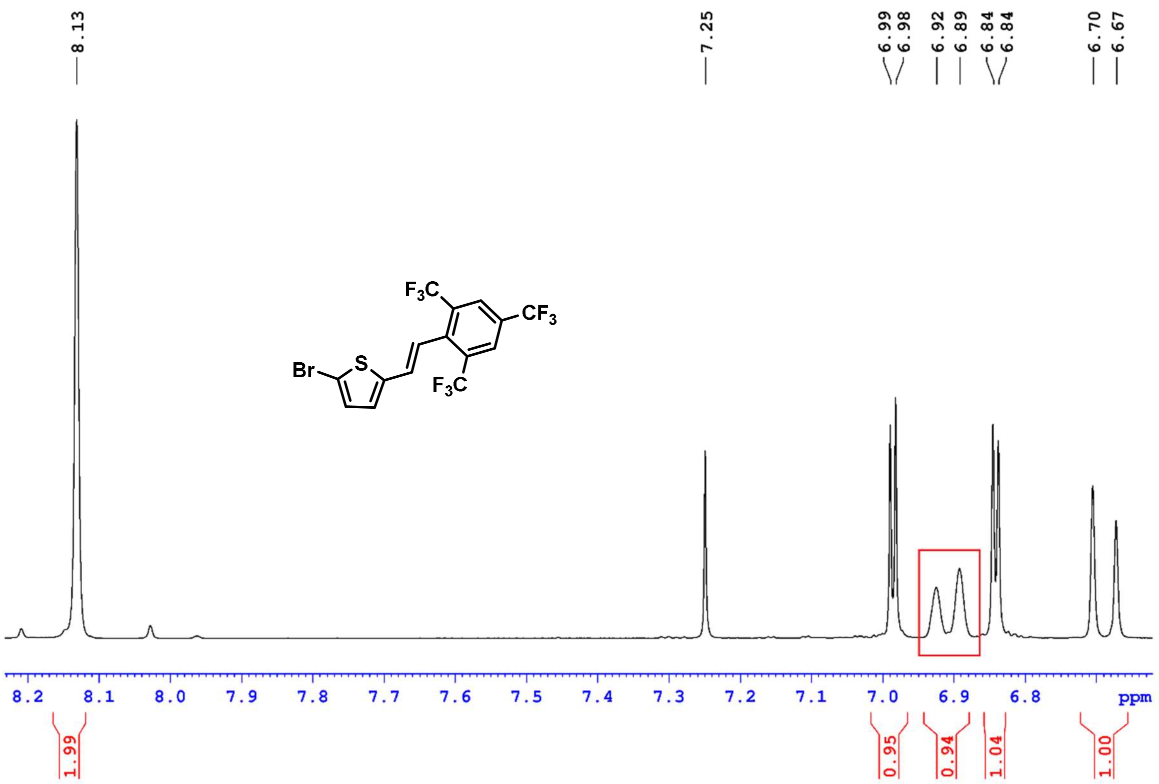

Figure S105. ^{1}H-NMR spectrum of 2-bromothiophene derivative **22c** (500 MHz, $CDCl_3$, 25 °C). The red box highlights the broadened signal corresponding to the ethylene π-spacer.

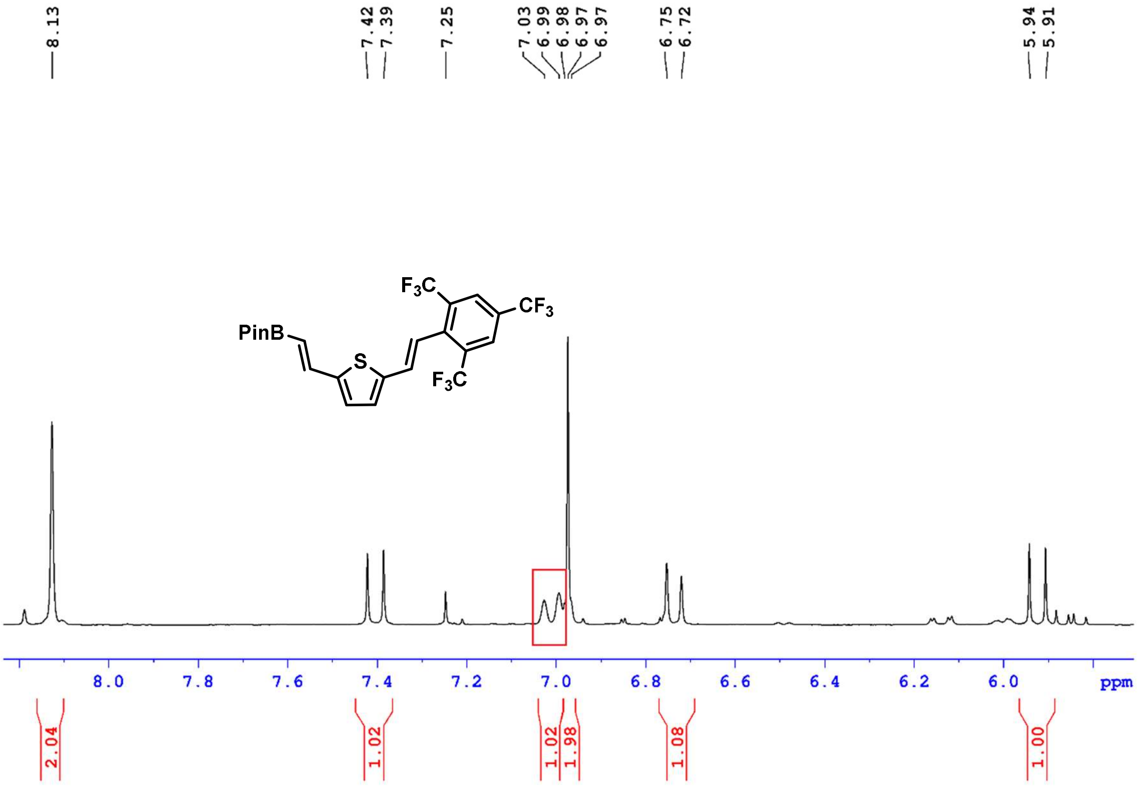

Figure S106. ^{1}H-NMR spectrum (olefinic region) of 2-bromothiophene derivative **27c** (500 MHz, $CDCl_3$, 25 °C). The red box highlights the broadened signal corresponding to the ethylene π-spacer adjacent to the tris(trifluoromethyl)phenyl unit.

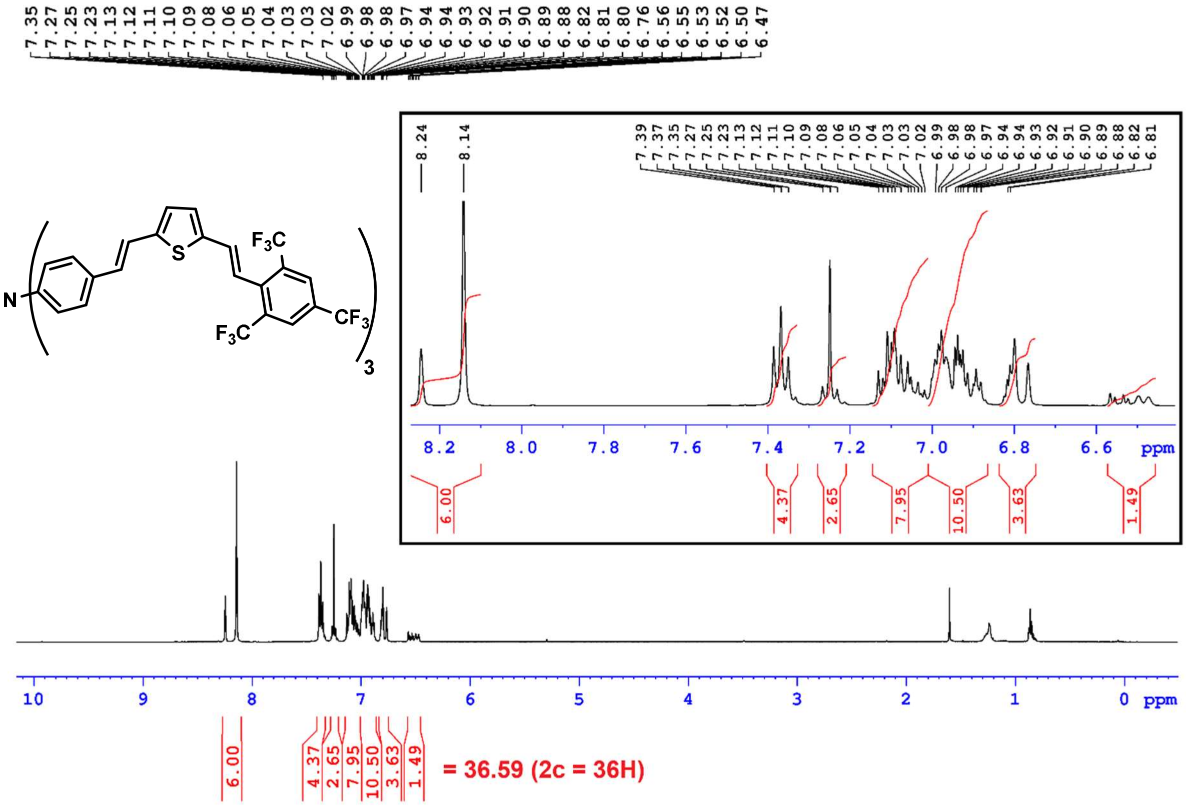

Figure S107. ^{1}H-NMR spectrum of fluorophore **2c** (500 MHz, $CDCl_3$, 25 °C).

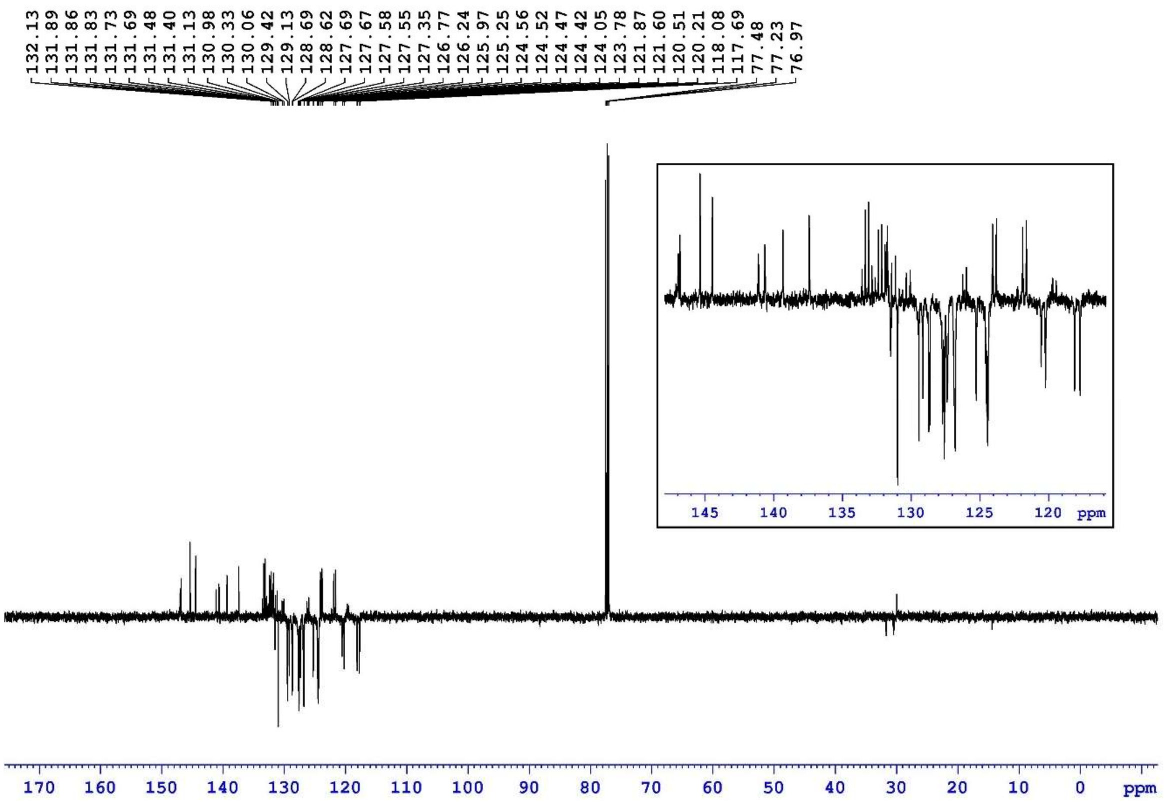

Figure S108. ^{13}C-NMR APT spectrum of fluorophore **2c** (125 MHz, $CDCl_3$, 25 °C).

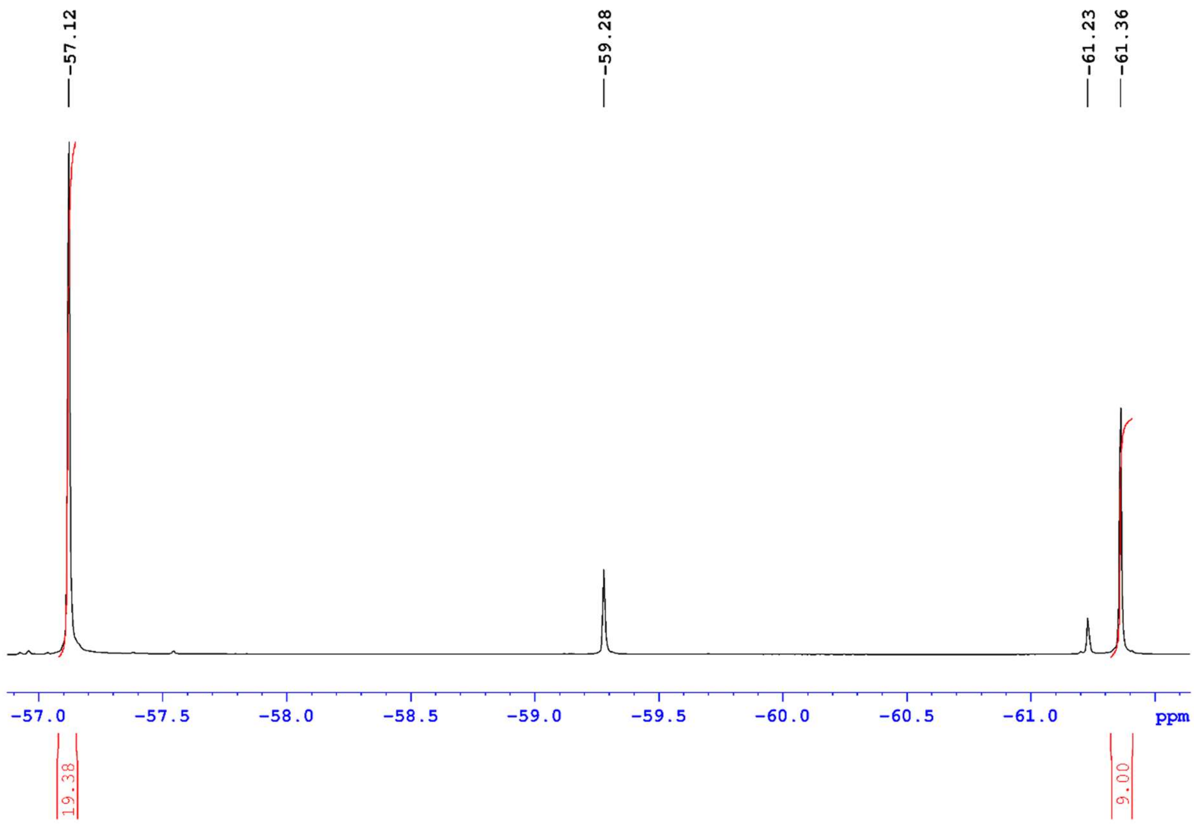

Figure S109. ^{19}F-NMR spectrum of fluorophore **2c** (470 MHz, $CDCl_3$, 25 °C).

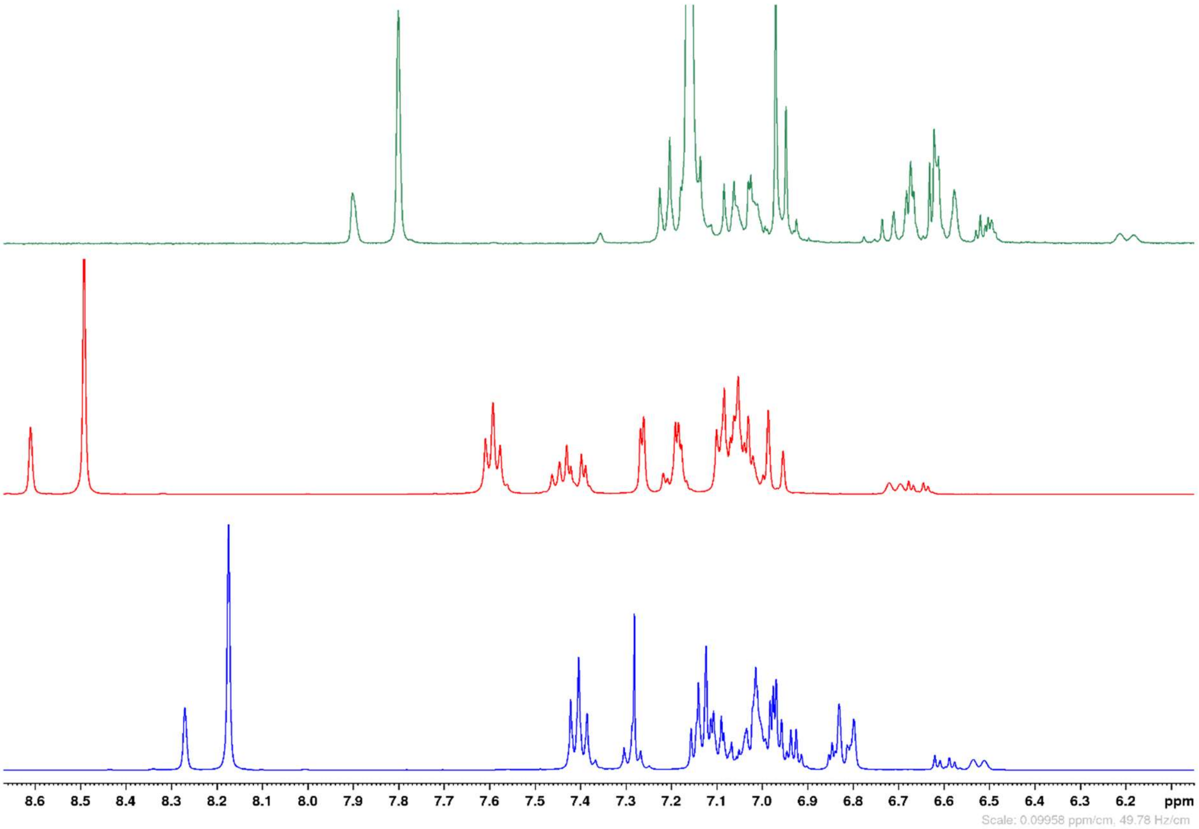

Figure S110. ^{1}H-NMR spectra of fluorophore **2c** measured in different solvents (**$CDCl_3$**, ***d_6*-DMSO**, **C_6D_6**).

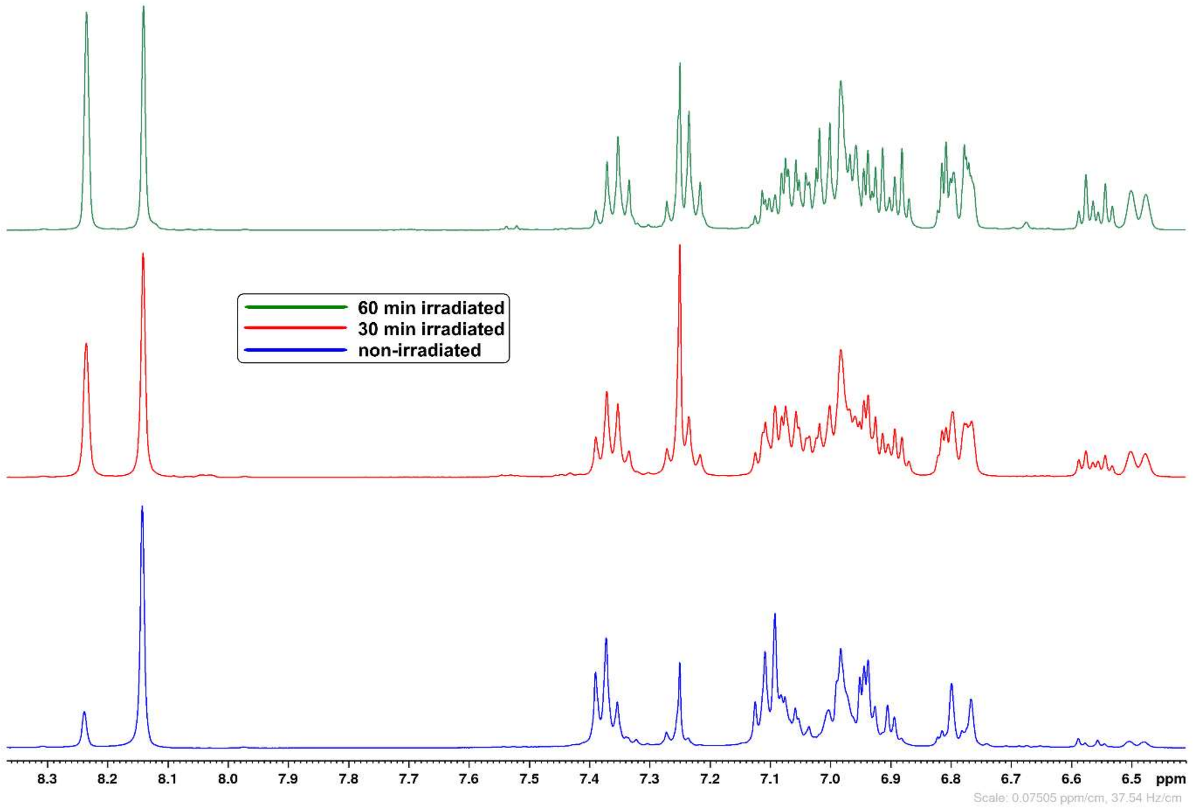

Figure S111. ^{1}H-NMR spectra of fluorophore **2c** under irradiation (Royal Blue LED, λ_{max}^{E} = 450 nm).

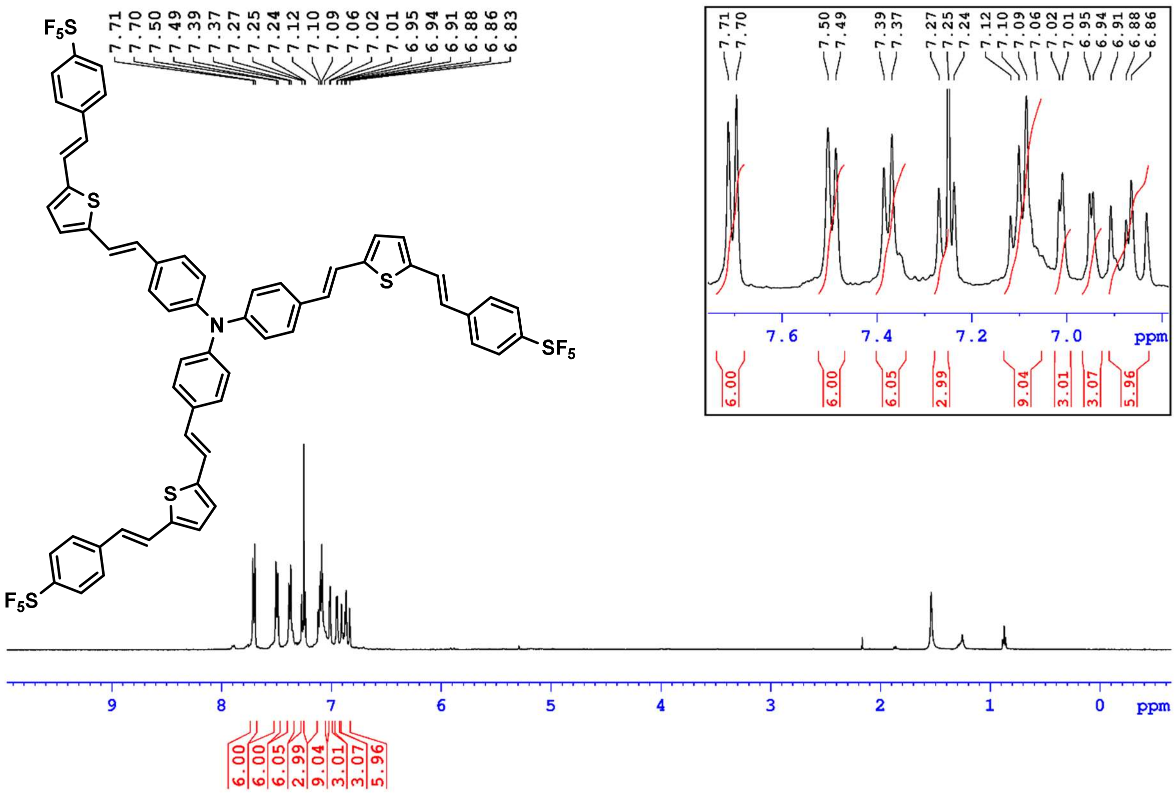

Figure S112. ^{1}H-NMR spectrum of fluorophore **2d** (500 MHz, $CDCl_3$, 25 °C).

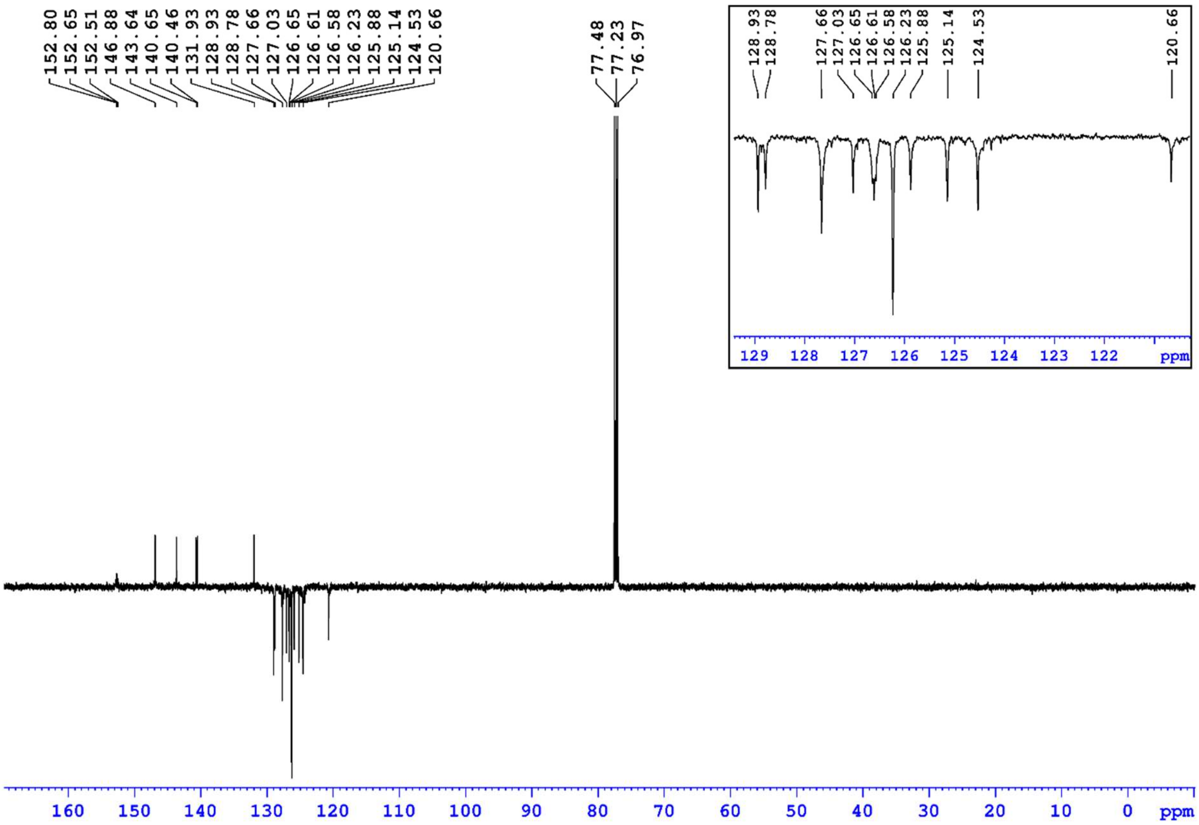

Figure S113. ^{13}C-NMR APT spectrum of fluorophore **2d** (125 MHz, $CDCl_3$, 25 °C).

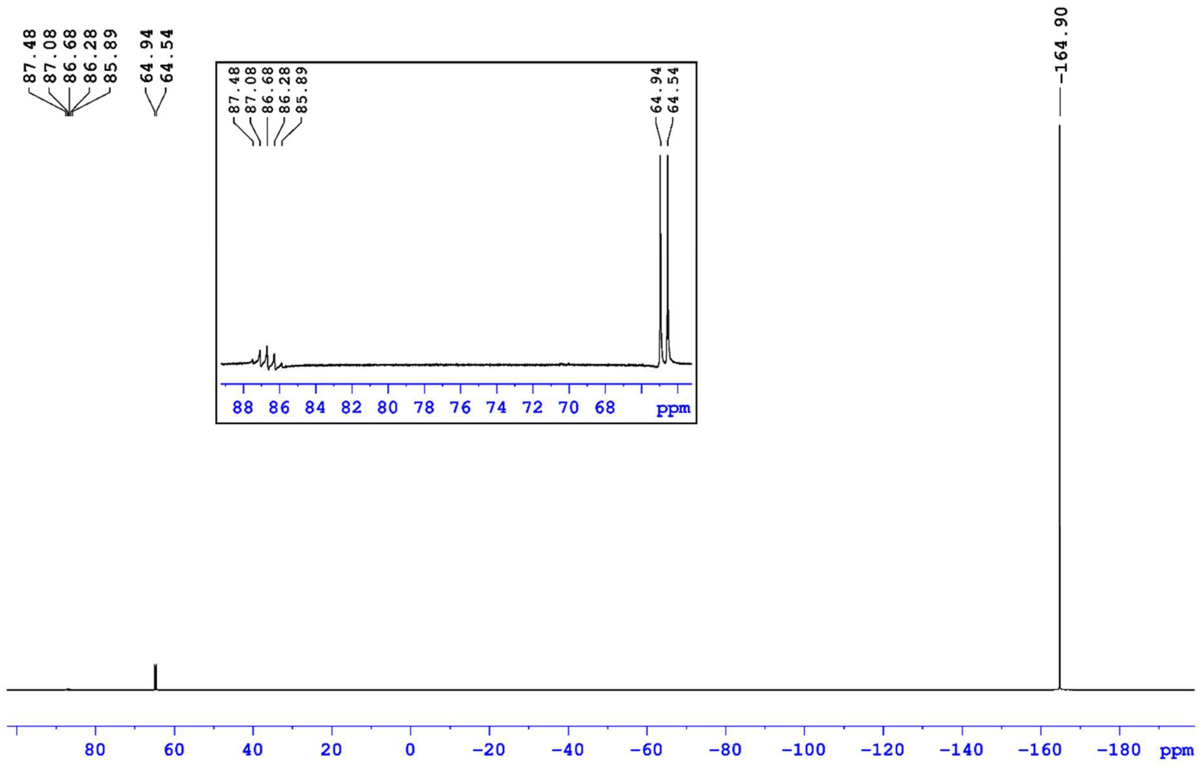

Figure S114. ^{19}F-NMR spectrum of fluorophore **2d** (470 MHz, $CDCl_3$, 25 °C).

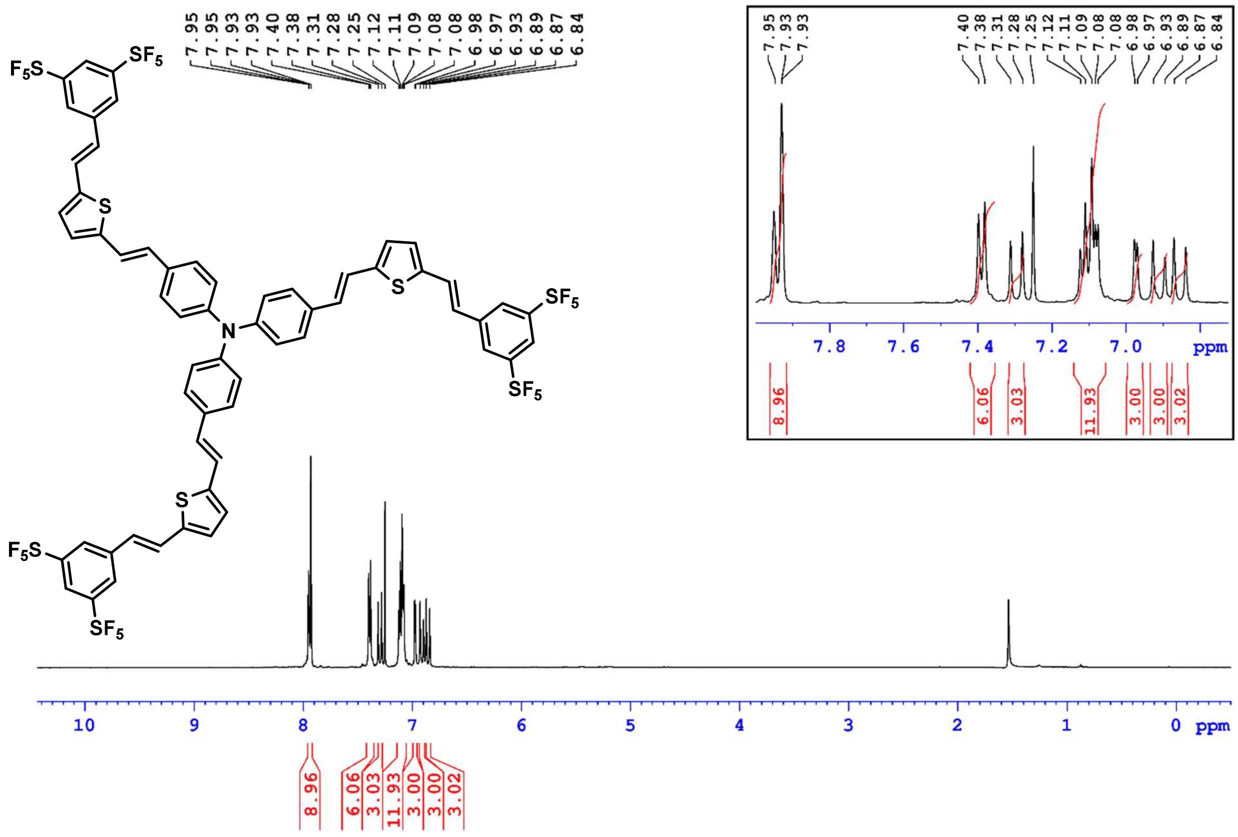

Figure S115. ^{1}H-NMR spectrum of fluorophore **2e** (500 MHz, $CDCl_3$, 25 °C).

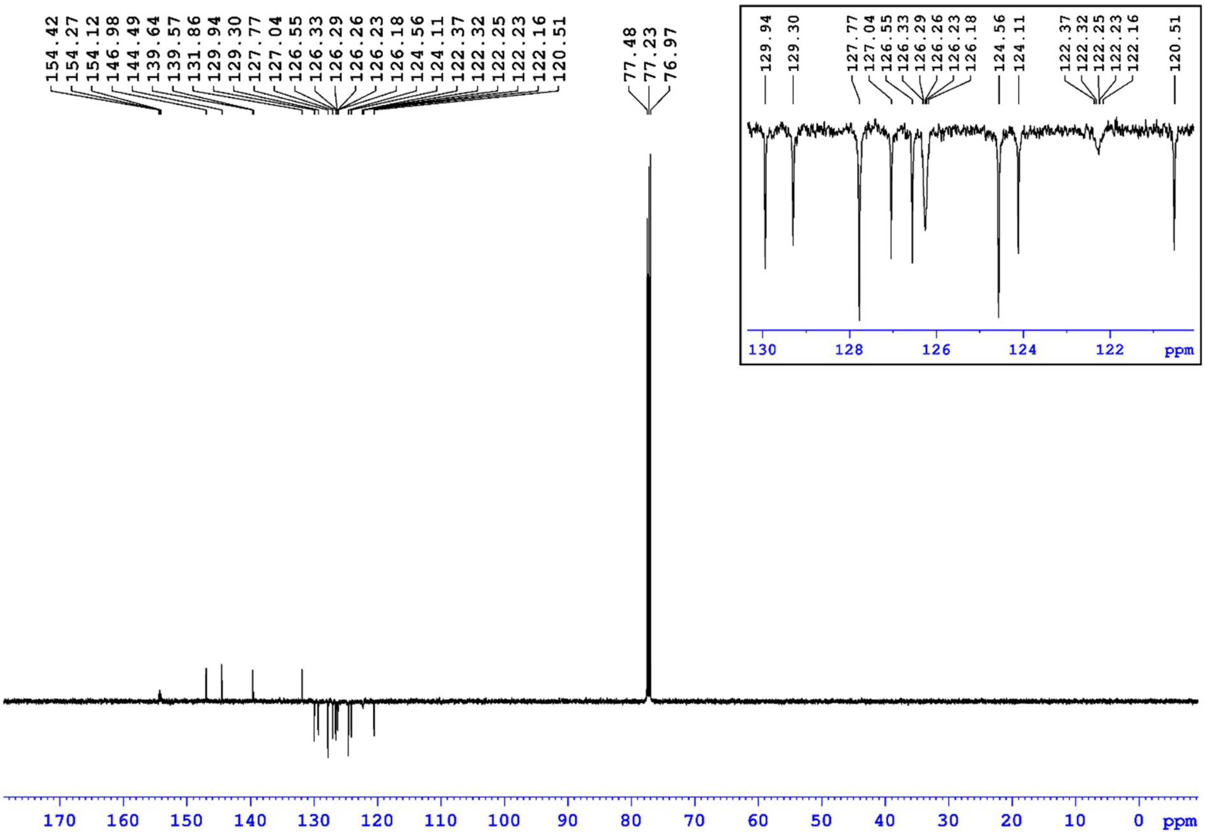

Figure S116. ^{13}C-NMR APT spectrum of fluorophore **2e** (125 MHz, $CDCl_3$, 25 °C).

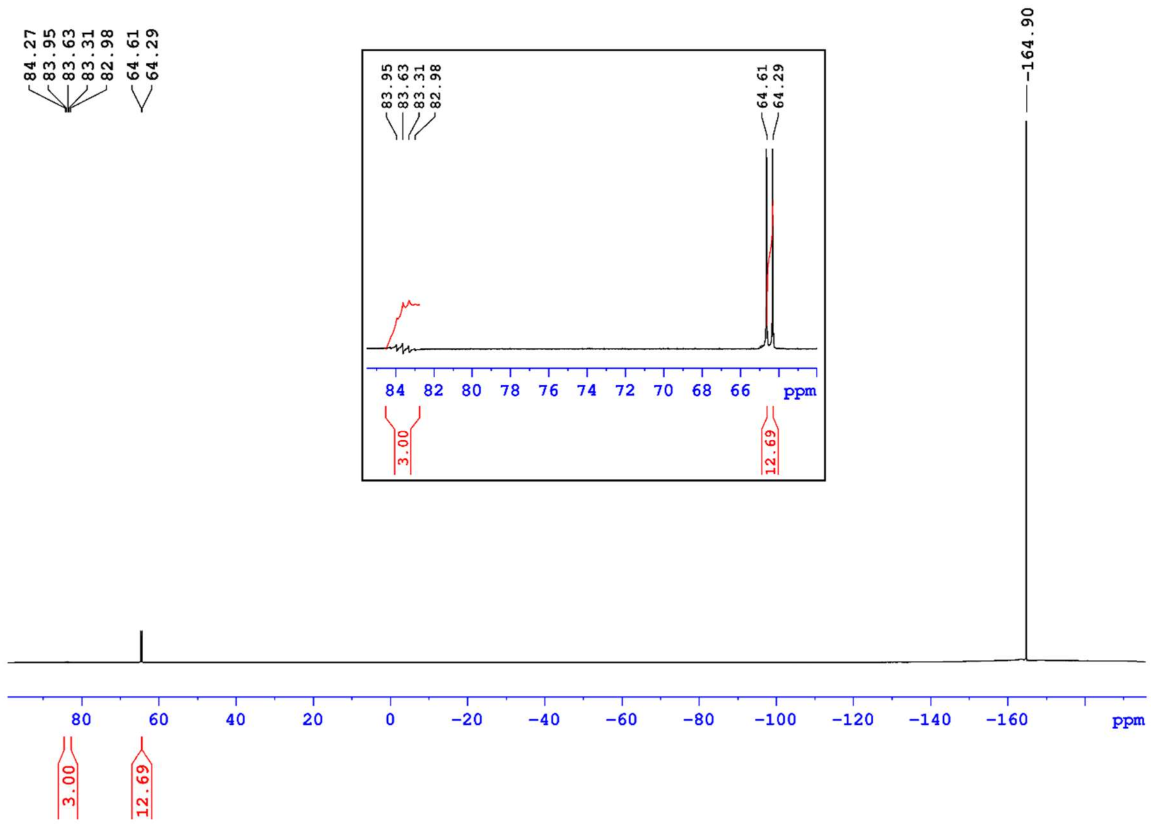

Figure S117. ^{19}F-NMR spectrum of fluorophore **2e** (470 MHz, $CDCl_3$, 25 °C).

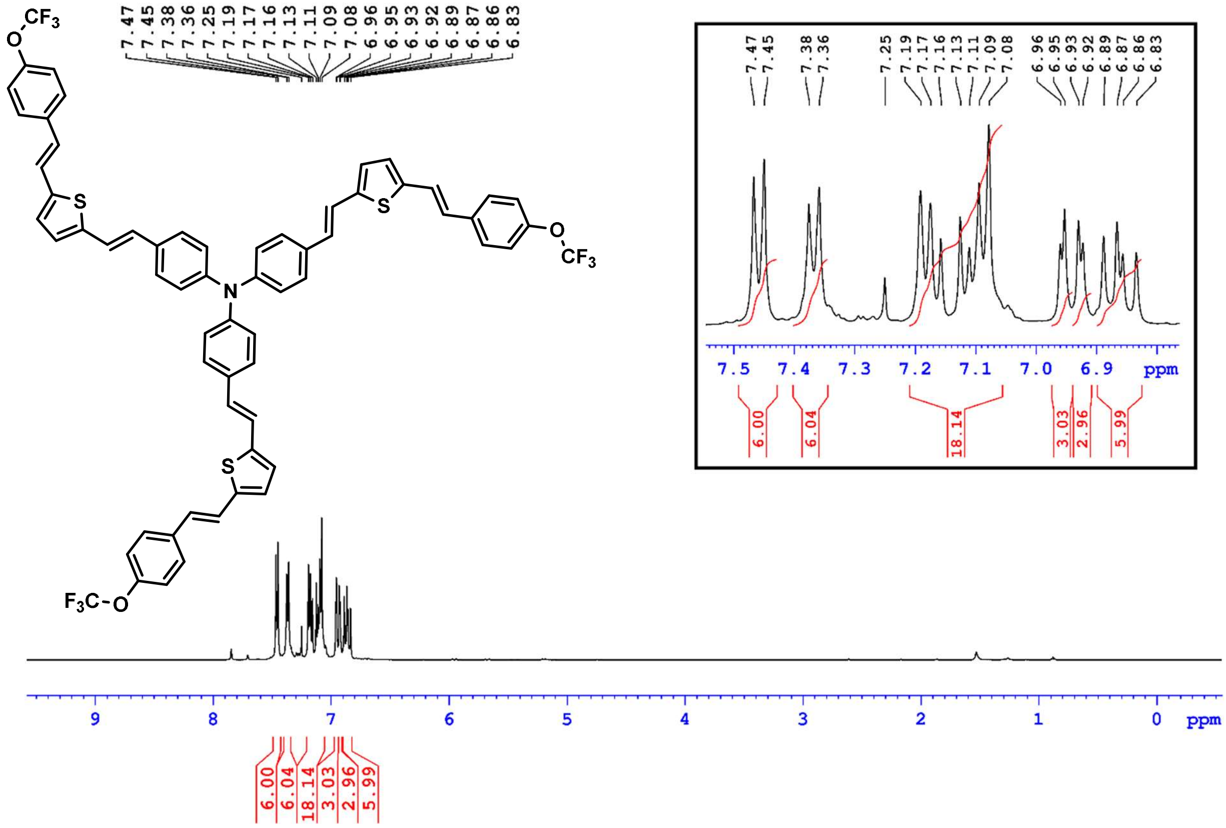

Figure S118. ^{1}H-NMR spectrum of fluorophore **3a** (500 MHz, $CDCl_3$, 25 °C).

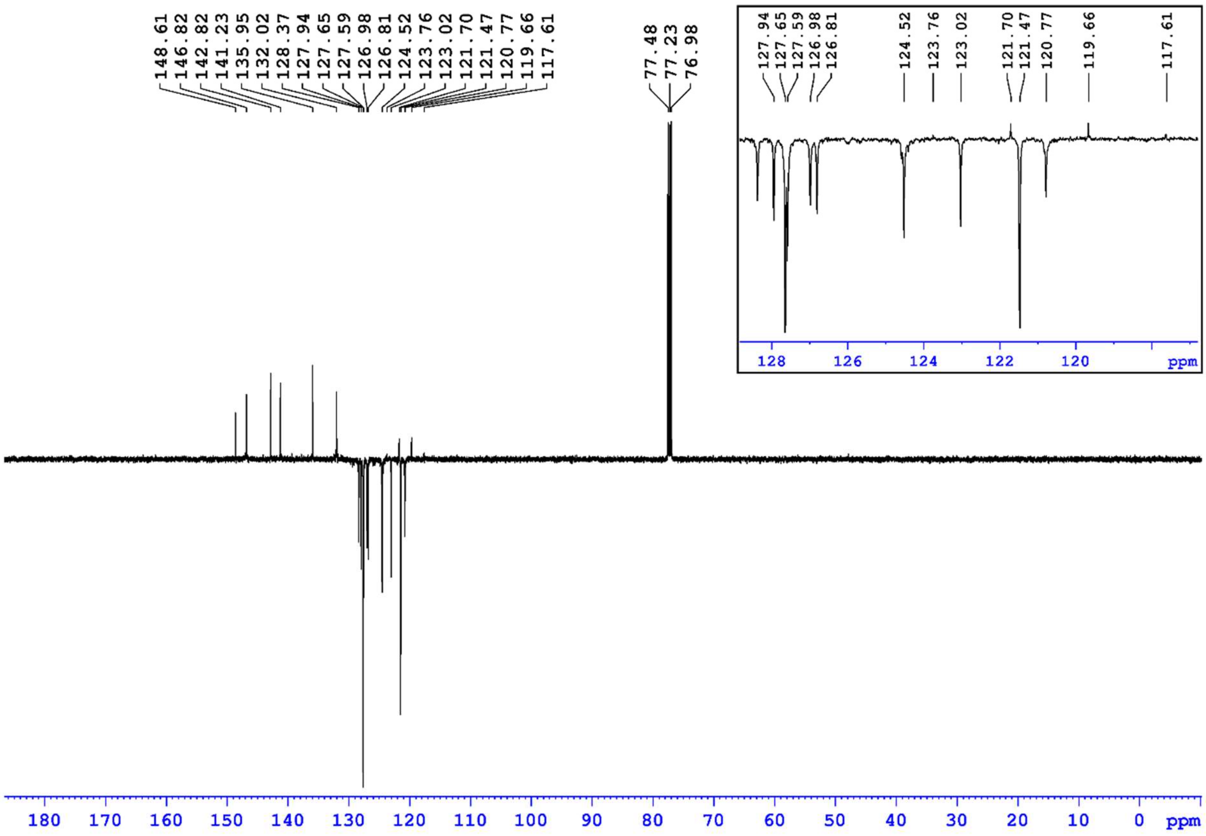

Figure S119. ^{13}C-NMR APT spectrum of fluorophore **3a** (125 MHz, $CDCl_3$, 25 °C).

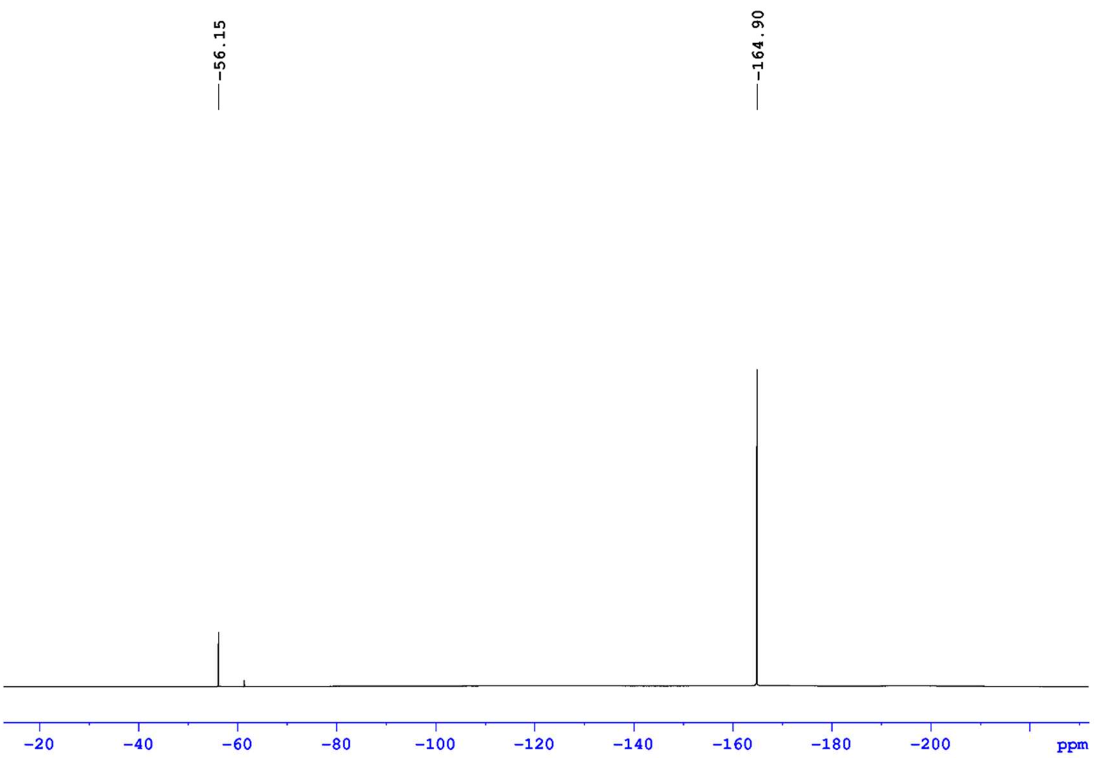

Figure S120. ^{19}F-NMR spectrum of fluorophore **3a** (470 MHz, $CDCl_3$, 25 °C).

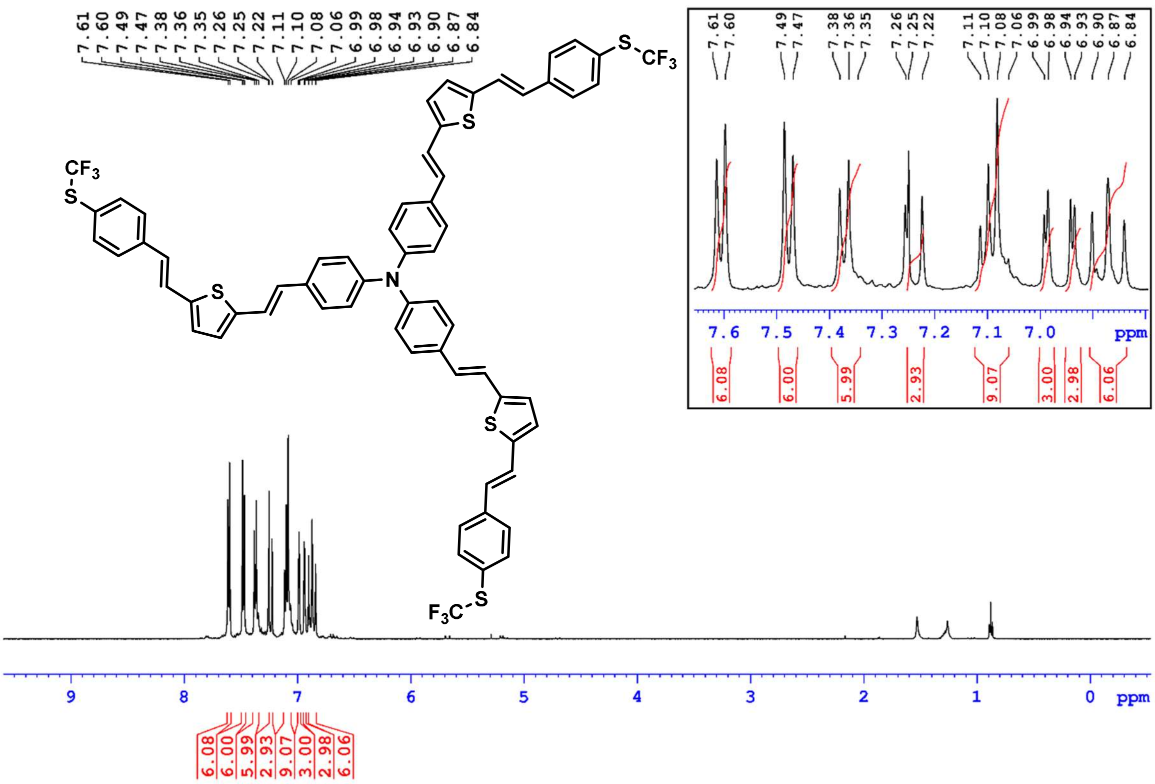

Figure S121. ^{1}H-NMR spectrum of fluorophore **3b** (500 MHz, $CDCl_3$, 25 °C).

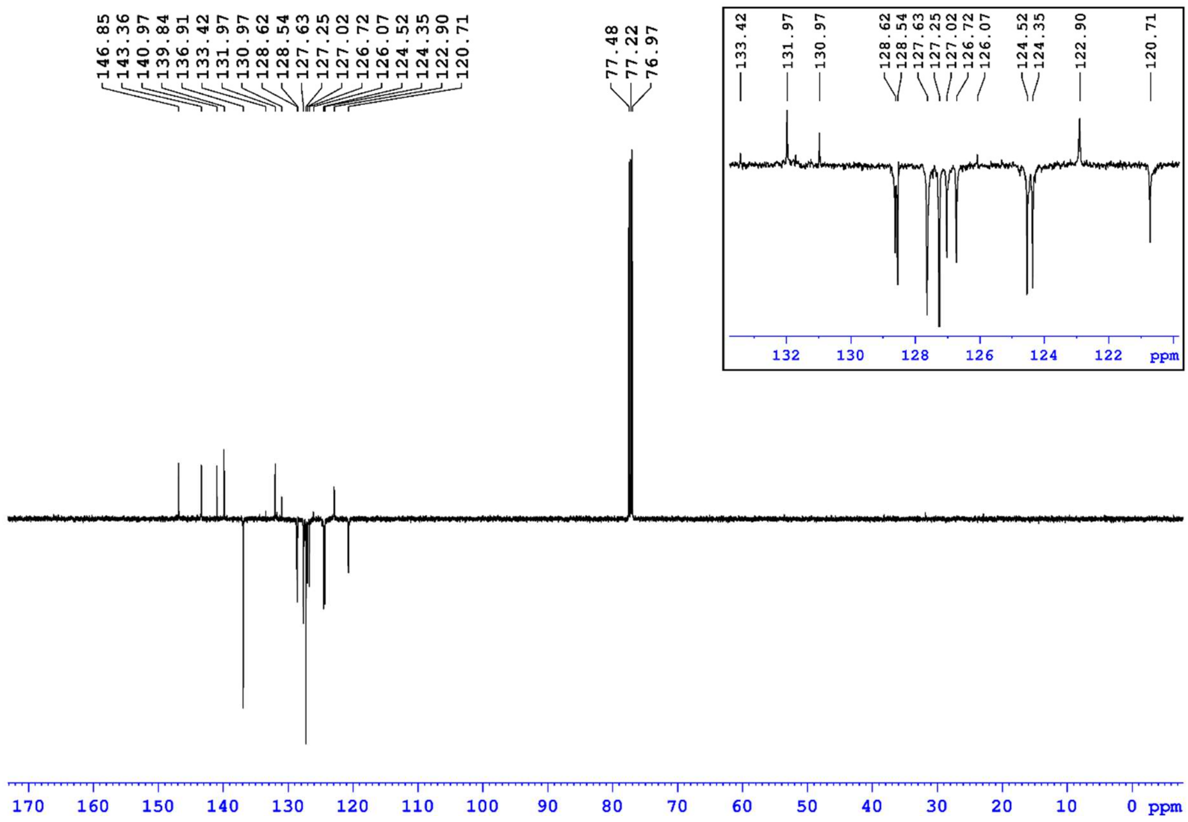

Figure S122. ^{13}C-NMR APT spectrum of fluorophore **3b** (125 MHz, $CDCl_3$, 25 °C).

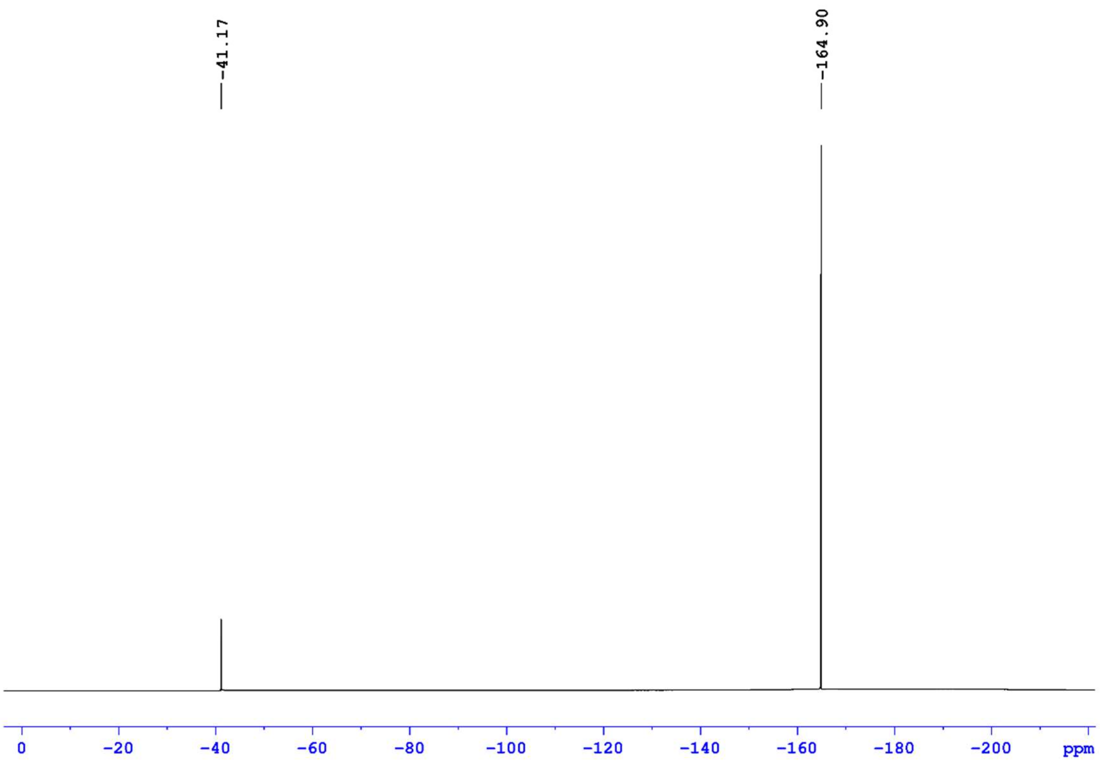

Figure S123. ^{19}F-NMR spectrum of fluorophore **3b** (470 MHz, $CDCl_3$, 25 °C).

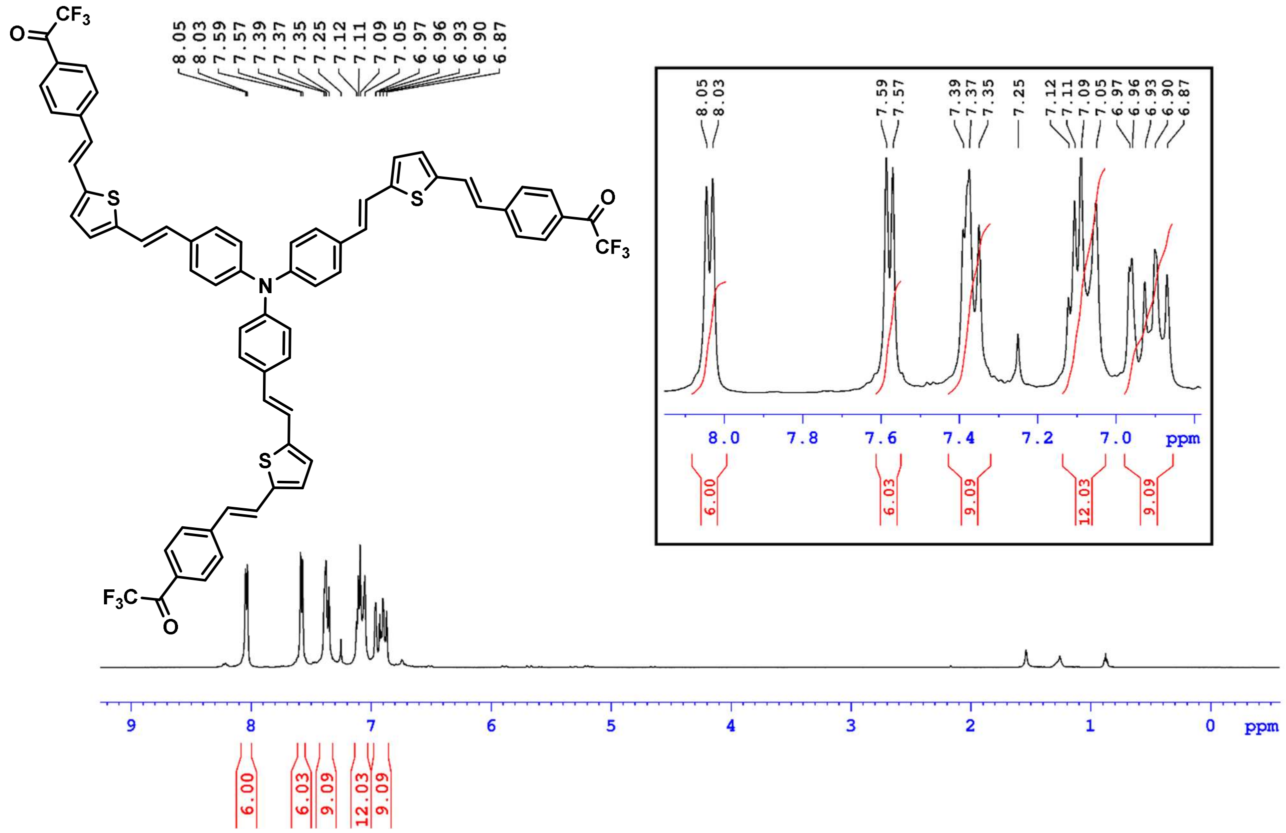

Figure S124. ^{1}H-NMR spectrum of fluorophore **4a** (500 MHz, $CDCl_3$, 25 °C).

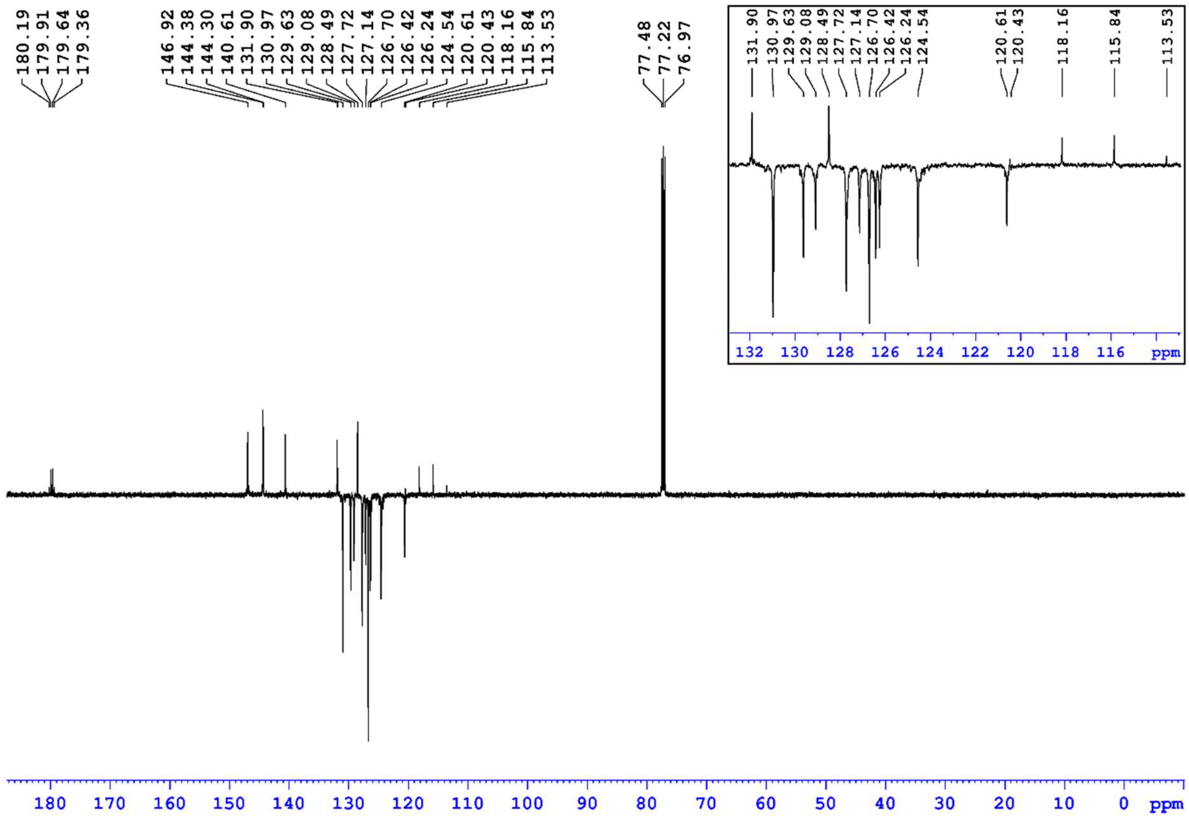

Figure S125. ^{13}C-NMR APT spectrum of fluorophore **4a** (125 MHz, $CDCl_3$, 25 °C).

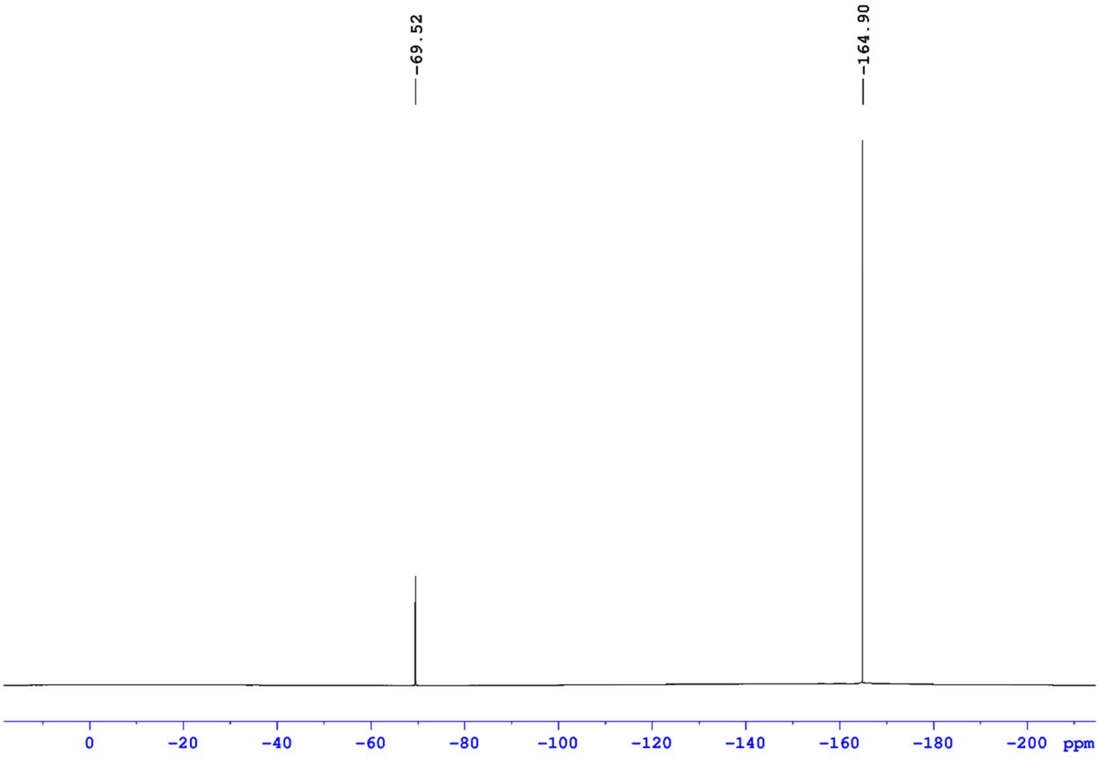

Figure S126. ^{19}F-NMR spectrum of fluorophore **4a** (470 MHz, $CDCl_3$, 25 °C).

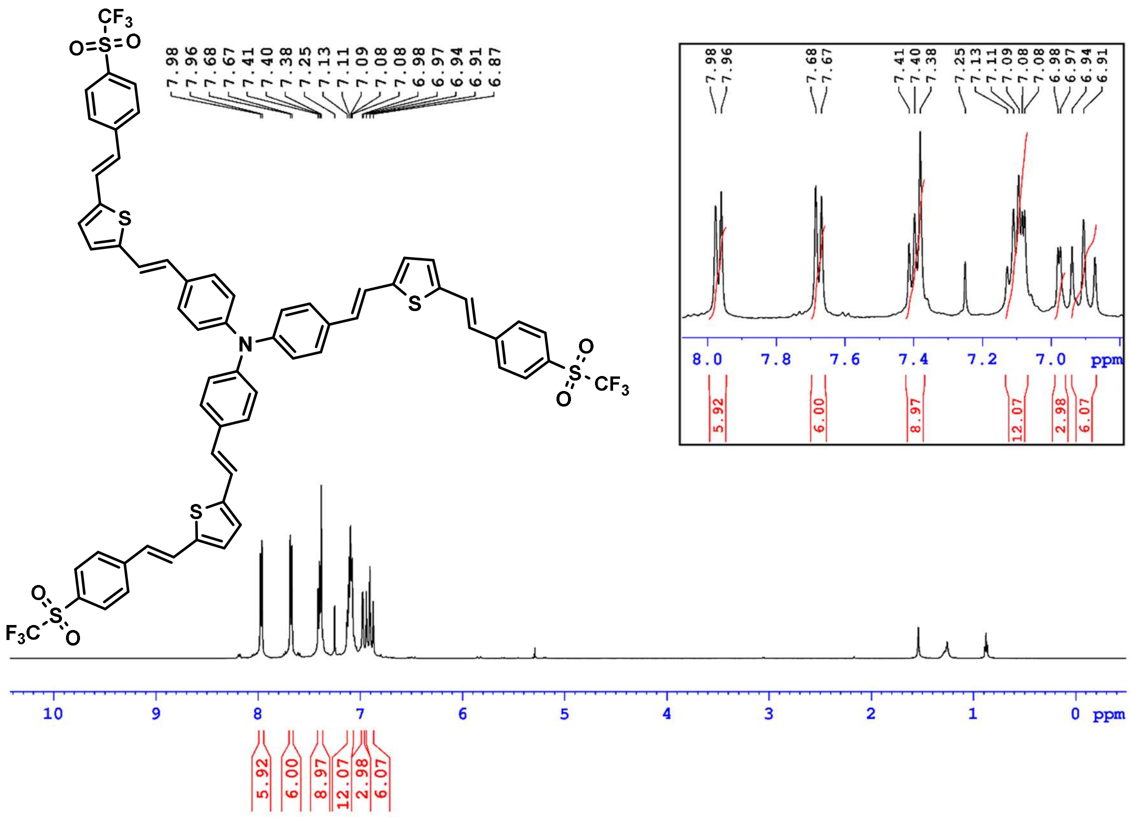

Figure S127. ^{1}H-NMR spectrum of fluorophore **4b** (500 MHz, $CDCl_3$, 25 °C).

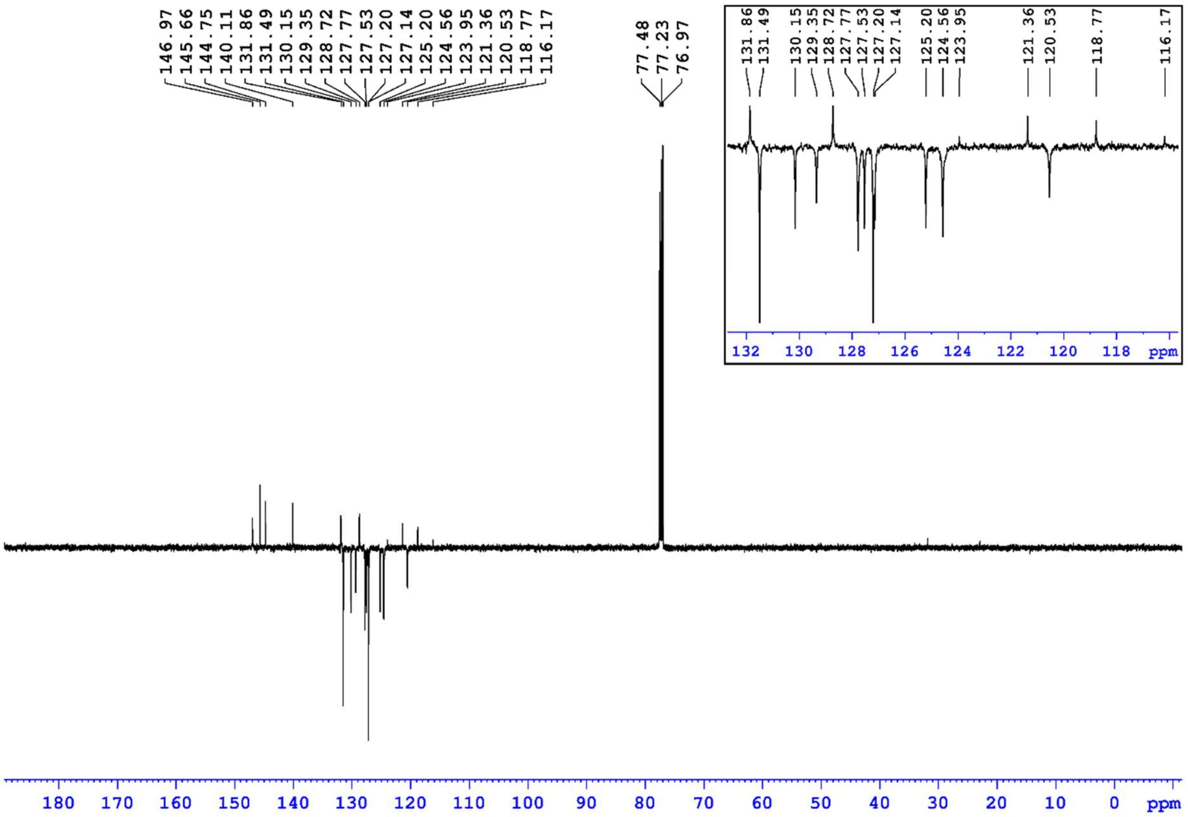

Figure S128. ^{13}C-NMR APT spectrum of fluorophore **4b** (125 MHz, $CDCl_3$, 25 °C).

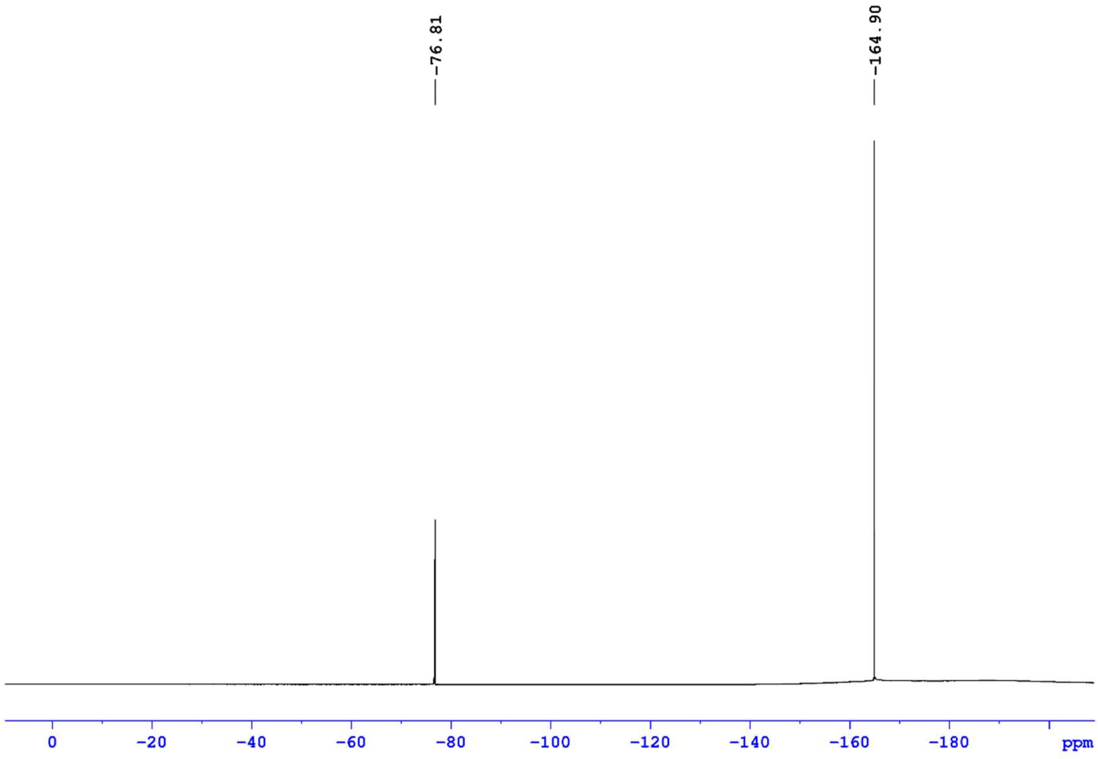

Figure S129. ^{19}F-NMR spectrum of fluorophore **4b** (470 MHz, $CDCl_3$, 25 °C).

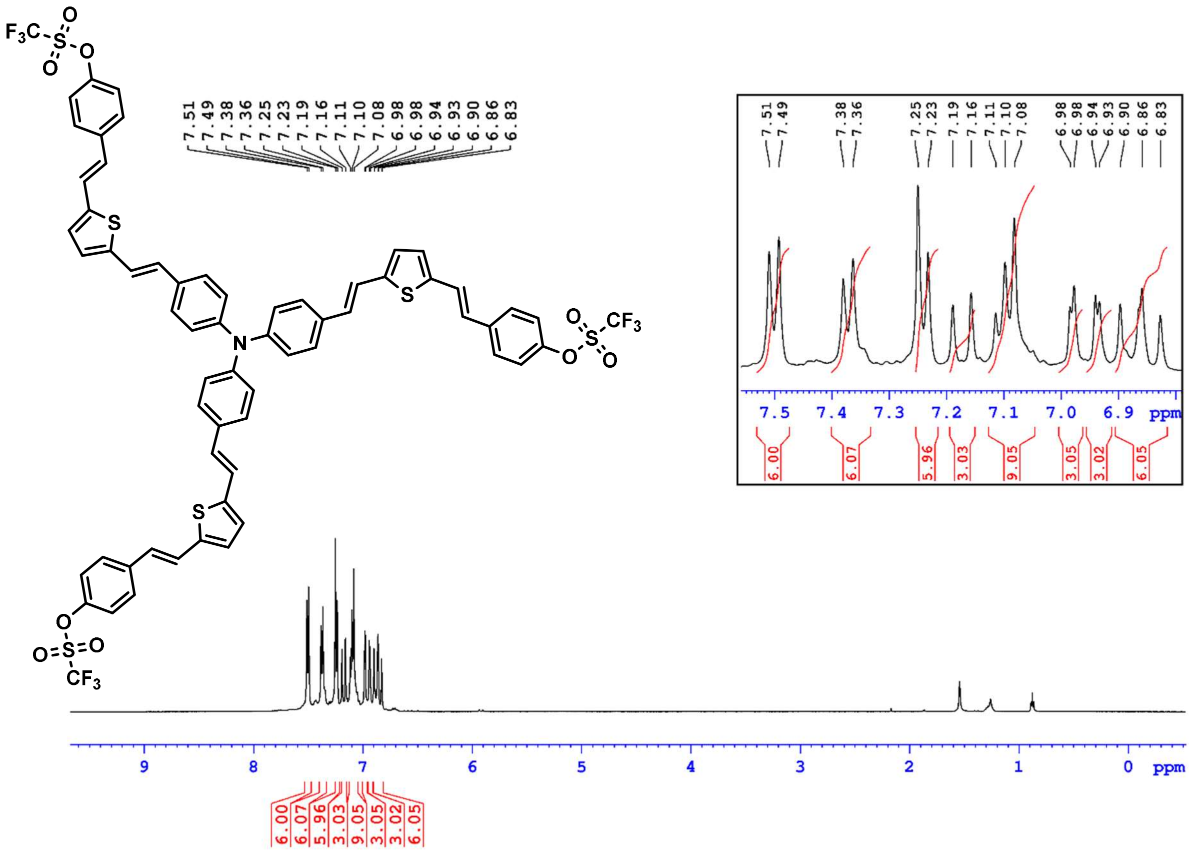

Figure S130. ^{1}H-NMR spectrum of fluorophore **4c** (500 MHz, $CDCl_3$, 25 °C).

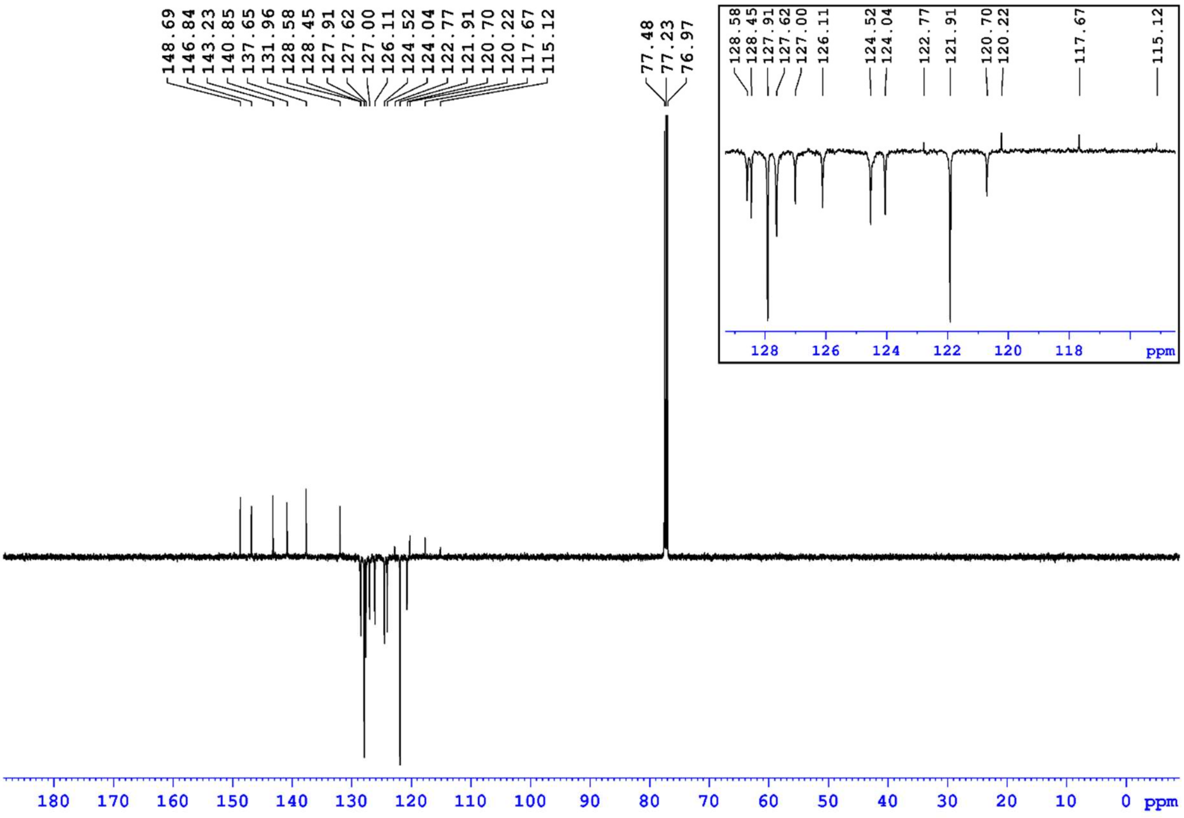

Figure S131. ^{13}C-NMR APT spectrum of fluorophore **4c** (125 MHz, $CDCl_3$, 25 °C).

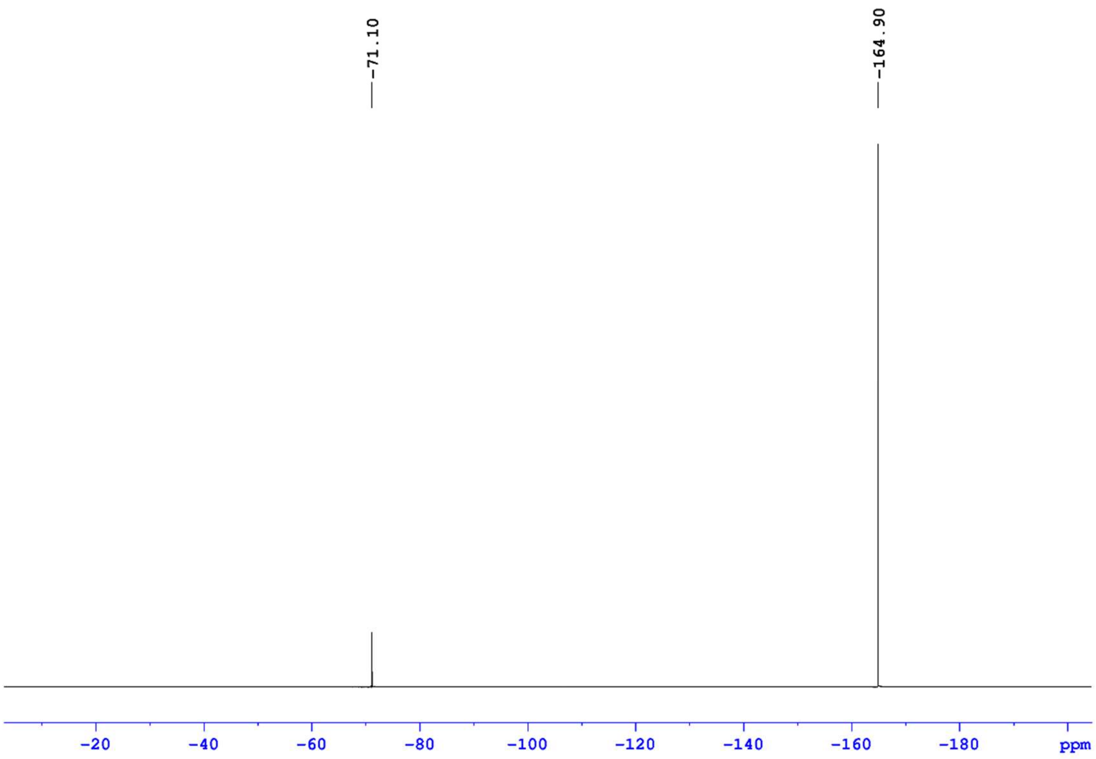

Figure S132. ^{19}F-NMR spectrum of fluorophore **4c** (470 MHz, $CDCl_3$, 25 °C).

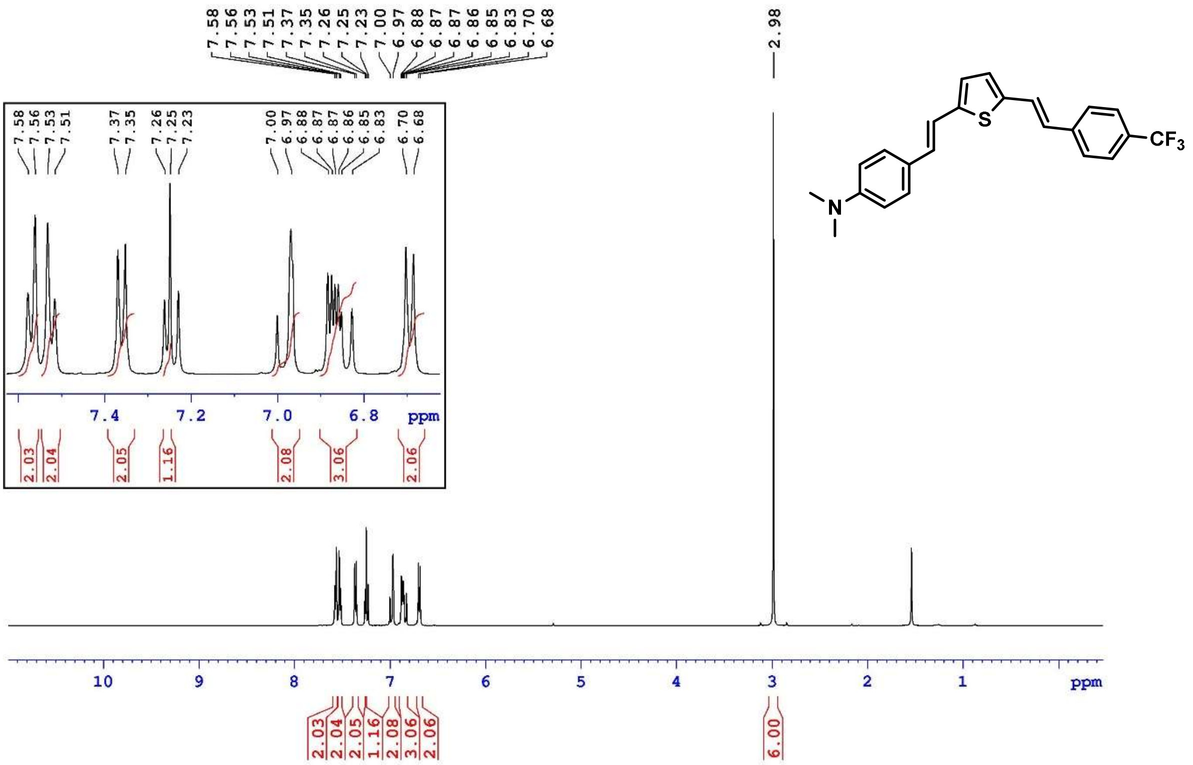

Figure S133. ^{1}H-NMR spectrum of fluorophore **2aL** (500 MHz, $CDCl_3$, 25 °C).

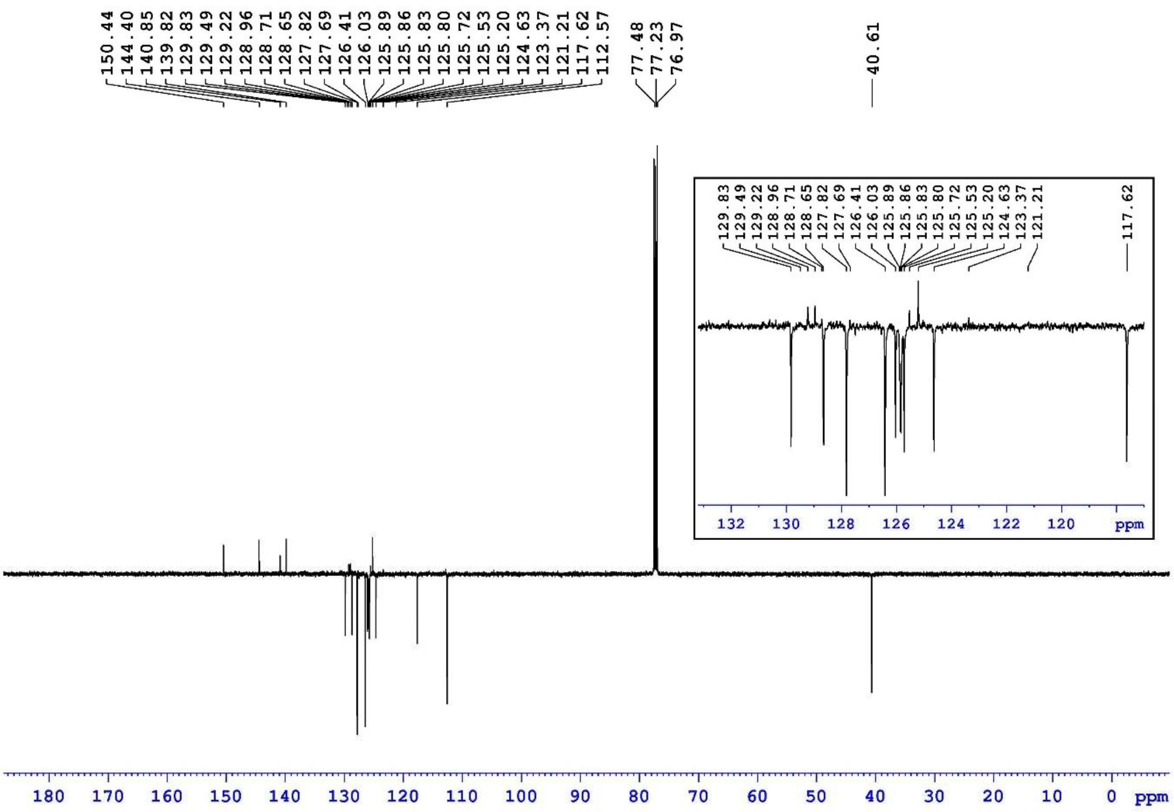

Figure S134. ^{13}C-NMR APT spectrum of fluorophore **2aL** (125 MHz, $CDCl_3$, 25 °C).

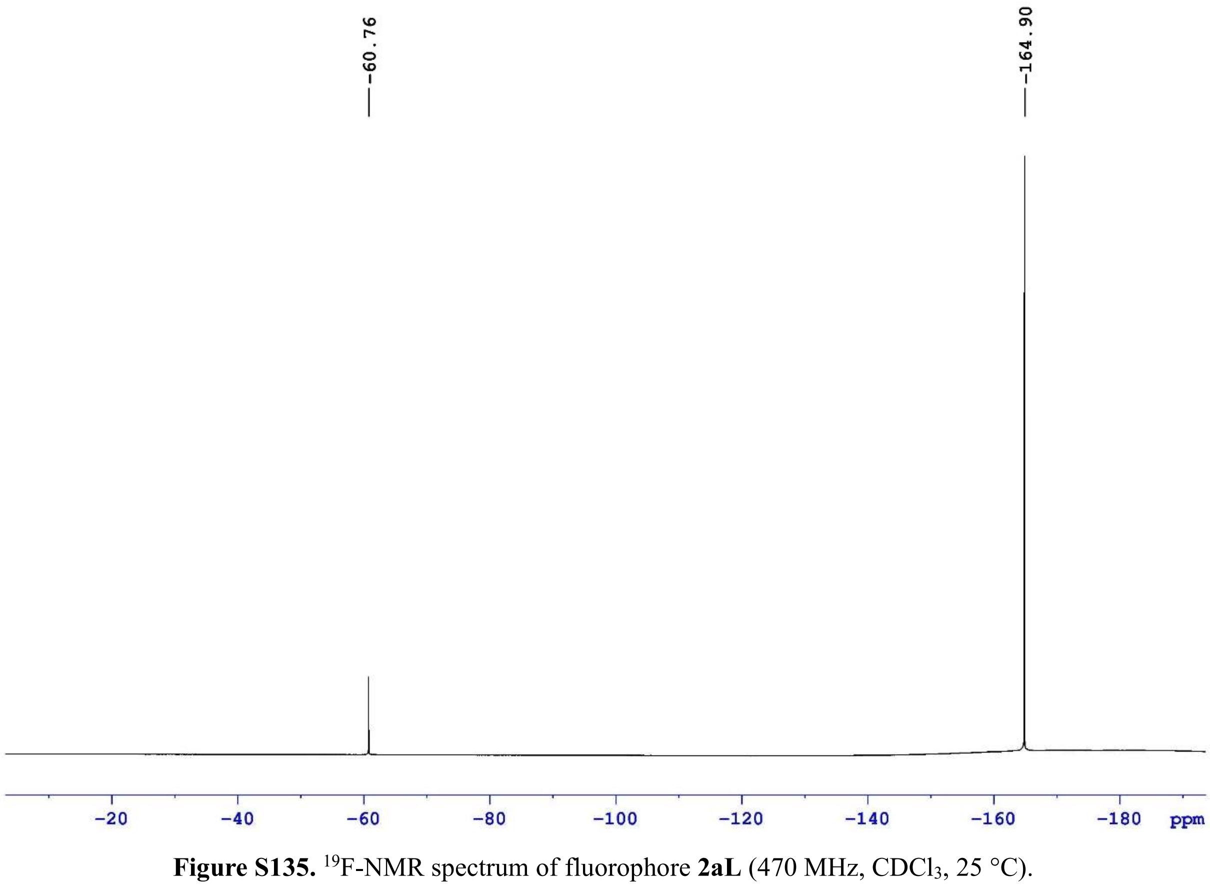

Figure S135. ^{19}F-NMR spectrum of fluorophore **2aL** (470 MHz, $CDCl_3$, 25 °C).

10.HR-MALDI-MS spectra

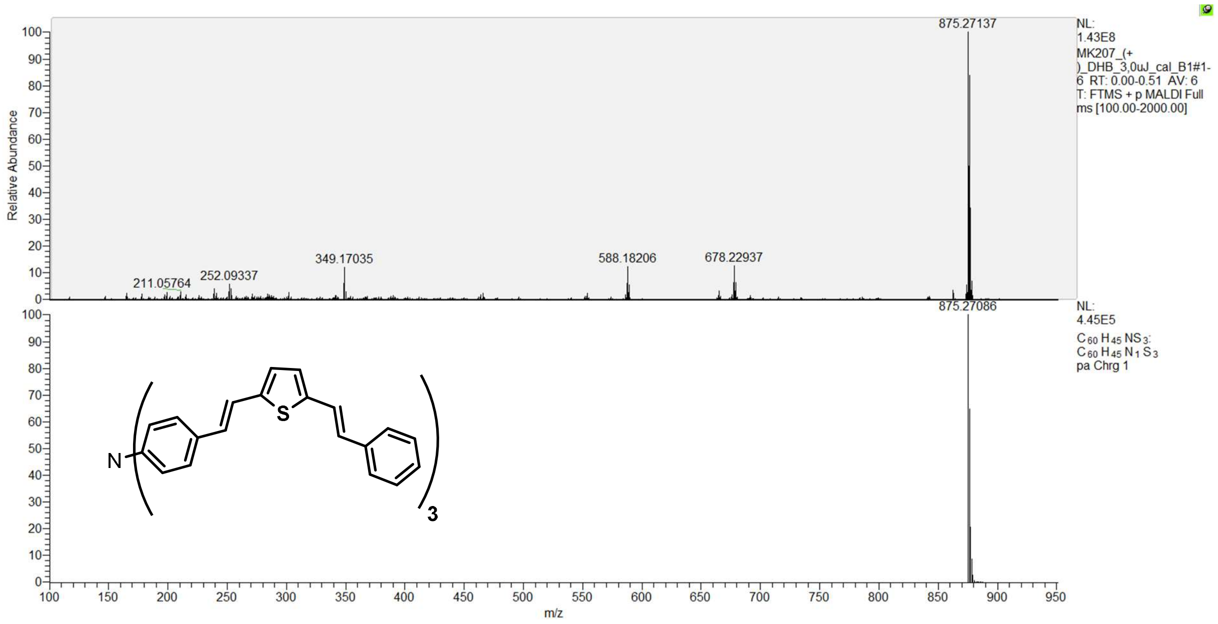

Figure S136. Measured (up) and predicted (down) HR-MALDI-MS spectra of fluorophore **PM1** $[M]^{+}$.

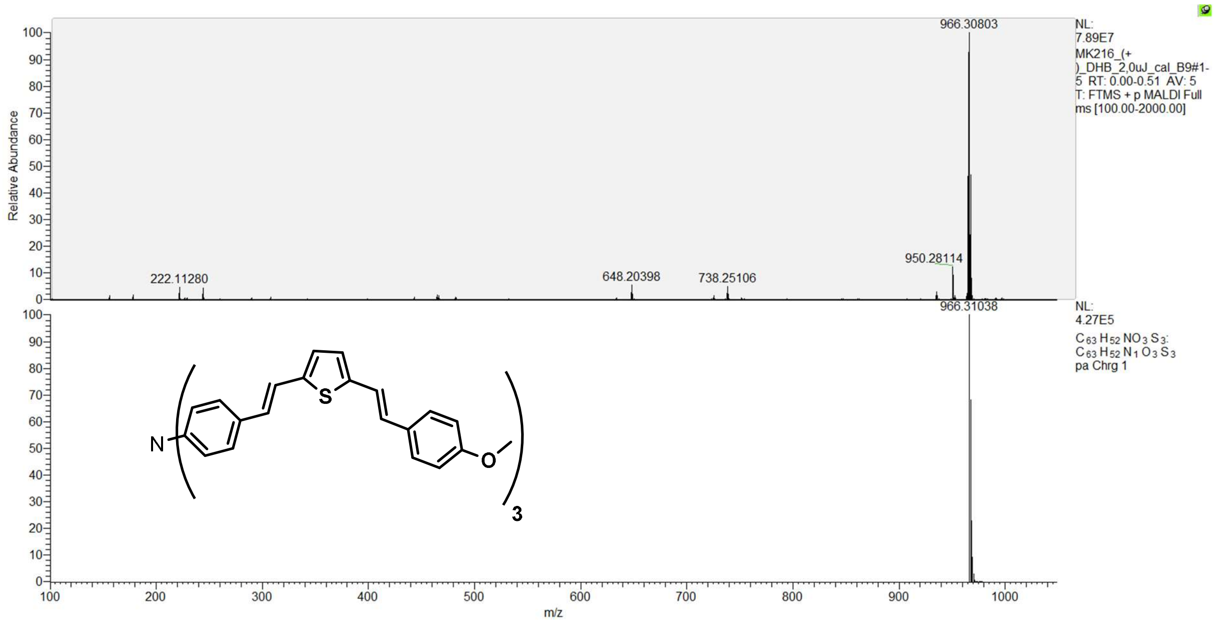

Figure S137. Measured (up) and predicted (down) HR-MALDI-MS spectra of fluorophore **PM2** $[M+H]^{+}$.

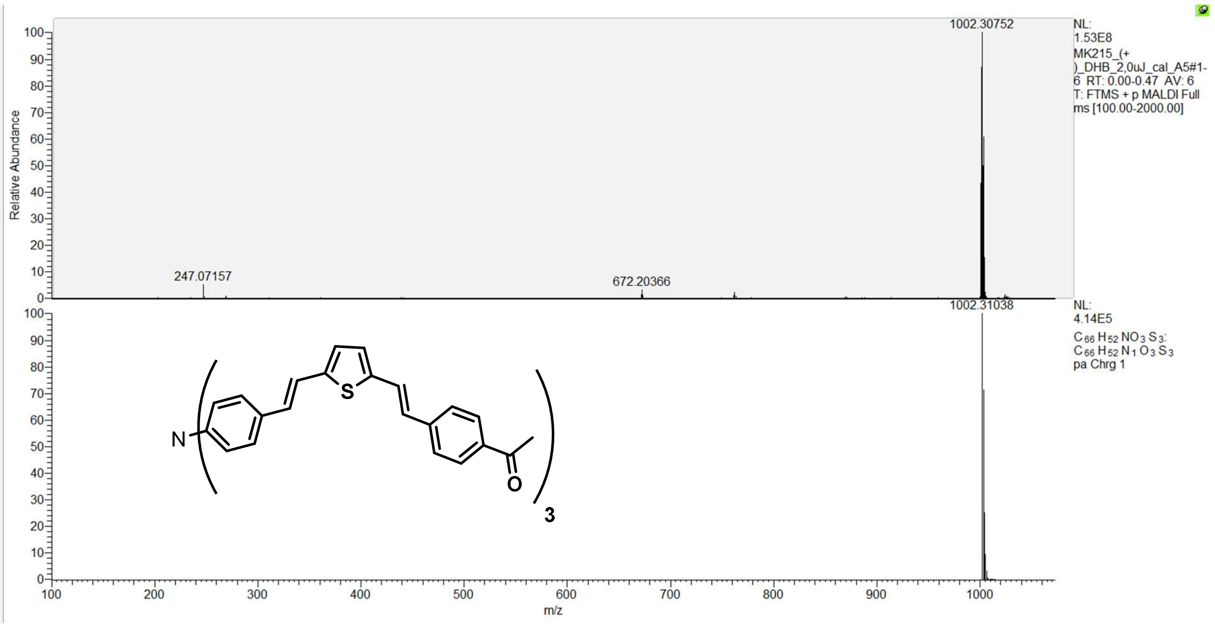

Figure S138. Measured (up) and predicted (down) HR-MALDI-MS spectra of fluorophore **PM3** $[M+H]^{+}$.

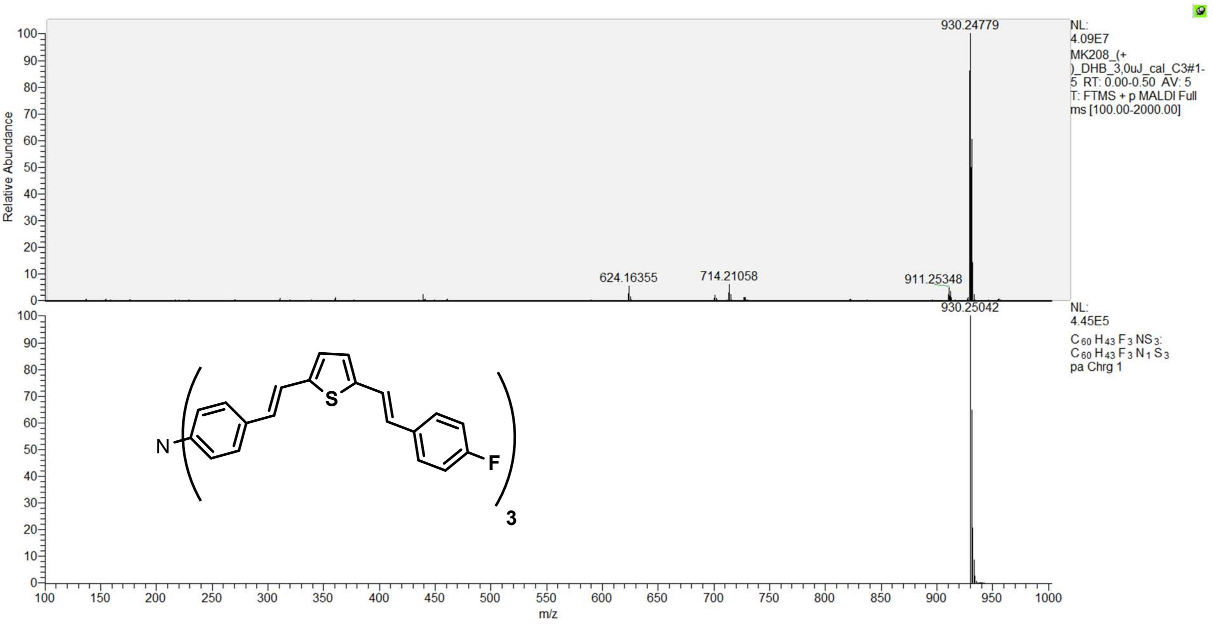

Figure S139. Measured (up) and predicted (down) HR-MALDI-MS spectra of fluorophore **1a** $[M+H]^{+}$.

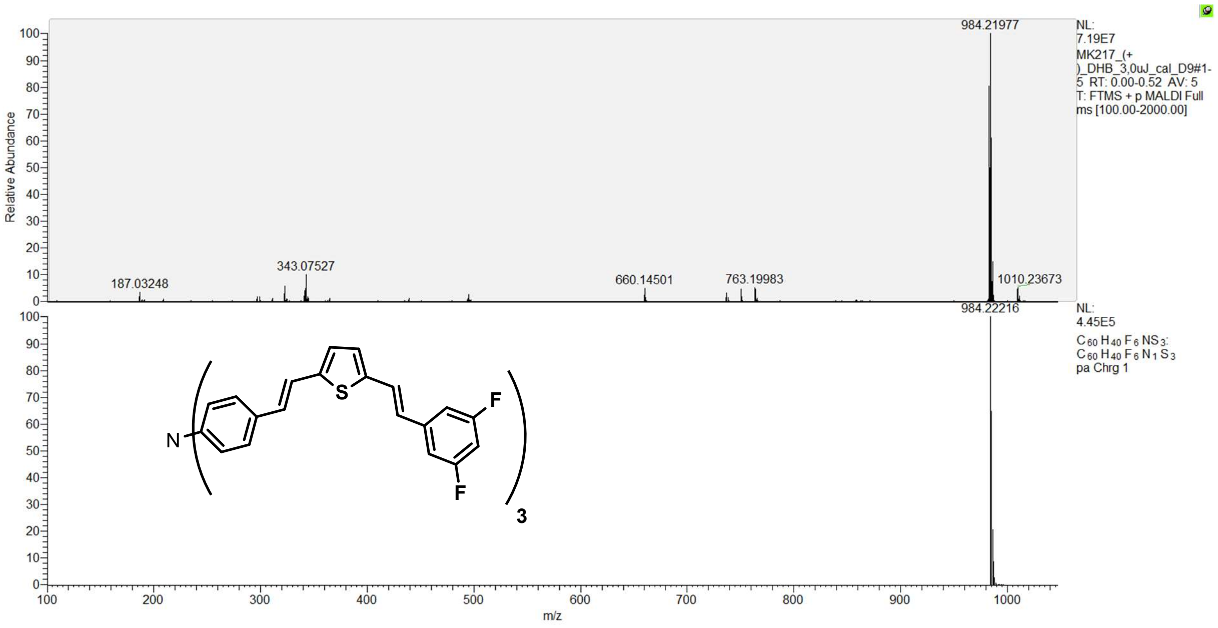

Figure S140. Measured (up) and predicted (down) HR-MALDI-MS spectra of fluorophore **1b** $[M+H]^+$.

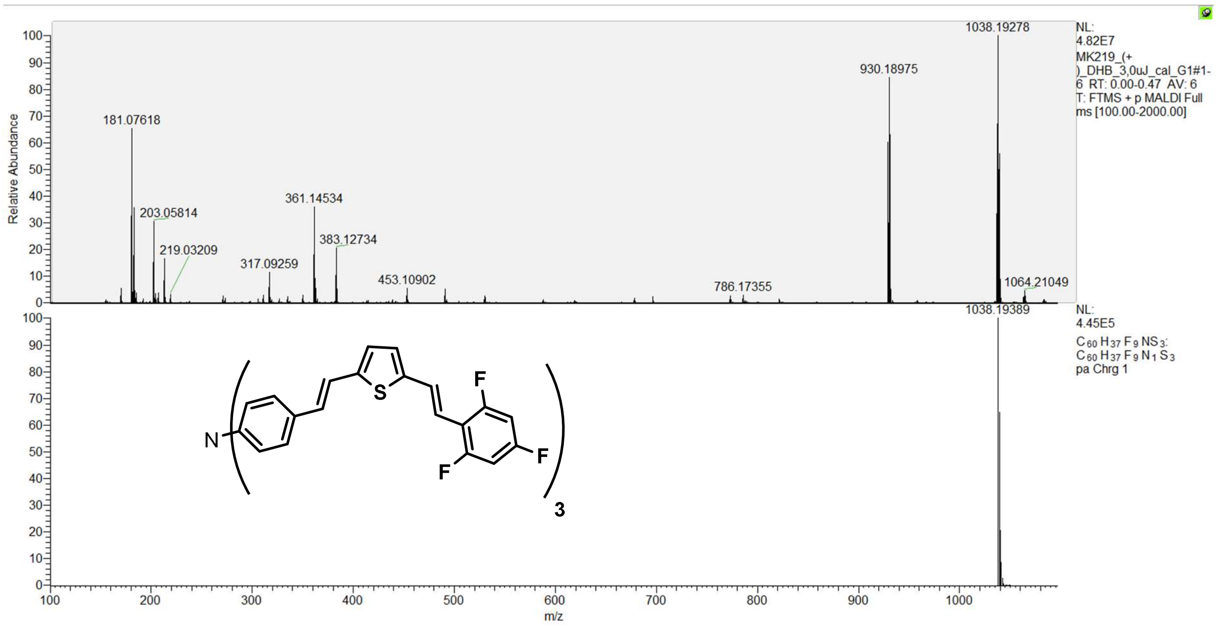

Figure S141. Measured (up) and predicted (down) HR-MALDI-MS spectra of fluorophore **1c** $[M+H]^+$.

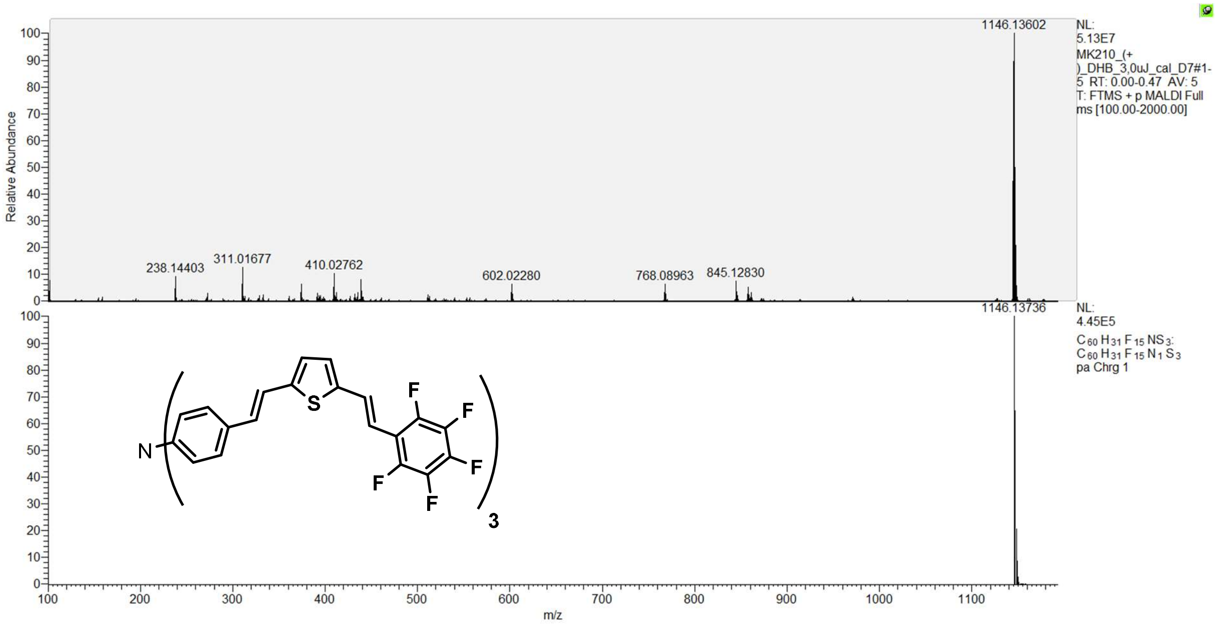

Figure S142. Measured (up) and predicted (down) HR-MALDI-MS spectra of fluorophore **1d** [M+H]$^+$.

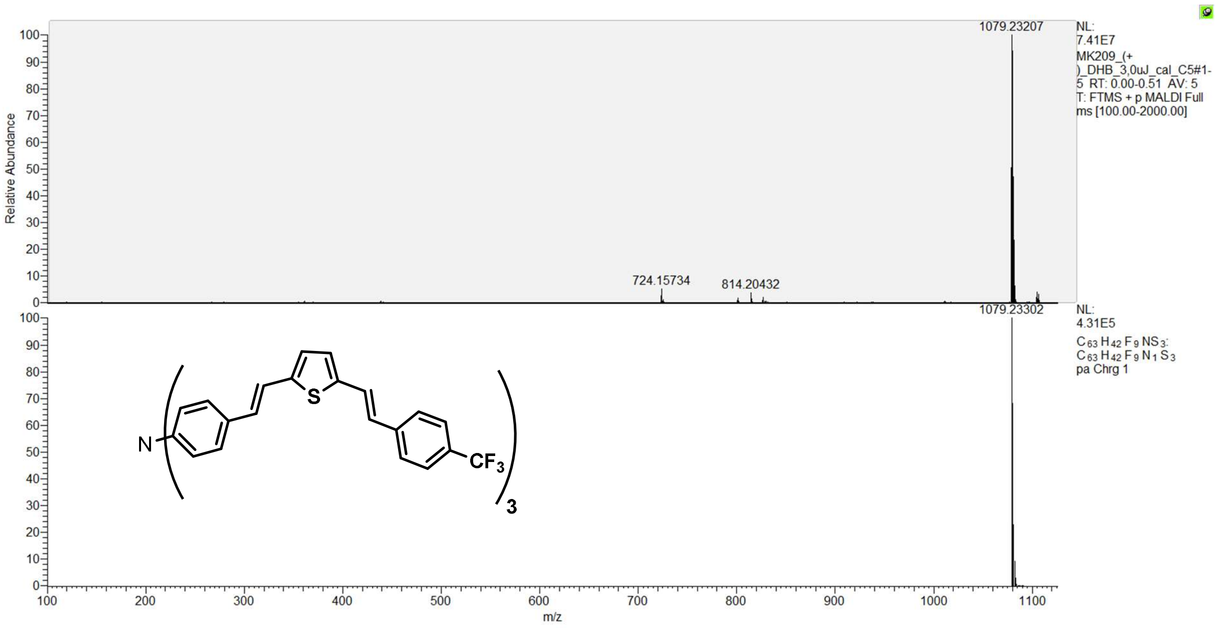

Figure S143. Measured (up) and predicted (down) HR-MALDI-MS spectra of fluorophore **2a** [M]$^+$.

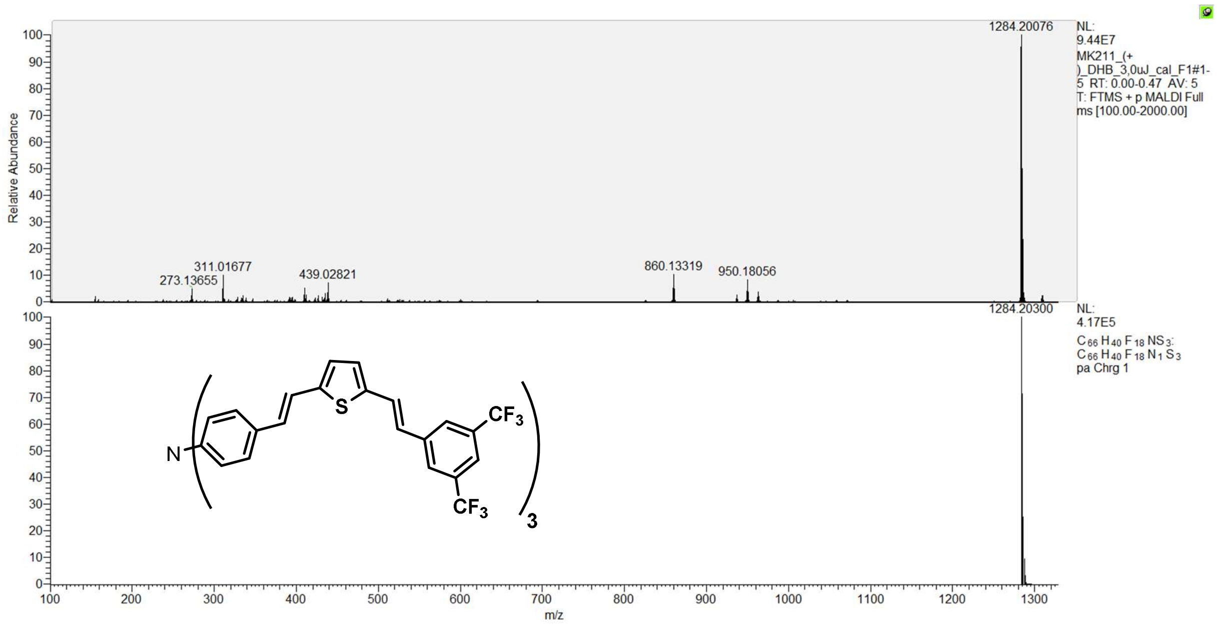

Figure S144. Measured (up) and predicted (down) HR-MALDI-MS spectra of fluorophore **2b** $[M+H]^+$.

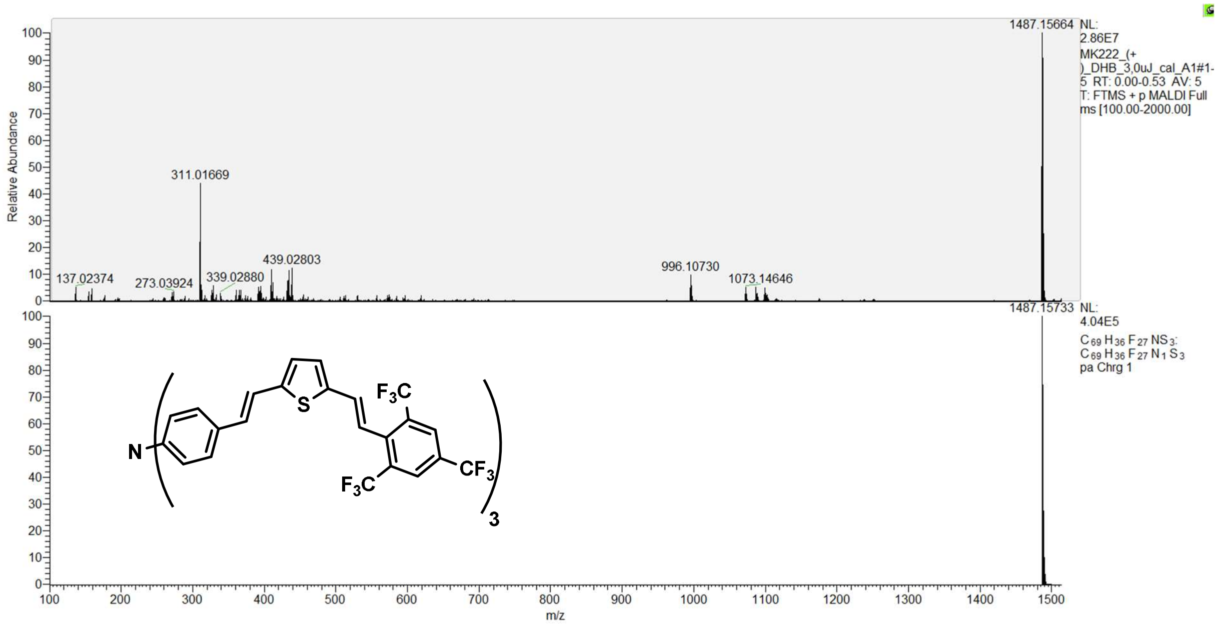

Figure S145. Measured (up) and predicted (down) HR-MALDI-MS spectra of fluorophore **2c** $[M]^+$.

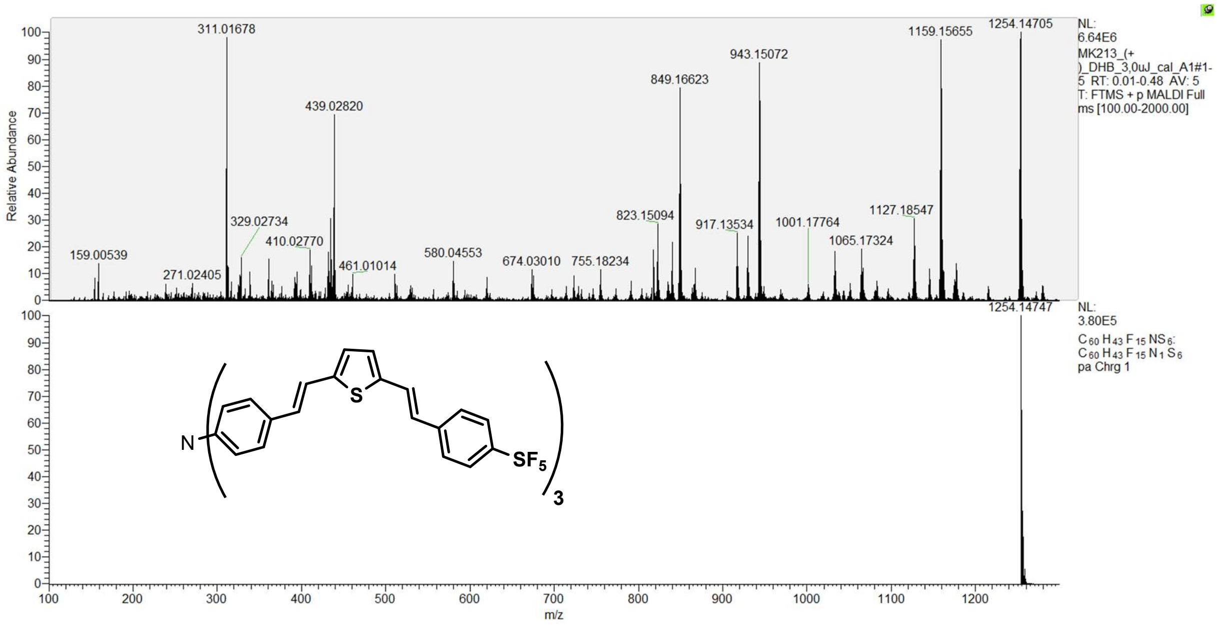

Figure S146. Measured (up) and predicted (down) HR-MALDI-MS spectra of fluorophore **2d** $[M+H]^+$.

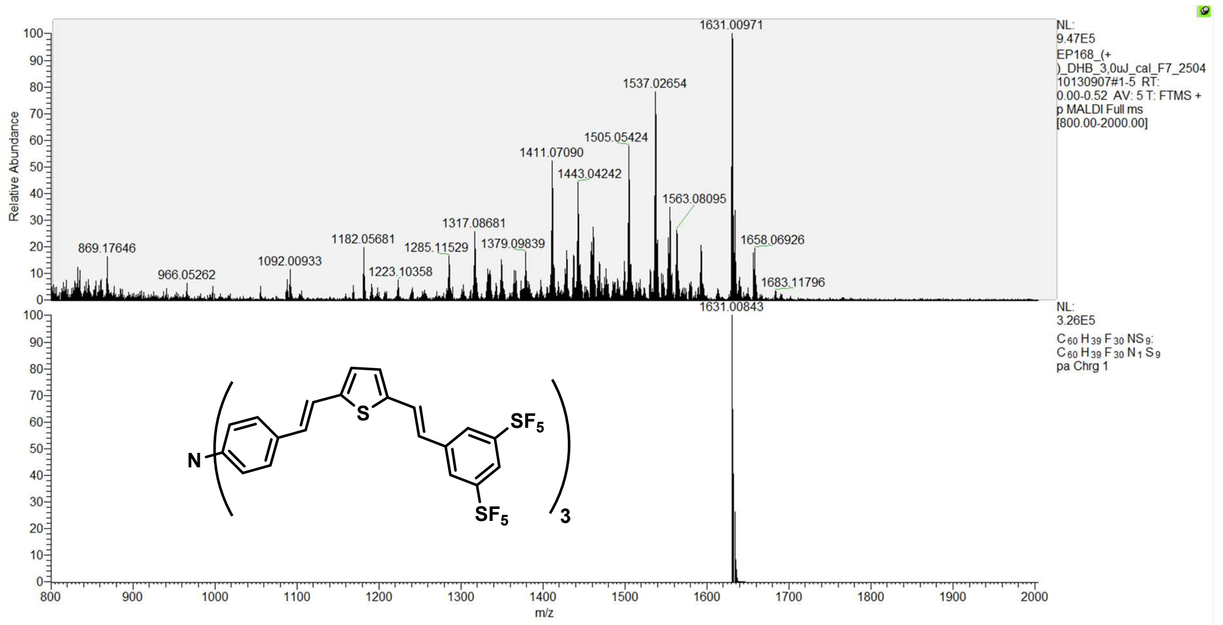

Figure S147. Measured (up) and predicted (down) HR-MALDI-MS spectra of fluorophore **2e** $[M]^+$.

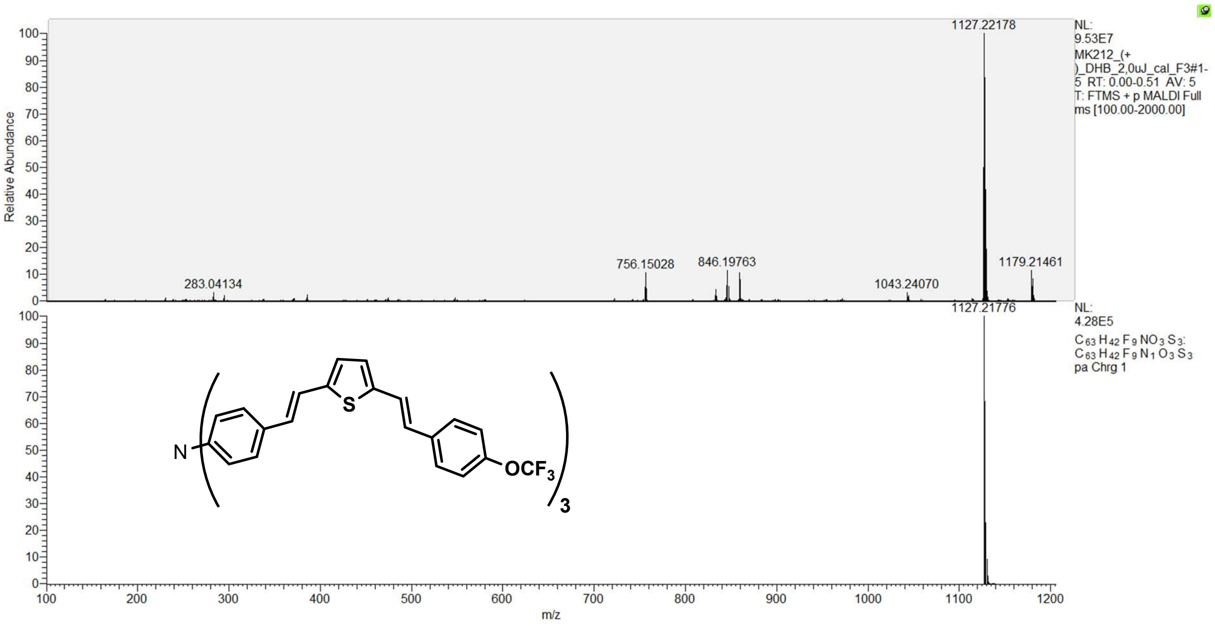

Figure S148. Measured (up) and predicted (down) HR-MALDI-MS spectra of fluorophore **3a** $[M]^+$.

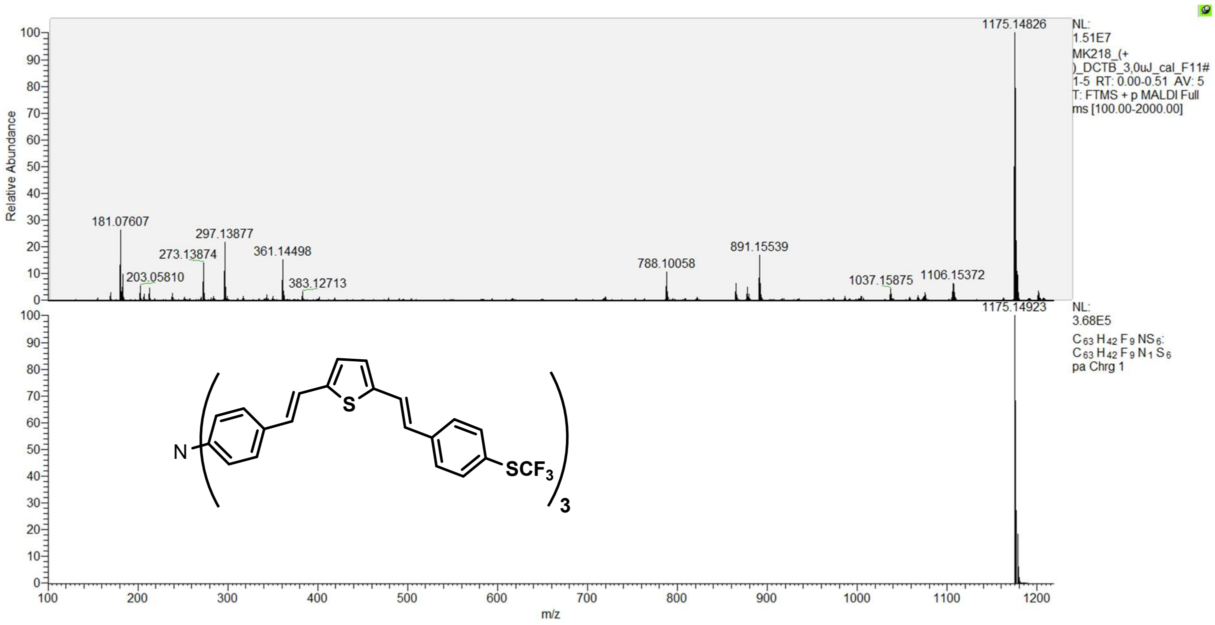

Figure S149. Measured (up) and predicted (down) HR-MALDI-MS spectra of fluorophore **3b** $[M]^+$.

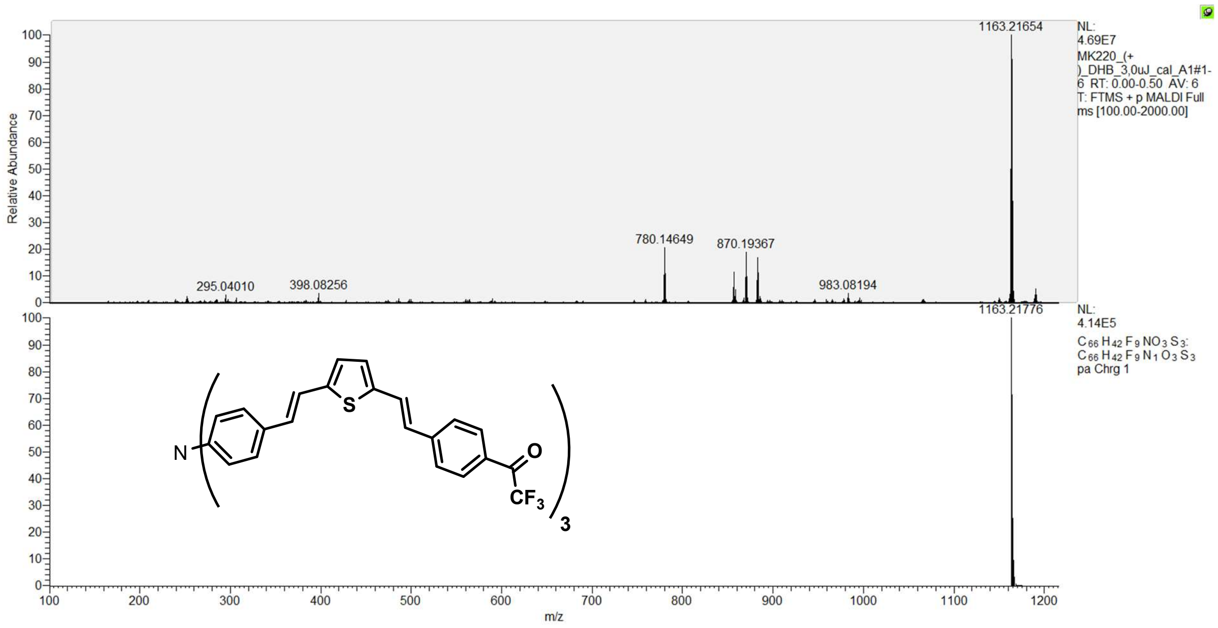

Figure S150. Measured (up) and predicted (down) HR-MALDI-MS spectra of fluorophore **4a** $[M]^+$.

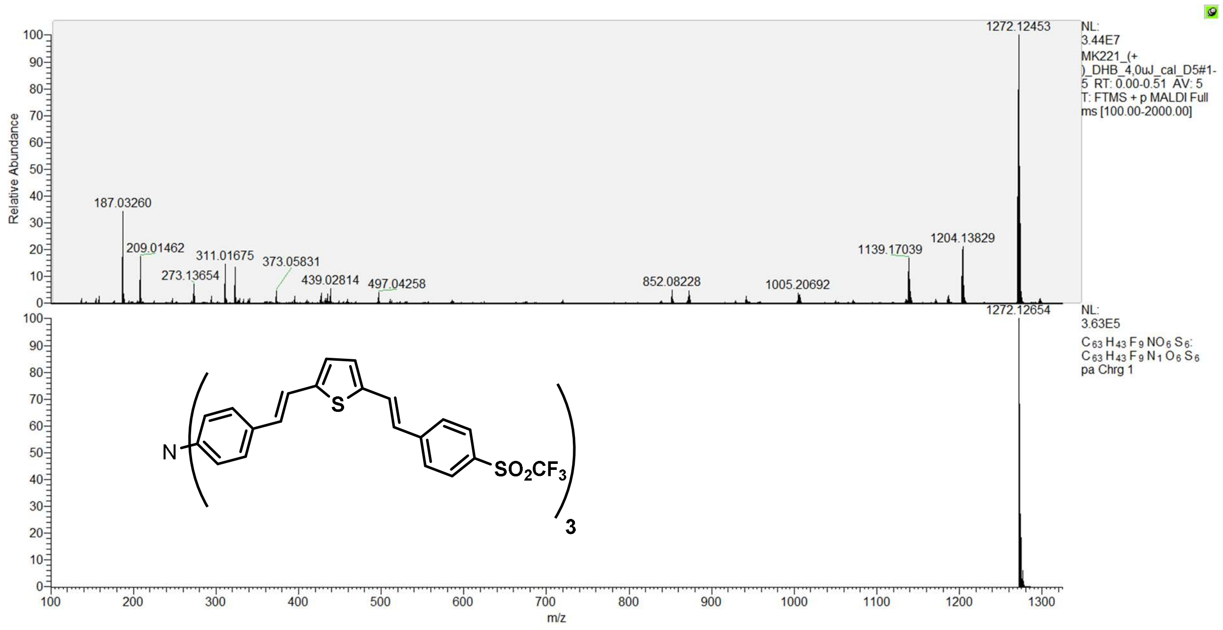

Figure S151. Measured (up) and predicted (down) HR-MALDI-MS spectra of fluorophore **4b** $[M+H]^+$.

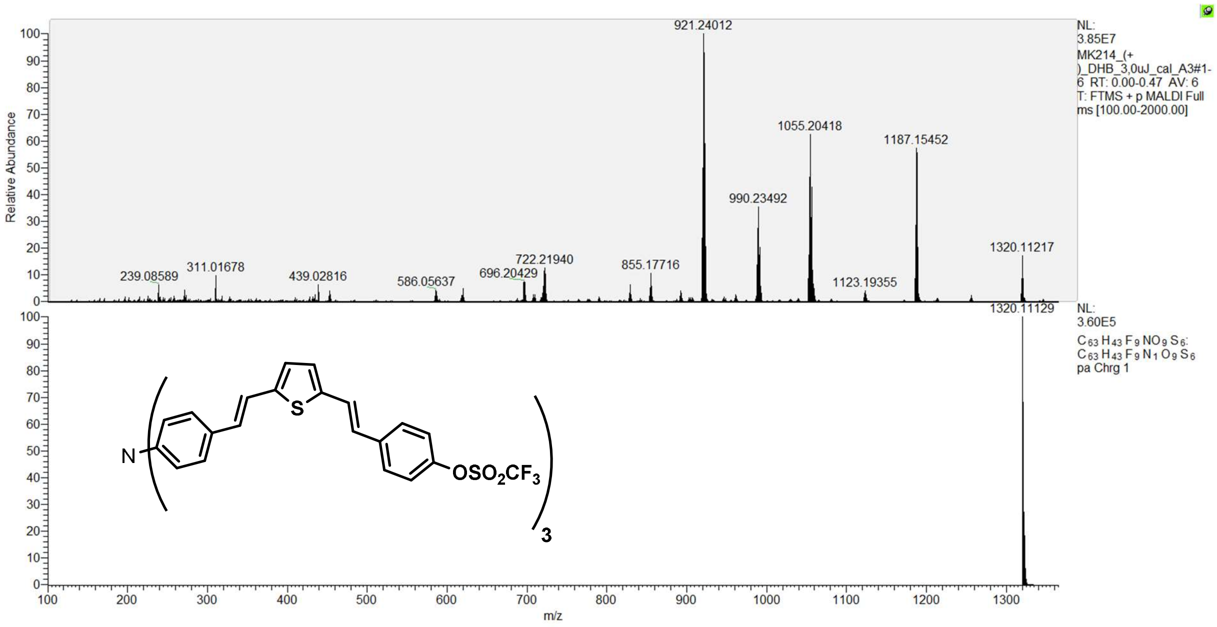

Figure S152. Measured (up) and predicted (down) HR-MALDI-MS spectra of fluorophore **4c** $[M+H]^+$.

11. Literature

[1] L. Porrès, O. Mongin, C. Katan, M. Charlot, T. Pons, J. Mertz, M. Blanchard-Desce, "Enhanced Two-Photon Absorption with Novel Octupolar Propeller-Shaped Fluorophores Derived from Triphenylamine" *Org. Lett.* **2004**, *6*, 47.

[2] B. L. Edelbach, B. M. Pharoah, S. M. Bellows, P. R. Thayer, C. N. Fennie, R. E. Cowley, P. L. Holland, "An Expedient Synthesis of 2,4,6-Tris(trifluoromethyl)aniline" *Synthesis* **2012**, *44*, 3595.

[3] S. Mori, N. Tsuemoto, T. Kasagawa, E. Nakano, S. Fujii, H. Kagechika, "Development of Boron-Cluster-Based Progesterone Receptor Antagonists Bearing a Pentafluorosulfanyl (SF_5) Group" *Chem. Pharm. Bull.* **2019**, *67,* 1278.

[4] A. M. Brouwer, "Standards for Photoluminescence Quantum Yield Measurements in Solution" *Pure Appl. Chem.* **2011**, *83*, 2213.

[5] M. Fakis, V. Petropoulos, P. Hrobárik, J. Nociarová, P. Osuský, M. Maiuri, G. Cerullo, "Exploring Solvent and Substituent Effects on the Excited State Dynamics and Symmetry Breaking of Quadrupolar Triarylamine End-Capped Benzothiazole Chromophores by Femtosecond Spectroscopy" *J. Phys. Chem. B* **2022**, *126*, 8532.

[6] K. Seintis, Ç. Şahin, I. Sigmundová, E. Stathatos, P. Hrobárik, M. Fakis, "Solvent-Acidity-Driven Change in Photophysics and Significant Efficiency Improvement in Dye-Sensitized Solar Cells of a Benzothiazole-Derived Organic Sensitizer" *J. Phys. Chem. C* **2018**, *122*, 20122.

[7] C. Xu, W. W. Webb, "Measurement of Two-Photon Excitation Cross Sections of Molecular Fluorophores with Data from 690 to 1050 nm" *J. Opt. Soc. Am. B* **1996**, *13*, 481.

[8] N. S. Makarov, M. Drobizhev, A. Rebane, "Two-Photon Absorption Standards in the 550–1600 nm Excitation Wavelength Range" *Opt. Express* **2008**, *16*, 4029.

[9] X. Zeng, C. Gong, H. Guo, H. Xu, J. Zhang, J. Xie, "Efficient Heterogeneous Hydroboration of Alkynes: Enhancing the Catalytic Activity by Cu(0) Incorporated $CuFe_2O_4$ Nanoparticles" *New J. Chem.* **2018**, *42*, 17346.

[10] A. Payet, B. Blondeau, J. B. Behr, J. L. Vasse, "Synthesis of 1,3-Disubstituted Cyclohexenes from Dienylethers: Via Sequential Hydrozirconation/Deoxygenative Cyclisation" *Org. Biomol. Chem.* **2019**, *17*, 798.

[11] M. Wu, C. Yan, D. Zhuang, R. Yan, "Metal-Free C-S Bond Formation in Elemental Sulfur and Cyclobutanol Derivatives: The Synthesis of Substituted Thiophenes" *Org. Lett.* **2022**, *24*, 5309.

[12] K. Itami, T. Nokami, Y. Ishimura, K. Mitsudo, T. Kamei, J.-I. Yoshida, "Diversity-Oriented Synthesis of Multisubstituted Olefins through the Sequential Integration of Palladium-Catalyzed Cross-Coupling Reactions. 2-Pyridyldimethyl(vinyl)silane as a Versatile Platform for Olefin Synthesis" *J. Am. Chem. Soc.* **2001**, *123*, 11577.

[13] M. H. Aukland, F. J. T. Talbot, J. A. Fernández-Salas, M. Ball, A. P. Pulis, D. J. Procter, "An Interrupted Pummerer/Nickel-Catalysed Cross-Coupling Sequence" *Angew. Chem. Int. Ed.* **2018**, *57*, 9785.

[14] J. Qu, C. T. Cao, C. Cao, "Determining the Excited-State Substituent Constants of Furyl and Thienyl Groups" *J. Phys. Org. Chem.* **2018**, *31*, e3799.

[15] A. Vasseur, J. Muzart, J. Le Bras, "Dehydrogenative Heck Reaction of Furans and Thiophenes with Styrenes under Mild Conditions and Influence of the Oxidizing Agent on the Reaction Rate" *Chem. Eur. J.* **2011**, *17*, 12556.

[16] M. Fecková, M. Klikar, C. Vourdaki, I. Georgoulis, O. Pytela, S. Achelle, Z. Růžičková, M. Fakis, P. Beier, F. Bureš, "Selective Employment of Electronic Effects of the Pentafluorosulfanyl Group across Linear and Tripodal Push-Pull Chromophores with Two-Photon Absorption" *Mater. Adv.* **2025**, *6*, 5713.

[17] Z. Shi, J. Davies, S. H. Jang, W. Kaminsky, A. K. Y. Jen, "Aggregation Induced Emission (AIE) of Trifluoromethyl Substituted Distyrylbenzenes" *Chem. Commun.* **2012**, *48*, 7880.

[18] L. U. Colmenares, R. S. H. Liu, "^{19}F NMR Evidence for Restricted Rotation of the Retinyl Chromophore in Doubly Labeled Visual Pigment Analogs" *J. Am. Chem. Soc.* **1992**, *114*, 6933.

[19] R. Chen, L. U. Colmenores, J. R. Thiel, R. S. I. Liu, "Spiral Hexatrienes. The Hindered cis Isomer of Mini-carotene-3 and Hexakis-(2,2',4,4',6,6'-trifluoromethyl)stilbene" *Tetrahedron Lett.* **1994**, *35*, 7177.

[20] L. U. Colmenares, R. S. H. Liu, "Fluorinated Phenyirhodopsin Analogs. Binding Selectivity, Restricted Rotation and ^{19}F-NMR Studies" *Tetrahedron*, **1996**, *52*, 109.

[21] G. M. Sheldrick, SHELXL-97, Program for Crystal Structure Refinement, University of Göttingen, Göttingen (Germany), **2008**.

[22] M. Klikar, I. V. Kityk, D. Kulwas, T. Mikysek, O. Pytela, F. Bureš, "Multipodal Arrangement of Push-Pull Chromophores: a Fundamental Parameter Affecting Their Electronic and Optical Properties" *New J. Chem.* **2017**, *41*, 1459.

[23] C. Hansch, A. Leo, R. W. Taft, "A Survey of Hammett Substituent Constants and Resonance and Field Parameters" *Chem. Rev.* **1991**, *91*, 165.

[24] F. Bureš, O. Pytela, F. Diederich, "Solvent Effects on Electronic Absorption Spectra of Donor-Substituted 11,11,12,12-Tetracyano-9,10-anthraquinodimethanes (TCAQs)" *J. Phys. Org. Chem.* **2009**, *22*, 155.

[25] M. J. Frisch, G. W. Trucks, H. B. Schlegel, G. E. Scuseria, M. A. Robb, J. R. Cheeseman, G. Scalmani, V. Barone, G. A. Petersson, H. Nakatsuji, X. Li, M. Caricato, A. V. Marenich, J. Bloino, B. G. Janesko, R. Gomperts, B. Mennucci, H. P. Hratchian, J. V. Ortiz, A. F. Izmaylov, J. L. Sonnenberg, D. Williams-Young, F. Ding, F. Lipparini, F. Egidi, J. Goings, B. Peng, A. Petrone, T. Henderson, D. Ranasinghe, V. G. Zakrzewski, J. Gao, N. Rega, G. Zheng, W. Liang, M. Hada, M. Ehara, K. Toyota, R. Fukuda, J. Hasegawa, M. Ishida, T. Nakajima, Y. Honda, O. Kitao, H. Nakai, T. Vreven, K.

Throssell, J. A. Montgomery Jr., J. E. Peralta, F. Ogliaro, M. J. Bearpark, J. J. Heyd, E. N. Brothers, K. N. Kudin, V. N. Staroverov, T. A. Keith, R. Kobayashi, J. Normand, K. Raghavachari, A. P. Rendell, J. C. Burant, S. S. Iyengar, J. Tomasi, M. Cossi, J. M. Millam, M. Klene, C. Adamo, R. Cammi, J. W. Ochterski, R. L. Martin, K. Morokuma, O. Farkas, J. B. Foresman, D. J. Fox, Gaussian®16W Software Package, Program for the Theoretical (TD-)DFT Calculations, Wallingford (USA), **2016**.

[26] O. Pytela, OPChem, (n.d.)., Program for Visualization of Structures, University of Pardubice, Pardubice (Czechia), 2023.

[27] O. Pytela, OPStat, (n.d.)., Program for Mathematical and Statistical Calculations, University of Pardubice, Pardubice (Czechia), 2018.
